# Supplementary material for: Discovery and pharmacophoric characterization of chemokine network inhibitors using phage-display, saturation mutagenesis and computational modelling
Source: Nat Commun. 2023 Sep 16;14:5763. doi: 10.1038/s41467-023-41488-z (PMC10505172; doi:10.1038/s41467-023-41488-z)
Supplement: Supplementary file 4 — Source Data [file 41467_2023_41488_MOESM4_ESM.zip › SourceData/scripts/xoctet_20230808081637.tar!/xoctet/metadata/data_analysis_octet.pdf]

Octet® System Data Analysis User Guide

Release 11.0

Pall ForteBio LLC
47661 Fremont Boulevard
Fremont, CA 94538
888.OCTET-QK
650.322.1360
www.fortebio.com

Table of Contents

Chapter 1:
Welcome. 5

About Octet Systems .6

What's New in Octet Data Acquisition Software, Release 10.0. 7

What's New in Octet Data Analysis Software, Release 9.0 7

What's New in Octet Data Analysis Software, Release 8.2 7

What's New in Octet System Data Analysis Software, Release 8.1 .9

What's New in Octet System Data Analysis Software, Release 8.0 10

Conventions and Symbols Used in This Guide 11

Pall ForteBio Technical Support. 12

Chapter 2:
Getting Started. 13

Launching the Octet System Data Analysis Software 14

Main Menu 15

Chapter 3:
21 CFR Part 11 Compliance. 19

Octet System 21 CFR Part 11 Software Overview. 20

ForteBio GxP Server Module 21

Selecting a Server Location 22

Starting a User Session 25

Compliance Features. 27

Experiment and Method File Compliance 28

Verifying Digital Signatures. 28

Viewing the Data Settings Change History 30

Viewing the Audit Trail. 31

Changing Projects During a User Session 33

Changing the User Password. 34

Locking the Application. 34

Ending a User Session. 35

Chapter 4:
Quantitative Analysis 37

Working with Experiments 38

Loading an Experiment for Analysis . .38

Editing Experiments 40

Viewing Binding Curves. 48

Closing Experiments 52

Analyzing Data 52

Analyzing Binding Data. 52

Specifying Analysis Settings. 53

Working with Analyzed Data 60

Saving Standards Data. 68

Saving Analysis Settings. 68

Processing Batch Quantitation Analysis .68

Creating a Settings_DataAnalysis.ini File 69

Selecting Experiments and Running the Batch Analysis 70

Exporting Data 72

Saving Raw Data 72

Saving a Quantitation Results Report 73

Masking Data 75

Multi-Step Advanced Quantitation Experiments 75

Chapter 5:
Basic Kinetics Analysis 79

Working with Experiments. 80

Starting a Basic Kinetics Experiment. 80

Loading an Experiment for Analysis . 80

Analyzing Binding Data. 84

Editing Experiments. 85

Closing Experiments 88

Working with Raw Data 89

Viewing Raw Data 89

Exporting Raw Data 92

Quantitating Raw Data 92

Processing Kinetic Data. 95

Step 1: Sensor Selection 95

Step 2: Reference Subtraction 98

Step 3: Align Y Axis 103

Step 4: Interstep Correction 104

Step 5: Process 105

Step 6: Viewing Results. 106

Step 7: Saving Results and/or Processing Parameters 112

Kinetics Analysis. 113

Curve Fitting Analysis 114

Steady State Analysis 119

Processing Batch Kinetics Analysis 120

Creating a Kinetic Settings_DataAnalysis.ini File. 120

Creating a Kinetic Settings_WellInfo.xml File (Optional) 121

Creating a Kinetic Settings_TableInfo.xml File (Optional) 121

Selecting Experiments and Running the Batch Analysis 122

Kinetics Analysis Results 124

Fitting View and Residual View. 124

Individual View 127

Grouping Results for Viewing 129

Selecting Data for Viewing. 130

Analysis Results Table. 131

Working with the Analysis Results Table 133

Searching Analysis Results 134

Color-Coding Data 137

Sorting Analysis Results. 140

Working with Graphs. 142

X-Y Graphs 142

Iso-Affinity Graphs. 143

Steady State Analysis Graphs 144

Data Export Options 144

Masking Data. 145

Generating a Report. 146

Changing the Column Order in a Saved Report. 147

Experiment Summary Options 148

Processing Options 149

Analysis Options. 152

Epitope Binning 153

Appendix A:
Using Octet384 Systems with an Automation Interface 165

Automation Interface Overview 166

Design of the Automation Interface. . . . 166

Automation Interface Control Setup 167

Analysis Automation API 167

Appendix B:
21 CFR Part 11 Software Administrator Options 171

Installing the Data Acquisition 21 CFR Part 11 Software 172

Installing the Data Analysis 21 CFR Part 11 Software 174

Installing the ForteBio GxP Server Module177

Database Backup and GxP Server Module Upgrade 182

Step 1: Backup the Version 8.0 Database 182

Step 2: Upgrade to GxP FBServer Module v8.2. 182

Administrator Account Setup 182

Starting an Administrator User Session 187

Accessing Administrator Options 190

Administrator Tabs 191

User Account Administration. 192

Group Administration. 197

Project Administration 200

Administrator Constants. 201

Event Log. 204

Accessing the ForteBio GxP Server Module Directly 206

Restarting the Pall ForteBio GxP Server Module 209

CHAPTER 1:
Welcome

About Octet Systems 6

What's New in Octet Data Acquisition Software, Release 10.0 7

What's New in Octet Data Analysis Software, Release 9.0 7

What's New in Octet Data Analysis Software, Release 8.2 7

What's New in Octet System Data Analysis Software, Release 8.1 9

What's New in Octet System Data Analysis Software, Release 8.0 10

Conventions and Symbols Used in This Guide 11

Pall ForteBio Technical Support 12

ABOUT OCTET SYSTEMS

Octet systems enable real-time quantitation or kinetic characterization of biomolecular interactions. Each system includes:

- Octet instrument
- Computer
- Hardware accessories
- Octet software modules—Data Acquisition and Data Analysis

For more details on the Data Acquisition software, see the *Octet System Data Acquisition User Guide*.

***Table 1-1:** Octet System Software Functions*

Octet Software	*Functions*
Data Acquisition	• Define quantitation or kinetic experiments and save them for future use.<br>• Define custom assays.<br>• Run experiments and acquire binding data.<br>• View and save binding data.
Data Analysis	• Analyze binding data and view analysis results.<br>• Export or copy analysis results.<br>• Generate reports of quantitation or kinetic results.

For information on preparing samples for quantitation or kinetics experiments, please see the appropriate Pall ForteBio biosensor product instructions.

WHAT'S NEW IN OCTET DATA ACQUISITION SOFTWARE, RELEASE 10.0

1. Support for the Octet RED96e system

WHAT'S NEW IN OCTET DATA ANALYSIS SOFTWARE, RELEASE 9.0

1. Our New Data Analysis HT 9.0 software is now available as part of the Octet Analysis software suite, and installs with Octet System Data Analysis software. You can use it for advanced epitope binning analysis on multiple plates, multiple sensor trays and multiple experiments.
2. Support for the Octet K2 system.
3. The new Mask Data feature lets you make a copy of your data folder with all proprietary info such as sample ID, sample info, sensor type and sensor info hidden, or 'masked', in both the .fmf and .frd files. For more information, see page 75 for quantitative analysis or page 145 for kinetics analysis.

***NOTE:** The Mask Data feature is not available in Octet Data Analysis 21 CFR Part 11 software.*

4. You can now assign % K_D1 and K_D2 contributions to interactions in the 2:1 (HL) model for kinetic analysis curve fitting (see page 116).
5. The Order Columns dialog is now available again in the Data Table on the Results Tab for quantitative analysis (see page page 62).
6. Values in data tables can now be edited directly by clicking on a table cell and typing into the cell. See page 42 for quantitative analysis or page 87 for kinetics analysis.
7. For kinetic experiments, all reference wells and sensors assigned in the Octet Data Acquisition Software now also display as reference wells and sensors in the Octet Data Analysis Software. They no longer need to be reassigned.

WHAT'S NEW IN OCTET DATA ANALYSIS SOFTWARE, RELEASE 8.2

***NOTE:** The new features listed below are applicable to version 8.2 of Octet Data Analysis 21 CFR Part 11 software only.*

1. Now whenever a raw data or results report is exported, the contents of the .xls file are locked and cannot be changed.
2. New Audit Trail options:

- The Audit Trail now includes the option to display experiment-specific events in addition to the existing project- and machine-specific events (see page 32). To view audit logs for a specific experiment, click on the **Experiment** drop-down list and select an entry:

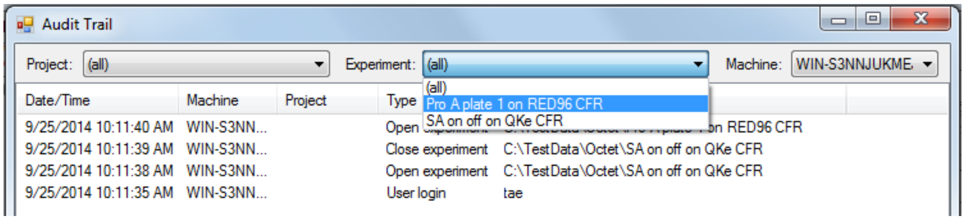

Figure 1-1: *Selecting an Experiment in the Audit Trail*

- You can now print the Audit Trail (see page 33).
- All actions related to data analysis, processing and inclusion or exclusion of data are now also logged in the Audit Trail.

3. The file extension for the data analysis settings file, Settings_DataAnalysis.ini, was changed from .ini to .fsd. The full file name will now be Settings_DataAnalysis.fsd.
4. Electronic signatures are now also added to the data analysis settings file, Settings_DataAnalysis.fsd, and non-CFR standards data (.fsc) files (see page 28).
5. Changes made to data analysis settings are now logged in a Settings Change History which is archived within the experiment folder (see page page 30).
6. Exported raw data and results reports now include the software name, version and export date and time on the Summary tab of the report.

For Software Administrators:

7. Octet Data Acquisition 21 CFR 11 software version 8.2 requires version 8.2 of the GxP Server module. Administrators should also install this with version 8.2 software.
8. Octet Data Acquisition 21 CFR 11 software version 8.2 uses a new database schema which is automatically installed and configured during software installation.

***IMPORTANT**: It is strongly recommended that you make a backup copy of the existing database before installing and upgrading to GxP FBServer Module v8.2. Please see page 182 for instructions.*

9. The same GxP Server module can now be used both for Octet and BLItz® systems. This is available for GxP Server module versions 8.2 and higher only, which will now also log events in BLItz Pro software for BLItz instruments.
10. New Event Log options (see page 204):

- Machine names logged will also include BLItz instrument names when these instruments use the same GxP Server module as other Octet instruments. This is available when using GxP Server module version 8.2 and higher only.
- A new Software field is available that displays which software the event was logged in. This is available for GxP Server module version 8.2 and higher only, and includes logging of Octet Data Acquisition, Octet Data Analysis and BLItz Pro software events.

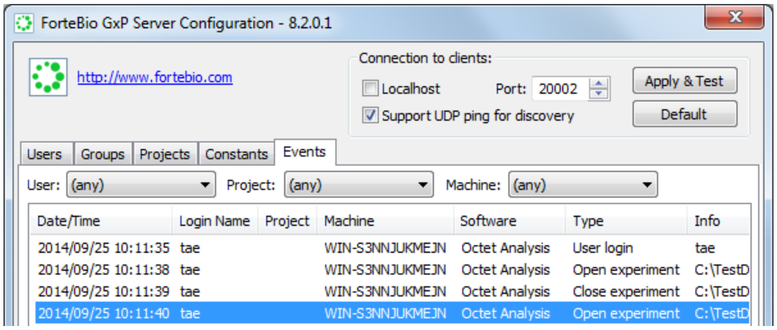

Figure 1-2: *Events Tab*

WHAT'S NEW IN OCTET SYSTEM DATA ANALYSIS SOFTWARE, RELEASE 8.1

1. Octet System Data Analysis software version 8.1 and higher are now compatible with 64-bit versions of Windows 7.
2. The new High Precision Streptavidin (SAX) Biosensor is available in the Sensor Type list for both quantitation and kinetics.
3. Cursors in the Analysis tab (Tab 3). You can now move two cursors (1 and 2) anywhere along the x-axis to get the time (in seconds) and nm response on the y-axis.

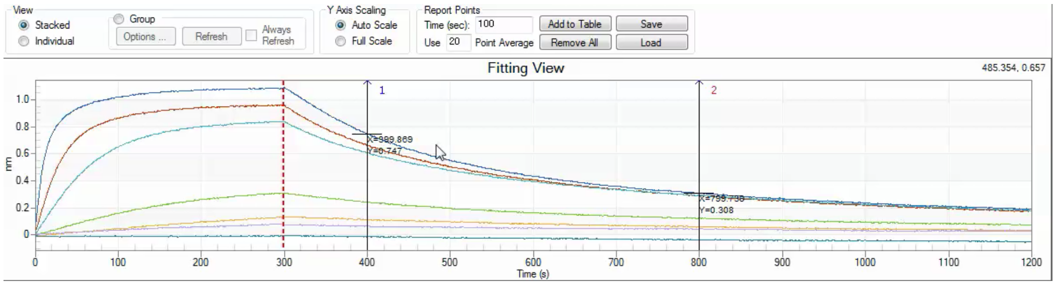

- Each cursor can be dragged along the x-axis by clicking on the arrow next to the cursor number (1 or 2) and then dragging it to the desired x-axis location.

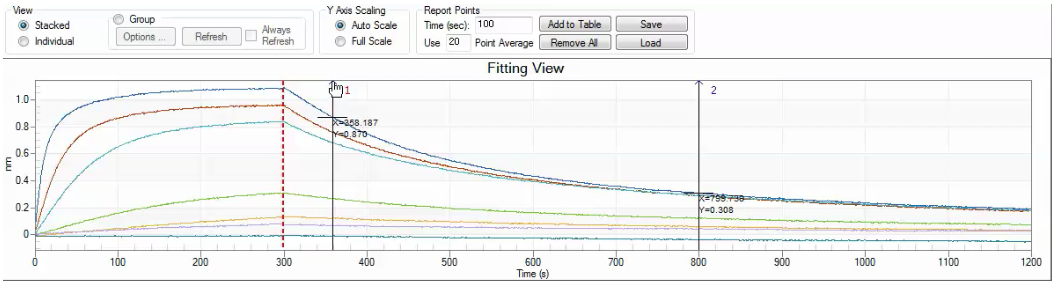

- You can also add the time and response data from the cursors to the data table. To do this, right click on the graph and select **Add Report Point at Cursor #**.

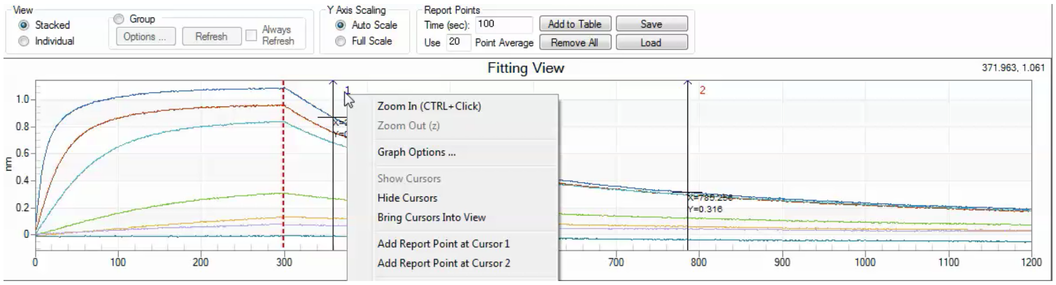

WHAT'S NEW IN OCTET SYSTEM DATA ANALYSIS SOFTWARE, RELEASE 8.0

The following features are new for the Octet System Data Analysis software, Release 8.0:

1. The Sample plate and the Reagent plate are now referred to as "Plate 1" and "Plate 2" in the software.
2. Support for the Octet HTX system. See "What you should know about the New Octet HTX System" on page 212 for more details on our newest system.
3. Data analysis options for the Octet HTX system:
    a. Kinetics analysis is the same on the Octet HTX system as it is on other Octet systems. See "Basic Kinetics Analysis" on page 79 for more information.
    b. Quantitative analysis is the same on the Octet HTX system as it is on other Octet systems. See "Quantitative Analysis" on page 37 for more information.
    c. For Multi-Step Advanced Quantitation experiments, see page 75.
    d. For Epitope Binning experiments, see page 153.
4. New data analysis options for Octet RED96, RED384, QK384 and QK^e systems:
    a. Multi-Step Advanced Quantitation experiments on page 75.
    b. Epitope Binning experiments on page 153.

CONVENTIONS AND SYMBOLS USED IN THIS GUIDE

NOTE: *Presents pertinent details on a topic. For example, general information, tips or alternate options.*

IMPORTANT: *Indicates the assay or procedure will not work if the guidelines provided are not properly followed.*

WARNING: *Informs the user that specific actions could cause irreversible consequences or damage.*

Table 3: *Octet Instrument Labels*

Symbol	Definition
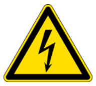	Electrical hazard
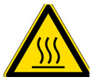	Heat/hot
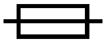	Fuse

PALL FORTEBIO TECHNICAL SUPPORT

You can contact Pall ForteBio technical support at:

Pall ForteBio LLC
47661 Fremont Boulevard
Fremont, CA 94538
USA
Tel: +1-650-322-1360
Fax: +1-650-322-1370
E-mail: fortebio_support@pall.com

CHAPTER 2:

Getting Started

Launching the Octet System Data Analysis Software 14

LAUNCHING THE OCTET SYSTEM DATA ANALYSIS SOFTWARE

NOTE: *The installation shall be performed by Pall ForteBio LLC personnel only.*

WARNING: *If the Octet system is not used as specified, injury to the user and/or damage to the instrument may result.*

NOTE: *Do not position the Octet instrument such that it is difficult to disconnect the power.*

NOTE: *For information about how to connect the Octet instrument to the computer, refer to the insert sheet that is provided with the Octet instrument.*

To launch the system and the Octet Data Analysis software:

1. Turn the Octet instrument on using the power switch located on the external electrical box.

NOTE: *The instrument requires a minimum of one-hour warm-up time. It is recommended that you leave the instrument on for a minimum of eight hours prior to use.*

2. Launch the Data Analysis software by double-clicking the Data Analysis desktop icon.

NOTE: *When using the CFR 11 version of the Octet System Data Analysis software, you are required to log in and start a user session before the software launches. For more information, refer to* *"Starting a User Session" on page 25.*

Launching the Octet System Data Analysis software application displays the **main screen** (Figure 2-1).

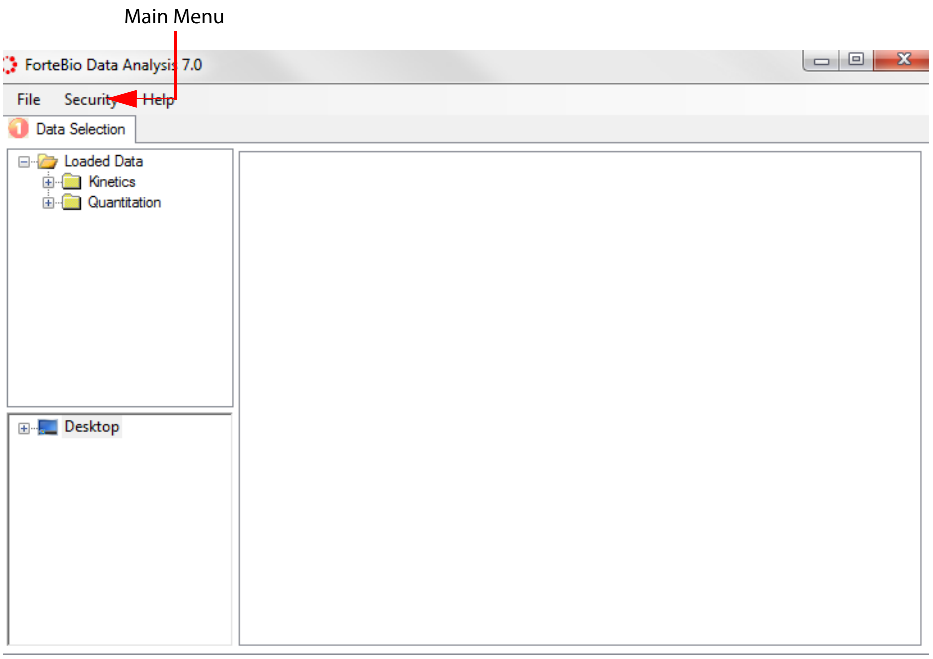

Figure 2-1: Main Screen for Data Analysis

Main Menu

The main menu is located in the upper left corner of the main screen (Figure 2-1). Menu options are described in this section.

Figure 2-1 displays the non-21 CFR Part 11 main menu; Figure 2-2 displays the main menu for the 21 CFR Part 11.

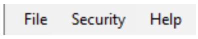

Figure 2-2: Main Menu—21 CFR Part 11 Version of the Data Analysis Software

File Menu

The **File** menu (Figure 2-3) allows users to open and save re-analyze, work with experiments in different modes, save reports, and set port options.

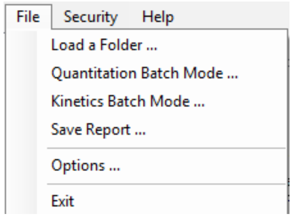

Figure 2-3: *File Menu*

Table 2-1: *File Menu Commands*

Menu Command	*Function*
**Load a Folder**	Loads an experiment method file (.frd).
**Quantitation Batch Mode**	Opens the Quantitation Batch Mode dialog box.
**Kinetics Batch Mode**	Opens the Kinetics Batch Mode dialog box.
**Save Report**	Saves all open report files.
**Options**	Allows you to determine the port automation options:<br>• TCP-IP with a *localhost* option<br>• Serial (RS-232)
**Exit**	Closes the application after prompting to save any changes.

NOTE: *When using the 21 CFR Part 11 version of the Octet System Data Analysis software, only 21 CFR Part 11-compliant experiments and re-analyze generated using the 21 CFR Part 11 version of the software can be opened. Files generated using the non-compliant version of the software or with a non-compliant system cannot be opened, and a message indicating this will be presented.*

Security Menu

The **Security** menu is only available in the 21 CFR Part 11 version of the Octet System Data Analysis software.

__NOTE:__ The __Security__ menu is only available in the CFR 11 version of the Octet System Data Analysis software. For complete details on menu options, refer to “Compliance Features” on page 27.

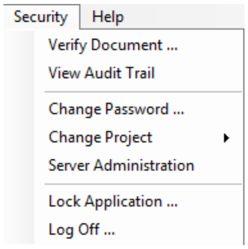

***Figure 2-4:** Security Menu*

***Table 2-2:** Security Menu Commands*

Menu Command	*Function*
**Verify Document**	Utility that tests if a method (.fmf) or data (.frd) file was created using a CFR version of the ForteBio software.
**View Audit Trail**	Displays the recorded events for CFR documentation. Events may be viewed by project or machine.
**Change Project**	Switches active projects or run experiment without a project title (“none”).
**Change Password**	Edits password for active user
**Server Administration**	Modifies settings for users, groups, projects and constants.
**Lock Application**	Disabled the Octet System Data Analysis software with a screen lock. A password is required to unlock the program.
**Logoff**	Exits the program. A password is required to log in again.

Help Menu

The **Help** menu provides access to software and instrument support information.

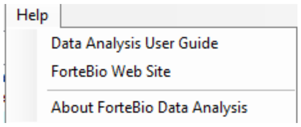

Figure 2-5: *Help Menu*

Table 2-3: *Help Menu Commands*

Menu Command	*Function*
**Data Analysis User Guide**	Opens the online *Octet System Data Analysis Software User Guide*.
**ForteBio Web Site**	Opens a web browser and displays the Pall ForteBio web page (www.fortebio.com).
**About ForteBio Data Analysis**	Displays software, user, and instrument information.

NOTE: *Clicking the Pall ForteBio logo (in the upper right corner of the main screen) also displays the About window.*

CHAPTER 3:

21 CFR Part 11 Compliance

Octet System 21 CFR Part 11 Software Overview 20

ForteBio GxP Server Module 21

Selecting a Server Location 22

Starting a User Session 25

Compliance Features 27

OCTET SYSTEM 21 CFR PART 11 SOFTWARE OVERVIEW

The Data Acquisition and Data Analysis software for Octet systems is available in an optional 21 CFR Part 11 version that enables users in GMP and GLP laboratories to comply with 21 CFR Part 11 regulations. This version of the software includes features such as user account management, audit trails and electronic signatures. In addition, the 21 CFR Part 11 version utilizes the Pall ForteBio GxP Server module to manage the information recorded during user sessions. This chapter explains how to use the Pall ForteBio GxP Server module, compliance features and administrative functions specific to the 21 CFR Part 11 versions of the Data Acquisition and Data Analysis software.

NOTE: *The 21 CFR Part 11 Data Analysis software only opens data files generated in CFR data acquisition. 21 CFR Part 11 files from software version 6.X and up are fully compatible with the 8.X 21 CFR Part 11 software.*

NOTE: *For details on how to install the Octet System Data Acquisition or Data Analysis software, see* *Appendix B, 21 CFR Part 11 Software Administrator Options on page 171*.

FORTEBIO GXP SERVER MODULE

***NOTE**: It is highly recommended that the ForteBio GxP Server Module not be installed on the computer connected to the Octet instrument. Instead, it should be configured on the administrator's computer that is connected to the server. This allows the administrator to control the users who have accounts and specific privileges in the 21 CFR Part 11 Data Acquisition software. Both the Octet system computer and the computer with the ForteBio GxP Server Module should be connected to the server.*

When the Data Acquisition or Data Analysis 21 CFR Part 11 software is launched, users are prompted to logon to the Pall ForteBio GxP Server module. This initiates a user session where all system, software and user events are recorded. During user sessions, the GxP Server module manages and stores this recorded information.User sessions are closed when the user logs out or a set period of inactivity is reached. A new user session is initiated each time a user accesses the software.

NOTES:

Version 8.2 of Data Acquisition and Data Analysis 21 CFR Part 11 software require version 8.2 of the GxP Server module. The software will automatically check the version of the GxP Server module in use and display a message if it is incompatible with version 8.2 software. Please contact your administrator to install version8.2 of the GxP Server module if this happens.

For details on how to install the ForteBio GxP Server module, see Appendix B, 21 CFR Part 11 Software Administrator Options on page 171.

The ForteBio GxP Server is required for 21 CFR Part 11.

The GxP Server can be installed in multiple locations with user selection of the employed copy at the launch of the Acquisition or Analysis software, although a single copy per network is recommended by ForteBio to ensure that all records are saved to one location.

SELECTING A SERVER LOCATION

NOTES:
Please contact your administrator to determine best location to use for the GxP Server module.

Once the GxP Server module host location is selected, this location should be used as the default selection for the user account. It does not need to be reselected each time a new user session is initiated.

Users must select the host location of the GxP Server module during the login process. The GxP server can be run on the local host computer where the Data Acquisition or Data Analysis software is installed or from a network location.

To select a server location:

NOTE: *You must select the host location of the GxP Server module during the login process. You can use the GxP Server on the local host computer where the Data Acquisition or Data Analysis software is installed, or from a network location.*

1. Launch the Data Analysis software by double-clicking the Data Analysis desktop icon.

   The **Login** dialog box displays (Figure 3-1).

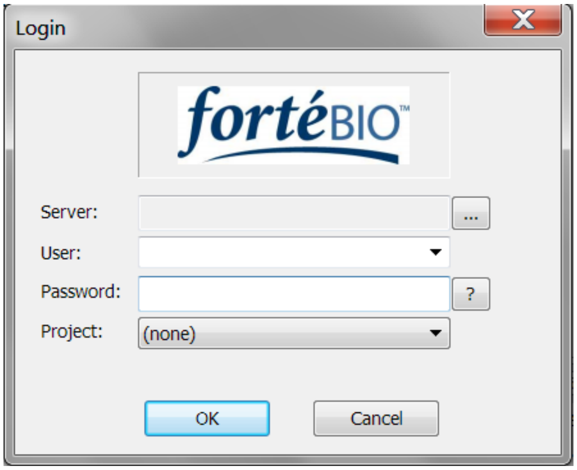

Figure 3-1: *Login Dialog Box*

2. Click **...** (browse) to select a **Server** location.

   The **Authentication Server** dialog box displays (Figure 3-2).

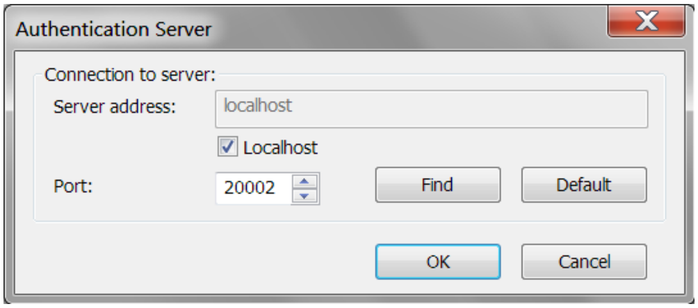

Figure 3-2: *Authentication Server Dialog Box*

3. Click **Default** to recall the default server settings of localhost and Port 2002.

   - ***Local host***—If the local computer is to be used as the GxP Server module host, click the **Localhost** check box. Change the **Port** number if necessary.
   - ***Remote host on same subnet***—If the GxP Server module is hosted on the same subnet, deselect the **Localhost** check box and click **Find**. A list of potential GxP Server module addresses will be listed. Choose the desired location from the list and click **OK**.

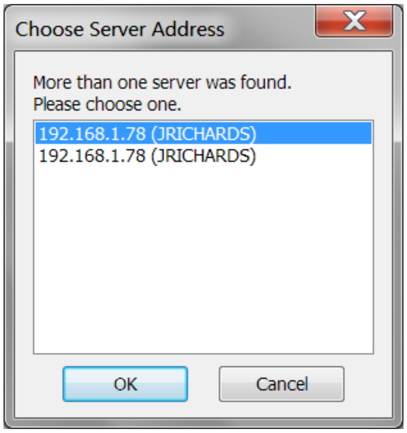

Figure 3-3: *Choose Server Address*

   - ***Remote host on another subnet***—If the GxP Server module is hosted on a different subnet, deselect the **Localhost** check box. Enter the IP address of the computer hosting the GxP Server module.

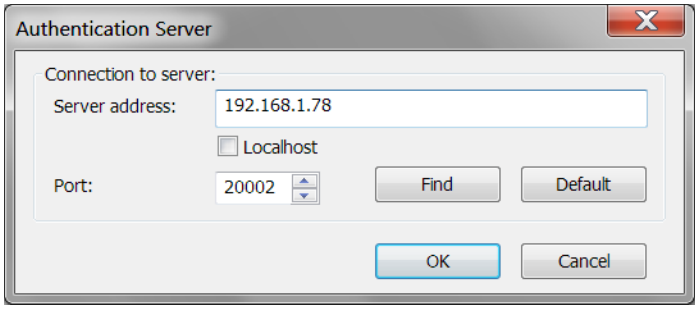

Figure 3-4: *Authentication Server Dialog Box*

4. When the GxP Server module host location has been selected or entered, click **OK** to save changes and exit the **Authentication Server** dialog box.

   The GxP Server module location is listed as the **Server** in the **Login** dialog box (Figure 3-5).

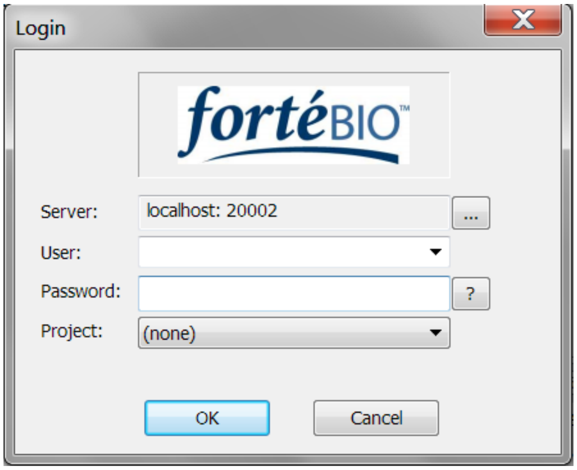

Figure 3-5: *Login Dialog Box—GxP Server Information*

STARTING A USER SESSION

NOTE: *Before starting your first user session, contact your administrator to determine the GxP Server module host location to use.*

To start a user session:

1. Launch the Data Acquisition or Data Analysis software by double-clicking the respective desktop icon.

   The **Login** dialog box displays:

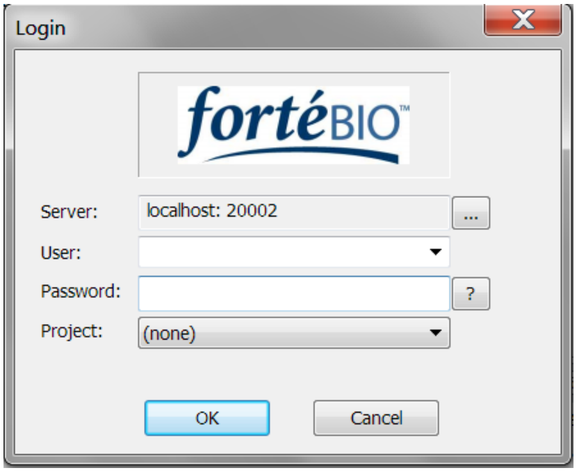

Figure 3-6: *Login Dialog Box*

2. Confirm that the **Server** location is correct. If not, see “Selecting a Server Location” on page 22.
   - If the *local* machine is to be used as the GxP server, ensure that **Localhost** is selected.
   - If a *remote* machine is to be used and it is located on the same subnet, deselect **Localhost** and click **Find** to display a list of potential GxP server addresses. Choose the desired GxP server from this list and click **OK**. Click **OK** in the Authentication Server dialog to finish. The new GxP server should be listed in the Login dialog box (next to Server).
   - If a remote machine is to be used and it is located on a different subnet, deselect **Localhost** and enter the IP address of the machine running the GxP server. Click **OK** to close the authentication server.
3. Select your login name from the **User** drop-down list (Figure 3-7). (For the first time logging in, select **Administrator**.)

***NOTE:** To start an administrator session, select **Administrator** in the **User** drop-down list.*

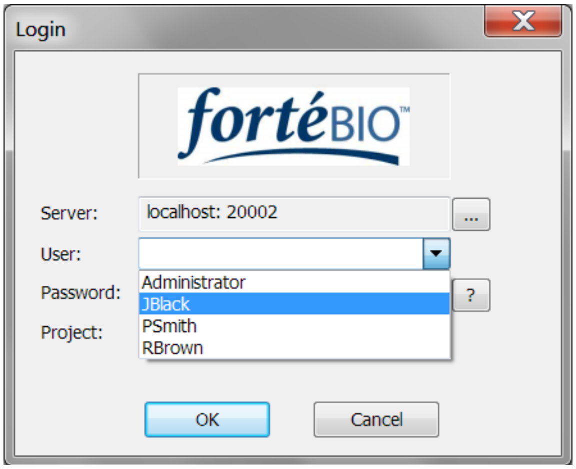

***Figure 3-7:** Selecting Login Username*

4. Enter your password in the **Password** field. Click **?** for a password reminder if needed (Figure 3-8). (For the first time logging in, leave the **Password** field blank.)

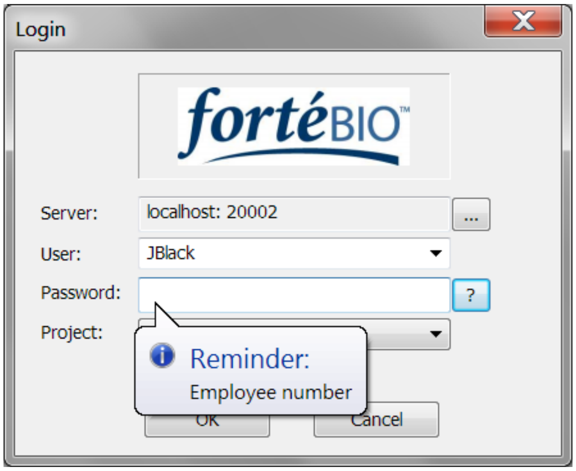

***Figure 3-8:** Password Reminder Option*

5. Optional. Select a project from the **Project** drop-down list (Figure 3-9). (For the first time logging in, leave as **(none)**.)

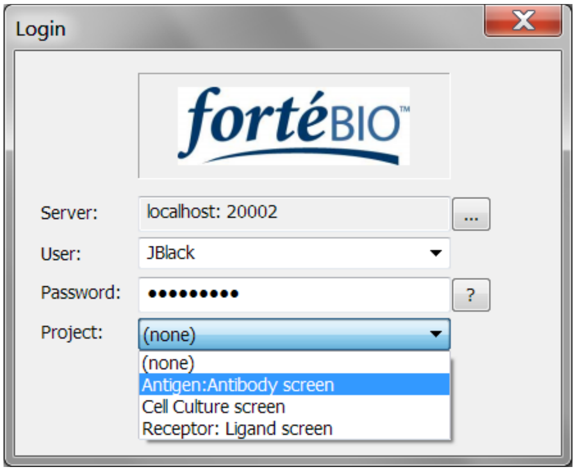

Figure 3-9: *Project Selection*

6. Click **OK.**

   The Data Acquisition or Data Analysis software launches and starts the user session. During the session, the user account and project selected at login are displayed in the Data Acquisition software status bar.

NOTES:

Software operation may be restricted based on your user privileges. For more information on user privileges, please contact your administrator.

*User sessions are automatically locked after a period of inactivity which is set by the administrator. The **Login** box will display and a message indicating the session has been locked will be shown. You can choose to log back into the session or log off at this time. User sessions will not be locked during experimental data acquisition.*

To create and edit new users, groups, and projects, see "User Account Administration" on page 192, "Group Administration" on page 197, and "Project Administration" on page 200.

COMPLIANCE FEATURES

You can access the 21 CFR Part 11 compliant features provided in the 21 CFR Part 11 versions of the Data Acquisition and Data Analysis software by selecting the **Security** menu from the main menu (Figure 3-10).

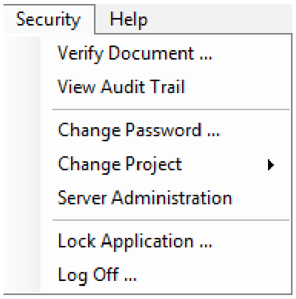

Figure 3-10: *Security Menu*

NOTES:
The ***Server Administration*** *option in the* ***Security*** *menu can be accessed only if you have administrator or review privileges.*

Security *menu options in the Data Acquisition and Data Analysis software applications are identical.*

Experiment and Method File Compliance

When using the 21 CFR Part 11 version of the Octet System Data Acquisition and Data Analysis software, only 21 CFR Part 11 compliant experiments and method files generated using the 21 CFR Part 11 version of the software can be opened. Files generated using the non-compliant version of the software cannot be opened, and a message indicating this will be presented.

Verifying Digital Signatures

Electronic signatures are added to method (.fmf) and data (.frd) files which ensure they were generated with 21 CFR Part 11-compliant software. In Octet Data Analysis software version 8.2 and higher, electronic signatures are also added to data analysis settings files (Settings_DataAnalysis.fsd) and non-CFR standards data (.fsc) files.

The electronic signature of files can be verified. To do this:

1. Click **Security** > **Verify Document**.

   The **Verify Digital Signature** dialog box displays (Figure 3-11).

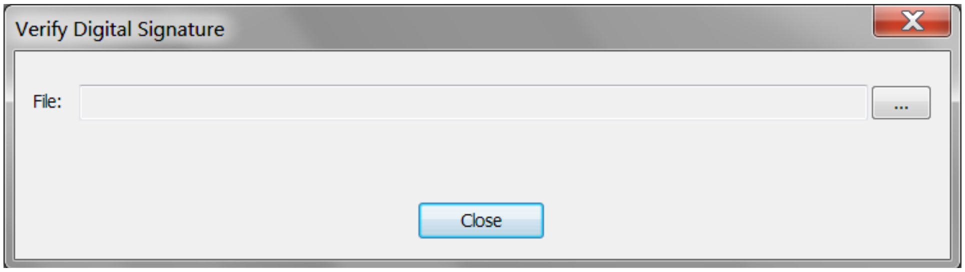

Figure 3-11: Verify Digital Signature

2. Click **...** to browse for the desired file (.fmf, .frd, .ini, .fsd or .fsc).

NOTE: *When verifying digital signatures, both method (.fmf) and data (.frd, .fsc) files can be selected in the Data Acquisition and Data Analysis software.*

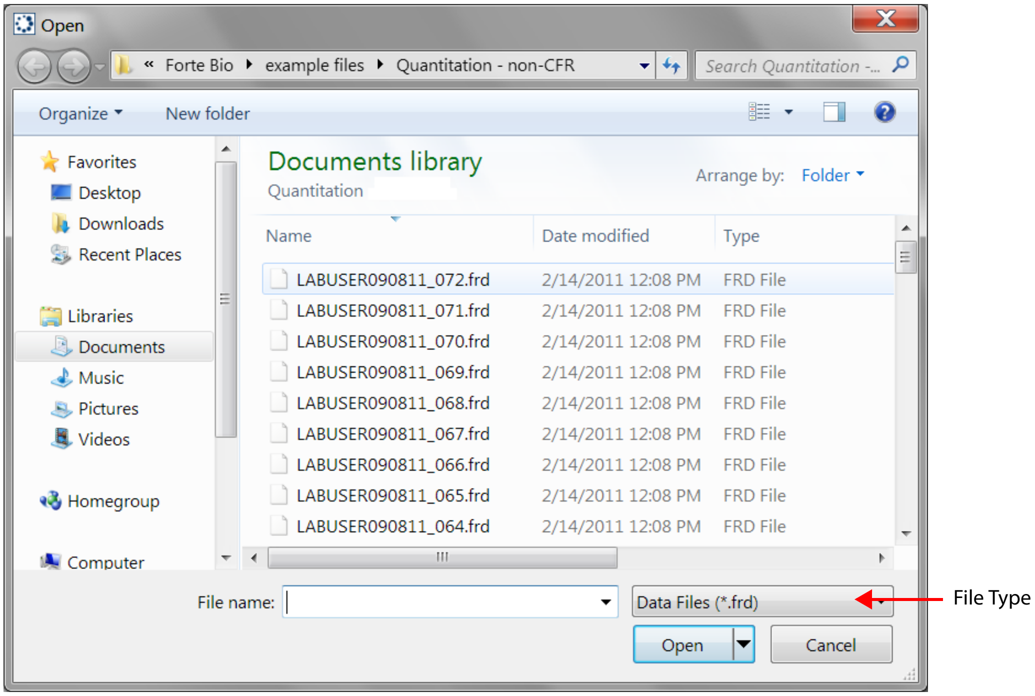

Figure 3-12: File Selection

To change the file type available for selection, click the file type box and select a different format (Figure 3-13).

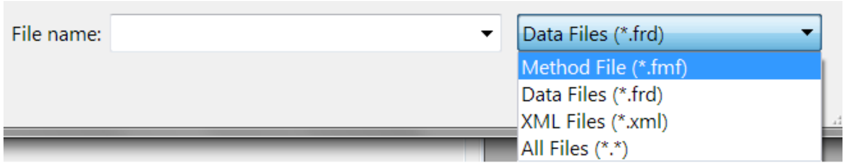

Figure 3-13: *Changing File Type*

3. Select the desired file and click **OK**.

   A message displays in the **Verify Digital Signature** dialog box, indicating file compliance status (**Compliant** or **Non-Compliant**) (Figure 3-14).

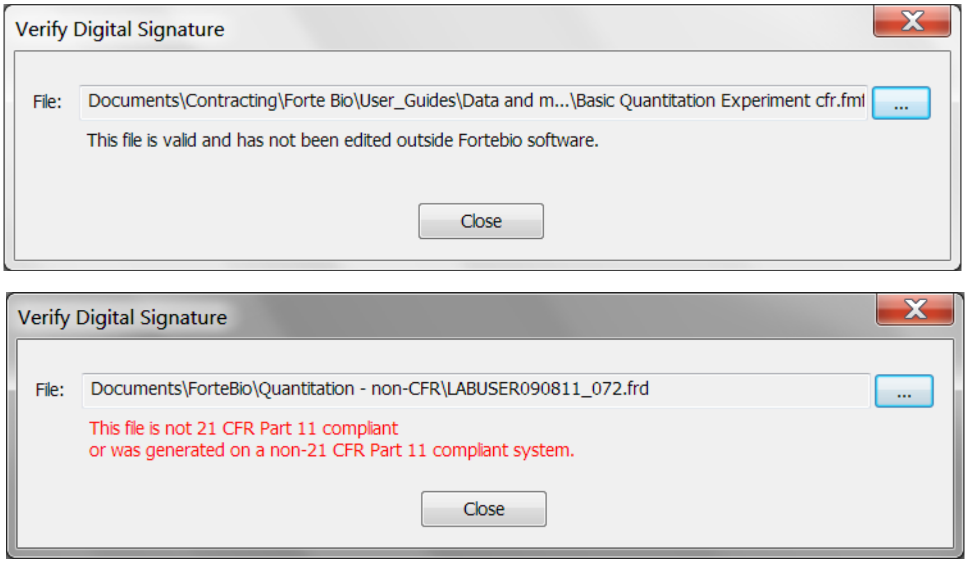

Figure 3-14: *File Compliant (top), File Not Compliant (bottom)*

Viewing the Data Settings Change History

In Octet Data Analysis 21 CFR Part 11 software versions 8.2 and higher, a history of changes made to analysis settings are also archived within the experiment folder. To view the settings change history for the experiment currently open, select **Security** from the main menu and click **View Data Settings Change History**.

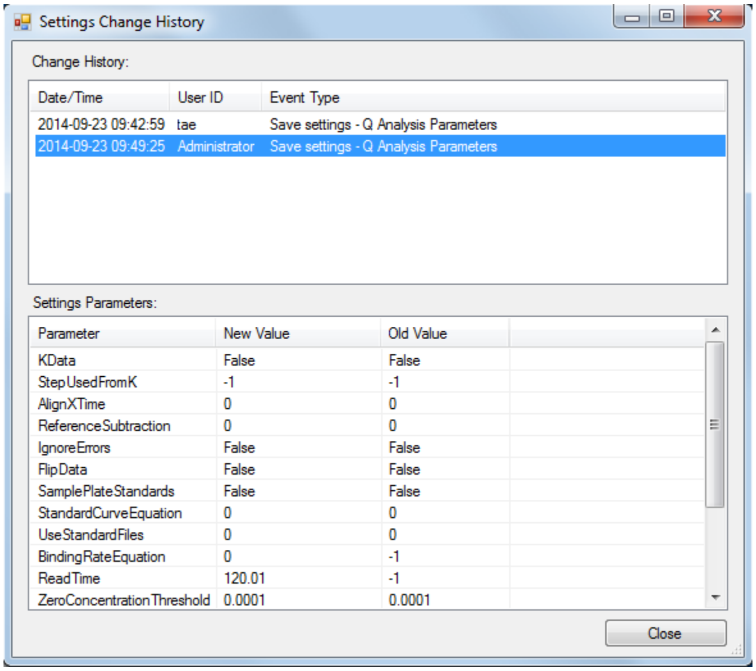

Figure 3-15: Data Settings Change History

The change history includes the old and new values for each setting whenever they are:

- Changed from the default setting
- Changed from a previous setting

Viewing the Audit Trail

The Audit Trail displays a historical log of user, system and software events recorded during user sessions. To view and display the Audit Trail, click **Security** > **View Audit Trail** (Figure 3-16).

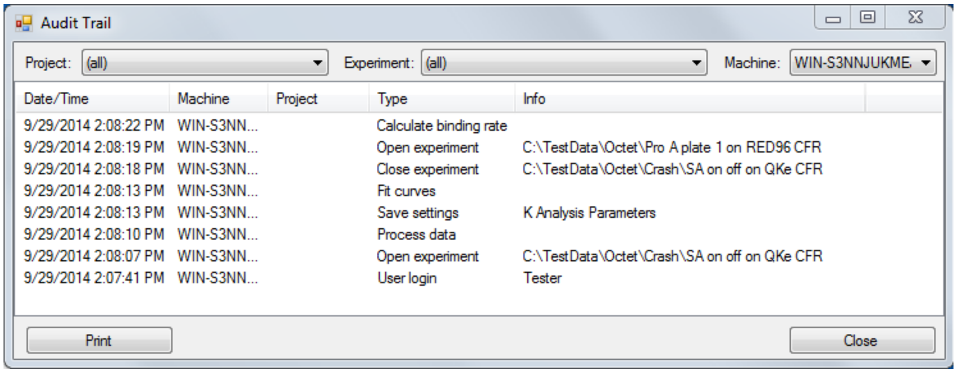

Figure 3-16: Audit Trail

***NOTE**: Events displayed in the Audit Trail are those associated with the user account that is currently logged in and active only.*

Sorting Events in the Audit Trail

Events in the Audit Trail can be sorted by clicking any of the column headers (Figure 3-17).

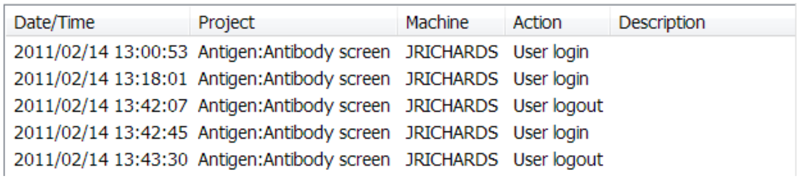

Date/Time	Project	Machine	Action	Description
2011/02/14 13:00:53	Antigen:Antibody screen	JRICHARDS	User login	
2011/02/14 13:18:01	Antigen:Antibody screen	JRICHARDS	User login	
2011/02/14 13:42:07	Antigen:Antibody screen	JRICHARDS	User logout	
2011/02/14 13:42:45	Antigen:Antibody screen	JRICHARDS	User login	
2011/02/14 13:43:30	Antigen:Antibody screen	JRICHARDS	User logout	

***Figure 3-17:** Events Listed in the Audit Trail*

By default, the events initially displayed in the Audit Trail are those associated with the project selected at login and the machine (computer) currently being used. To view events for a specific project, experiment or computer, click the **Project**, **Experiment** or **Machine** drop-down list and select an entry (Figure 3-18).

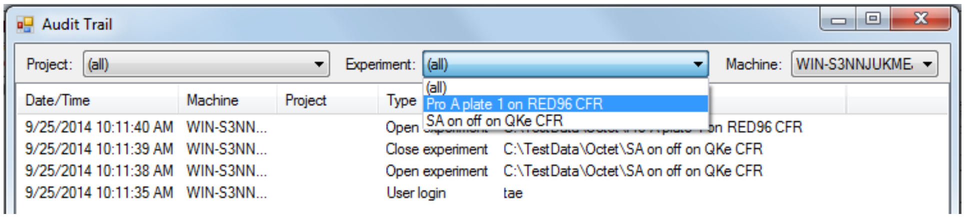

***Figure 3-18:** Selecting an Experiment in the Audit Trail*

***NOTE:** Selections can be made in either one or both of the **Project** or **Machine** drop down lists.*

The list only displays events for the selected entries (Figure 3-19).

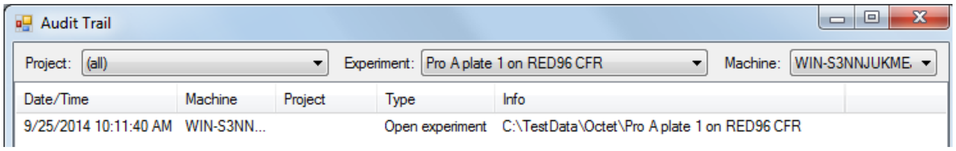

***Figure 3-19:** Experiment-Based Audit Trail Events*

In addition to the specific project and machine selections, the following list options are also available:

- **(any)**—Displays all project and/or machine events for the user account.
- **(none)**—Displays all project or machine events not associated with a specific project (**Project** list only).

Printing the Audit Trail

To print the Audit Trail, from the main menu click the **Print** button in the window. The Audit Trail will print in tabular format based on the current selection of project, experiment and machine events:

Octet Analysis(8.2.0.1) Audit Trail [Machine:CAMP-KANGT, Project:Test Project, Experiment:(all)]				
Date/Time	Machine	Project	Type	Info
8/27/2014 9:31:15 AM	CAMP-KANGT	Test Project	Save settings	Q Std Curve View Options
8/27/2014 9:31:10 AM	CAMP-KANGT	Test Project	Calculate binding rate	
8/27/2014 9:30:59 AM	CAMP-KANGT	Test Project	Save standard data	C:\TestData\Octet QKe_Quantitation_CFR_FB-40334\StandardCurve...
8/27/2014 9:30:49 AM	CAMP-KANGT	Test Project	Calculate binding rate	
8/27/2014 9:30:44 AM	CAMP-KANGT	Test Project	Open experiment	C:\TestData\Octet QKe_Quantitation_CFR_FB-40334
8/27/2014 9:30:26 AM	CAMP-KANGT	Test Project	User login	tae

Figure 3-20: *Printed Audit Trail*

Changing Projects During a User Session

During an active session, you can switch to another project in the Data Acquisition or Data Analysis software without having to log out.

To change projects during a user session:

1. Click **Security** > **Change Projects**.

   A list of projects assigned to your user account displays with the active project high-lighted (Figure 3-21).

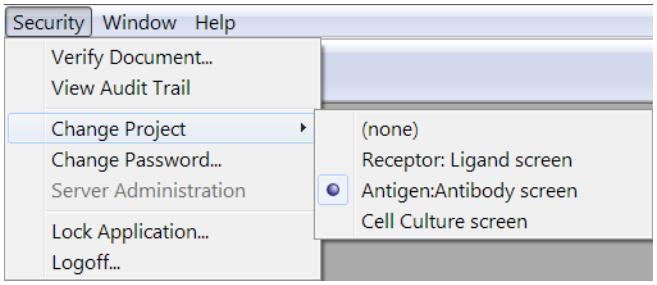

Figure 3-21: *Changing Projects*

2. Select the desired project from the list.

   The selected project becomes the active project for the user session.

Changing the User Password

To change the user password:

1. Initiate a new user session with your existing password.
2. When the software launches, click **Security** > **Change Password**.

   The **Change Password** dialog box displays (Figure 3-22).

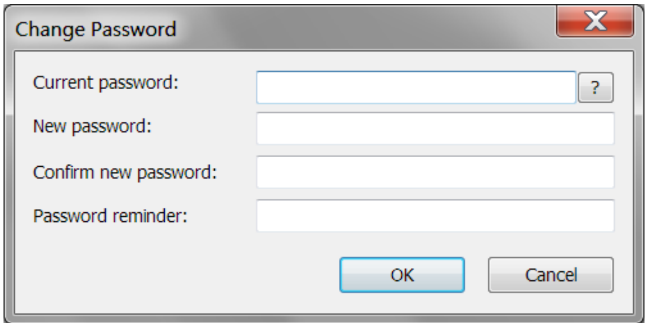

Figure 3-22: *Change Password*

3. Enter the **Current password** for your user account. Click **?** for a password reminder.
4. Enter the **New Password**, confirm the new password, and optionally enter a **Password reminder**.
5. Click **OK** to save the changes and exit.

Locking the Application

The Data Acquisition or Data Analysis software can be locked during a user session to prevent another user from interrupting a session or experiment. When the application is locked, any experiments started will continue to run.

To lock the Octet System Data Acquisition software application, click **Security** > **Lock Application**.

The Octet System Data Acquisition software is placed in locked mode immediately and the **Application Locked** window displays (Figure 3-23).

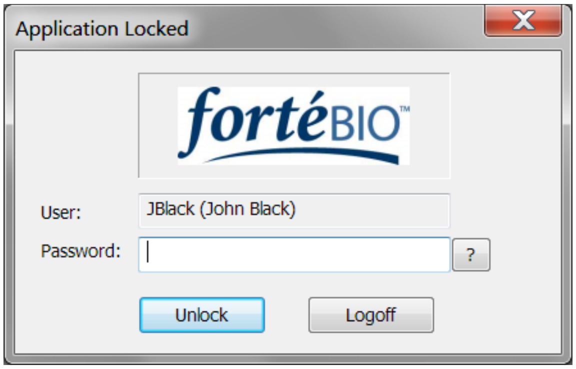

Figure 3-23: Application Locked

The application will remain locked until it is unlocked or the active user logs off.

- ***Unlock***—To resume the user session, enter your password and click **Unlock**.
- ***Log off***—To discontinue the user session, click **Logoff**.

Ending a User Session

To end a user session:

1. Click **Security** > **Log Off**.
2. Click **OK** in the dialog box displayed.

NOTE: *You will be logged out of your user session automatically if there is no user interaction with the software after a specified period of time. This time is set by the administrator, and allows the ForteBio GXP Server Module to proved added security.*

CHAPTER 4:

Quantitative Analysis

Working with Experiments 38
Analyzing Data 52
Saving Standards Data 68
Saving Analysis Settings 68
Processing Batch Quantitation Analysis 68
Exporting Data 72
Masking Data 75
Multi-Step Advanced Quantitation Experiments 75

WORKING WITH EXPERIMENTS

A quantitation experiment enables you to determine sample concentration using a reference set of standards. After an experiment is run, start a data analysis session (double-click the icon on the desktop); see “Analyzing Binding Data” on page 52.

Loading an Experiment for Analysis

A data analysis session can be used to:

- Load and analyze an experiment.
- Re-analyze an experiment.

NOTE: *More than one experiment can be opened during a session. If multiple quantitation experiments are open, the analysis includes the data from all of the biosensors that are check marked in the* ***Results*** *tab.*

NOTE: *When using the 21 CFR Part 11 version of the Octet System Data Acquisition software, only 21 CFR Part 11-compliant experiments and re-analyze generated using the 21 CFR Part 11 version of the software can be opened. Files generated using the non-compliant version of the software or with a non-compliant system cannot be opened, and a message indicating this will be presented.*

To load an experiment:

1. On the desktop, click the icon, or click the Windows **Start** button and select **All Programs** > **ForteBio** > **Data Analysis 7.0**.

   The Data Selection tab displays (Figure 4-1).

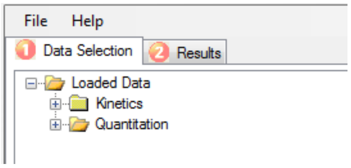

Figure 4-1: *Data Selection Tab*

2. Load an experiment: right-click the experiment folder in the workstation directory tree and select **Load Folder**, or on the menu bar, click **File** > **Load a Folder**.
3. In the Loading Files dialog box, enter the folder name or click the **Browse** button ..., select the desired folder, and click **Load**.

   The experiment is added to the Loaded Data directory tree (see Figure 4-2 example).

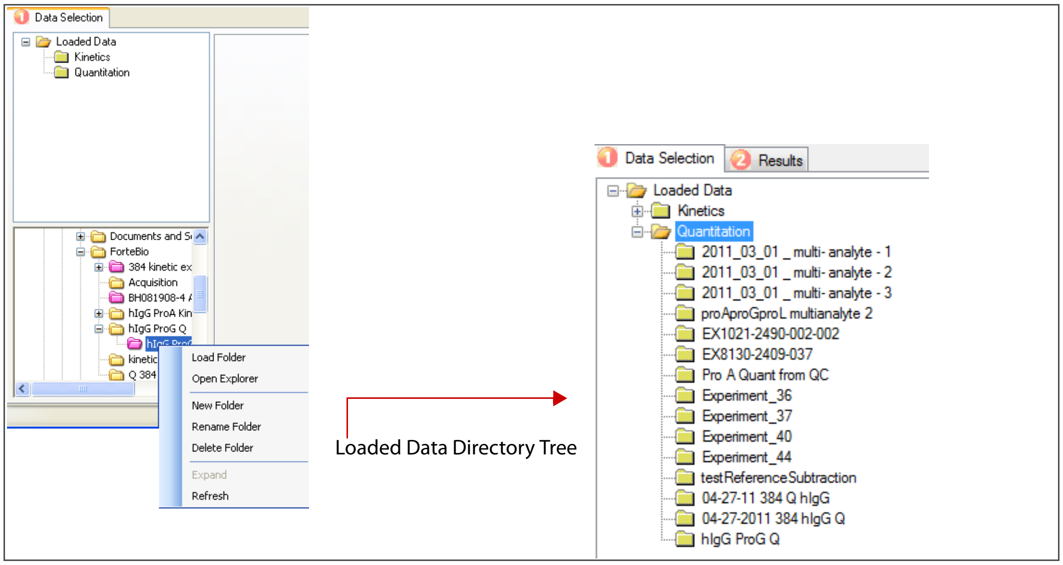

Figure 4-2: Data Selection Window—Loading an Experiment

4. In the Loaded Data directory, click the experiment name to open.

   The binding curves, sample plate, and sample plate table appear (Figure 4-3).

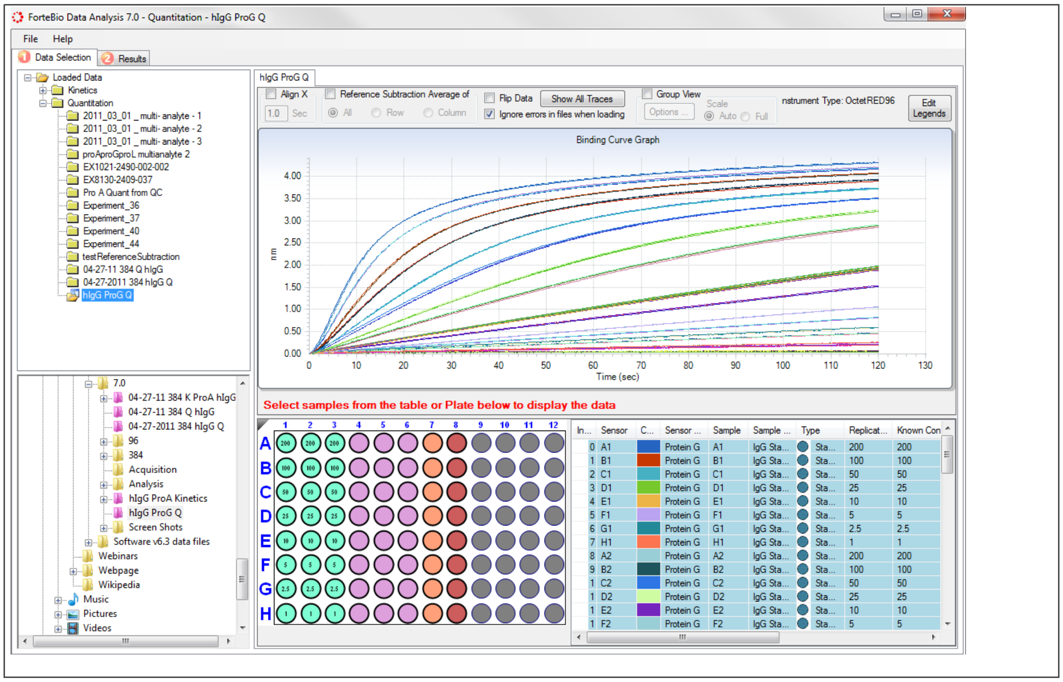

Figure 4-3: Data Selection Window—Opening an Experiment

5. Repeat steps 2–3 to load and open another experiment.

***NOTE:** When multiple experiments are loaded, select an experiment by clicking its tab above the binding chart (in the Data Selection window), or click the experiment name in the Loaded Data directory tree.*

Editing Experiments

The Octet System Data Acquisition software enables you to change sample designations, standard concentrations, or exclude samples from analysis. For example, you can exclude a standard that does not meet the sample r^2 or residual threshold, then re-analyze the data. You can also modify some processing parameters.

***NOTE:** If you are using the 21 CFR Part 11 version of the Octet System Data Acquisition software, editing options are disabled.*

Changing Sample Designations

To change sample designations:

1. Click the **Data Selection** tab, then perform one of the following tasks:
   - In the *sample plate map*, select the well(s), right-click, and select one of the following options (see left image in Figure 4-4):
     - Change to Standard
     - Change to Unknown
     - Change to Control
     - Change to Reference
     - Edit Sample Information
   - In the *results table*, right-click a table cell and make a selection from the drop-down menu (see right image in Figure 4-4).

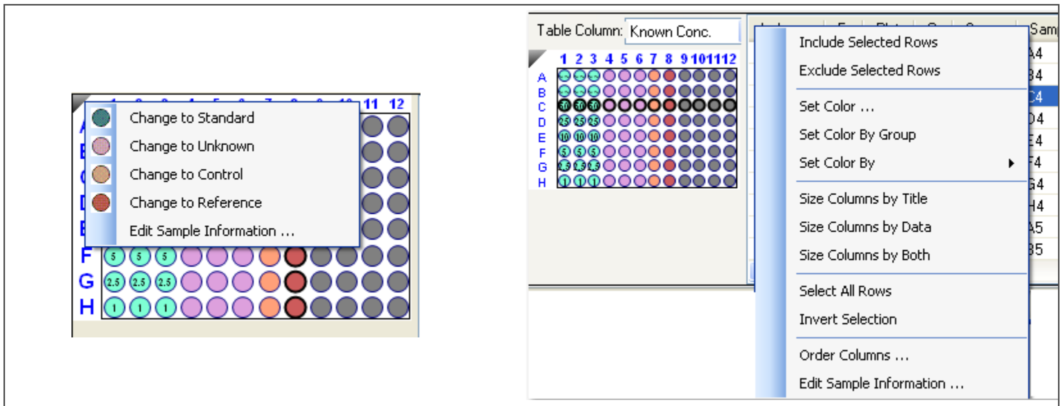

Selecting wells in the sample plate map

Selecting a cell in the Well Type column of the results table

***Figure 4-4:** Changing Sample Designations*

Excluding/Including Samples from Analysis

To toggle sample analysis in the Results window, perform one of the following tasks:

- In the *sample plate map,* select the well(s), right-click and select **Exclude Wells**. If the selection is already excluded from analysis, select **Include Wells** to return (include) the samples to the analysis.
- In the *results table*, to exclude wells, deselect the check box in the first column, or right-click the selected rows and select **Exclude Selected Wells**. If the selection is already excluded from analysis, click the check box for the desired rows, or right-click the desired rows, and select **Include Selected Wells**.

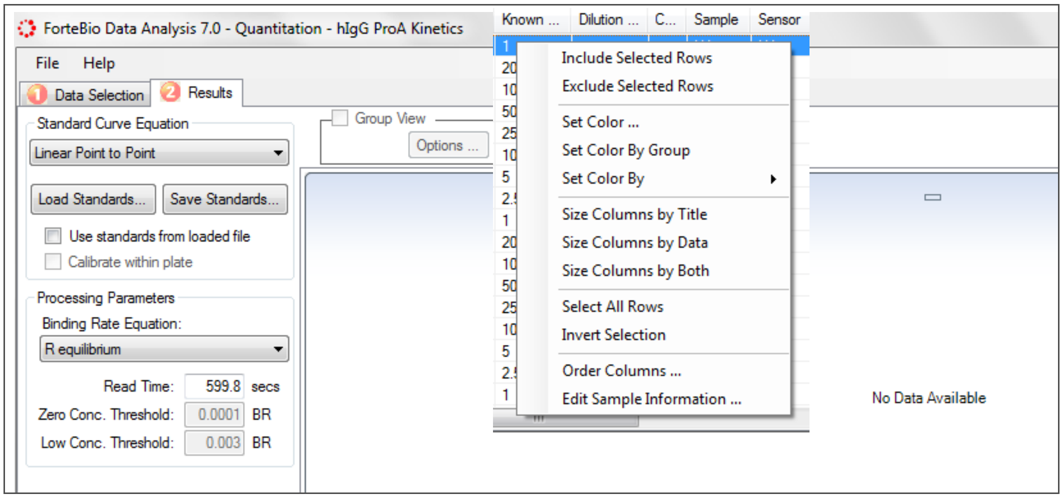

Figure 4-5: Excluding Samples from Analysis

Editing Table Information

The following information in the table can be edited directly. Just click in the cell, then type to make changes (Figure 4-6):

- Sample ID
- Replicate Group
- Known Conc
- Dilution Factor

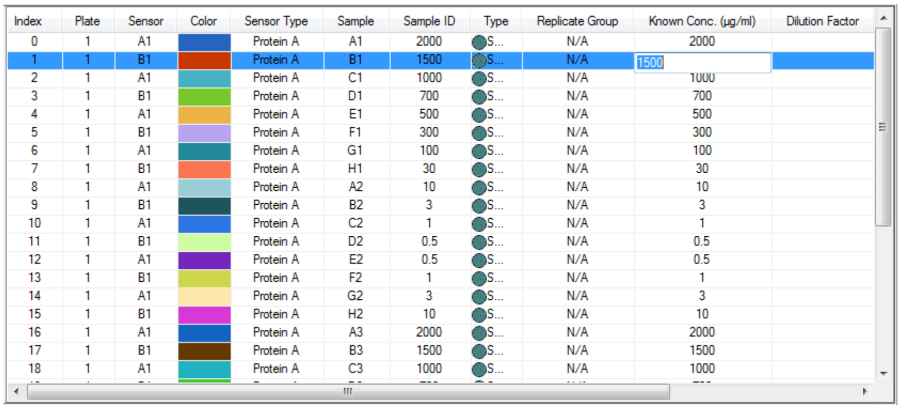

Index	Plate	Sensor	Color	Sensor Type	Sample	Sample ID	Type	Replicate Group	Known Conc. (µg/ml)	Dilution Factor
0	1	A1		Protein A	A1	2000	S...	N/A	2000	
1	1	B1		Protein A	B1	1500	S...	N/A	1500	
2	1	A1		Protein A	C1	1000	S...	N/A	1000	
3	1	B1		Protein A	D1	700	S...	N/A	700	
4	1	A1		Protein A	E1	500	S...	N/A	500	
5	1	B1		Protein A	F1	300	S...	N/A	300	
6	1	A1		Protein A	G1	100	S...	N/A	100	
7	1	B1		Protein A	H1	30	S...	N/A	30	
8	1	A1		Protein A	A2	10	S...	N/A	10	
9	1	B1		Protein A	B2	3	S...	N/A	3	
10	1	A1		Protein A	C2	1	S...	N/A	1	
11	1	B1		Protein A	D2	0.5	S...	N/A	0.5	
12	1	A1		Protein A	E2	0.5	S...	N/A	0.5	
13	1	B1		Protein A	F2	1	S...	N/A	1	
14	1	A1		Protein A	G2	3	S...	N/A	3	
15	1	B1		Protein A	H2	10	S...	N/A	10	
16	1	A1		Protein A	A3	2000	S...	N/A	2000	
17	1	B1		Protein A	B3	1500	S...	N/A	1500	
18	1	A1		Protein A	C3	1000	S...	N/A	1000	

Figure 4-6: *Editing Cells in Data Table*

Editing Standard Concentration or Well Information

To edit standard concentration or well information:

1. Click the **Data Selection** tab.
2. In the results table, click a **Conc.** or **Well Info** cell, and enter the desired information. (To access a shortcut menu of editing commands, right-click the cell.)

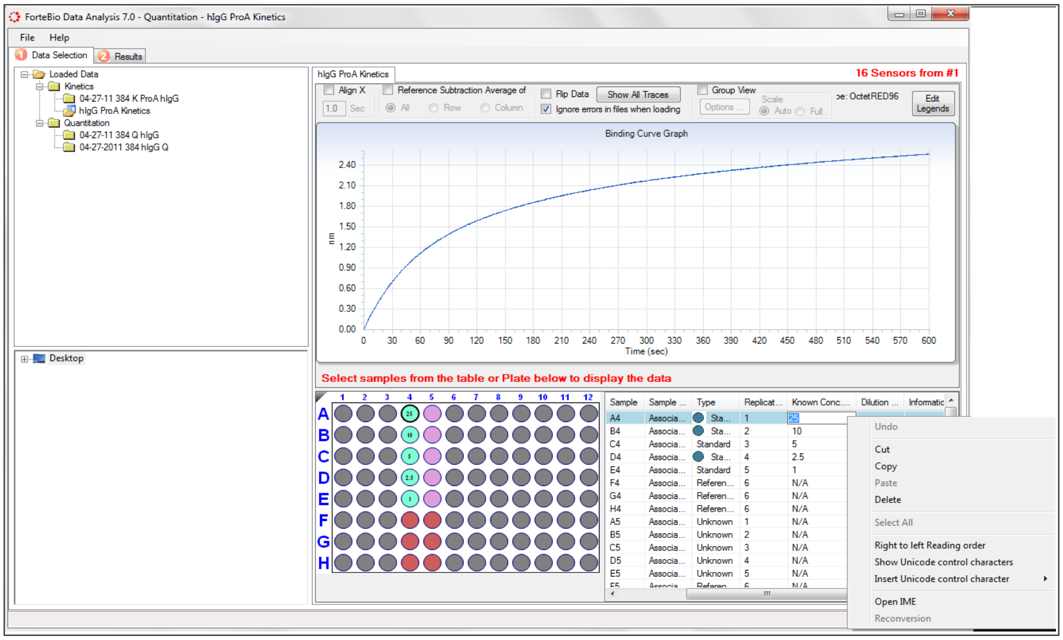

Figure 4-7: Data Selection Window—Editing Sample Information

Editing Processing Parameters

To edit processing parameters:

1. Modify the following parameters as appropriate:
   - **Read time**—The amount of data that is analyzed.
   - **Zero concentration threshold**—Binding rates that are less than the zero concentration threshold are considered zero.
   - **Low concentration threshold**—Clicking **Calculate binding rate!** causes the initial rate to be calculated using both a linear and exponential equation. The low concentration threshold determines which value is reported in the results table. Changing this threshold can improve the precision of low concentration samples.
     - If the result from a linear fit is below the low concentration threshold, then the value from the linear fit is reported in the analysis table.
     - If the result from a linear fit is greater than the low concentration threshold, then the value from the exponential fit is reported in the analysis table.
2. In the Results window, select the cell to edit and enter a new value. Or, right-click the cell to access a shortcut menu of edit commands.

   The modified parameters are saved in the Settings_DataAnalysis.ini or Settings_DataAnalysis.fsd file when you click **Calculate Binding Data!**.

NOTE: *In Octet Data Analysis 21 CFR Part 11 software versions 8.2 and higher, the Settings_DataAnalysis.ini file name extension is Settings_DataAnalysis.fsd. Electronic signatures are also added to data analysis settings (.fsd) files.*

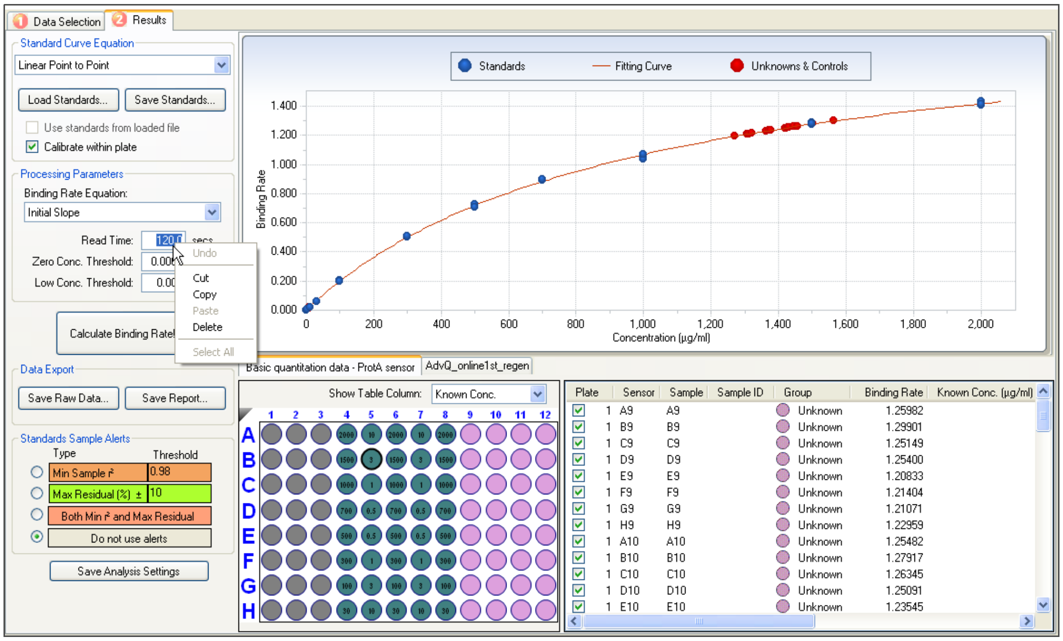

Figure 4-8: Editing Processing Parameters in the Results Window

Defining Replicate Groups

The Replicate Group feature enables data to be organized into custom groups during analysis (see Figure 4-9). Replicates can be defined during acquisition (or analysis) as a group. For each group, the average binding rate, average concentration, and corresponding standard deviation, CV% are calculated.

To define replicate groups:

1. Enter replicate grouping information in the Results table: right-click and select **Edit Sample Information** (Figure 4-9).

Index	Include	Color	Sensor Location	Sensor Type	Sensor Info	Replicate Group	Baseline Loc.
20	x		C2	SA (Streptavidin)		3	C3
21	x		C2	SA (Streptavidin)		3	C3
22	x		D2	SA (Streptavidin)		4	D3
23	x		D2	SA (Streptavidin)		4	D3
24	x		E2	SA (Streptavidin)		5	E3
25	x		E2	SA (Streptavidin)		5	E3
26	x		F2	SA (Streptavidin)		6	F3
27	x		F2	SA (Streptavidin)		6	F3
28	x		G2	SA (Streptavidin)		6	G3
29	x		G2	SA (Streptavidin)		6	G3
30	x		H2	SA (Streptavidin)		6	H3
31	x		H2	SA (Streptavidin)		6	H3
32	x		A3	SA (Streptavidin)		1	A3

Figure 4-9: *Edit Sample Information*

NOTE: *Replicate Group information can also be entered in the Octet System Data Acquisition software.*

Assigning Replicate Groups in the Sample Plate Map

To assign Replicate Groups in the Sample Plate Map:

1. Select the samples to group, right-click and select **Set Well Data**.
2. In the Set Well Data dialog box (see Figure 4-10), enter a name in the Replicate Group box and click **OK**.

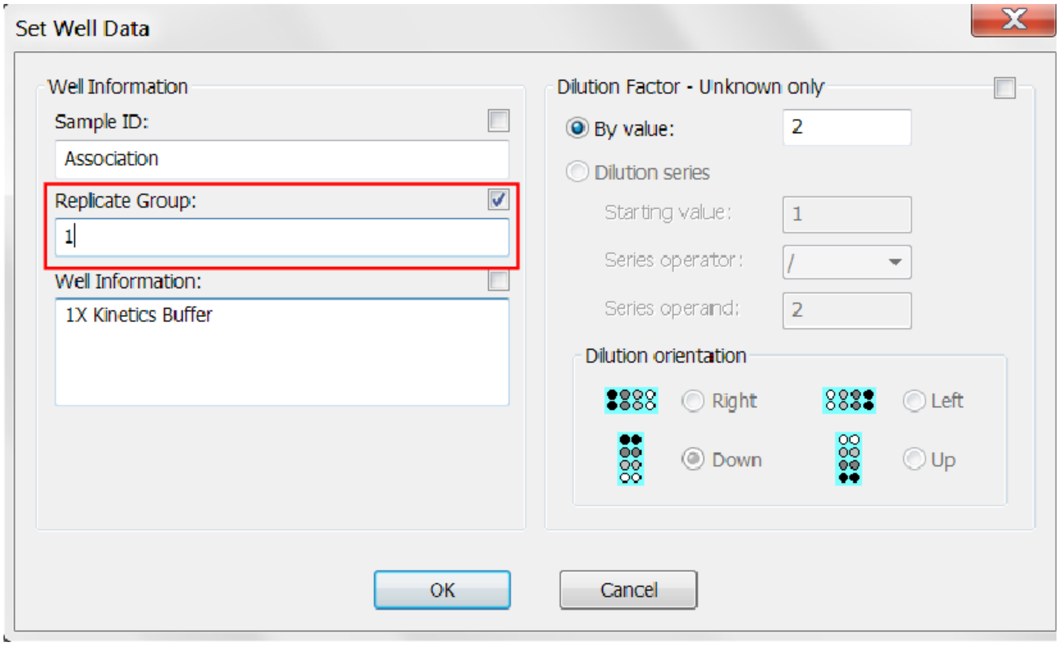

Figure 4-10: *Set Well Data Dialog Box—Add Replicate Group from the Sample Plate Map*

3. Repeat the previous steps to assign new samples to the existing Replicate Group, or to designate another set of samples to a new Replicate Group. Multiple groups can be used in an experiment.

IMPORTANT: *The Octet System Data Analysis software will only recognize and group samples that use the same Replicate Group names—spacing and capitalization must be identical. For example, samples assigned to Group 2 and group2 are treated as two groups.*

NOTE: *When performing a Multiple Analyte experiment in Data Acquisition, if the same Replicate Group name is used with different biosensor types, they will be treated as separate groups. Statistics for these groups will be calculated separately for each biosensor type.*

Wells in the Sample Plate Map will show color-coded outlines as a visual indication of which wells are in the same group (see Figure 4-11).

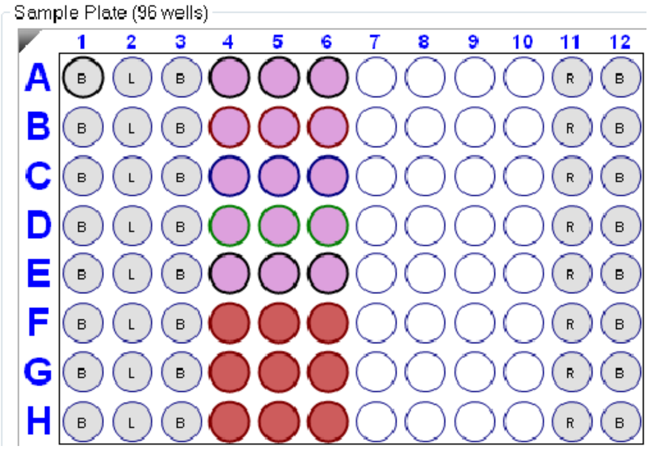

Figure 4-11: *Replicate Groups Displayed in Sample Plate Map*

The Sample Plate Table updates with the Replicate Group names entered (see Figure 4-12).

Sample Plate Table

Concentration units: µg/ml

Molar concentration units: nM

Well	Sample ID	Replicate Group	Type	Conc (µg/ml)	MW (kD)	Molar Conc (nM)	Information
A4	Association	1	Sample	10	150	66.67	1X Kinetics Buffer
B4	Association	2	Sample	5	150	33.33	1X Kinetics Buffer
C4	Association	3	Sample	2.5	150	16.67	1X Kinetics Buffer
D4	Association	4	Sample	1.25	150	8.333	1X Kinetics Buffer
E4	Association	5	Sample	0.625	150	4.167	1X Kinetics Buffer
F4	Association	6	Reference				1X Kinetics Buffer
G4	Association	6	Reference				1X Kinetics Buffer
H4	Association	6	Reference				1X Kinetics Buffer
A5	Association	1	Sample	10	150	66.67	1X Kinetics Buffer
B5	Association	2	Sample	5	150	33.33	1X Kinetics Buffer
C5	Association	3	Sample	2.5	150	16.67	1X Kinetics Buffer
D5	Association	4	Sample	1.25	150	8.333	1X Kinetics Buffer
E5	Association	5	Sample	0.625	150	4.167	1X Kinetics Buffer
F5	Association	6	Reference				1X Kinetics Buffer
G5	Association	6	Reference				1X Kinetics Buffer
H5	Association	6	Reference				1X Kinetics Buffer

***Figure 4-12:** Replicate Groups Displayed in Sample Plate Table*

Assigning Replicate Groups in the Sample Plate Table

To assign Replicate Groups in the Sample Plate Table:

1. Double-click the desired cell in the Replicate Group table column.
2. Enter a group name (see Figure 4-13).

Sample Plate Table

Concentration units: µg/ml

Molar concentration units: nM

Well	Sample ID	Replicate Group	Type	Conc (µg/ml)	MW (kD)	Molar Conc (nM)	Information
A4	Association	1	Sample	10	150	66.67	1X Kinetics Buffer
B4	Association	2	Sample	5	150	33.33	1X Kinetics Buffer
C4	Association	3	Sample	2.5	150	16.67	1X Kinetics Buffer
D4	Association	4	Sample	1.25	150	8.333	1X Kinetics Buffer
E4	Association	5	Sample	0.625	150	4.167	1X Kinetics Buffer
F4	Association	6	Reference				1X Kinetics Buffer
G4	Association	6	Reference				1X Kinetics Buffer
H4	Association	6	Reference				1X Kinetics Buffer

***Figure 4-13:** Add Replicate Group from the Sample Plate Table*

Edit commands (**Cut**, **Copy**, **Paste**, **Delete**) and shortcut keys (Cut [**Ctrl+x**], Copy [**Ctrl+c**], Paste [**Ctrl+v**], Undo [**Ctrl+z**]) are available in the Sample Plate Table. To view edit commands, double-click the cell. This highlights the value and allows it to be edited. Next, right-click to view the edit menu.

***NOTE:** The right-click menu is context-dependent. Right-clicking on a cell where the value is not highlighted and in edit mode opens the Sample Plate Map menu used to designate sample types.*

3. Repeat the previous steps to assign new samples to the existing Replicate Group, or to designate another set of samples to a new Replicate Group. Multiple groups can be used in an experiment.

IMPORTANT: *The Octet System Data Analysis software only recognizes and groups samples that use the same Replicate Group names where spacing and capitalization must be identical. For example, samples assigned to Group 2 and group2 are treated as two groups.*

Viewing Binding Curves

To view binding curves, in the sample plate, select a well(s), or in the sample plate table, select a row(s).

To select non-adjacent rows or wells, press and hold the **Ctrl** key while clicking the wells or rows.

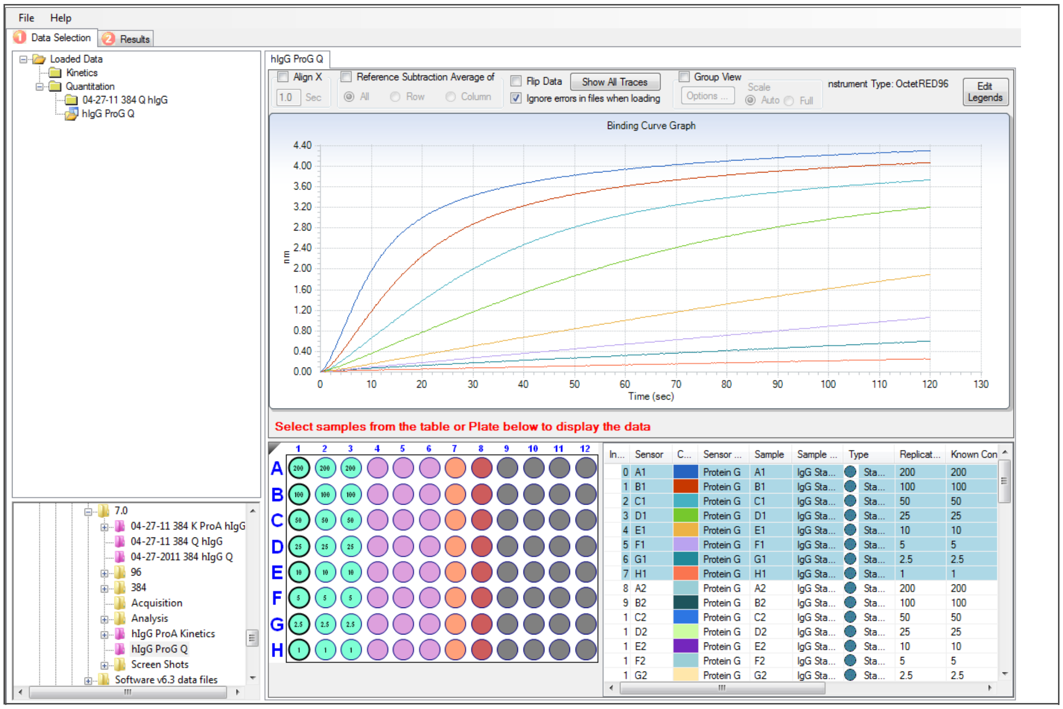

Figure 4-14: *Selecting Sample Wells or Rows to Display in the Binding Curve Graph*

Viewing Options

Table 4-1: *Viewing Options in the Data Selection Window*

Option	Description
**Align X**	Select this option if there is an artifact at the beginning of the binding step to remove. Enter a time (seconds) at which to start the alignment.
**Reference Subtraction Average of**	If the experiment includes reference biosensors, select the **Reference Subtraction** option and select one of the following:<br>• **All**—Computes the average binding curve from the reference wells and subtracts this average from each sample curve.<br>• **Row**—If a row includes both samples and references, the Octet System Data Acquisition software computes the average reference curve for the row and subtracts this curve from the samples in the same row.<br>• **Column**—If a column includes both samples and references, the Octet System Data Acquisition software computes the average reference curve for the column and subtracts this curve from the samples in the same column.
**Ignore errors in files when loading**	If this option is selected, the Octet System Data Acquisition software ignores errors in data files. All data files, regardless of errors or runtime issues, will be loaded for analysis.
**Show All Traces**	Displays all binding curves.
**Flip Data**	The Flip Data function inverts signals from positive to negative or from negative to positive. This is used most often when the observed nm shift is negative due to the presence of large analytes, such as phage, cells, and lipoparticles on the biosensor surface. For examples of flipping data, see Figure 4-15 and Figure 4-16.
**Grouped View**	Displays graphs in custom groupings. Choose this option to display graphs organized into groups according to sample attribute or results category. This is a highly useful feature when working with large data sets.<br>• **Options**—Click to show the Grouped View Options dialog box.<br>• **Refresh**—Updates the graph display.

Table 4-1: Viewing Options in the Data Selection Window (Continued)

Option	Description
**Edit Legends**	Select the sample information displayed in the legend. Options include **Sensor**, **Sample**, **Sample ID**, **Group**, and **Concentration**.

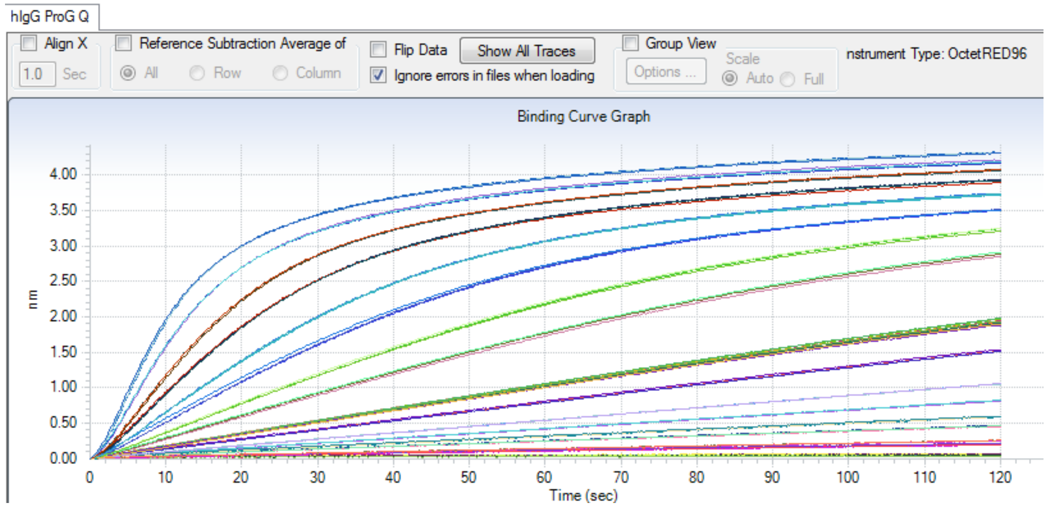

Figure 4-15: Flip Data—Example of Flipping Data to Example in Figure 4-16

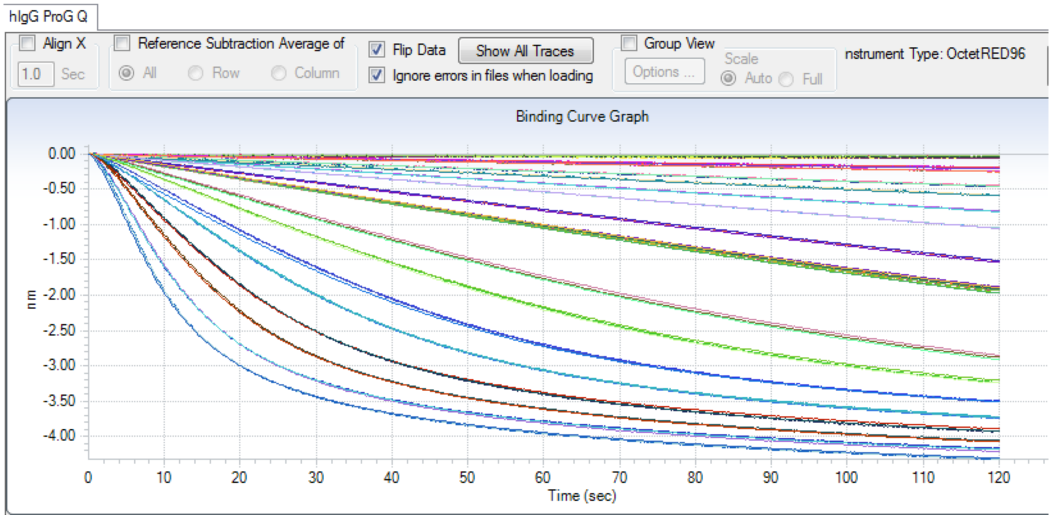

Figure 4-16: New Flip Data from Figure 4-15

Opening Binding Curve in Separate Window

To open the binding curve chart in a separate window, double-click the graph.

Customizing Appearance of Graph or Binding Curve

To customize the appearance of the graph or a binding curve; see Figure 4-17. Right-click the graph or a curve for a shortcut menu of display options.

- Hover over a binding curve to display a tooltip of the X-Y coordinates.
- Right-click the graph to view a shortcut menu of display options.
- Right-click a curve to view a shortcut menu of display options.
- Right-click the graph and select **Toolbar**. Toolbar buttons enable you to save, copy, or print the graph.

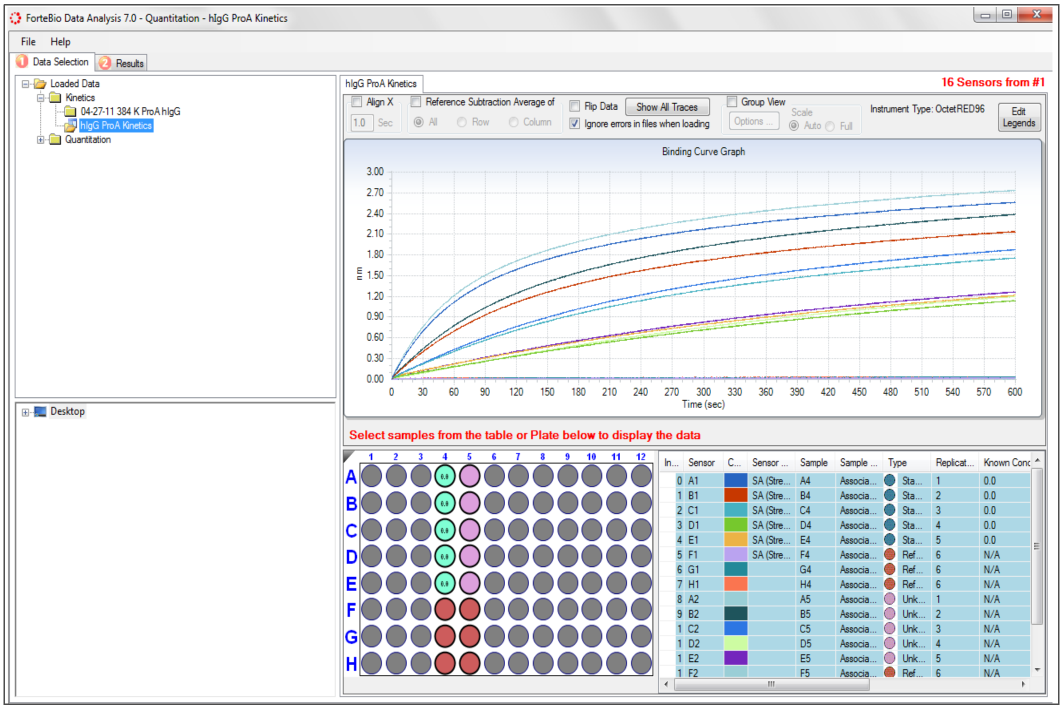

Figure 4-17: Viewing Binding Curves

Closing Experiments

To close an experiment, in the Loaded Data directory tree, right-click the experiment name and select **Remove Run** (see left side of Figure 4-18).

To close all experiments in the Kinetics or Quantitation folder, right-click the folder and select **Remove All** (see right side of Figure 4-18).

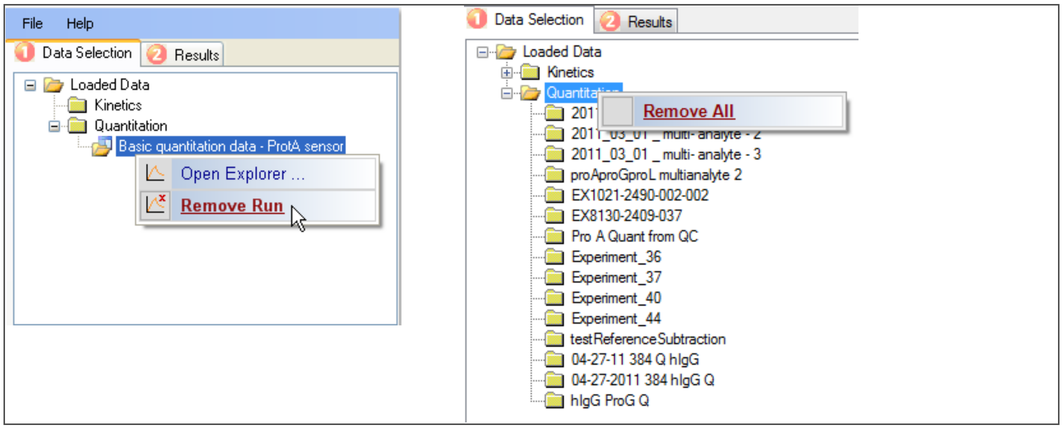

Figure 4-18: *Closing a Selected Experiment (left) or All Experiments (right) in the Quantitation or Kinetics Folder*

ANALYZING DATA

More than one experiment can be opened during a session. If multiple quantitation experiments are open, the analysis includes the data from all of the biosensors that are selected in the **Results** tab.

NOTES:

When analyzing a large number of experiments, batch mode may be more convenient. For more details on batch analysis, see "Processing Batch Quantitation Analysis" on page 68.

In Octet Data Analysis 21 CFR Part 11 software versions 8.2 and higher, a history of changes made to analysis settings are also archived within the experiment folder.

Analyzing Binding Data

To analyze the binding data:

1. Start an analysis session and select the experiment(s) to use.
2. Select a standard curve equation.

3. If the sample plate does not include standards, load a standards file (.fsc).
4. Confirm or set new values for the processing parameters.
5. Calculate the binding rate.

NOTE: *For information on preparing biosensors, see the product insert packed with the biosensors. For information on data acquisition, see the Data Acquisition User Guide.*

Specifying Analysis Settings

NOTE: *In Octet Data Analysis 21 CFR Part 11 software versions 8.2 and higher, whenever you exclude/include wells in the Binding Rate Calculation or exclude/include selected rows, these actions are now logged in the Audit Trail.*

To specify analysis settings:

1. If the experiment includes reference biosensors, click the **Reference Subtraction Average of** check box (Figure 4-19) and select to average the data by one of the following:
   - **All**—Computes the average binding curve from the reference wells and subtracts this average from each sample curve.
   - **Row**—If a row includes both samples and references, the Octet System Data Acquisition software computes the average reference curve for the row and subtracts this curve from the samples in the same row.
   - **Column**—If a column includes both samples and references, the Octet System Data Acquisition software computes the average reference curve for the column and subtracts this curve from the samples in the same column.

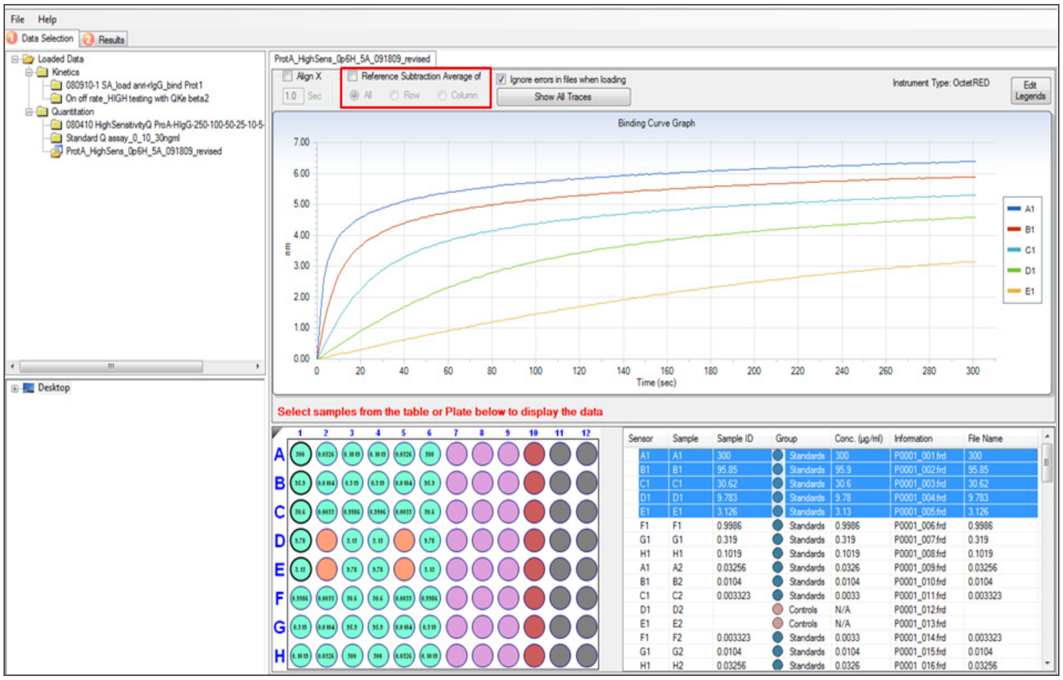

__Figure 4-19:__ Reference Subtraction Average of Methods

2. Confirm the sample designations (for details, see “Changing Sample Designations” on page 40).
3. Confirm the standard concentrations (for details on changing standard concentration, see “Editing Standard Concentration or Well Information” on page 42).
4. Click the **Results** tab and select samples to include in the analysis (for details, see “Excluding/Including Samples from Analysis” on page 41).

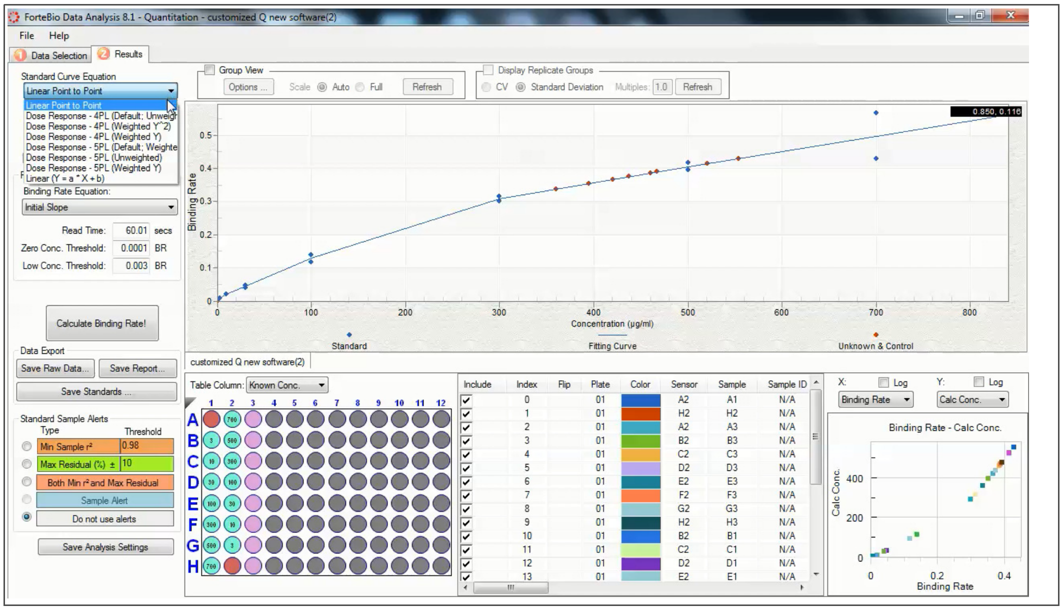

Figure 4-20: Results Window

5. Select a standard curve equation from the drop-down list:
   - **Linear Point to Point**—The Octet System Data Acquisition software connects the points of the standard curve with straight line segments.
   - **Dose Response–4PL (Default; Unweighted)**—A symmetrical dose response curve. No points are weighted during the curve fitting.
   - **Dose Response–4PL (Weighted Y2)**—Anon-symmetrical dose response curve with weighting applied as 1/Y2.
   - **Dose Response–4PL (Weighted Y)**—A non-symmetrical dose response curve with weighting applied as 1/Y (as Y increases, weighting decreases).
   - **Dose Response–5PL (Default; Weighted Y2)**—A non-symmetrical dose response curve with weighting applied as 1/Y2.
   - **Dose Response–5PL (Unweighted)**—A symmetrical dose response curve. No points are weighted during the curve fitting.
   - **Dose Response–5PL (Weighted Y)**—A non-symmetrical dose response curve with weighting applied as 1/Y.

NOTE: *The Octet System Data Acquisition software uses the data from the standards in all of the open experiment(s) to generate one standard curve. Standards with the same concentration are treated as replicates. Remove any standards or samples that you do not want to include in the analysis. (For*

more details on excluding samples, see "Excluding/Including Samples from Analysis" on page 41. Alternatively, an experiment can be analyzed using only the standards from the same experiment plate or from a user-selected experiment).

6. Optional. If multiple experiments are open and you want to analyze an experiment using the standards from the same experiment plate, click the **Calibrate within plate** check box.

***NOTE:** When two quantitation datasets are opened, you have the option to merge data across plates. This happens, by default, if the **Calibrate within plate** check box is not checked. Further to this, replicate groups of standards will be merged and the statistics will be calculated across the entire new replicate group, BUT replicate groups of unknowns will not be merged.*

***NOTE:** You can navigate between multiple experiments using the tabs above the sample plate map.*

7. Optional. Analyze the data using standards from another experiment:

   Standard Curve Within Plate: use this when multiple data sets are loaded. Binding rates and concentrations are calculated according to the standards in the individual plate.

   Standard Curve by Sensor Type: when you select this option, standard curves are generated based on sensor types, and sample binding rates and concentrations are calculated according to the standards with the same sensor type.

   To select a standard curve:

   a. Select either **Standard Curve Within Plate** or **Standard Curve by Sensor Type** (Figure 4-21).

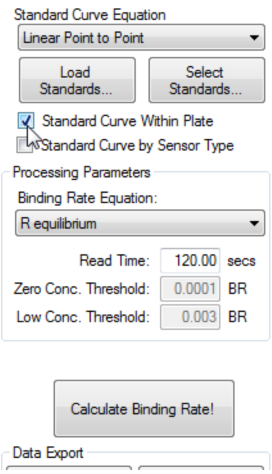

Figure 4-21: *Selecting a Standard Curve*

b. Click **Load Standards** to load previously saved standard curves (for example Standard 1, Standard 2, and Standard 3, see Figure 4-22), and click **Select Standards**.

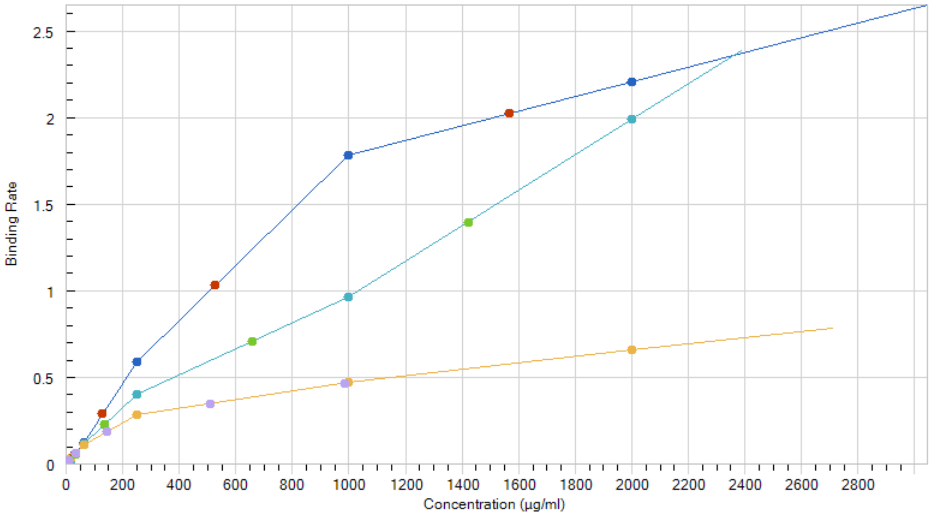

Figure 4-22: *Previously Saved Standard Curves Generated by Sensor Type*

The **Select Standards** dialog box (Figure 4-23) displays with all of the standard curves, including the one in the plate.

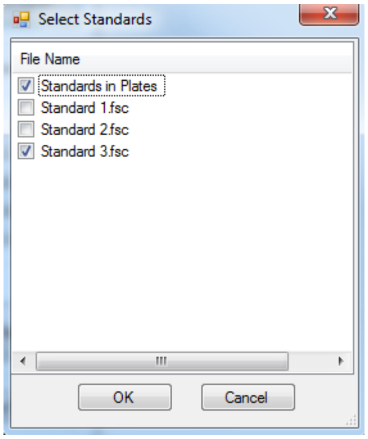

Figure 4-23: Select Standards Dialog Box

c. Click to select the standards you want to use, then click **OK**.

d. In the Standard Curve Equation box (Figure 4-21), click **Calculate Binding Rate!**.

Binding rates/concentrations are calculated based on the selected standard curves. The binding rates of the loaded standard curve will display in the results table, and the Plate column will display the standard curve file name (Figure 4-24).

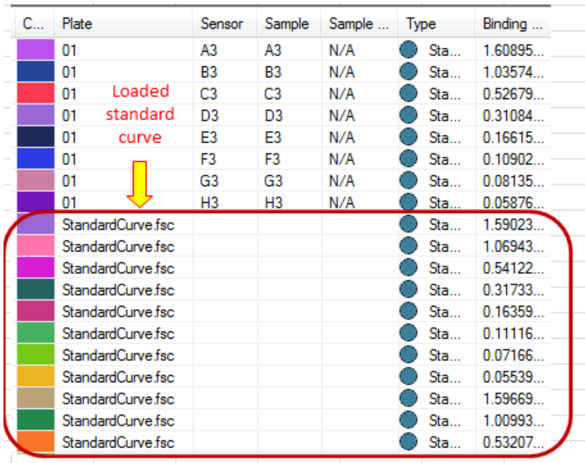

C...	Plate	Sensor	Sample	Sample ...	Type	Binding ...
	01	A3	A3	N/A	Sta...	1.60895...
	01	B3	B3	N/A	Sta...	1.03574...
	01	C3	C3	N/A	Sta...	0.52679...
	01	D3	D3	N/A	Sta...	0.31084...
	01	E3	E3	N/A	Sta...	0.16615...
	01	F3	F3	N/A	Sta...	0.10902...
	01	G3	G3	N/A	Sta...	0.08135...
	01	H3	H3	N/A	Sta...	0.05876...
	StandardCurve.fsc				Sta...	1.59023...
	StandardCurve.fsc				Sta...	1.06943...
	StandardCurve.fsc				Sta...	0.54122...
	StandardCurve.fsc				Sta...	0.31733...
	StandardCurve.fsc				Sta...	0.16359...
	StandardCurve.fsc				Sta...	0.11116...
	StandardCurve.fsc				Sta...	0.07166...
	StandardCurve.fsc				Sta...	0.05539...
	StandardCurve.fsc				Sta...	1.59669...
	StandardCurve.fsc				Sta...	1.00993...
	StandardCurve.fsc				Sta...	0.53207...

Figure 4-24: Loaded Standard Curve

8. Confirm or edit the processing parameter settings (for details, see "Editing Processing Parameters" on page 43)

   - **Binding Rate Equation**—The curve-fitting equation that models the binding data.
     - **Initial Slope**—Calculates the initial slope of the acquired quantitation data (nm/second). Choose this equation for a basic quantitation or basic quantitation with regeneration experiment.

- **R equilibrium**—This equation is recommended for an advanced quantitation experiment that includes amplification. This equation uses the calculated equilibrium, not the initial slope, to model the data.

- **Read Time**—The length of acquired data analyzed (seconds).
- **Zero Conc. Threshold**—Calculated binding rates less than the zero concentration threshold are reported as zero in the results table.
- **Low Conc. Threshold**—Clicking **Calculate Binding rate!** causes the initial rate to be calculated using both a linear and exponential equation. The low concentration threshold determines which value is reported in the results table. If the result from a linear fit is below the low concentration threshold, then the value from the linear fit is reported in the analysis table. If the result from a linear fit is greater than the low concentration threshold, then the value from the exponential fit is reported in the analysis table. Changing this threshold can improve the precision of low concentration samples.

9. Click **Calculate Binding Rate!**

   The standard curve and results table display (Figure 4-25).

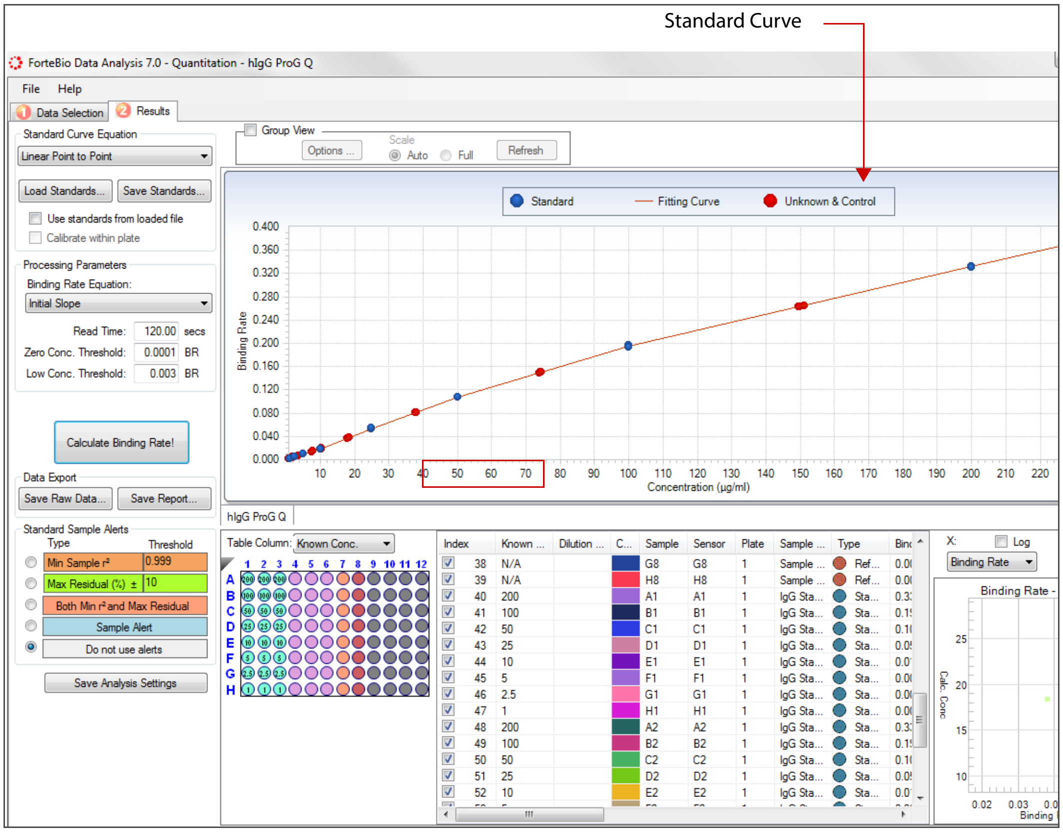

Figure 4-25: Results Window for a Quantitation Experiment

Working with Analyzed Data

On the Results window, the following parameters (see columns) define the analyzed data:

- **Check boxes** toggle the corresponding well's data between inclusion and exclusion from the data analysis:
    - To exclude a sample from subsequent analyses, deselect the corresponding biosensor number in the results table and click **Calculate Binding Rate!** to re-analyze. Or, select one or more wells in the results table, right-click and select **Exclude Wells**.
    - To include a sample in subsequent analyses, checkmark the corresponding biosensor number in the results table and click **Calculate Binding Rate!** to re-analyze. Or, select one or more wells in the results table, right-click and select **Include Wells**.
- **Plate**—A unique number assigned to individual sample plates. If a standard curve was loaded from another experiment, the plate column will display the file name used for the standard curve.
- **Sensor**—The biosensor number.
- **Index**—A unique number assigned to each data point during data analysis.
- **Dilution factor**—The dilution factor used to prepare the assay sample. The dilution factor is multiplied by the well concentration to determine the *calculated concentration*.
- **Well concentration**—The concentration of the analyte determined from the standard curve. The well concentration is multiplied by the dilution factor to determine the *calculated concentration*.
- **Flip**—Inverts the magnitude of all data. Used during analysis of large particles where negative signals can be observed.
- **Information**—Annotations about the sample.
- **Replicate Group**—A set of replicate values organized as a set to facilitate calculation of statistics.
    - BR AVG—The average binding rate of the replicate group
    - BR SD—The standard deviation of the binding rate of the replicate group
    - BR CV—The coefficient of variance of the binding rate of the replicate group
    - Concentration avg—The average concentration of the replicate group
    - Concentration SD—The standard deviation of the concentration of the replicate group
    - Concentration CV—The coefficient of variance of the concentration of the replicate group
- **Sensor Type**—The biosensor chemistry utilized in the assay.
- **Lot Number**—The lot number of the biosensor tray used in the assay
- **Sample**—The well location in the sample plate.

- **Sample ID**—The name of the sample entered in the Octet System Data Acquisition software.
- **Group Type**—The well designation (Standard, Unknown, Reference, or Control).
- **Binding Rate**—The rate of sample binding to the biosensor computed by the Octet System Data Acquisition software using the binding rate equation specified.
- **Known Conc.**—The user-specified standard concentration that was entered during sample plate definition.
- **Calc. Conc.**—The sample concentration computed from the standard curve.
- **Residual (%)**–Residual = (Expected standard concentration—Calculated standard concentration)/Expected standard concentration
- **r2 (COD)**—The r^2 of the curve fit used to determine the binding rate.
- **Well Information**—User-specified notes about the wells.
- **Cycle**—Number of biosensor regeneration cycles.

Sample Plate Map

The Sample Plate Map displays well data, and can be used to select which type of results to display. Select the type of results to display in the sample plate map (**Show Table Column** drop-down list). For example, select **Calc. Conc.** to display the computed sample concentrations on the map.

Results Table

The Results Table displays detailed results for each well in the plate map. To edit table contents, right click in the table and select a menu item (Figure 4-26).

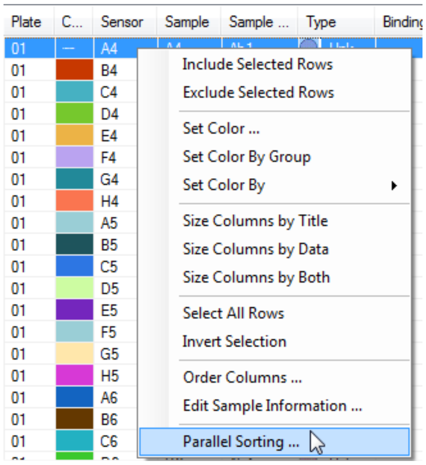

Figure 4-26: *Right-click Results Table Options.*

Analysis results table options:

- **Include Wells**—Removes the "X" in the Include column for the selected biosensors. Re-run the analysis to include these biosensors in the analysis.
- **Exclude Wells**—Adds an "X" in the Include column for the selected biosensors. Re-run the analysis to exclude these biosensors from the analysis.
- **Set Color**—Opens the color palette that enables you to choose a color for the selected results.
- **Set Color By Group**—Color-codes the results according to the groups set in the Grouped View Options dialog box.
- **Set Color By**—Enables you to color-code results according to a user-selected category from the analysis results table.
- **Size Columns by Title**—Automatically sets the column width to fit the column title.
- **Size Columns by Data**—Automatically sets the column width to fit the data.
- **Size Columns by Both** —Automatically sets the column width to fit the data and the column table.
- **Select All Rows**—Selects all biosensors in the table and displays the data in the Fitting view and graphs.
- **Invert Selection**—Changes the wells status so that included wells become excluded wells and excluded wells become included wells. You must re-run the analysis to apply the inverted settings.
- **Set Column Order —**Opens a dialog box that enables you to change the order of the table columns.
- **Parallel Sorting—**Allows you to sort results in parallel based on specific sorting parameters.

Sorting Results Table Entries

The information in the results table can be sorted in ascending or descending order or in parallel based on specific sorting parameters.

1. To sort the entries in ascending, alphanumeric order, click a column header.
2. To sort the entries in descending order, click the column header again.
3. To perform parallel sorting:
   a. Right-click the Results Table and select **Parallel Sorting** (Figure 4-27).

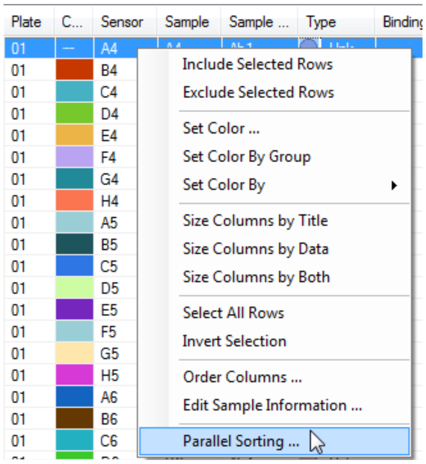

Figure 4-27: *Parallel Sorting Menu*

b. In the Parallel Sorting dialog box, select the desired sorting parameters and click **OK** (Figure 4-28).

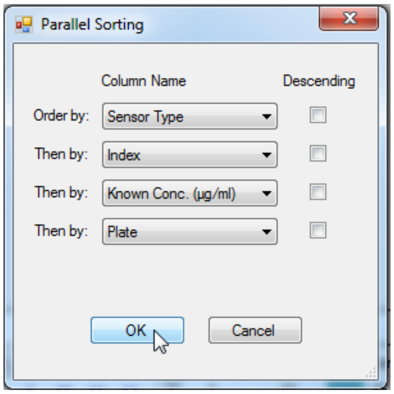

Figure 4-28: *Parallel Sorting Dialog Box*

The Results Table will re-sort based on the parameters and order selected.

Viewing Data

The Interactive Binding Graph and Group View simplify data visualization.

- Selecting one or multiple data points either in the Binding Graph, Sample Plate Map, Results Table, X-Y graph, or Group View highlights the corresponding wells in all views (Figure 4-29).

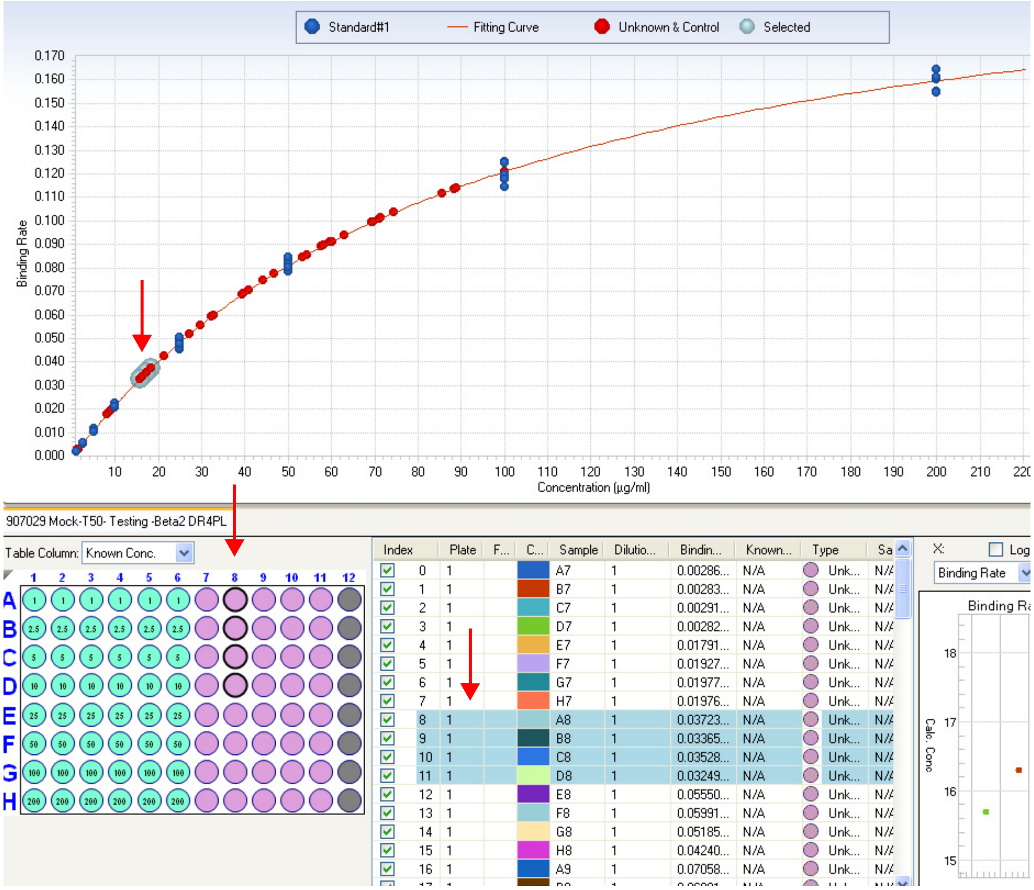

Figure 4-29: Interactive Binding Graph and Group View with Multiple Data Points Selected

- If the data or wells selected were analyzed using different biosensors, separate binding graphs by sensor type will also display (Figure 4-30).

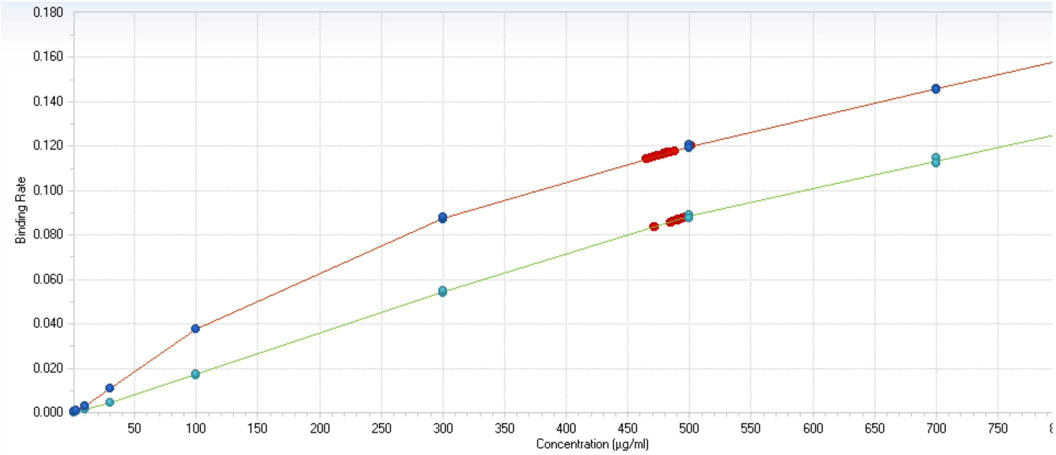

Figure 4-30: Binding Graphs by Sensor Type

Applying Alerts

Applying Standard Alerts

In the Results window, you can select threshold(s) that are applied to the standards. You can also edit the alert threshold value:

- **Min Sample r^2**—The threshold r^2 value for a standard or unknown binding curve. If the r^2 value of a standard or unknown binding curve is less than the threshold value, the standard or unknown sample is highlighted in the results.
- **Max Residual**—Specifies a threshold residual value for standards. If a calculated standard concentration deviates +10% or greater from the expected concentration, the standard is highlighted in the results.
- **Sample Alert**—Specifies highlights data that fit user specified criteria. Thresholds can be set for r2, max residual, or both.
- **Both Min r2 and Max Residual**—Applies both the Min Sample r2 and Max Residual thresholds to the data.
- **Do not use alerts**—Select if you do not want to apply any thresholds to the unknown or standard sample data.

To apply a standards alert:

1. In the sample plate map, select the type of data to display data from the drop-down list.
2. Select a type of alert.

   Samples that do not meet the threshold are highlighted in the sample plate map and results table.
3. Edit an alert threshold value:

   a. Select the cell and enter a new value.

   a. Right-click the cell to display a shortcut menu of editing commands.

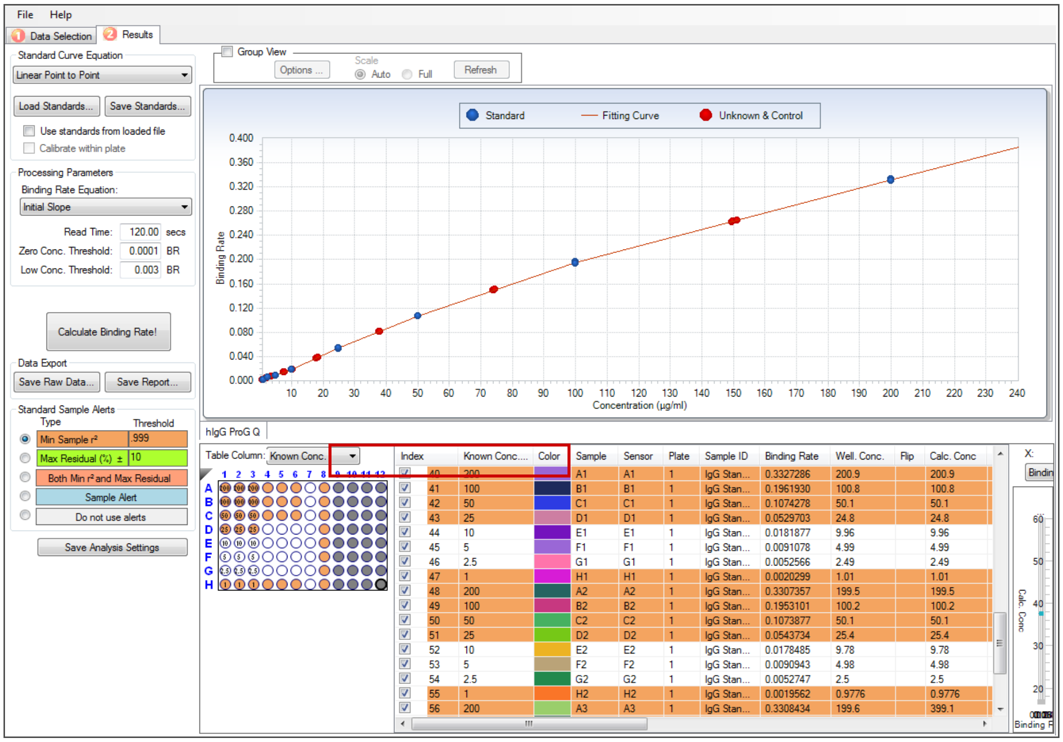

Figure 4-31: Results Window—Displaying Standards Sample Alerts

Applying Sample Alerts

The Sample Alert highlights data that fit user specified criteria.

To apply sample alerts:

1. Set the thresholds for **r2**, **max residual**, or both (Figure 4-32).

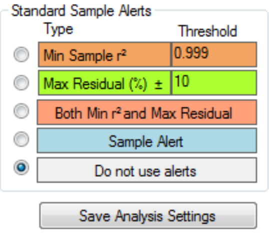

Figure 4-32: Sample Alerts

2. Alternatively, set either a positive or negative threshold on the standard deviation of either the binding rate or the calculated well concentration (Figure 4-33).

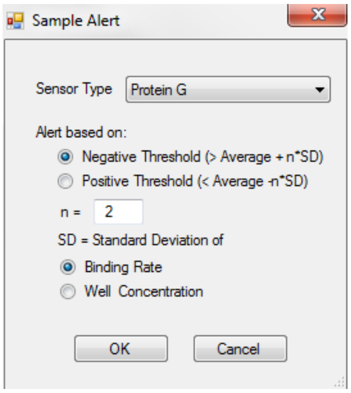

Figure 4-33: Sample Alert Dialog Box

Rows that match criteria specified in the “Sample Alert” are highlighted in the results table of the quantitation experiment (Figure 4-34).

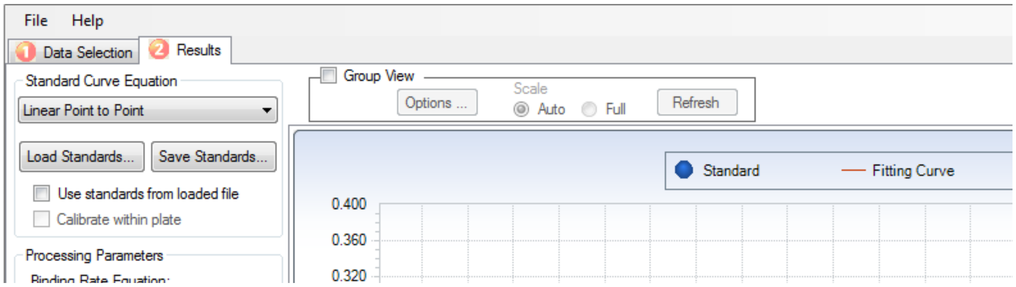

Figure 4-34: Sample Alert Results

The highlighted rows are marked in the Alert column in the analysis table (Figure 4-35).

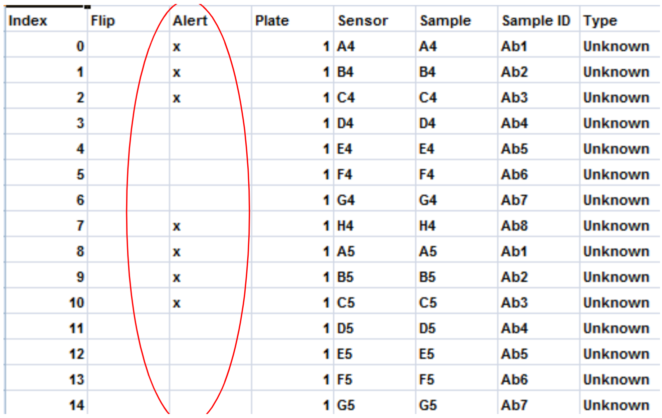

Index	Flip	Alert	Plate	Sensor	Sample	Sample ID	Type
0		x	1	A4	A4	Ab1	Unknown
1		x	1	B4	B4	Ab2	Unknown
2		x	1	C4	C4	Ab3	Unknown
3			1	D4	D4	Ab4	Unknown
4			1	E4	E4	Ab5	Unknown
5			1	F4	F4	Ab6	Unknown
6			1	G4	G4	Ab7	Unknown
7		x	1	H4	H4	Ab8	Unknown
8		x	1	A5	A5	Ab1	Unknown
9		x	1	B5	B5	Ab2	Unknown
10		x	1	C5	C5	Ab3	Unknown
11			1	D5	D5	Ab4	Unknown
12			1	E5	E5	Ab5	Unknown
13			1	F5	F5	Ab6	Unknown
14			1	G5	G5	Ab7	Unknown

Figure 4-35: Alert Column

SAVING STANDARDS DATA

NOTE: *In Octet Data Analysis 21 CFR Part 11 software versions 8.2 and higher, electronic signatures are added to standards data (.fsc) files.*

After analysis, the standards data can be saved for use with other quantitation experiments; to do so:

1. In the **Results** tab (see Figure 4-25: on page 59), click **Save Standards**.
2. In the displayed dialog box, select the file folder and enter a filename (.fsc).
3. Click **Save**.

SAVING ANALYSIS SETTINGS

To save the analysis settings in the Results and Data Selection windows, click **Save Analysis Settings**.

A Settings_DataAnalysis file (.ini or .fsd) is saved in the experiment folder. These settings are displayed the next time the experiment is loaded.

NOTES:

In Octet Data Analysis 21 CFR Part 11 software versions 8.2 and higher, the Settings_DataAnalysis.ini file name extension is Settings_DataAnalysis.fsd. Electronic signatures are also added to data analysis settings (.fsd) files.

In Octet Data Analysis 21 CFR Part 11 software versions 8.2 and higher, a history of changes made to analysis settings are also archived within the experiment folder.

*The Settings_DataAnalysis.ini or. fsd file is also automatically saved when you click **Calculate Binding Rate!** The Settings_DataAnalysis.ini or .fsd file may also be used during batch processing.*

PROCESSING BATCH QUANTITATION ANALYSIS

In batch mode, multiple quantitation data sets may be processed without attended operation. The Octet System Data Acquisition software analyzes experiment data using the data processing parameters in a designated Settings_DataAnalysis.ini or .fsd file. The experiments in a batch can be analyzed using the same or different .ini or .fsd files. The processed data can be saved to either the original data folder or an alternative folder.

Creating a Settings_DataAnalysis.ini File

NOTES:

In Octet Data Analysis 21 CFR Part 11 software versions 8.2 and higher, the Settings_DataAnalysis.ini file name extension is Settings_DataAnalysis.fsd. Electronic signatures are also added to data analysis settings (.fsd) files.

*The Settings_DataAnalysis.ini or .fsd file is also automatically saved when you click **Calculate Binding Rate!***

To create a Settings_DataAnalysis.ini or Settings_DataAnalysis.fsd file:

1. Load and open an experiment that will be included in the batch.

   The Data Selection window displays (Figure 4-36).

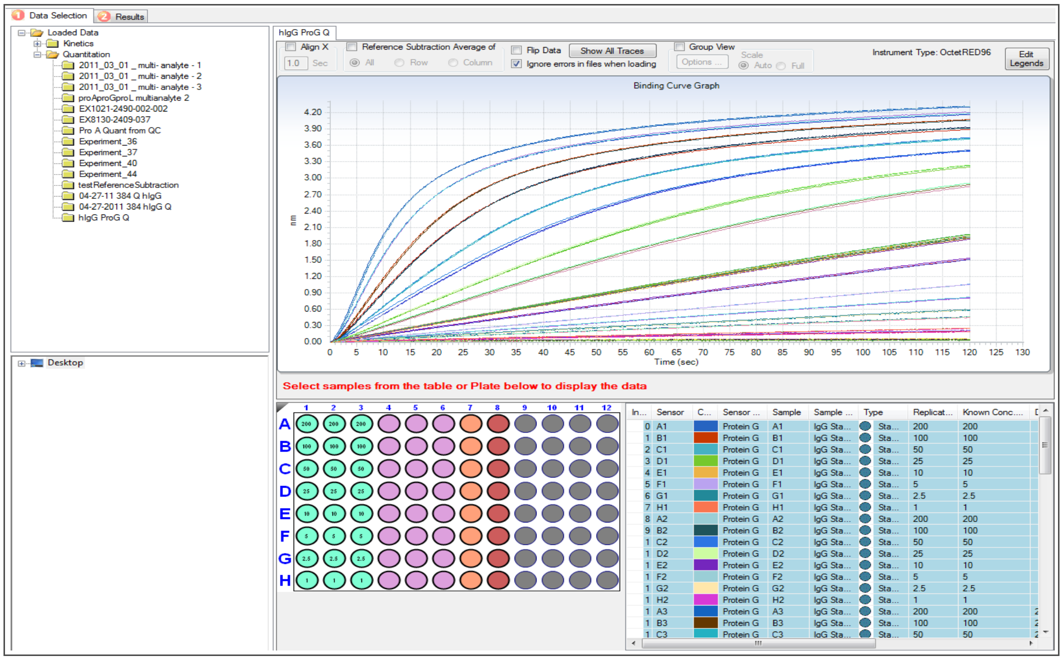

Figure 4-36: *Data Selection Window*

2. Set the data viewing options (refer to Table 4-1 on page 49).

   The Results window displays (Figure 4-37).

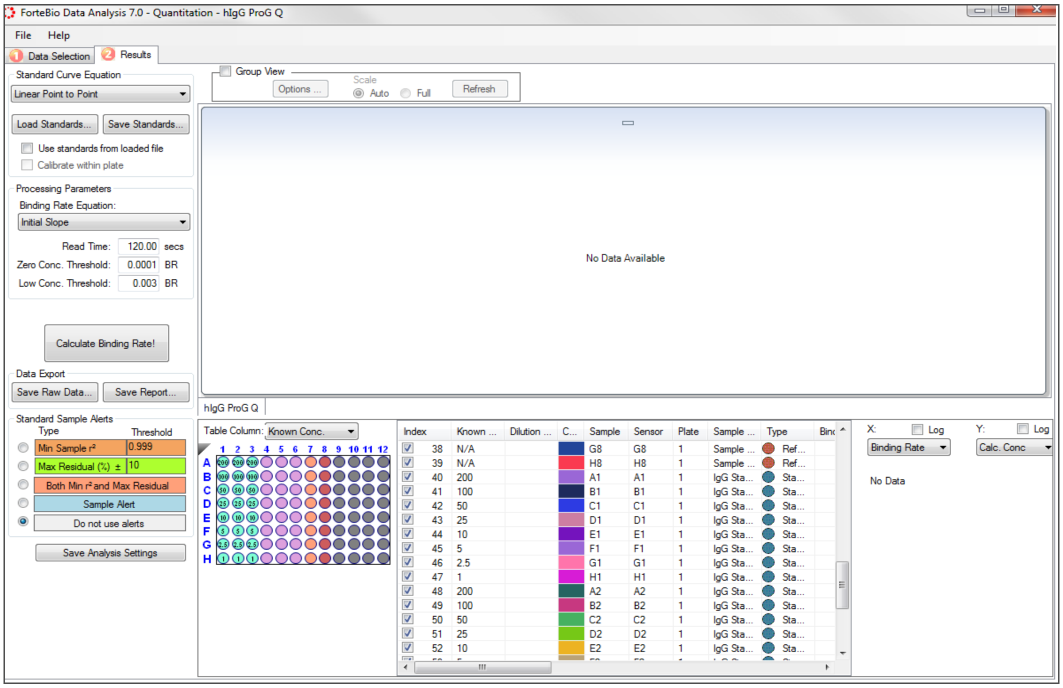

Figure 4-37: Results Window

3. Set the analysis options (standard curve equation, processing parameters, standards sample alerts). For more details, see "Analyzing Data" on page 52.
4. Click **Save Analysis Settings**.

   The .ini or .fsd file is saved in the experiment folder.
5. Optional. Analyze each experiment using a different .ini or .fsd file; repeat steps1–4 to create a file for each experiment in the batch.

Selecting Experiments and Running the Batch Analysis

NOTE: *In Octet Data Analysis 21 CFR Part 11 software versions 8.2 and higher, the Settings_DataAnalysis.ini file name extension is Settings_DataAnalysis.fsd. Electronic signatures are also added to data analysis settings (.fsd) files.*

To select experiments and run the batch analysis:

1. On the menu bar, click **File** > **Quantitation Batch Mode**.

   The Quantitation Batch Model dialog box displays (Figure 4-38).

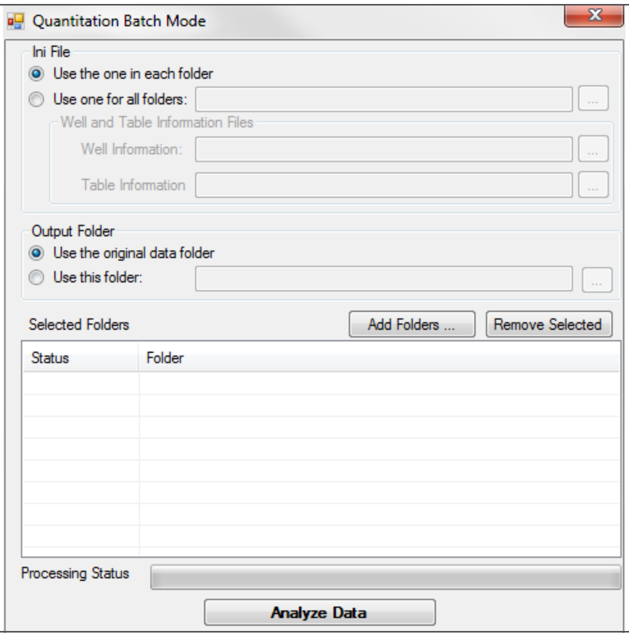

Figure 4-38: Quantitation Batch Mode Dialog Box

2. Set the batch mode options as follows:
   - **.ini or .fsd File**
     - **Use the one in each folder—**Analyzes each experiment using the .ini or .fsd file found in the same experiment folder.
     - **Use one for all folders—**Analyzes all experiments using a user-selected .ini or .fsd file.
     - **Well Information**—Specifies a path within the experiment folder to the **Settings_WellInfo.xml** file. Select only a Well Information file if you modified the original well information by editing (for more information, see “Editing Experiments” on page 40). The **Settings_WellInfo.xml** file can be found in the experiment folder of an experiment that has been edited in the Octet System Data Acquisition software. Select a well information file if the **Use one for all folders** option is selected.
   - **Output Folder**
     - **Use the original data folder**—Analysis results are saved in the experiment folder.
     - **Use this folder—**Saves the analysis results to a user-selected folder.
3. Click **Add Folders** to select the experiments for batch analysis.
4. In the displayed dialog box, select an experiment folder and click **Add**. Repeat to select each experiment in the batch.
5. Optional. Remove experiments from the batch: select the corresponding folders and click **Remove Selected**.

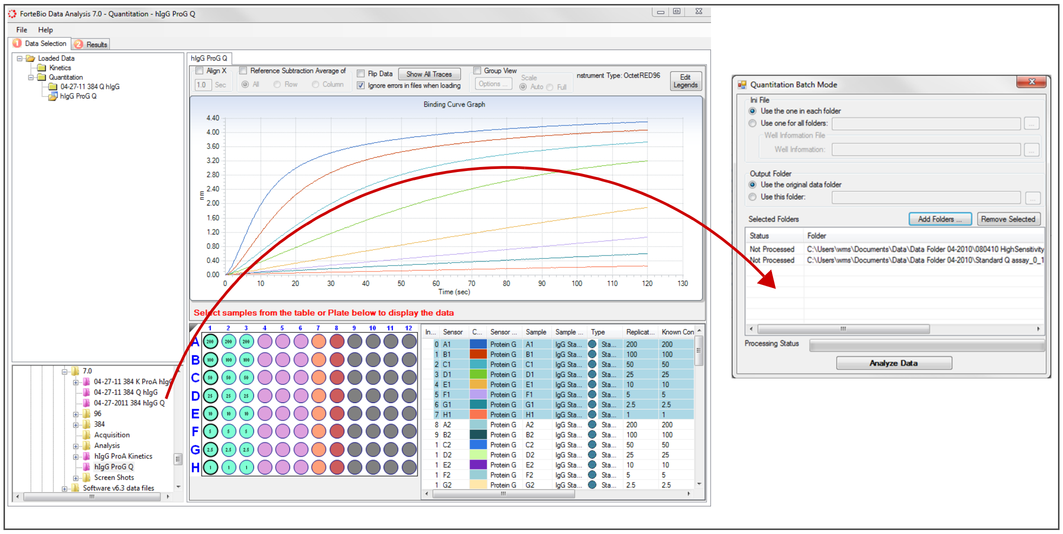

Figure 4-39: *Selecting Experiments for Batch Analysis*

6. Click **Analyze Data**.

EXPORTING DATA

Raw data or quantitation result reports can be exported.

NOTE: *In Octet Data Analysis 21 CFR Part 11 software versions 8.2 and higher, the Summary Worksheet tab of exported reports will now also include the software name, version and the export date and time, and once a report is exported it is locked*

Saving Raw Data

To save raw data:

1. In the Results window (Figure 4-31), click **Save Raw Data**.
2. In the displayed dialog box, select a destination directory.
3. Enter a filename and click **Save**.

   The raw data are saved as a .csv file that can be opened in a spreadsheet application such as Microsoft® Excel® software. In Octet Data Analysis software versions 8.2 and higher, once the report is exported, the contents of the exported .xls file are locked and cannot be changed.

Saving a Quantitation Results Report

All information in the results window can be saved to a report. The Octet System Data Acquisition software generates an Excel spreadsheet (.xls file) that includes the current contents of the results window:

- Calibration curve(s)

NOTE: *An exported standard curve path is displayed on the saved report on the Standard Curve tab.*

- Sample plate map that shows the user-selected type of data
- Results table

NOTE: *If multiple experiments are open, the report will include a separate worksheet for each experiment.*

To save a quantitation results report:

1. On the menu bar, click **File** > **Save Report**, or click the **Save Report** button.

   The Report Selection dialog box displays; see Figure 4-40.

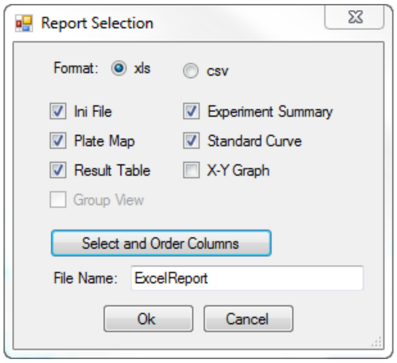

Figure 4-40: *Report Selection Dialog Box*

2. Select the components from the analysis to be exported, enter a file name and click **Save**.

   The report is saved to the data folder. In Octet Data Analysis software versions 8.2 and higher, once the report is exported, the contents of the exported .xls file are locked and cannot be changed.

Changing the Column Order in a Saved Report

You can customize the columns and their order in a saved report. These customized report settings are also saved as the default for future reports.

1. In the Report Selection dialog box (Figure 4-41), click **Save Report** > **Select and Order Columns** > **OK**.

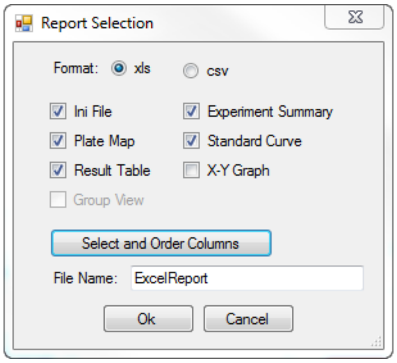

***Figure 4-41:** Report Selection Dialog Box*

2. The Select And Order Columns dialog box displays (Figure 4-42). Select and order the columns, then click **OK**.

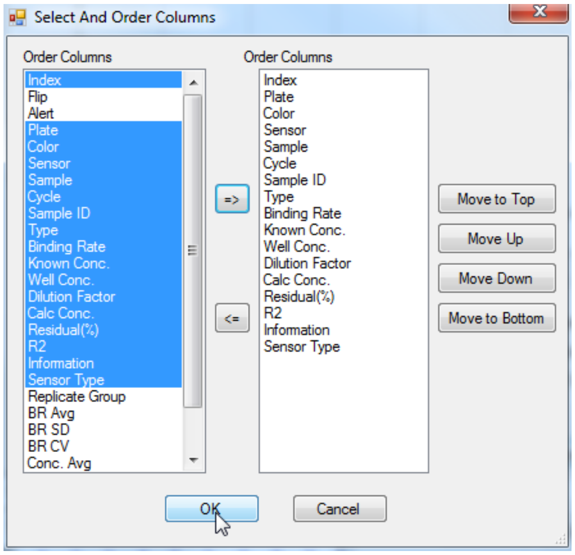

***Figure 4-42:** Select And Order Columns Dialog Box*

3. In the Report Selection dialog box (Figure 4-41), click **OK**. The Results tab will now display the selected columns and the desired column order.

MASKING DATA

The Mask Data feature lets you make a copy of your data folder with all proprietary info such as sample ID, sample info, sensor type and sensor info hidden, or 'masked' in both the .fmf and .frd files.

NOTE: *The Mask Data feature is not available in Octet Data Analysis 21 CFR Part 11 software.*

To do this:

1. In the Data Selection Tab, right click on an experiment in the folder tree.
2. Select **Mask Data** (Figure 4-43).

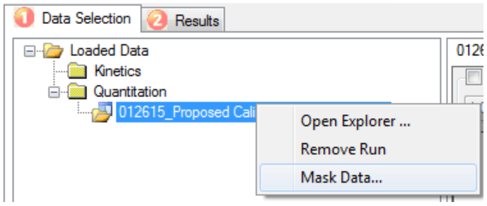

Figure 4-43: *Selecting Mask Data Option.*

A copy of your data folder can then be saved in the desired location.

MULTI-STEP ADVANCED QUANTITATION EXPERIMENTS

The multi-step selection interface for Advanced Quantitation methods increases the flexibility to add more assay steps prior to the Sample or Detection steps for all Octet systems. In addition, all steps in an Advanced Quantitation assay may be viewed and analyzed in the Octet Data Analysis software.

On Octet HTX systems specifically, the Advanced Quantitation application combines the flexibility of the user-selectable Read Head with easier visualization of all the steps in a quantitation assay, including multiple steps preceding the detection or sample step. Users can configure the initial assay steps with pick up of 8, 16, 32, 48 or 96 biosensors separately from the later detection steps. Analysis from 8 or 16 biosensors provides the greatest sensitivity and finer signal resolution whereas data acquisition from 32, 48 or 96 biosensors provides higher throughput.

An Advanced Quantitation experiment enables you to determine sample concentration using a reference set of standards in assays with multiple steps or dips before the Detection step or Sample step (Octet HTX systems only). After an experiment is run, start a data analysis session. The steps to analyze the quantitation data include:

Table 4-2: *Octet Data Analysis Steps for Advanced Quantitation Assays*

Octet Software	Functions
Data Analysis	1. Load and open the experiment data.<br>2. Select a standard curve equation.<br>3. If the experiment does not include standards, load a standards file (.fsc).<br>4. Confirm or set new values for the processing parameters.<br>5. Calculate the binding rate.

The Advanced Quantitation Analysis feature provides an easier visualization of all the steps in the assay, including multiple steps preceding the detection or sample step.

Starting a data analysis session and viewing the raw data are explained “Working with Experiments” on page 38.

1. After loading an Advanced Quantitation data file, open the Data Selection (Tab 1).
2. Click on a step on the right graph to show an enlarged graph for the selected step on the left:

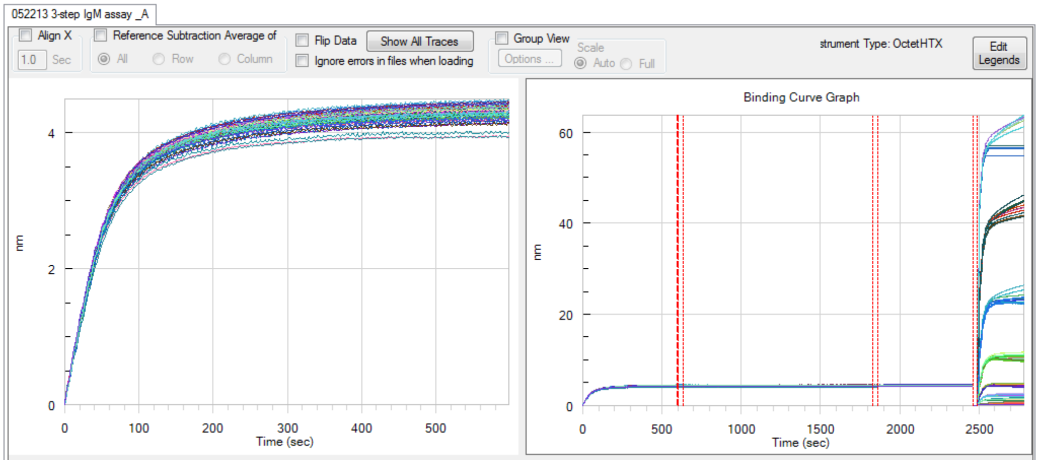

Figure 4-44: *Viewing Step Graph Data*

3. Click on individual sample wells from the table or plate to display individual well data:

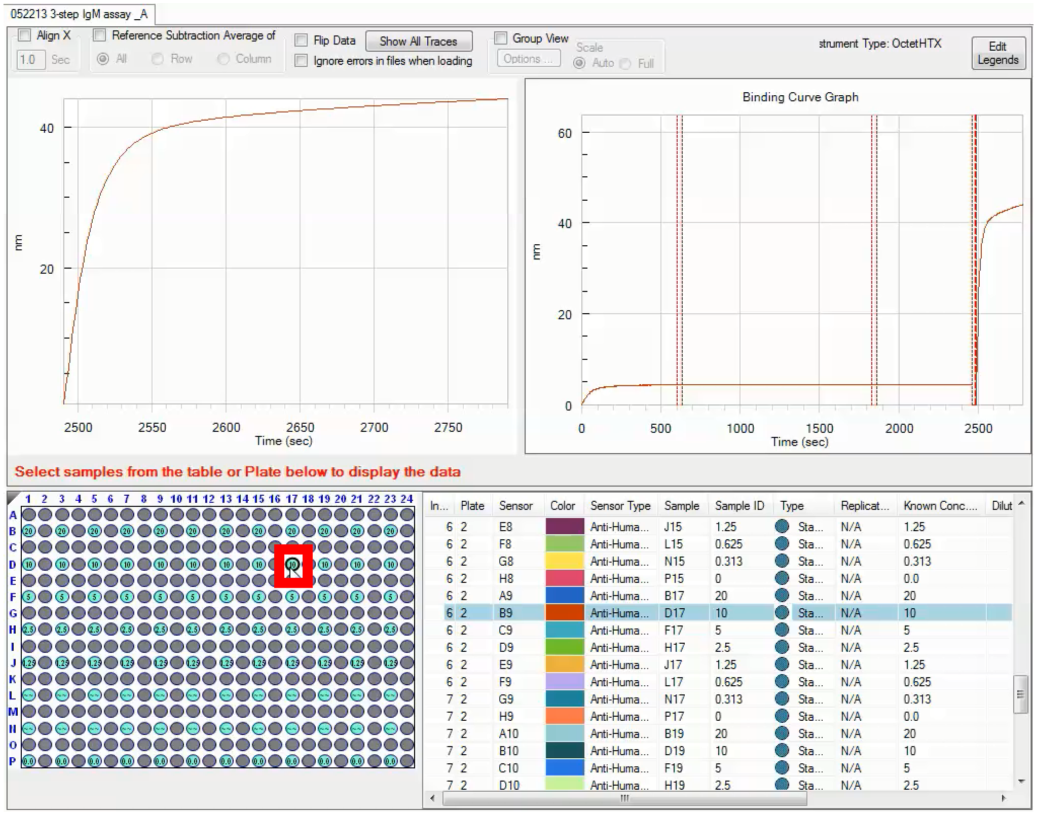

Figure 4-45: Viewing Well Data

4. Click on Tab 2 (Results). Analysis will be done on the selected step shown in the left graph, with the Detection step selected as the default step. The Plate Map shows sample locations only, the Plate Table shows the selected detection step only, and the Results Table on Tab 2 will update as a step is selected:

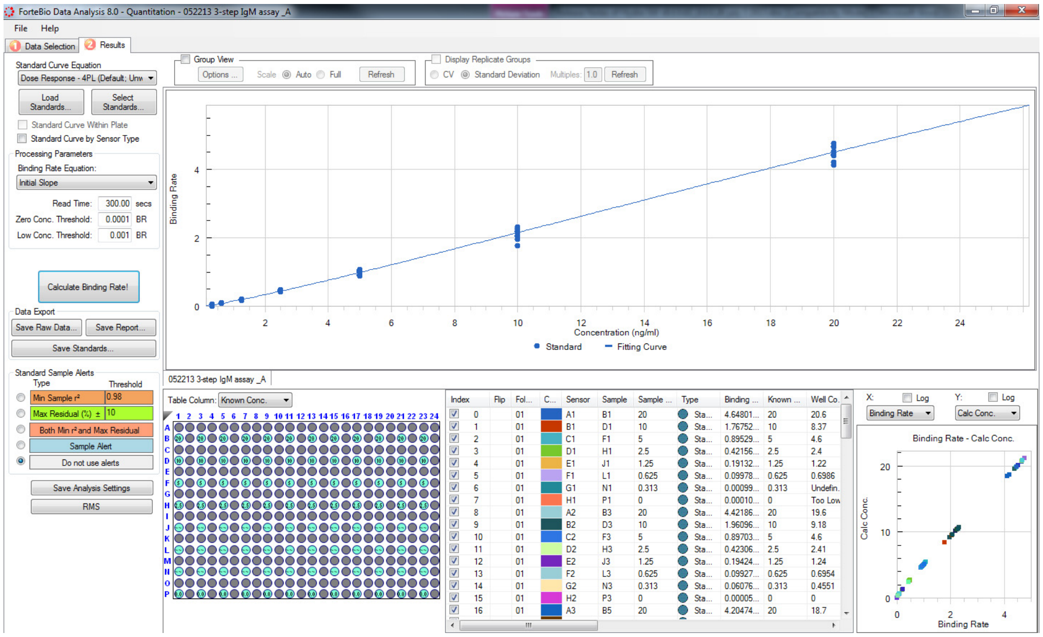

__Figure 4-46:__ Viewing Analyzed Data

***NOTE:** The Sample plate and the Reagent plate are now referred to as "Plate 1" and "Plate 2" in the software.*

5. Proceed with data analysis as described in "Analyzing Data" on page 52.

CHAPTER 5:
Basic Kinetics Analysis

Working with Experiments 80
Working with Raw Data 89
Processing Kinetic Data 95
Kinetics Analysis 113
Processing Batch Kinetics Analysis 120
Kinetics Analysis Results 124
Working with Graphs 142
Data Export Options 144
Masking Data 145
Generating a Report 146
Epitope Binning 153

WORKING WITH EXPERIMENTS

Starting a Basic Kinetics Experiment

A basic kinetics experiment enables you to determine the association and dissociation rate of a molecular interaction. After an experiment is run, start a data analysis session (double-click the icon on the desktop).

There are several ways to start a basic kinetics experiment:

- Use the Experiment wizard.
- Open a method file (fmf). An experiment method file (.fmf) is automatically saved after you define and run an experiment.
- On the menu bar, click **Experiment** > **Templates** > **Kinetics**.

NOTE: *When using the 21 CFR Part 11 version of the Octet System Data Acquisition software, only 21 CFR Part 11-compliant experiments and re-analyze generated using the 21 CFR Part 11 version of the software can be opened. Files generated using the non-compliant version of the software or with a non-compliant system cannot be opened, and a message indicating this will be presented.*

Loading an Experiment for Analysis

A data analysis session can be used to:

- Load and analyze an experiment.
- Re-analyze an experiment.

To load an experiment for analysis:

1. On the desktop, click the icon. Or, click the Windows **Start** button and select **All Programs** > **ForteBio** > **ForteBio Data Analysis 7.0**.

   The Data Selection window displays.

2. Load an experiment:

   a. Right-click the experiment folder in the workstation directory tree and select **Load Folder**; or, on the menu bar, click **File** > **Load a Folder**.

   b. In the Load Folder dialog box, enter the folder name or click the **Browse** button ... and select the desired folder, and then click **Load**.

   The experiment is added to the Loaded Data directory tree (Figure 5-1).

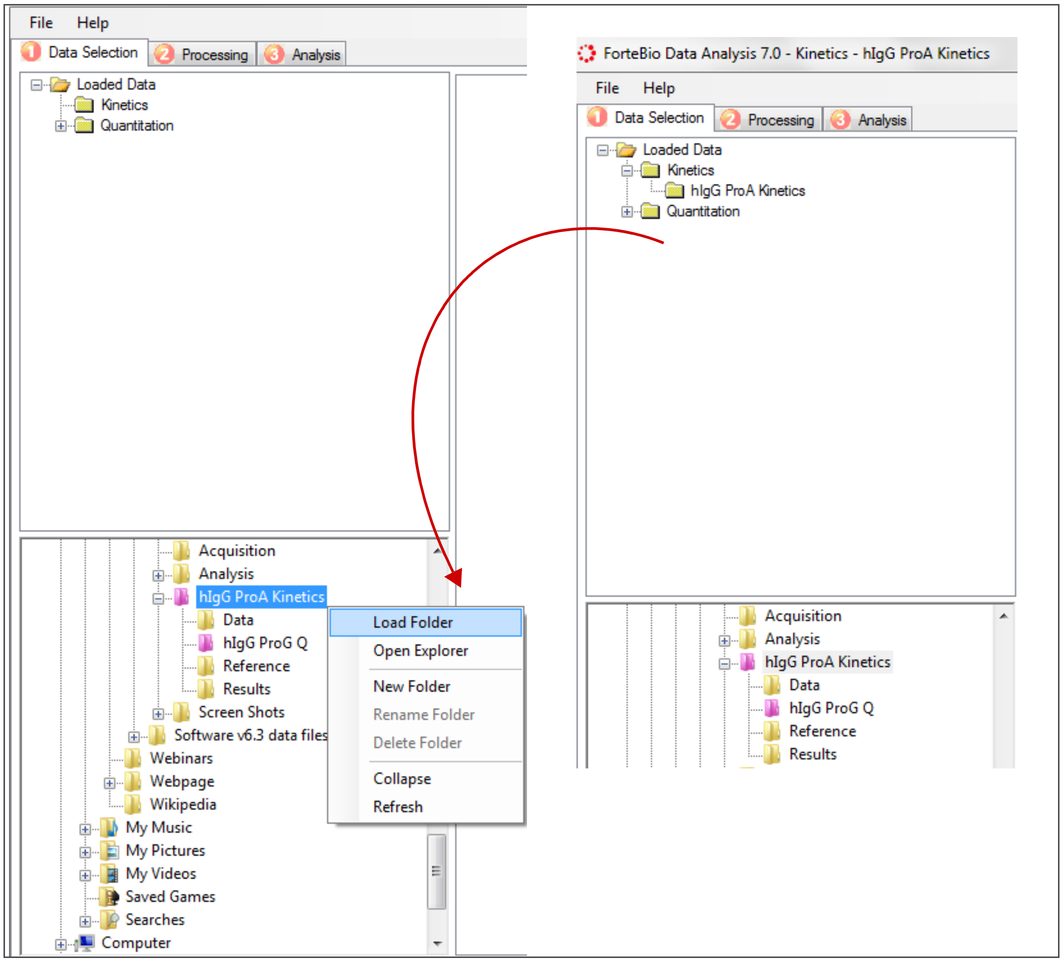

Figure 5-1: *Data Selection Window—Loading an Experiment*

3. In the Loaded Data directory, click an experiment name to open it.

   The experiment summary appears (Figure 5-2).

NOTE: *Multiple kinetics can be loaded, but only one kinetic can be open at a time. The icon in the Loaded Data directory tree indicates the open experiment.*

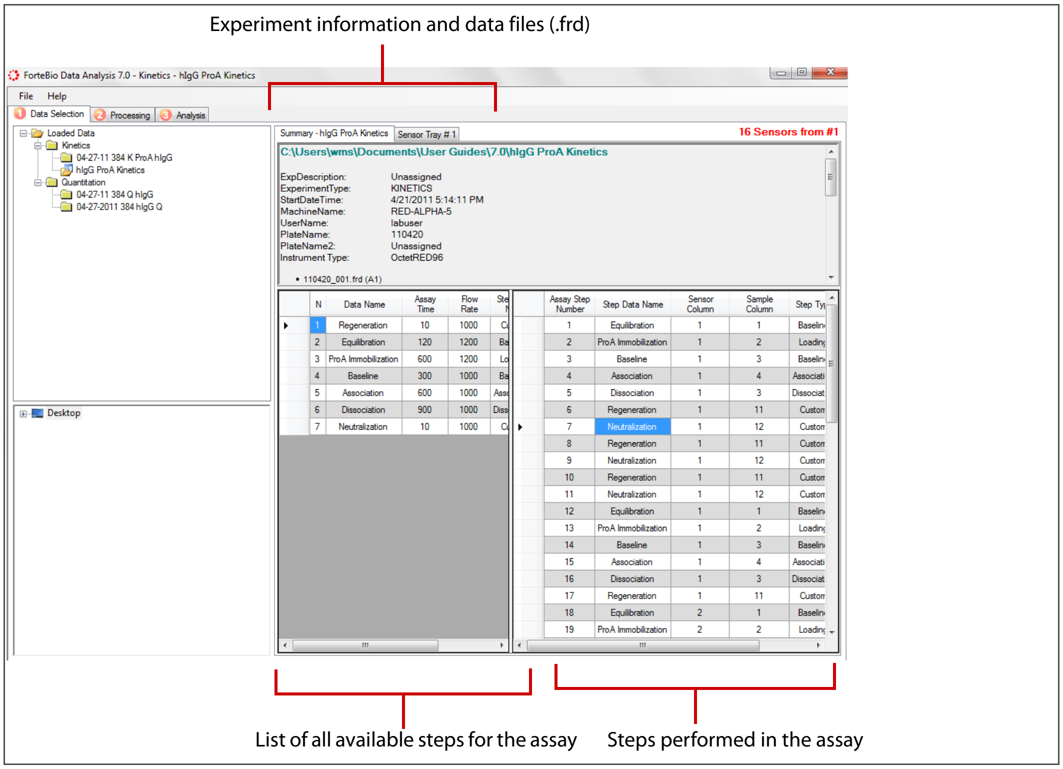

Figure 5-2: Data Selection Window—Displaying Experiment Summary Information

4. Optional. If any step types have been incorrectly assigned in the Octet System Data Acquisition software, change them before beginning analysis. To do so, right-click the step and select the correct step type from the shortcut menu (Figure 5-3).

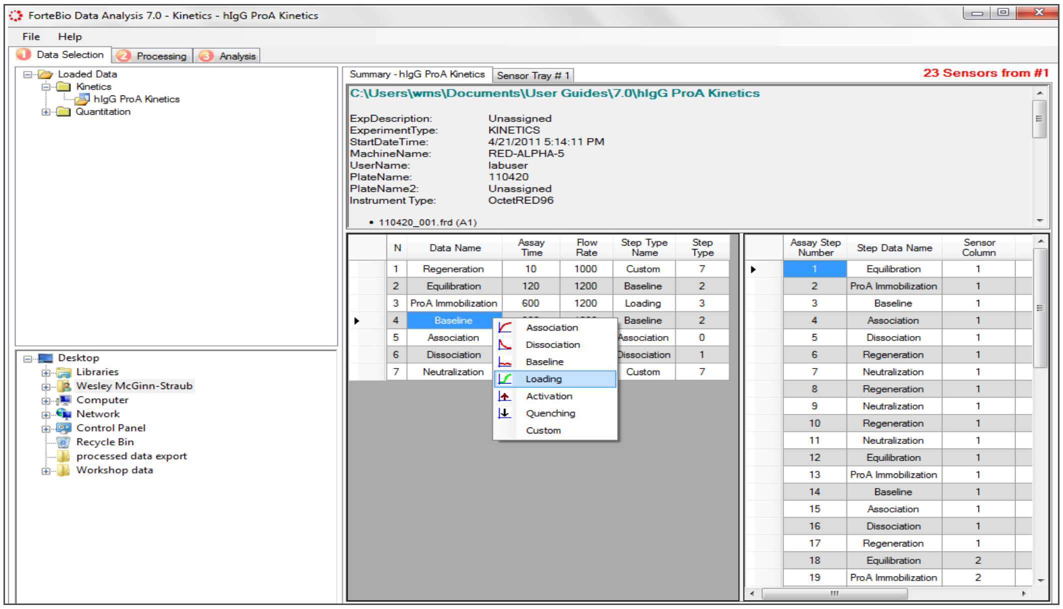

Figure 5-3: Changing a Step Type

5. In the Sensor Tray tab, confirm the biosensors to be analyzed.

   The first group of biosensors is automatically selected for analysis.

   Biosensors that are assigned to the same type of assay step and have the same assay (step) times are displayed in the same color, providing convenient identification of multiple types of assays executed in one experiment and using one biosensor tray (red 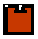 in Figure 5-4). Only biosensors of the same color processed simultaneously. This provides a convenient way to identify multiple experiments on a biosensor tray.

6. Select particular biosensors for the analysis:

   a. Click a biosensor. To select non-adjacent biosensors, press and hold the **Ctrl** key while you click the biosensors.

   b. Select a column(s) or row(s). To select non-adjacent columns or rows, press and hold the **Ctrl** key while you click the columns or rows.

   c. Draw a box around the biosensors using the mouse.

      The number of biosensors selected for analysis in the current tray will be displayed in the upper-right corner of the Sensor Tray tab.

7. Choose the **Ignore error in files** option when loading to load data files regardless of whether a runtime error was identified. This enables data visualization even if a sensor error occurred during runtime.

***NOTE:** Only the selected biosensors will be available in the Processing window.*

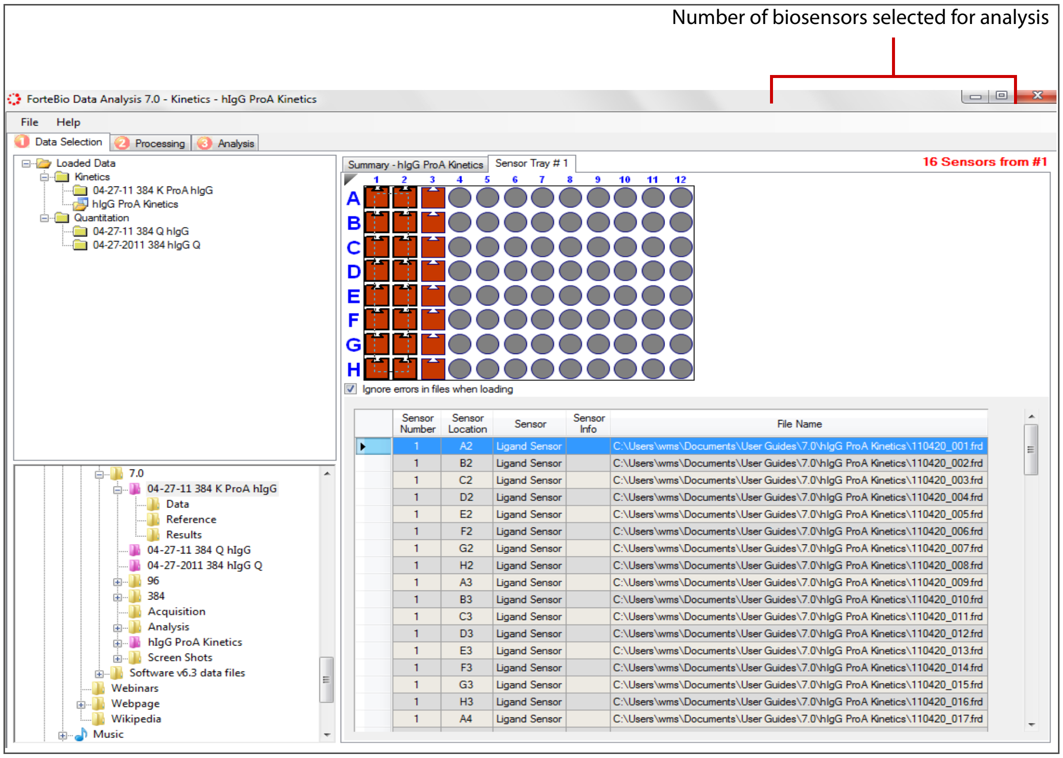

Figure 5-4: Selecting Biosensors for Analysis

Analyzing Binding Data

To analyze the binding data:

1. Start an analysis session and select the experiment(s) to use.

   a. Confirm or change the biosensor and sample type selected for the analysis.

   b. Process the data (compile binding curves with or without reference subtraction).

2. Analyze the binding data:

   - Curve fitting analysis—Determines the kinetic constants k_a, k_d and the affinity constant K_D from fusing a specified binding model.
   - Steady state analysis—Determines the affinity constant K_D from the calculated or measured equilibrium response.

3. View the results in graphical and tabular formats.

4. Export the results and generate a report.

Editing Experiments

The Octet System Data Acquisition software enables you to change sample designations, standard concentrations, or exclude samples from analysis. For example, you can exclude a standard that does not meet the sample r^2 or residual threshold, then re-analyze the data. You can also modify some processing parameters.

> ***NOTE:*** *If you are using the 21 CFR Part 11 version of the Octet System Data Acquisition software, editing options are disabled.*

Changing Sample Designations

To change sample designations:

1. Click the **Data Selection** tab, then do either of the following:
   - In the *sample plate map,* select the well(s), right-click and select an option from the shortcut menu.
   - In the *results table*, right-click a table cell in the Well Type column, then make a selection from the drop-down menu.

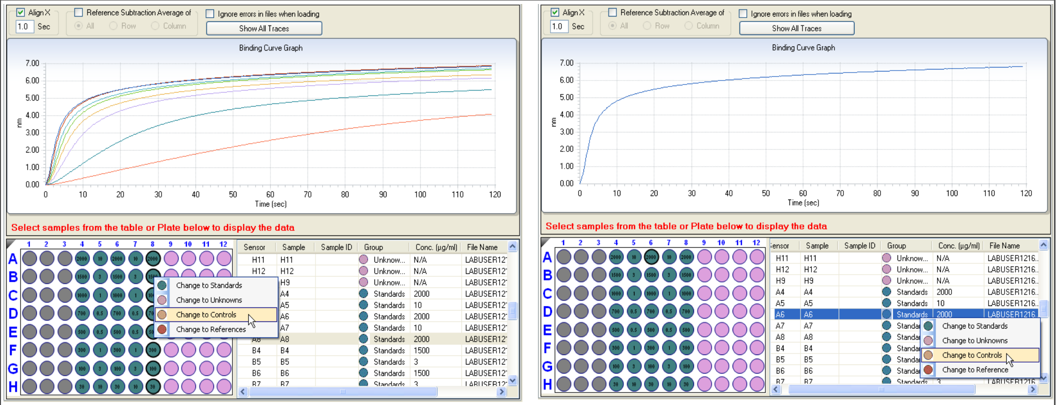

Selecting wells in the sample plate map — Selecting a cell in the Well Type column of the results table

Figure 5-5: *Changing Sample Designations*

Excluding/Including Samples from Analysis

To toggle sample analysis in the Results window, perform one of the following tasks:

- In the *sample plate map,* select the well(s), right-click and select **Exclude Wells** to remove the samples from the analysis. If the selection is already excluded from analysis, select **Include Wells** to return the samples to the analysis.
- In the *results table*, to exclude wells, deselect the check box in the first column or right-click selected rows and select **Exclude Wells**. If the selection is already excluded from analysis, click the check box for the desired rows or right-click desired rows and select **Include Wells**.

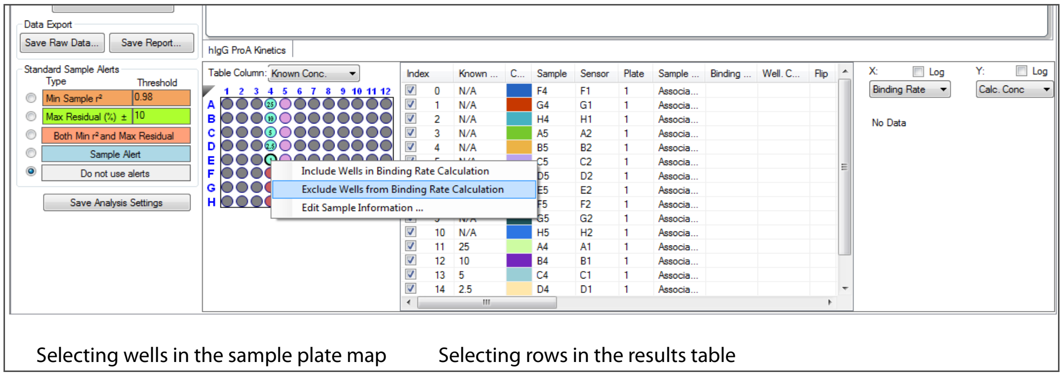

Figure 5-6: Excluding Samples from Analysis

Editing Sample Information

The following information in the table can be edited directly in the table in the Processing Tab, under sensor selection. Just click in the cell, then type to make changes (Figure 5-7):

- Sample ID
- Molar Conc

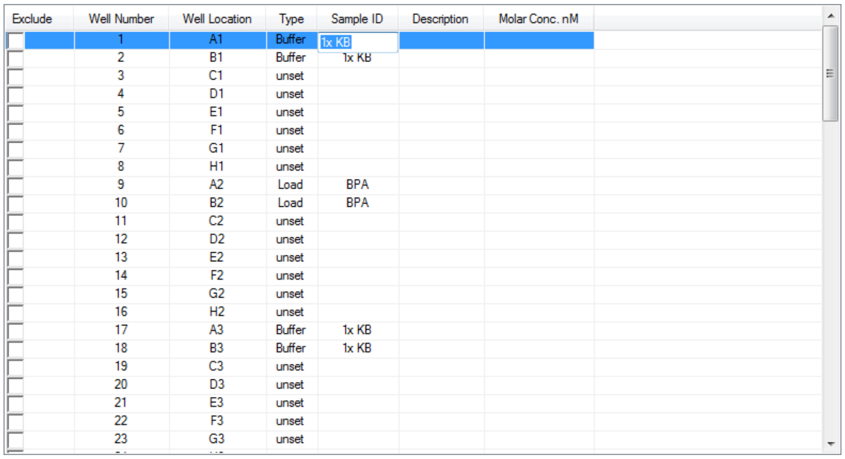

Figure 5-7: *Editing Cells in Data Table*

Sample information can also be edited by right-clicking the cell in the table, then selecting **Edit Sample Properties** in the right-click menu (Figure 5-8).

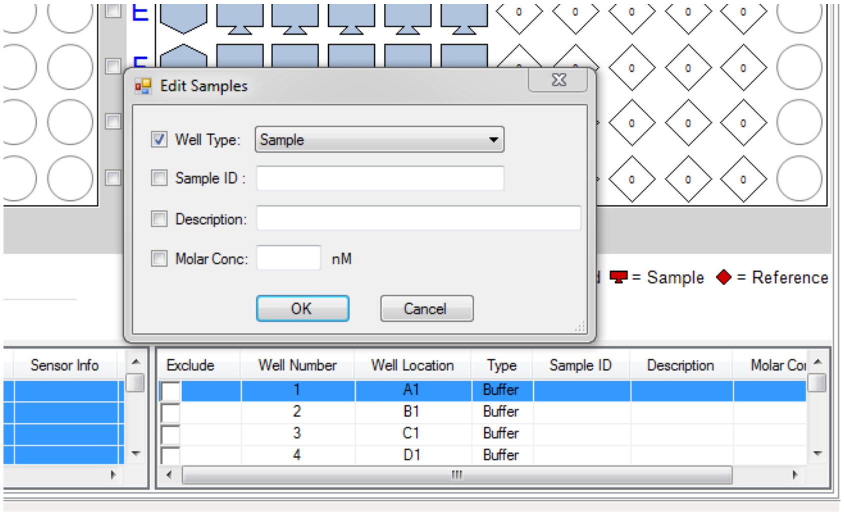

Figure 5-8: *Edit Sample Information Dialog Box*

Closing Experiments

To close an experiment, in the Loaded Data directory tree, right-click the experiment name and select **Remove Run** (see left side of Figure 5-9).

To close all experiments in the Kinetics or Quantitation folder, right-click the folder and select **Remove All** (see right side of Figure 5-9).

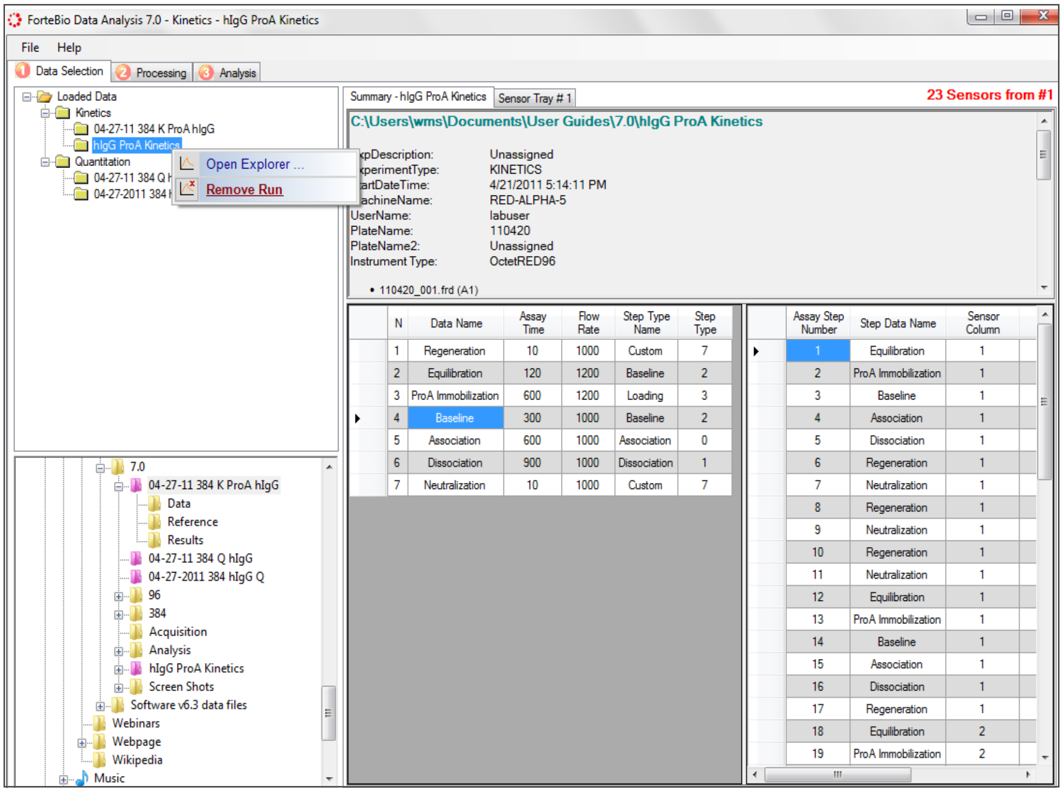

Figure 5-9: Closing a Kinetics Experiment

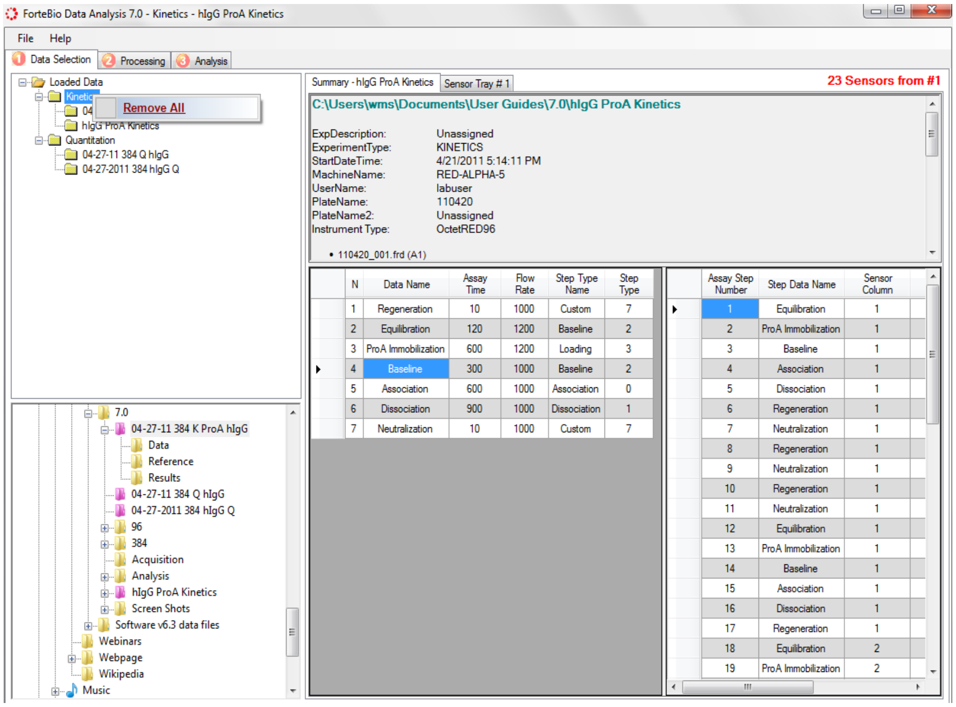

Figure 5-10: Removing All Kinetics Experiments from Processing

WORKING WITH RAW DATA

Viewing Raw Data

In the Processing window, the Raw Data view enables you to conveniently examine the binding data.

To view raw data:

1. In the Processing window, the **Processing** tab and confirm that the **Raw Data View** option is selected.

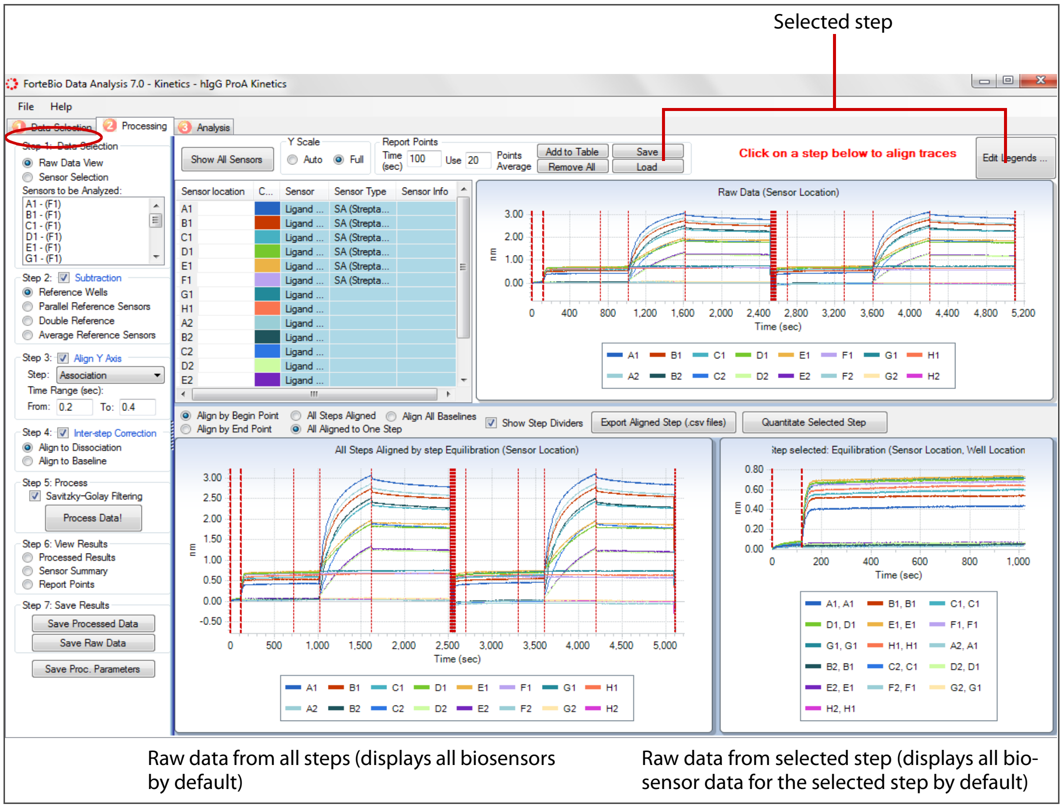

***Figure 5-11:** Processing Window—Raw Data View Selected*

2. View the step data and align the binding curves (click a step bounded by ¦ ¦ lines in the Raw Data chart).

***NOTE:** The ¦ ¦ lines represent individual assay steps. Populate the step charts (below) with detailed views of an individual step by clicking inside the step boundaries.*

The “All Steps Aligned by step xx” chart displays all of the assay data aligned by the step selected in the Raw Data chart (Figure 5-12).

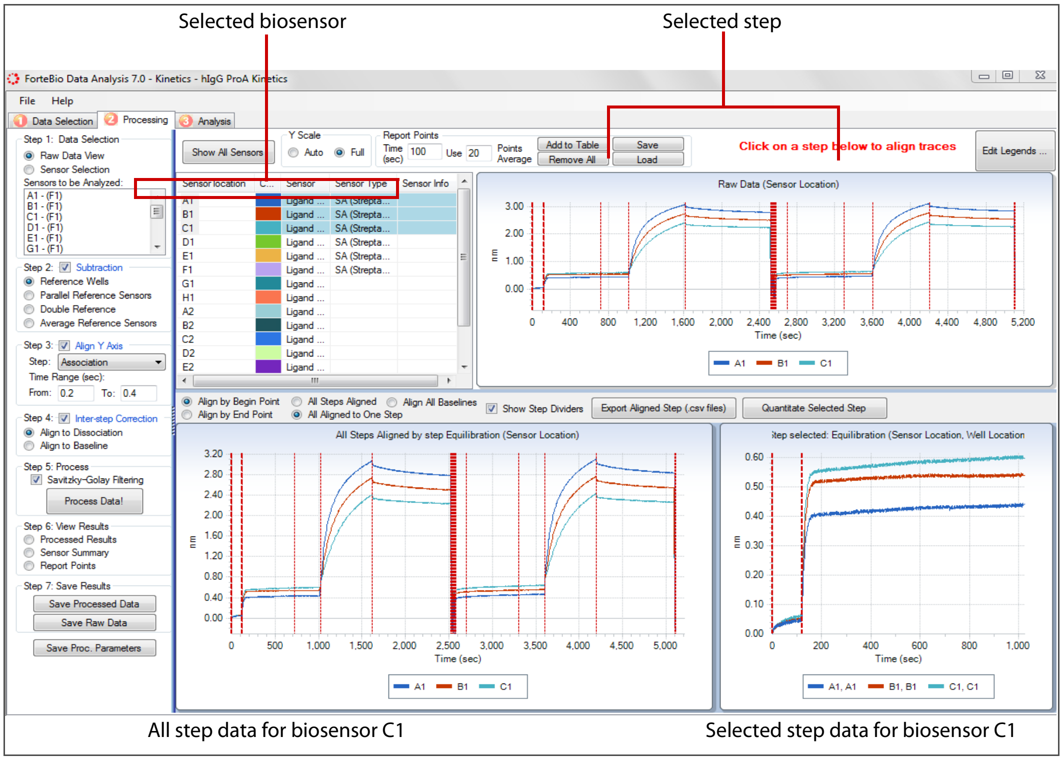

Figure 5-12: *Processing Window (Raw Data View)—Displaying Data from a Single-Selected Biosensor (C1)*

3. Select which biosensor data to display:

    - To view the data from a selected biosensor, click the biosensor in the table.

      The step charts will display all of the binding data for the selected biosensor and the data from the selected step aligned starting at $y = 0$.

    - To display data from multiple biosensors in the step charts, select the biosensors in the Sensor location list.
    - To select a contiguous block of biosensors from the list, hold down the **Shift** key and click the first and last biosensors in the group.
    - To select non-contiguous biosensors, hold down the **Ctrl** key and click the desired biosensors.
    - To include all biosensor data in the step charts, click **Show All Sensors**.

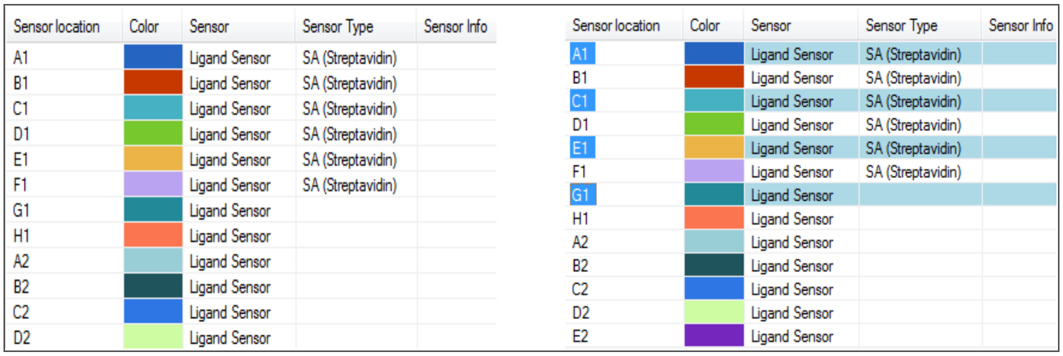

Sensor location	Color	Sensor	Sensor Type	Sensor Info
A1		Ligand Sensor	SA (Streptavidin)	
B1		Ligand Sensor	SA (Streptavidin)	
C1		Ligand Sensor	SA (Streptavidin)	
D1		Ligand Sensor	SA (Streptavidin)	
E1		Ligand Sensor	SA (Streptavidin)	
F1		Ligand Sensor	SA (Streptavidin)	
G1		Ligand Sensor		
H1		Ligand Sensor		
A2		Ligand Sensor		
B2		Ligand Sensor		
C2		Ligand Sensor		
D2		Ligand Sensor		

Sensor location	Color	Sensor	Sensor Type	Sensor Info
A1		Ligand Sensor	SA (Streptavidin)	
B1		Ligand Sensor	SA (Streptavidin)	
C1		Ligand Sensor	SA (Streptavidin)	
D1		Ligand Sensor	SA (Streptavidin)	
E1		Ligand Sensor	SA (Streptavidin)	
F1		Ligand Sensor	SA (Streptavidin)	
G1		Ligand Sensor		
H1		Ligand Sensor		
A2		Ligand Sensor		
B2		Ligand Sensor		
C2		Ligand Sensor		
D2		Ligand Sensor		
E2		Ligand Sensor		

Figure 5-13: *Selecting contiguous (left) and discontiguous (right) biosensors in the Sensor location list.*

4. Align the data (select an alignment option):
   - **Show All Steps Aligned**—Aligns all steps. Aligns baseline steps and association steps to the start of the step. Aligns all dissociation steps to the end of the step.
   - **All Aligned to One Step**—Enables the following options:
     - **Align by Begin Point**—Aligns the displayed curves to the start of the currently-selected step.
     - **Align by End Point**—Aligns the displayed curves to the end of the currently-selected step.
   - **Align All Baselines**—Aligns all steps according to the baseline step data.

Exporting Raw Data

To export raw data:

1. Click **Export Aligned Step (.csv files)**.
2. In the displayed dialog box, navigate to a destination directory, enter a filename, and click **Save**.

Quantitating Raw Data

To quantitate raw data from a selected step:

1. In the Raw Data view, select the step to quantitate and click **Quantitate Selected Step** (Figure 5-14).

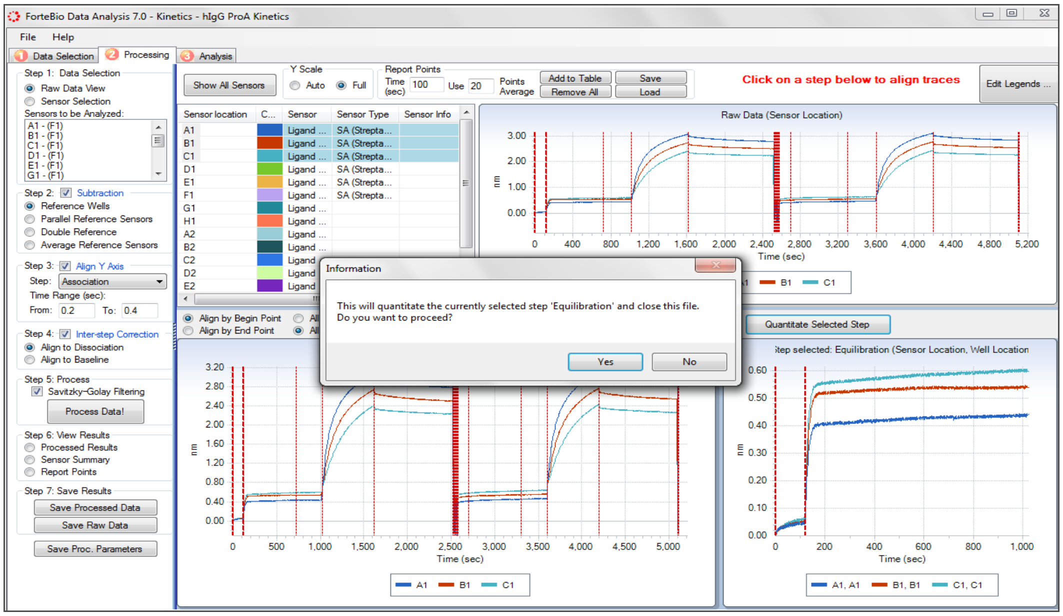

Figure 5-14: Processing Window—Raw Data View—Quantitating a Selected Step

2. In the system prompt that displays, click **Yes** to proceed with the quantitation.

   The selected step data is displayed in a quantitation window (Figure 5-15). By default, samples with defined concentration values are designated as standards and samples without defined concentration values are designated as unknowns.

NOTE: In quantitation mode, sample information can be modified.

For more details on viewing the quantitation data, see "Viewing Binding Curves" on page 48.

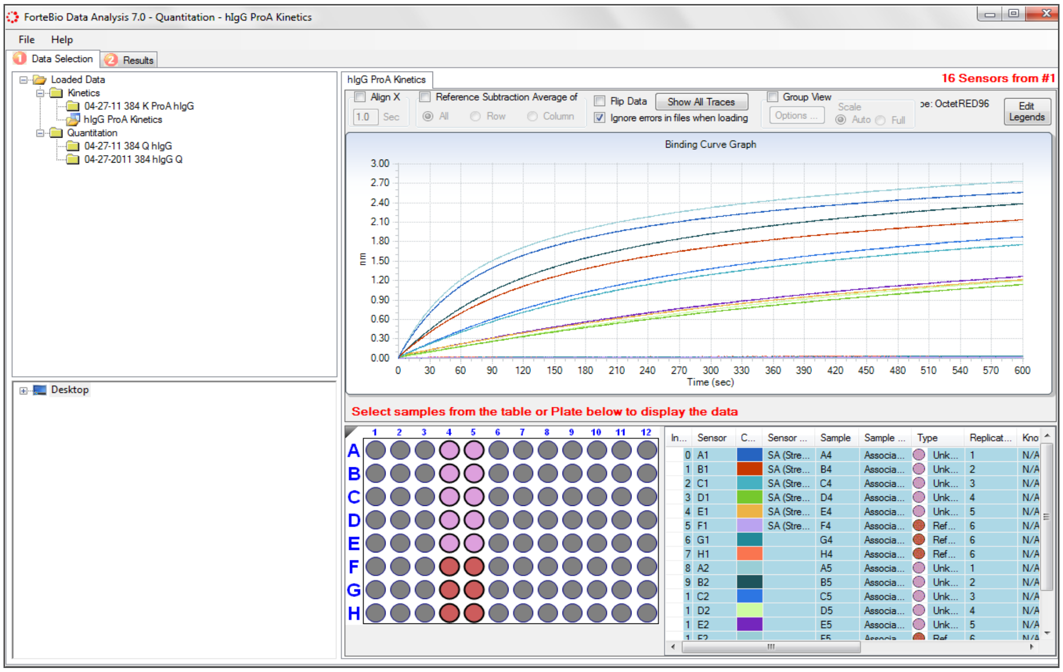

Figure 5-15: *Quantitation Window—Displaying Selected Step Data from a Kinetic Assay*

NOTE: *When step data from a kinetic assay is open, additional quantitation experiments cannot be opened.*

3. In the Results window, select a standard curve equation, set the processing parameters, and then click **Calculate Binding Rate!**

   The binding rates will be calculated and displayed; see Figure 5-16.

   For more details on analyzing quantitation data, see “Loading an Experiment for Analysis” on page 80

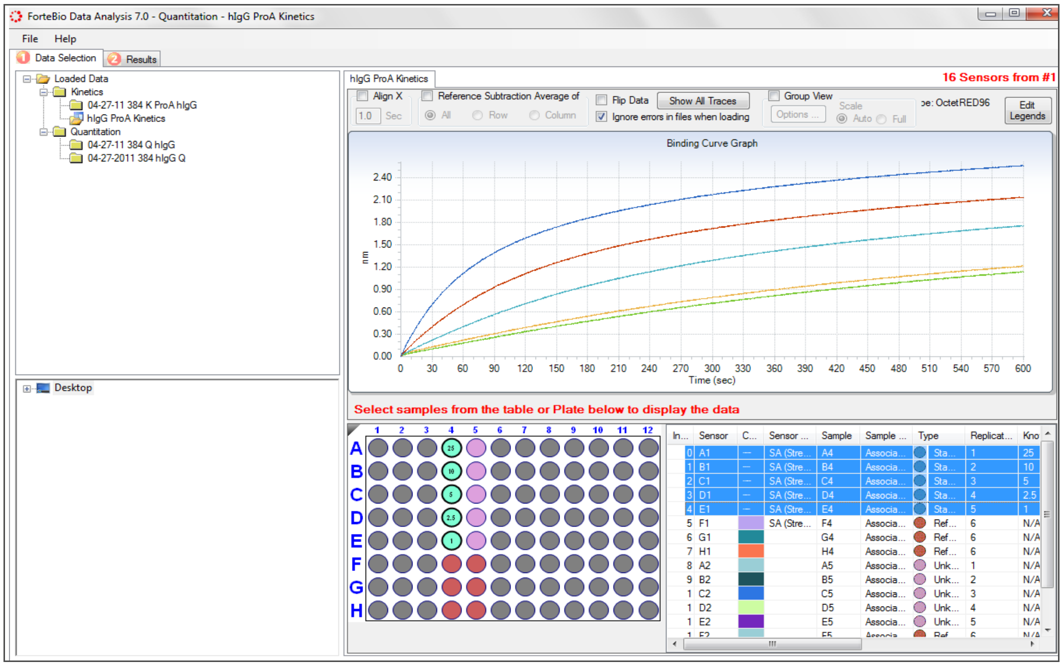

Figure 5-16: *Results Window with Calculated Binding Rates*

NOTE: *To return to kinetics analysis mode, reload the kinetics experiment data (in the Loaded Data directory tree, click the experiment).*

PROCESSING KINETIC DATA

The Processing window provides tools for correcting binding curves using different reference subtraction and alignment options.The data processing steps specify how to reference the data and produce the final binding curves (processed data).

Step 1: Sensor Selection

When a kinetics experiment is opened for the first time, the sensor tray map depicts all active biosensors as *ligand biosensors* (biosensors with immobilized ligand). If the experiment included *reference biosensors* (biosensors without immobilized ligand), you must specify their location in the sensor tray map. If the experiment included *reference buffer wells* (to correct for system drift), you must specify their location in the sample plate map.

Working with the Sample Tray Map and Sensor Tray Map

To use the sample tray map and the sensor trap map (Figure 5-17):

1. Hover the cursor over a biosensor or sample to display a tooltip with information about the item.
2. Click a biosensor or sample to highlight the associated row in the corresponding table at the bottom of the window.
3. Optional. Copy the sensor tray map or sample plate map to the system clipboard, right-click the map and select **Copy to Clipboard**.

   The clipboard contents can be saved as a graphic file for drawing applications.

Selecting Biosensors

To select biosensors:

1. In the **Step 1: Data Selection** pane, select the **Sensor Selection** option (Figure 5-17).

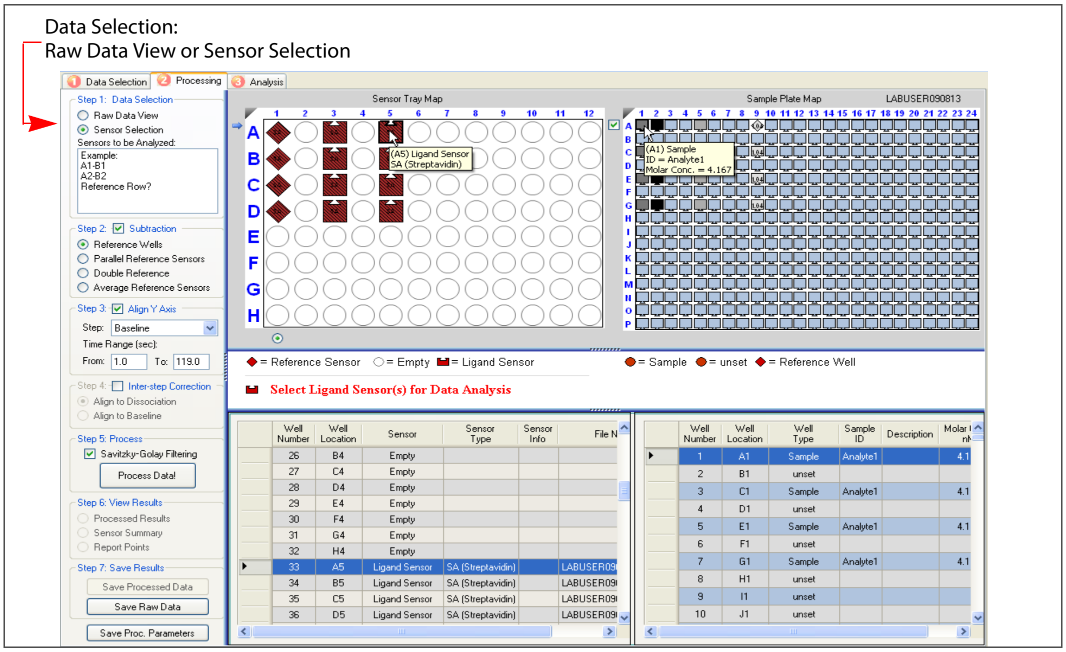

Figure 5-17: Selecting Biosensors in the Processing Window

2. Specify reference biosensors:
   a. In the sensor trap map, select the appropriate biosensors.
   b. Right-click and select **Change Sensor Type** > **Reference Sensor** (Figure 5-18).

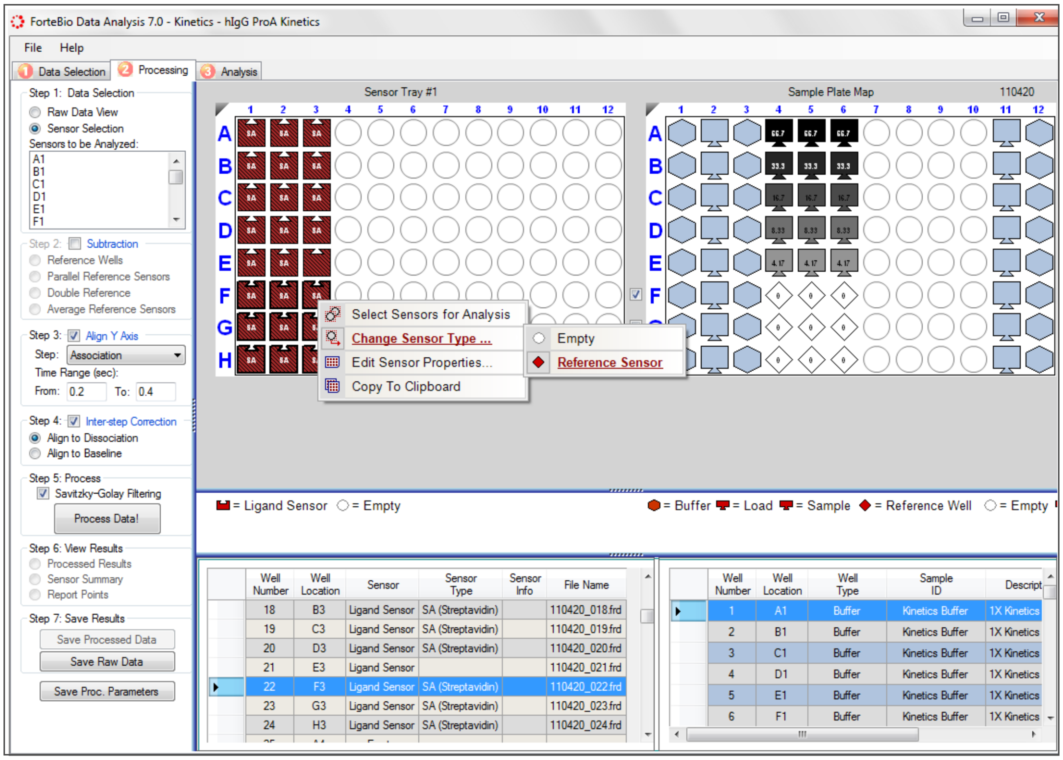

Figure 5-18: *Changing the Biosensor Type*

NOTE: *The biosensor designations can also be edited in the sensor tray table (Figure 5-17: on page 96).*

3. Specify reference wells:
   a. In the sample plate map, select the appropriate wells.
   b. Right-click and select **Change Well Type** > **Reference Well** (Figure 5-19).

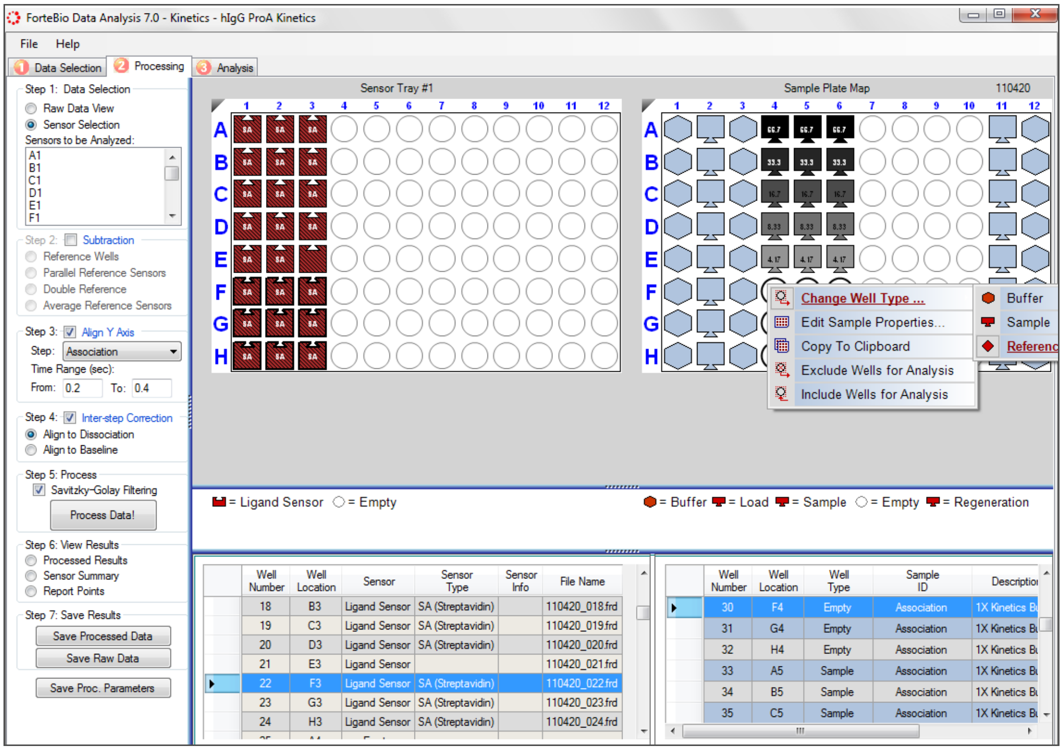

Figure 5-19: Specifying Reference Wells

4. Exclude wells from analysis:
   a. In the sample plate map, select the wells.
   b. Right-click and select **Exclude Wells for Analysis** (Figure 5-19).

Step 2: Reference Subtraction

Reference subtraction is optional and is not required for all applications. There are two types of references used in Octet experiments: reference biosensors and reference wells.

- **Reference biosensors**—Used as a references throughout the entire assay; for example, biosensors without active capture molecules.
- **Reference wells**—Contain only assay buffer, and are used to measure system drift.

To apply reference subtraction during data processing:

1. In the **Step 2:** ☐ **Subtraction** pane, select the **Subtraction** method.

   The subtractions that will be executed (based on the subtraction method, biosensor designation and sample plate well designation) are listed in the Sensors to be Analyzed box.

   If the subtraction method is not compatible with the sensor tray map and the sample plate map, a question mark is displayed in the **Sensors to be Analyzed** box.

2. Confirm the biosensor subtraction in the **Sensors to be Analyzed** box.

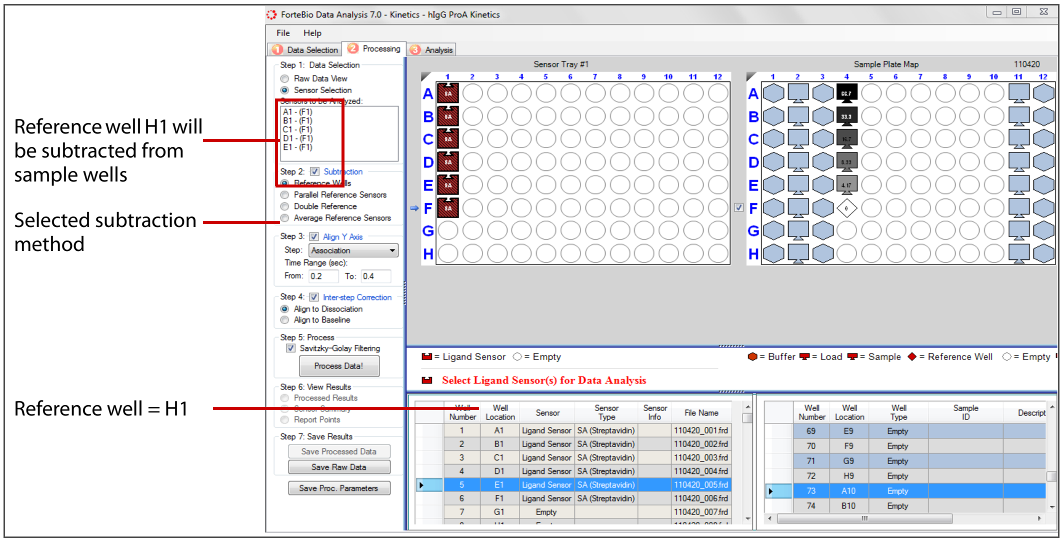

Figure 5-20: *Confirming the Reference Well is Subtracted from the Sample Wells*

If an experiment includes reference biosensors and reference wells, the Octet System Data Acquisition software offers multiple reference subtraction methods for data processing:

- **Reference Wells**—Corrects binding data for system drift. For example, drift is measured by the interaction of the immobilized biosensors with the assay buffer. This method requires at least one row of reference wells in the sample plate. If more than one row of reference wells is selected (checked), the signals are averaged and the average signal is subtracted from the samples.

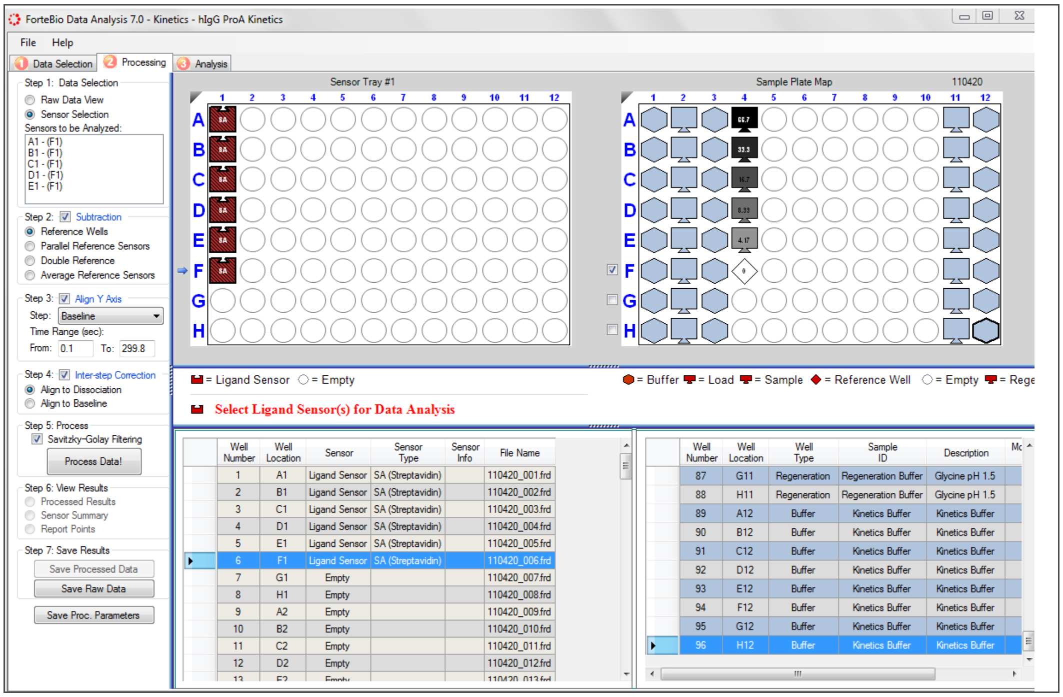

Figure 5-21: Reference Wells—Subtraction Method where Biosensor H1 is the Reference

- **Parallel Reference Sensors**—Corrects data for system artifacts or non-specific binding of the sample to the biosensor surface. This method requires one reference biosensor for each ligand biosensor.

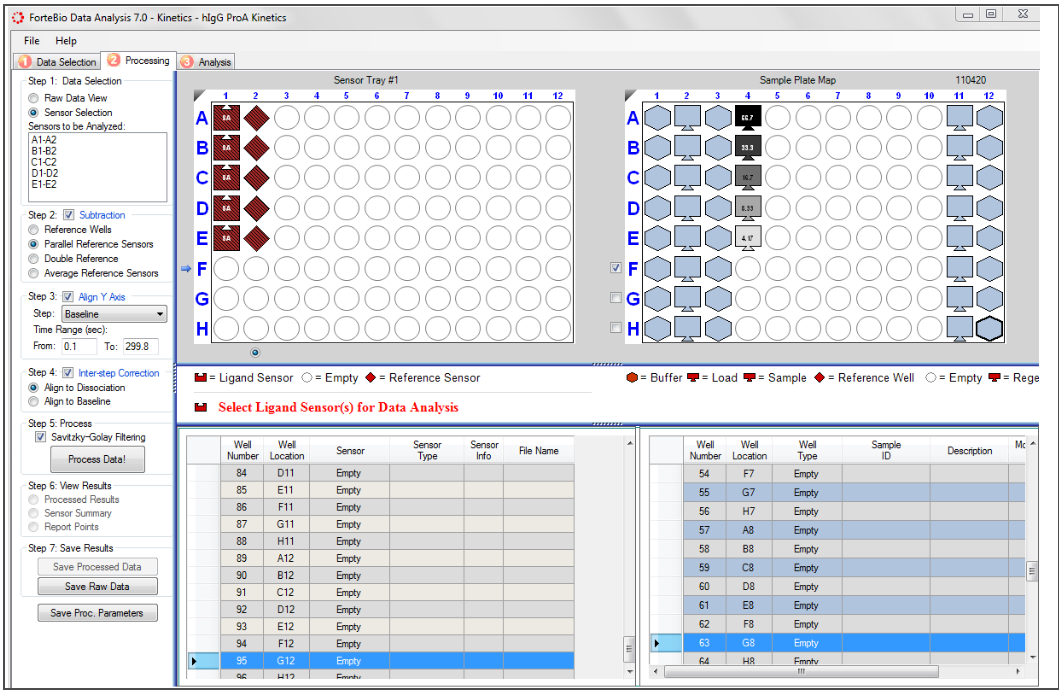

Figure 5-22: Parallel Reference Sensor—Reference Subtraction Method

- **Double Reference**—Corrects the binding data for signal due to system artifacts, non-specific binding, and system drift. This method requires one reference biosensor per ligand biosensor and one or more rows of reference buffer in the sample plate.

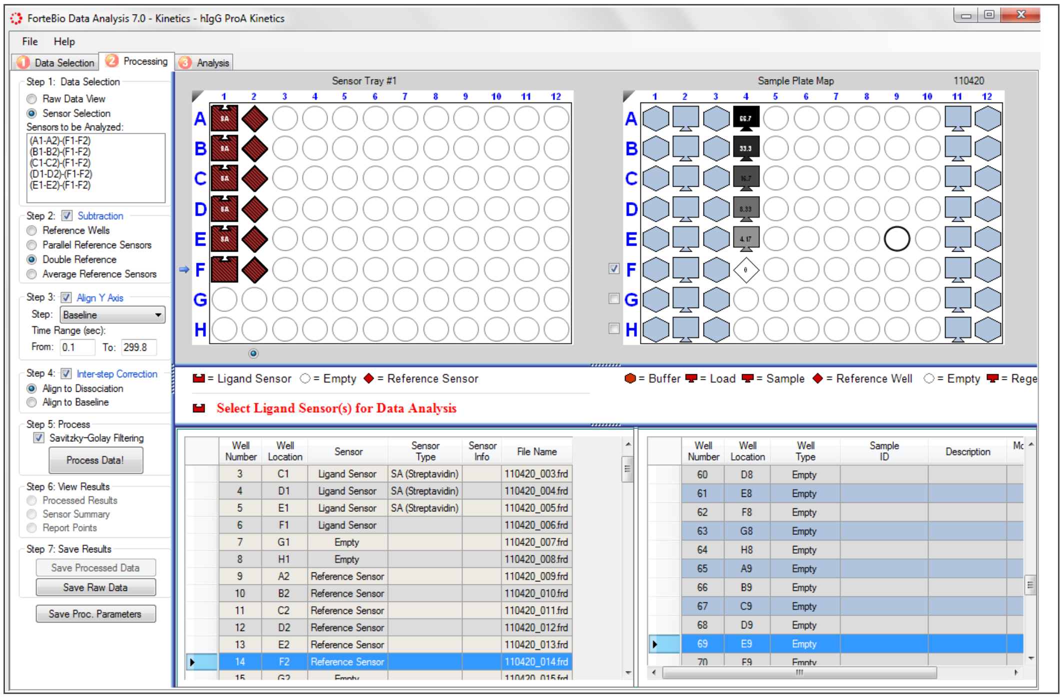

Figure 5-23: *Double Reference Subtraction Method*

NOTE: *In Figure 5-23, A1–A3=Target biosensor A1 corrected by reference biosensor A3, both probing reference wells B1–B3=Target biosensor B1 corrected by reference biosensor B3 for biosensor and microplate well artifacts, both probing positive sample. (B1–B3)–(A1–A3)=Double reference subtraction fully corrects for biosensor and microplate well artifacts and the effect of sample media.*

- **Average Reference Sensors**—Corrects the binding data using either a single reference biosensor or the average signal of multiple biosensors.

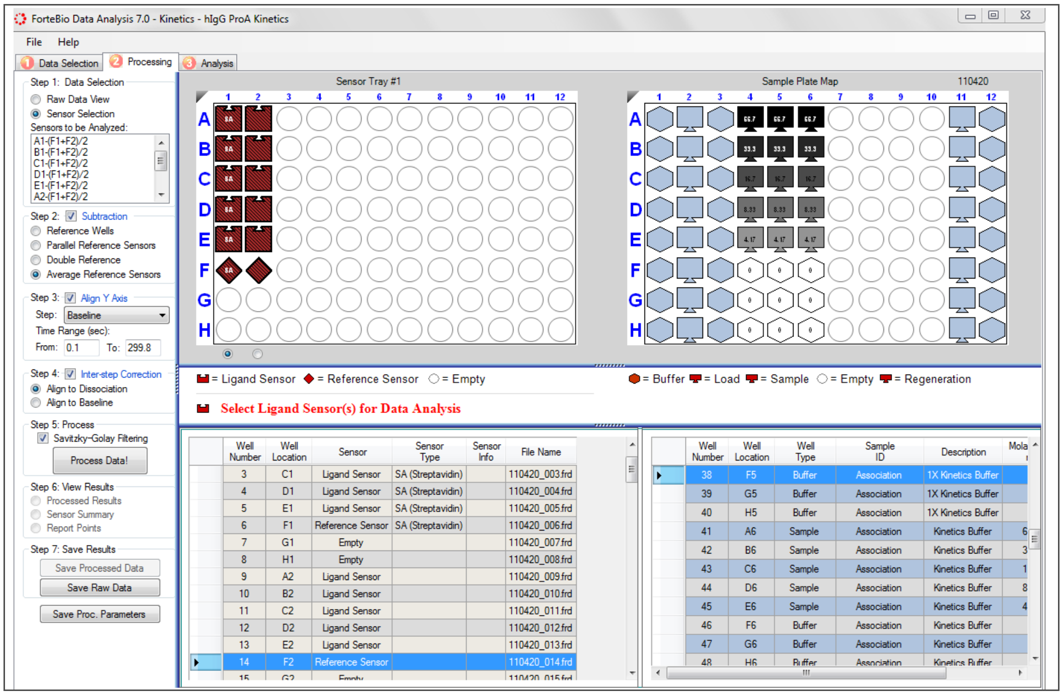

Figure 5-24: Average Reference Sensors Subtraction Method

***NOTE:** In Figure 5-24, AB1–(A1+A2+A3+A4)/4 = Target biosensor B1 corrected for sample medium interference by the average of all reference biosensors.*

Step 3: Align Y Axis

In order to fit curves correctly, they must be aligned to a common reference point upon both the X and Y axes:

- Alignment along the X axis is achieved during assay due to the parallel movement of all biosensors.
- Alignment along the Y axis is achieved using **Align Y Axis** by specifying both a step and time with which to execute the alignment.

The time range from the specified step will be used to calculate an average and that average will then be set to y=0. For example, for alignment to the baseline, select **baseline** and specify the time within the baseline step to set to an average y = 0.

To align to association:

1. Select the **Align Y Axis** option and make a selection from the **Step** drop-down list.
2. Confirm the time range defaults or enter new start and finish times. If you choose to align to the association step, set the shortest time range possible at the beginning of the association step to align Y=0.

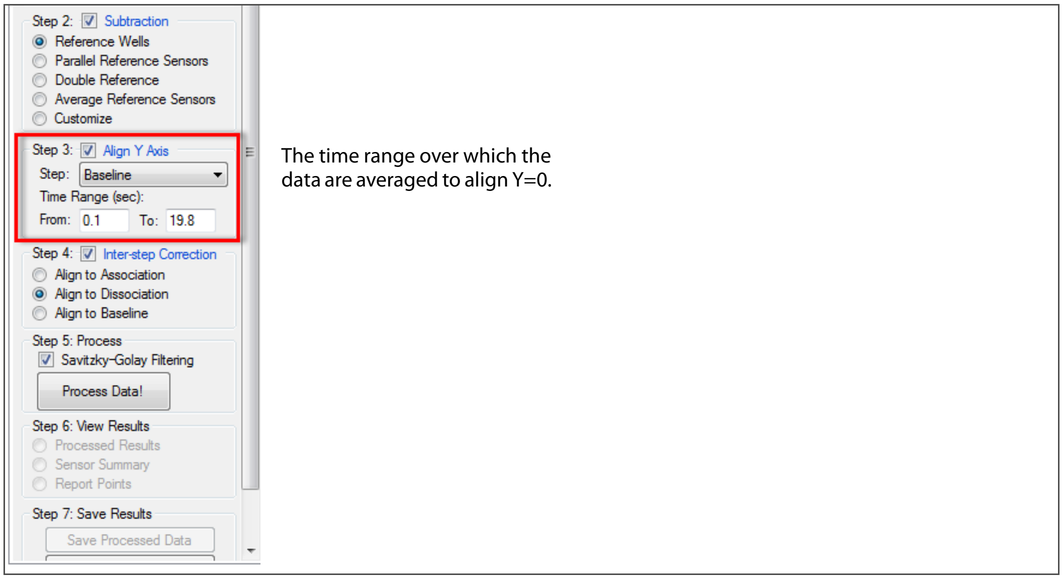

***Figure 5-25:** Aligning the Y Axis*

***NOTE:** The time window should be minimized to the beginning of the association. The data within this window is set to an average of zero and cannot be included in the final curve fit.*

Step 4: Interstep Correction

The interstep correction feature corrects misalignment between two steps due to system artifacts.

***IMPORTANT:** For the most effective interstep correction, the baseline and dissociation steps of an assay cycle must be performed in the same microplate well.*

- **Align to Association**—Moves the baseline step on the Y axis to align the end of the baseline with the beginning of the adjacent association step.
- **Align to Dissociation**—Moves the association step on the Y axis to align the end of the association step with the beginning of the adjacent dissociation step.
- **Align to Baseline**—Moves the association step on the Y axis to align the beginning of the association step with the end of the adjacent baseline step.

***NOTE:** Interstep correction is not recommended for very fast kinetics because some kinetic information may be lost.*

Step 5: Process

Savitzky-Golay filtering removes high-frequency noise from the data. Its use is optional, but is recommended unless the data being analyzed has less than 20 data points in a step.

To process the data (select and confirm the biosensors to analyze):

1. Apply Savitzky-Golay filtering by clicking the **Savitzky-Golay Filtering** check box; see Figure 5-26.

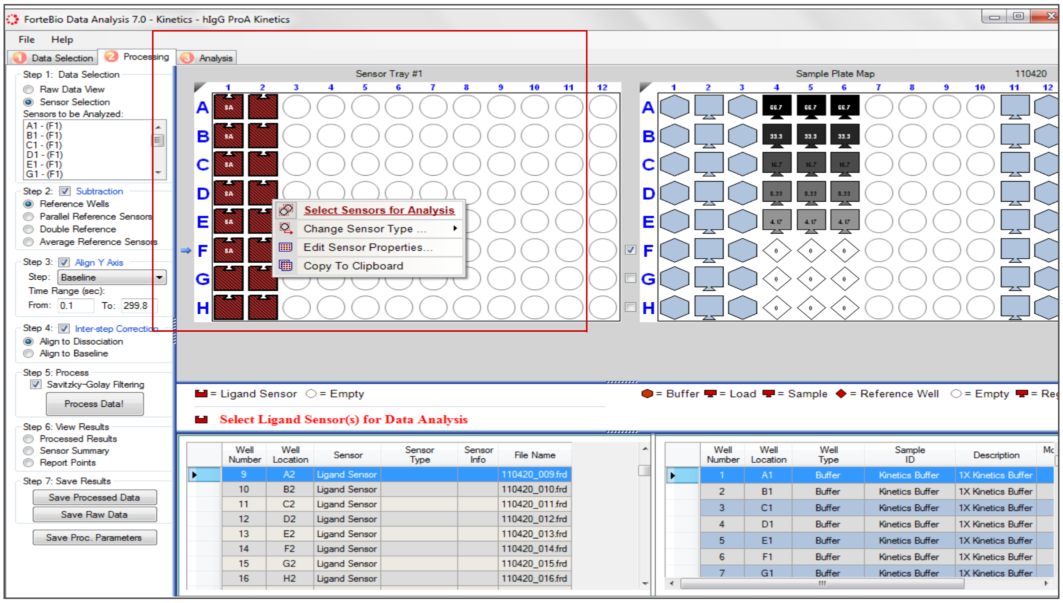

***Figure 5-26:** Selecting and Confirming the Biosensors to Analyze*

2. Click **Process Data!**.

   The processed binding curves are displayed with “Raw”, “Align-Y” and “Align-” windows.

   The processing parameters are automatically saved to the Settings_DataAnalysis.ini or Settings_DataAnalysis.fsd file in the data folder.

***NOTE:** In Octet Data Analysis 21 CFR Part 11 software versions 8.2 and higher, the Settings_DataAnalysis.ini file name extension is Settings_DataAnalysis.fsd. Electronic signatures are also added to data analysis settings (.fsd) files.*

The processing parameters may also be saved by clicking **Save Proc. Parameters**.

These processing settings are displayed the next time the experiment is loaded.

The processed results (Figure 5-27) include:

- **Raw Data**—Binding curves with no reference subtraction.
- **Subtracted Data**—Binding curves after the user-specified reference subtraction method is applied.
- **Align Y**—Binding curves after user-specified Y alignment.
- **Align X**—Binding curve association steps aligned at the same time point.

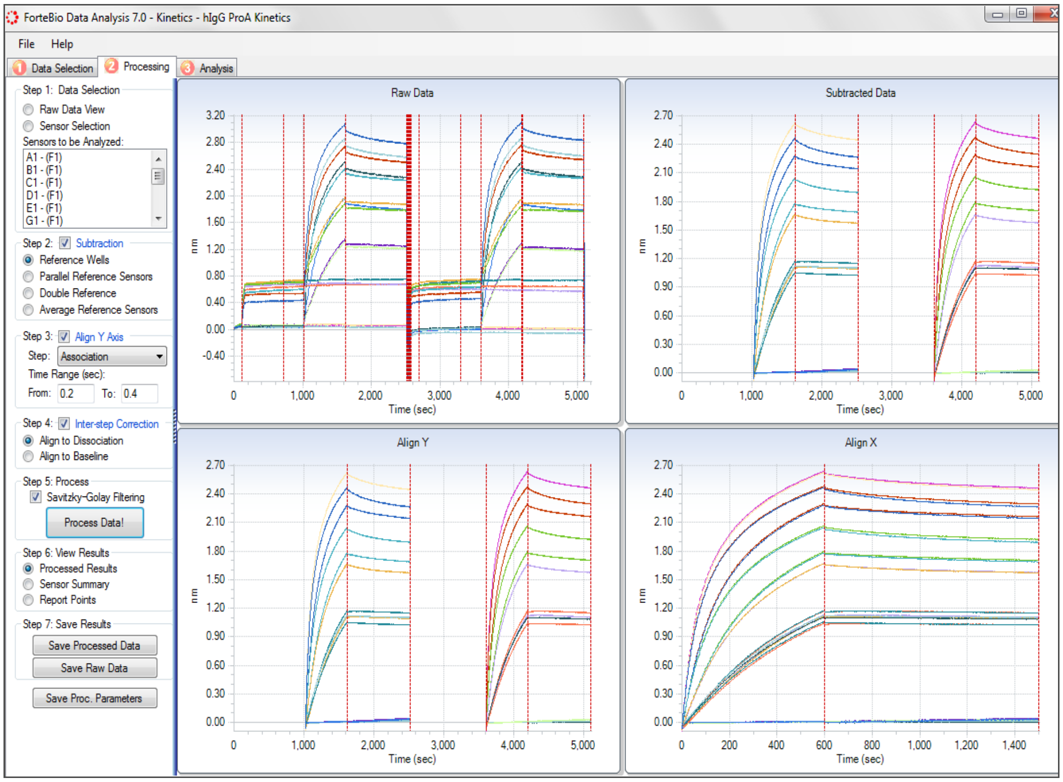

Figure 5-27: Processed Results View

3. Click **Save Proc. Parameters** to save the process settings.

   A Settings_DataAnalysis.ini or Settings_DataAnalysis.fsd file is saved in the experiment folder. These processing settings are displayed the next time the experiment is loaded.

Step 6: Viewing Results

The Octet System Data Acquisition software provides multiple display options (Figure 5-28) for processed data:

- “Processed Results” on page 107
- Sensor Summary
- Report Points

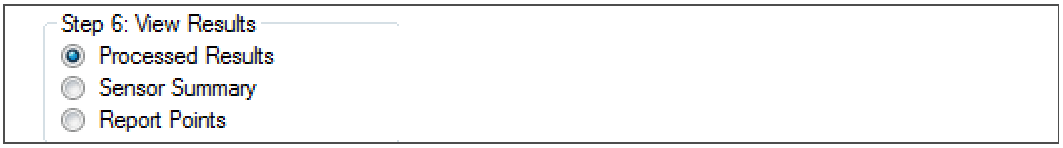

Figure 5-28: *Options for Viewing Results*

Processed Results

- Double-click a graph to display it in a separate window (Figure 5-29).
- Hover the cursor over a curve to highlight the curve and display a tooltip of the time (X axis) and nmshift (Y axis) at that point (Figure 5-29).
- Customize the graph by right-clicking the graph for a shortcut menu of display options (Figure 5-30).
- Customize the curve display, right-click the curve for a shortcut menu of display options (Figure 5-30).

NOTE: *The same display options are also available for binding charts in the Fitting and Residual views in the* ***Analysis*** *tab.*

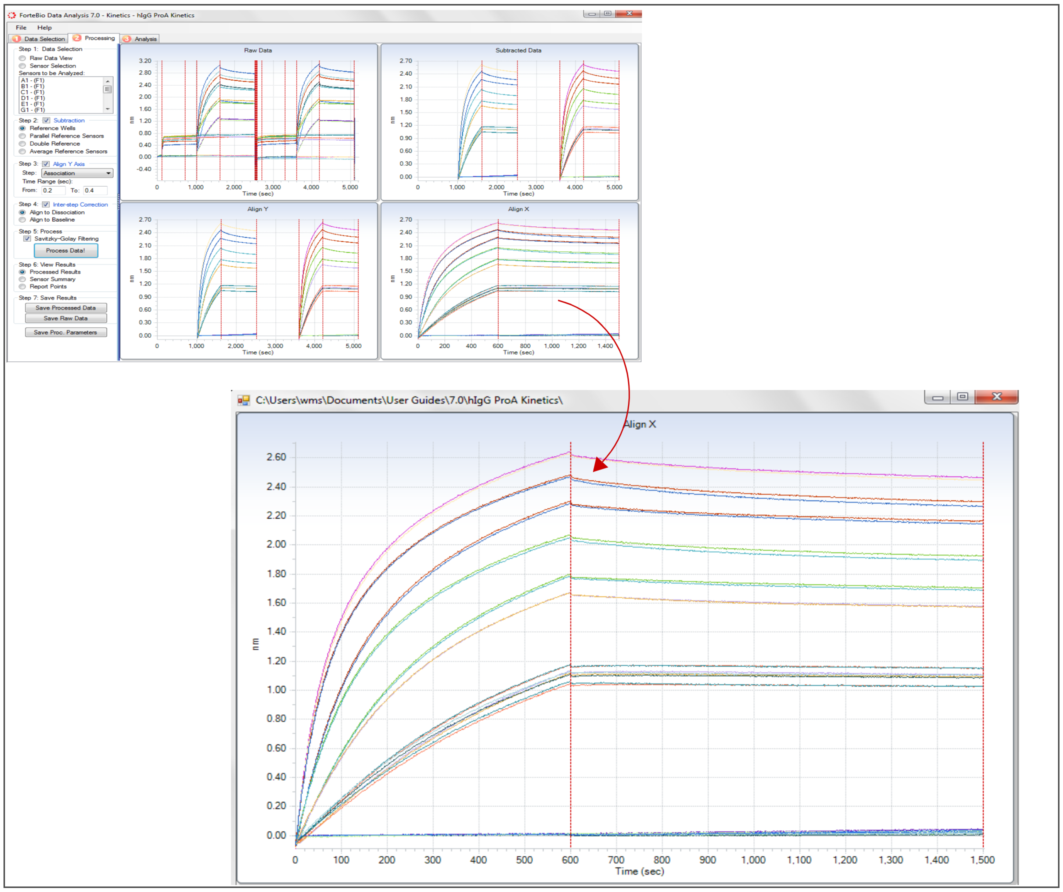

Figure 5-29: *Double-click a Binding Chart to View it in a Separate Window*

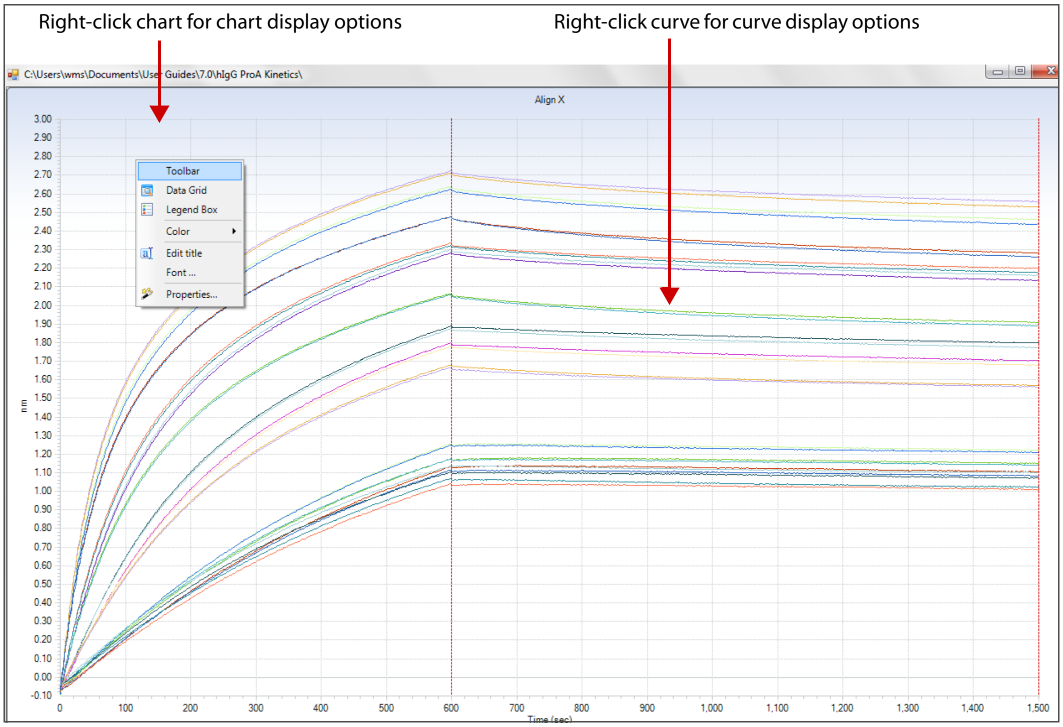

Figure 5-30: *Binding Chart—including Toolbar and Data Grid*

Sensor Summary

The Sensor Summary view displays the binding charts generated during data processing (Figure 5-31).

The Sensor Summary view controls enable you to select the sensor data to display:

- Hover over a binding curve displays a tooltip with sample data.
- Click a sensor tab or one of the arrow buttons in the Sensorgrams controls at the bottom of the window to view data for a specific biosensor.
- Set the number of sensorgrams per row and the number of rows to display.
- Use the Top View check boxes to show or hide the raw data and subtraction graphs in the top row.

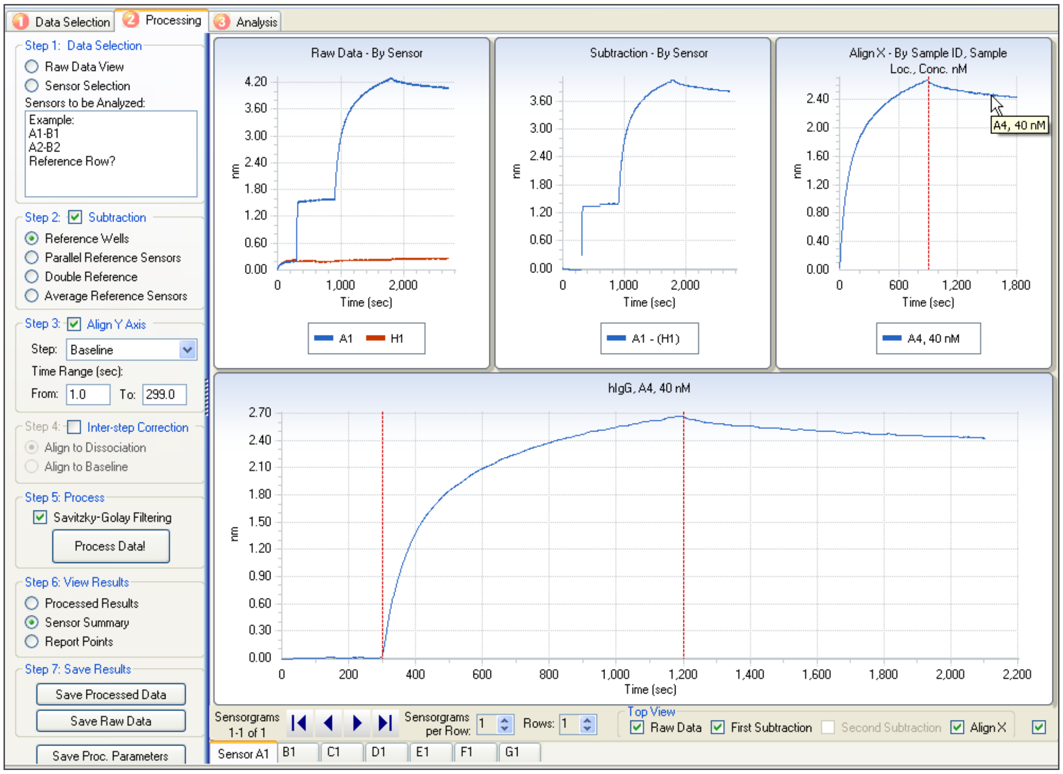

Figure 5-31: *Processed Data in the Sensor Summary View*

NOTE: *In Figure 5-31, the data were processed using the Reference Well sub-traction method.*

Report Points

The Report Points view displays the raw binding data in tabular format (Figure 5-32). Report point analysis can also be performed in the Analysis window; the data will be added to the Report Point Analysis table.

NOTE: *Click a sensor tab at the bottom of the binding chart to view data for a particular biosensor.*

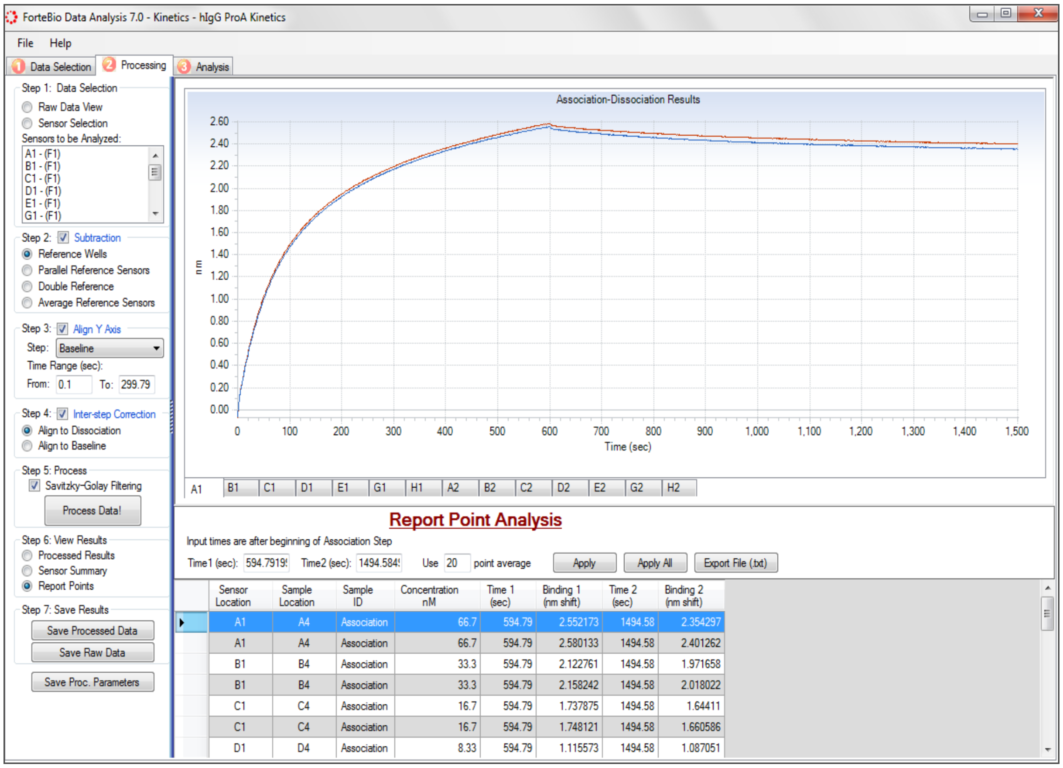

Figure 5-32: Report Points View of Processed Results

Report Point Analysis Features

- **Input times after beginning of Association Step**—The Octet System Data Acquisition software measures the sample binding (nm shift) at Time 1 (within the association step) and Time 2 (within the dissociation step). Both time points refer to time in seconds after the beginning of the association step. You can edit Time 1 or Time 2.
- **Use 20 point average**—Each reported nm shift in the table represents an average of 20 data points centered around the time point specified.
- **Apply**—If you modify Time 1 or Time 2, click **Apply** to re-analyze the sample binding of the active ligand biosensor at the new time points. Report points can also be determined in the Analysis window.
- **Apply All**—If you modify Time 1 or Time 2, click **Apply All** to re-analyze the sample binding of all ligand biosensors at the new time points.
- **Export File (.txt)**—Opens a Save As dialog box so that you can save the Report Point Analysis table.

Results Information

- **Sensor Location**—Ligand biosensor location.
- **Sample Location**—The well location of the sample in the sample plate.
- **Sample ID**—User-specified ligand biosensor information.

- **Concentration (mM)**—Sample concentration.
- **Time 1 (sec)**—Time at which the first binding measurement is acquired.
- **Binding 1 (nm shift)**—The binding signal at Time 1.
- **Time 2 (sec)**—Time at which the second binding measurement is acquired.
- **Binding 2 (nm shift)**—The binding signal at Time 2.

Step 7: Saving Results and/or Processing Parameters

> ***NOTE:*** *In Octet Data Analysis 21 CFR Part 11 software versions 8.2 and higher, a history of changes made to analysis settings are also archived within the experiment folder.*

In this step, you can save the following parameters:

- Raw data
- X,Y data for the curves in the final Processed Results graph to a file format that can be imported to third party applications like Scrubber2 from BioLogic Software.
- Processing parameter settings. The processing parameters are set to the saved values the next time the experiment is loaded.
- Binding chart

To save the processed data:

1. Click **Save Processed Data** (Figure 5-33).
2. In the displayed dialog box, select a destination folder and click **OK**.

   The Octet System Data Acquisition software generates a separate file (.xls) for each biosensor that includes the time, nm shift and sample concentration for each processed curve. This file is suitable for import into third party applications.

To save the raw data:

1. Click **Save Raw Data** (Figure 5-33).
2. In the displayed dialog box, select a destination folder and click **OK**.

   The Octet System Data Acquisition software generates one file (.xls) that includes *time* and *nm* shift data for all of the biosensors.

To save the processing parameters:

1. Click **Save Proc. Parameters** (Figure 5-33).

   The processing parameter values are saved as an **.ini** file in the experiment folder.

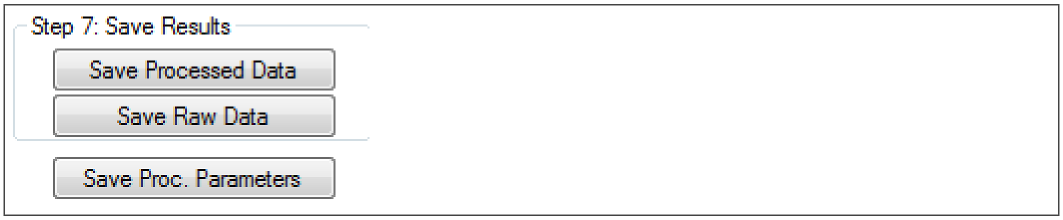

__Figure 5-33:__ Save Results Options

To print or copy a binding chart:

1. Right-click the chart and select **Toolbar** (Figure 5-34) to access the printing and copying buttons (the buttons will appear at the upper left of the binding chart).

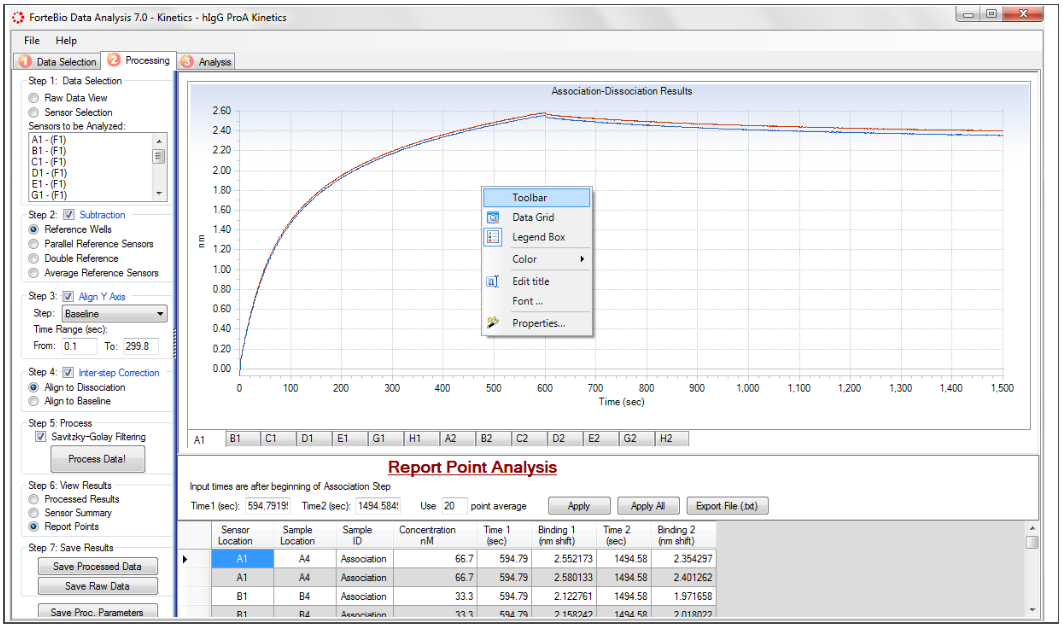

__Figure 5-34:__ Binding Chart Shortcut Menu

2. Select the desired command:

   - 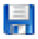 Opens a dialog box that enables you to save the chart in several different formats ((cfx, txt [data only], xml [properties only], bitmap, or metafile).
   - 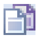 Opens a dialog box that enables you to copy the chart in several different formats (data only, bitmap, or metafile) to the system clipboard.
   - 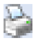 Opens a dialog box that enables you to print the chart.

KINETICS ANALYSIS

In the Analysis window, two types of kinetics analysis are available:

- **Curve fitting**—Determines the kinetic constants k_a, k_d and the affinity constant K_D by fitting the data to a specified binding model.
- **Steady state analysis**—Determines the affinity constant K_D from the calculated or measured equilibrium response.

NOTE: *In Octet Data Analysis 21 CFR Part 11 software versions 8.2 and higher, a history of changes made to analysis settings are also archived within the experiment folder.*

Curve Fitting Analysis

To analyze the processed kinetic data, specify the curve fitting options:

- Steps to analyze
- Curve fitting model
- Type of fitting (local or global) to apply to the data
- Step time to analyze

Curve Fitting Kinetics Analysis Options

- **Steps to Analyze**—Select the step(s) to include in the analysis: association, dissociation, or both.
  - **Association only**—Generates $k_{obs.}$
  - **Dissociation only**—Generates $k_{dis.}$
  - **Association & dissociation**—Generates k_{obs}, k_{on}, k_{dis}, and $K_{D.}$
- **Model**—The mathematical model that is used to generated the fitted view.
  - **1:1 Model**—Fits one analyte in solution binding to one binding site on the surface
  - **2:1 (HL) Model**—Fits the binding of one analyte in solution to two different binding sites on the surface. Kinetic parameters are calculated for two interactions (k_{on1}, k_{on2}, k_{dis1}, k_{dis2}, K_{D1}, K_{D2}). You can assign % K_D1 and K_D2 contributions to interactions in the 1:2 heterogeneous ligand model. For more details see page 116.
  - **1:2 Bivalent Analyte Model**—Fits the binding of one bivalent analyte to a monomeric immobilized ligand. Kinetic parameters are calculated for two interactions (k_{on1}, k_{on2}, k_{dis1}, k_{dis2}, K_{D1}, K_{D2}). The model is available in the **Analysis** tab—**Model** menu—in Kinetics mode.
  - **Mass Transport**—A Heterogeneous Ligand model that fits the binding of the analyte taking into account two steps: 1) transport of the analyte from the bulk solution to the surface, and 2) molecular interaction of the analyte with the ligand.

- **Fitting-Local**— If this option is selected, the Octet System Data Acquisition software computes kinetic constants for each curve. The constants that are calculated depend on the steps that are analyzed (association only, dissociation only, or association and dissociation).
  - **Full**—If this option is selected, the Octet System Data Acquisition software assumes that the off rate eventually reaches the pre-association baseline and forces the curve fit to that point.
  - **Partial**—If this option is selected, the Octet System Data Acquisition software does not assume the dissociation will reach the pre-association baseline.
- **Fitting-Global (Full)**—If this option is chosen, an analysis includes all of the binding curve data in the group and the Octet System Data Acquisition software generates kinetic constants for the entire group. The kinetic constants that are calculated depend on the model selected.
  - **By Sensor**—Groups all data from one biosensor (for example, Biosensor A1) together and applies a global fit to the group.
  - **By Color**—Groups all data that is the same color and applies a global fit to that group. For more details on defining colors by sample attributes, see “Working with the Analysis Results Table” on page 133.
- **Rmax Unlinked option for Global Fitting**—When fitting data, the theoretical *response maximum* (Rmax) can be calculated assuming equivalent surface capacity between biosensors (Rmax linked) or non-equivalent surface capacity between biosensors (Rmax unlinked).
- **Window of Interest (From Start of Step)**
  - **Association**—The time range of the association step data to analyze.
  - **Dissociation**—The time range of the dissociation step data to analyze.
  - **Use Entire Step Times**—Analyzes the entire time duration of the selected step(s).

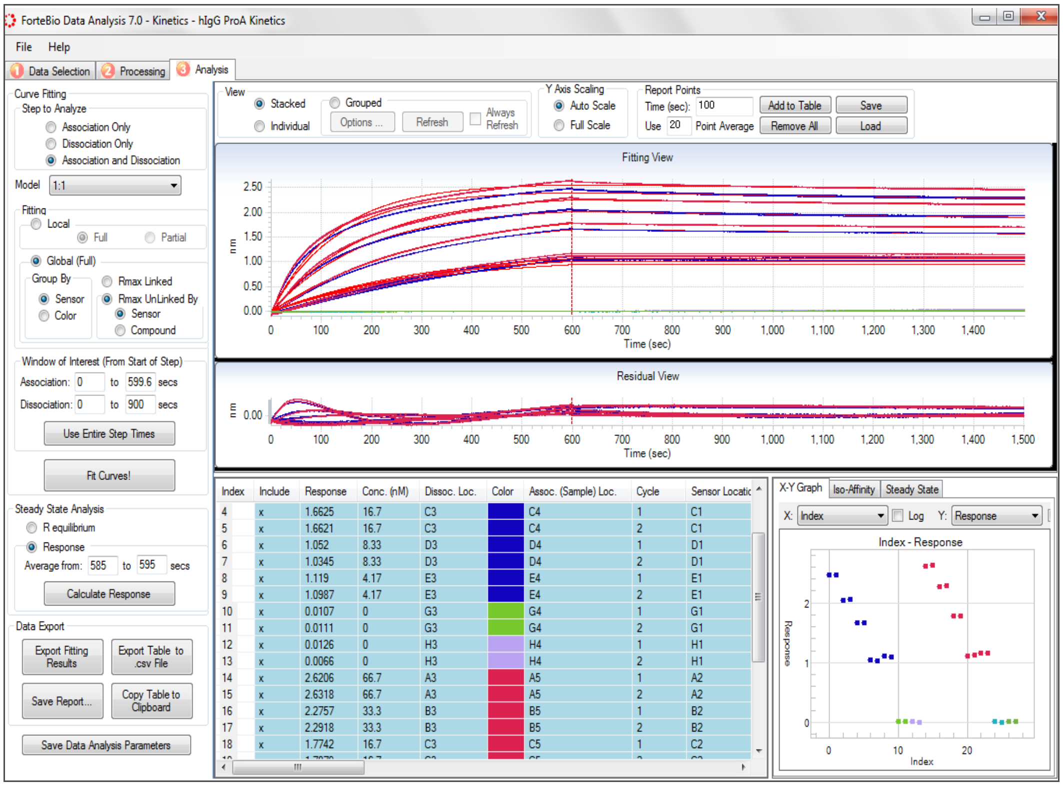

Figure 5-35: *Analysis Window*

Assigning K_D1 and K_D2 % to Interactions in the 2:1 Heterogeneous Ligand Model

To assign K_D1 and K_D2 % contributions to interactions in the 2:1 heterogeneous ligand model (Figure 5-36):

ID	Cycle	Conc. (nM)	Response	KD (M)	KD(%)	KD2	KD2(%)	KD Error	KD2 Error	kon(1/Ms)	kon2	kon Error	kon2 Error	kdis(1/s)
	1	11.2	0.8469	<1.0E-12	64	2.014E-10	36	5.813E-09	1.176E-09	2.877E05	1.100...	4.503E06	3.593E06	<1.0E-07
	1	500	2.2963	<1.0E-12	64	8.730E-09	36	4.938E-10	1.104E-09	2.430E05	1.077...	7.697E03	1.123E05	<1.0E-07
	1	250	2.2932	<1.0E-12	64	1.096E-08	36	<1.0E-12	6.908E-10	3.089E05	1.027...	4.739E03	4.879E04	<1.0E-07
	1	1.39	0.2672	<1.0E-12	58	<1.0E-12	42			4.008E06	5.639...			<1.0E-07
	1	125	2.1024	<1.0E-12	58	2.395E-08	42	<1.0E-12	2.478E-09	6.524E05	3.377...	1.732E04	2.285E04	<1.0E-07
	1	62.5	1.9716	<1.0E-12	57	1.544E-08	43	<1.0E-12	4.459E-09	6.806E05	4.955...	8.540E04	1.364E05	<1.0E-07
	1	31.3	1.701	<1.0E-12	53	8.841E-09	47	6.607E-10	5.567E-09	7.705E05	5.335...	1.833E05	2.946E05	<1.0E-07
	1	2.79	0.3723	<1.0E-12	50	<1.0E-12	50			3.517E06	3.517...			<1.0E-07
	1	22.3	1.2987	<1.0E-12	50	3.816E-09	50	5.466E-09	9.542E-09	5.388E05	5.066...	4.629E05	5.019E05	<1.0E-07
	1	5.57	0.5175	<1.0E-12	50	<1.0E-12	50			1.467E06	1.475...			<1.0E-07
ol	1	0	0.1596											
	1		0.1741											

Figure 5-36: *K_D1 and K_D2 % contributions*

Save the % contribution amount as a separate column in the Data Table in the Analysis tab:

- K_D1%=
- K_D2%=

The interaction that contributes to a higher percentage of the total interaction will be automatically assigned as K_D1.

Excluding Data from Analysis

***NOTE:** In Octet Data Analysis 21 CFR Part 11 software versions 8.2 and higher, whenever you exclude/include wells for analysis, this action is now logged in the Audit Trail.*

To exclude data from the analysis:

1. In the table:

   a. Select the biosensor data (rows) to exclude:

   - To select adjacent rows, hold down the **Shift** key while you click the first and last row in the selection.
   - To select non-adjacent rows, hold down the **Ctrl** key while you click the rows.

   b. Right-click the selected row(s), and select **Exclude Wells** (Figure 5-37) or press the space bar. Press the space bar again to toggle the include/exclude status of the curve.

   Biosensors that will be included in the analysis have an “X” in the **Include** column; excluded biosensors do not (Figure 5-37).

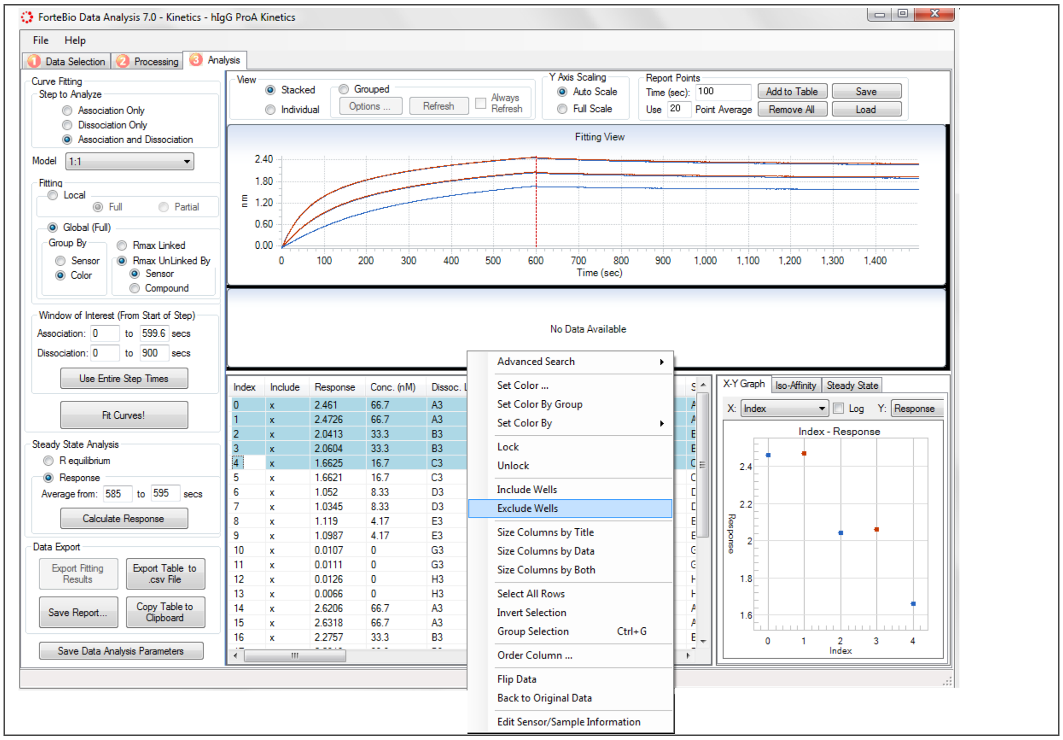

***Figure 5-37:** Excluding Wells from the Biosensor Table*

2. Click **Fit Curves!**

The analysis results are displayed; data from wells marked for exclusion will not be included (Figure 5-38).

Index	Include	Response	Conc. (nM)	Dissoc. Loc.	Color	Assoc. (Sample) Loc.	Cycle	Sensor Location	Sensor Type
0	x	2.4616	66.7	A3		A4	1	A1	SA (Streptavidi
1	x	2.4659	66.7	A3		A4	2	A1	SA (Streptavidi
2	x	2.0419	33.3	B3		B4	1	B1	SA (Streptavidi
3	x	2.0537	33.3	B3		B4	2	B1	SA (Streptavidi
4	x	1.6631	16.7	C3		C4	1	C1	SA (Streptavidi
5	x	1.6554	16.7	C3		C4	2	C1	SA (Streptavidi
6	x	1.0526	8.33	D3		D4	1	D1	SA (Streptavidi
7	x	1.0278	8.33	D3		D4	2	D1	SA (Streptavidi
8	x	1.1196	4.17	E3		E4	1	E1	SA (Streptavidi
9	x	1.092	4.17	E3		E4	2	E1	SA (Streptavidi
10	x	2.6095	66.7	A3		A5	1	A2	SA (Streptavidi
11	x	2.6293	66.7	A3		A5	2	A2	SA (Streptavidi
12	x	2.2646	33.3	B3		B5	1	B2	SA (Streptavidi
13	x	2.2893	33.3	B3		B5	2	B2	SA (Streptavidi
14	x	1.7631	16.7	C3		C5	1	C2	SA (Streptavidi

***Figure 5-38:** Analysis Results Table*

3. Save the settings in the Analysis window: click **Save Data Analysis Parameters** (Figure 5-39).

   A **Settings_DataAnalysis.ini** file is saved in the experiment folder. These settings are displayed the next time the experiment is loaded.

NOTE:** The **Settings_DataAnalysis.ini** file is also automatically saved when you click **Fit Curves!

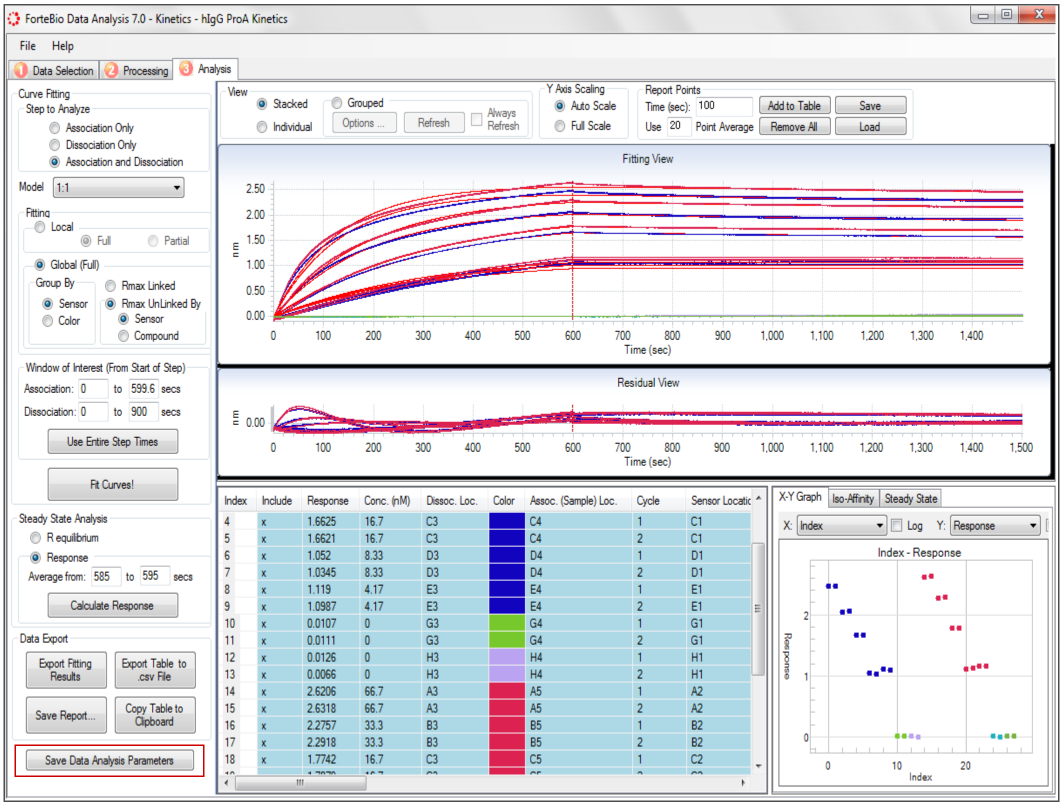

*Figure 5-39: **Save Data Analysis Parameters** Button—Analysis Window*

Steady State Analysis

To analyze the processed kinetic data:

1. Set the analysis options (for more details, see Table 5-1 on page 154).
2. Click **Calculate Response!** to display the analysis results. For more information on viewing results, see “Kinetics Analysis Results” on page 124.

Excluding Data from the Analysis

To exclude specific data from the analysis, remove the check mark next to the row in the analysis results table. Or, right-click the selected row(s) and select **Exclude Wells**.

Steady State Kinetics Analysis Options

- **R equilibrium**—Fits the binding curve to a 1:1 model and uses the calculated Req to determine the steady state affinity. If this option is selected, you first must perform a curve fitting kinetic analysis.
- **Response**—Takes the average response from the user-specified time window and uses it to calculated the steady state affinity.

- **Average from—**The of amount of equilibrium state data to analyze, from the time equilibrium was reached to the time at which the response should be calculated.

PROCESSING BATCH KINETICS ANALYSIS

In batch mode, multiple kinetic data sets may be processed without attended operation. The Octet System Data Acquisition software analyzes experiment data using the data processing parameters in the Settings_DataAnalysis.ini or .fsd file. Batch mode processing may be performed using either a single or multiple .ini or .fsd files.

During batch processing, sample well and sensor information may be substituted with new text, a useful feature if the naming convention requires editing after data acquisition.

- Sample Well information, which includes Well Type, Sample ID, Description and Molar concentration, can be replaced during batch processing by specifying a **Settings_WellInfo.xml** file during batch processing.
- Sensor information as well as some sample information, which includes Sensor Type, Sensor Info, Sample ID and Molar Concentration can be replaced by specifying a **Settings_TableInfo.xml** file during batch processing.
- If both **Settings_WellInfo.xml** and **Settings_TableInfo.xml** are specified, then the Sample ID and Molar Concentration values from **Settings_TableInfo.xml** are used during batch processing while the corresponding information from **Settings_TableInfo.xml** is ignored.

Creating a Kinetic Settings_DataAnalysis.ini File

NOTE: *In Octet Data Analysis 21 CFR Part 11 software versions 8.2 and higher, the Settings_DataAnalysis.ini file name extension is Settings_DataAnalysis.fsd. Electronic signatures are also added to data analysis settings (.fsd) files.*

To create a kinetic Settings_DataAnalysis.ini or Settings_DataAnalysis.fsd file:

1. Load and open a kinetic experiment that will be included in the batch process.
2. Click the **Processing** tab.
3. Enter processing parameters. (For more details see “Processing Kinetic Data” on page 95).
4. Click **Process Data!**.
5. Click the **Analysis** tab.

   This creates a **Settings_DataAnalysis.xml** file in the experiment folder.

NOTE: *To batch process data sets with individual .ini or .fsd files, create an* ***.ini*** *or* ***.fsd*** *file for each experiment in the batch.*

Creating a Kinetic Settings_WellInfo.xml File (Optional)

To create a kinetic **Settings_WellInfo.xml** file:

1. Load and open a kinetic experiment that will be included in the batch process.
2. Click the **Processing** tab and select **Sensor Selection**.
3. In the sample plate map, right-click a well and select **Edit Sample Properties**.
4. Enter the new information for **Well Type**, **Sample ID**, **Description** and **Molar Concentration**, and then close the dialog box.
5. Click **Process Data!** or **Save Proc. Parameters**.

   The **Settings_WellInfo.xml** file is saved to the experiment folder.

Creating a Kinetic Settings_TableInfo.xml File (Optional)

To create a kinetic **Settings_TableInfo.xml** file:

1. Load and open a kinetic experiment that will be included in the batch process.
2. Click the **Processing** tab.
3. Enter processing parameters.
4. Click **Process Data!**
5. Click the **Analysis** tab.

   The **Settings_TableInfo.xml** file is saved to the experiment folder.

NOTE: *If there is an existing* ***Settings_TableInfo.xml*** *file in the experiment folder, it will be overwritten. The original* ***Settings_TableInfo.xml*** *file that contained information entered during data acquisition is deleted.*

6. Right-click the analysis table and select **Edit Sample/Sensor Information**.
7. Enter the new information for **Sensor Type**, **Sensor Info**, **Sample Info** and **Molar Concentration**, and then close the dialog box.

   The **Settings_TableInfo.xml** file is saved to the experiment folder.

Selecting Experiments and Running the Batch Analysis

NOTE: *In Octet Data Analysis 21 CFR Part 11 software versions 8.2 and higher, the Settings_DataAnalysis.ini file name extension is Settings_DataAnalysis.fsd. Electronic signatures are also added to data analysis settings (.fsd) files.*

To select experiments and run the batch analysis:

1. Select **File** > **Kinetic Batch Mode**.

   The Quantitation Batch Mode dialog box displays (Figure 5-40).

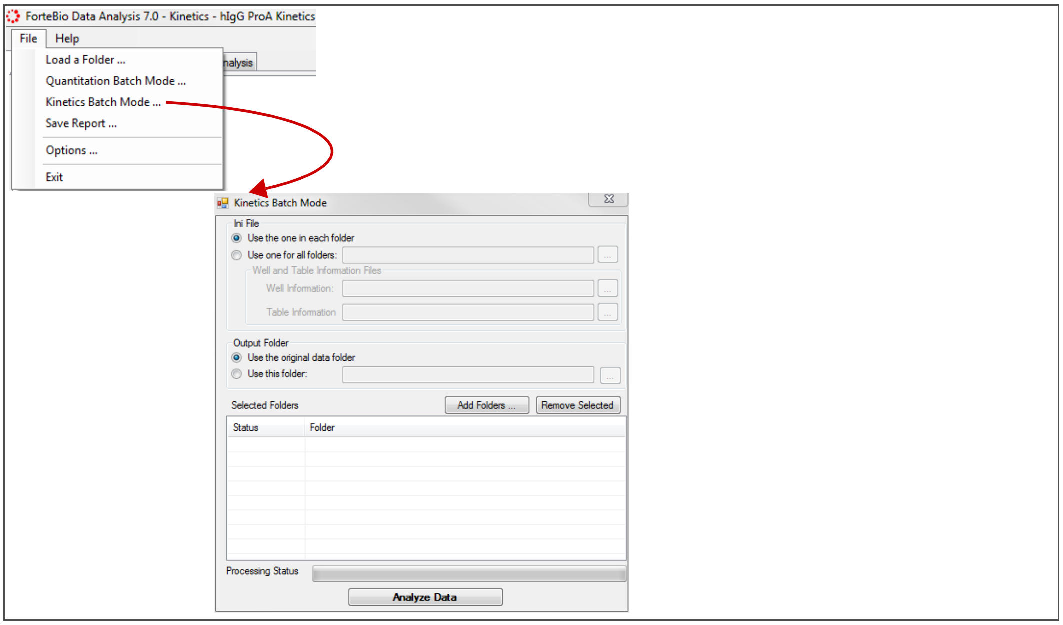

Figure 5-40: *Kinetics Batch Mode—Quantitation Batch Mode Dialog Box*

2. Set the batch mode options:

   - **Use the one in each folder**—Analyzes each experiment using the .ini or .fsd file found in the same experiment folder.
   - **Use one for all folders**—Analyzes all experiments using a single .ini or .fsd file that is selected by the user.
   - **Well Information**—Specifies a **Settings_WellInfo.xml** file in the experiment folder that contains four types of sample information:
     - Well Type
     - Sample ID
     - Description
     - Molar Concentration

This feature can be used to edit sample information after data acquisition. It is useful, for example, if naming conventions (within a project) change over time and the sample information used during data acquisition requires updating.

- Select a Well Information file only if you edited an experiment in data analysis. For more information about editing an experiment, see "Editing Experiments" on page 40. A **Settings_WellInfo.xml** file will be put in the experiment folder of any experiment that has been edited in the Octet System Data Acquisition software.
- Select a well information file if the **Use one for all folders** option is selected.

If no **.xml** file is specified, the well information from the original data acquisition file will be utilized.

- **Table Information**—Specifies a **Settings_TableInfo.xml** file in the experiment folder that contains four types of sensor/sample information:
  - Sensor Type
  - Sensor Info
  - Sample ID
  - Molar Concentration

  This feature can be used to edit sensor/sample information after data acquisition. It is useful, for example, if naming conventions (within a project) change over time and the sample information used during data acquisition requires updating or if the original plate assignment was incorrect. A **Settings_TableInfo.xml** file will be found in the experiment folder of any experiment after the data has been processed (in the **Processing** tab of the Octet System Data Acquisition software) and the **Analysis** tab has been activated. The **Settings_TableInfo.xml** file is updated after closing the Edit Sensor/Sample Information dialog box (of the **Analysis** tab). If no **.xml** file is specified, the well information from the original data acquisition file is used.

3. Select the experiments for batch analysis:
   a. Click **Add Folders**.
   b. In the displayed dialog box, select an experiment folder and click **Add**. Repeat to select each experiment in the batch.
   c. Optional. To remove an experiment(s) from the batch, select the folder(s) and click **Remove Selected**.
4. Click **Analyze Data**.

KINETICS ANALYSIS RESULTS

Kinetics analysis results (Figure 5-41) are presented in graphical and tabular formats. Some viewing options in the Analysis window do not require data fitting (analysis) and are available for processed data.

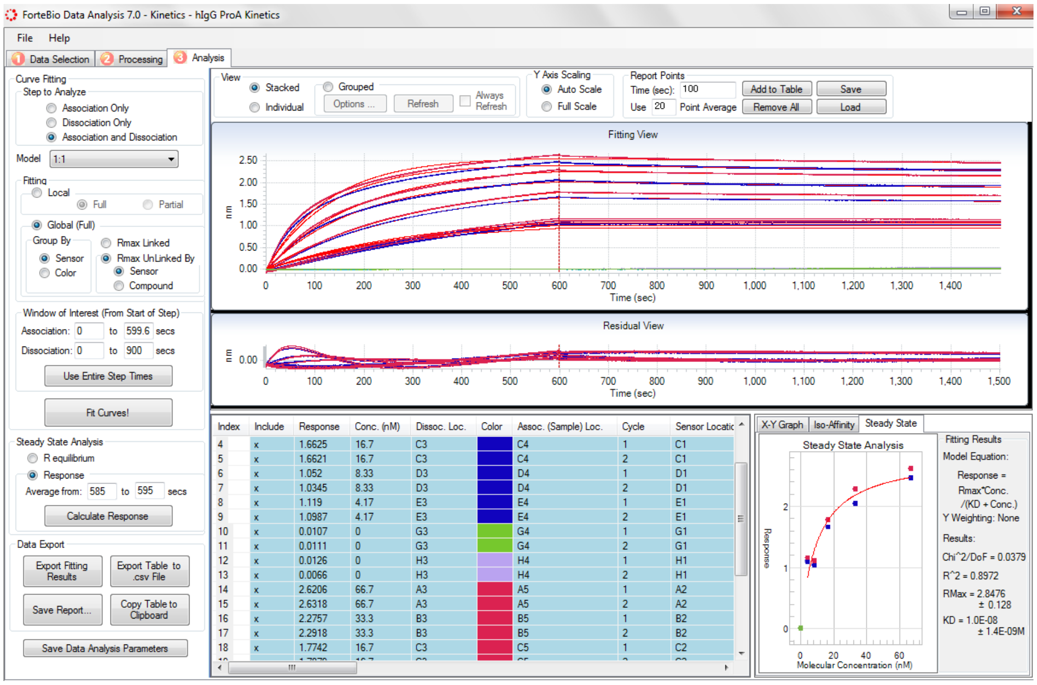

Figure 5-41: Analysis Window with Sample Curve Fitting Results

Fitting View and Residual View

When the analysis is completed, the fitting view displays the processed binding data and the fitted binding curve (red) for all analyzed biosensors (Figure 5-42). The residual view displays the difference between the raw binding data and the fitted curve for all analyzed biosensors. For more details on graph options, see “Working with Graphs” on page 142.

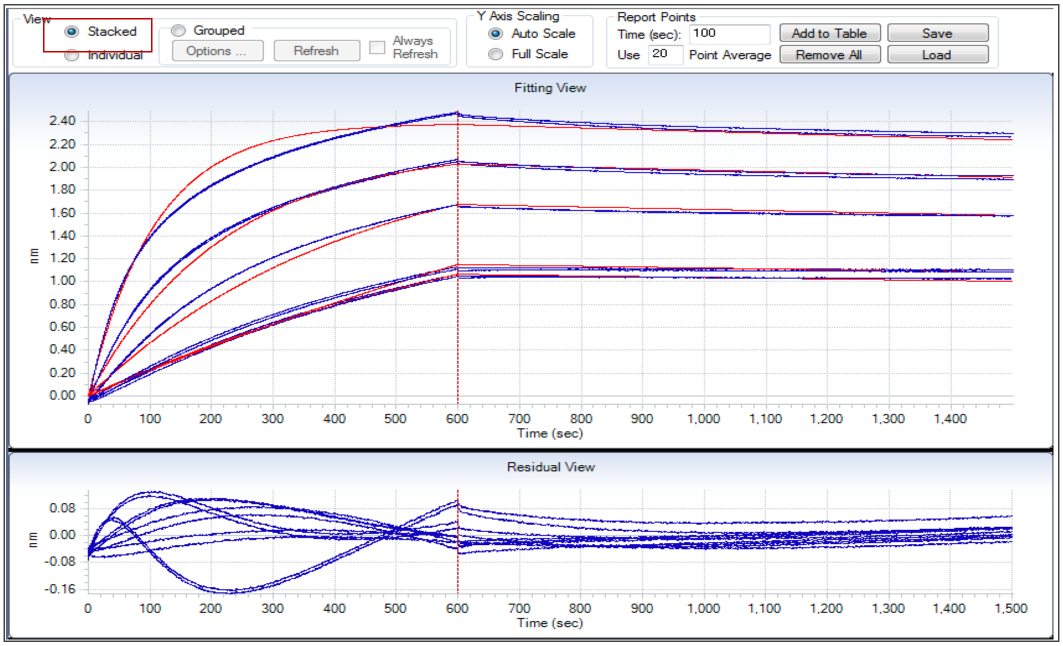

Figure 5-42: Fitting View and Residual View—Stacked Option

NOTE: In Figure 5-42, a local, full fitting analysis was applied to the data. (For detailed information on graph display options, see "Step 6: Viewing Results" on page 106.)

Fitting view options:

- **Stacked**—Displays the binding curves of all ligand biosensors in one graph (Figure 5-42).
- **Individual—**Displays the binding curve from each biosensor in a separate graph (Figure 5-43).
- **Grouped**—Displays graphs organized into groups according to sample attribute or results category. This is a highly useful feature when working with large data sets.
  - **Options**—Click to display the Grouped View Options dialog box.
  - **Refresh**—Updates the graph display.
- **Y Axis Scaling**
  - **Auto Scale**—Scales the y axis to the data in each graph.
  - **Full Scale**—Scales the y axis to in all graphs to the range needed to accommodate all of the data.
- **Report Points**
  - **Time (sec)**—A user-specified time point in the experiment. The Octet System Data Acquisition software computes the response at that time point.

- **Use __ Point Average**—Each data point represents an average of the number of data points specified centered around the time point chosen.
- **Add to Table**—Adds the response computed for the user-specified time point to the analysis results table. Up to 10 response points can be added to the table.
- **Remove All**—Removes all of the response data for user-specified time points from the analysis results table.

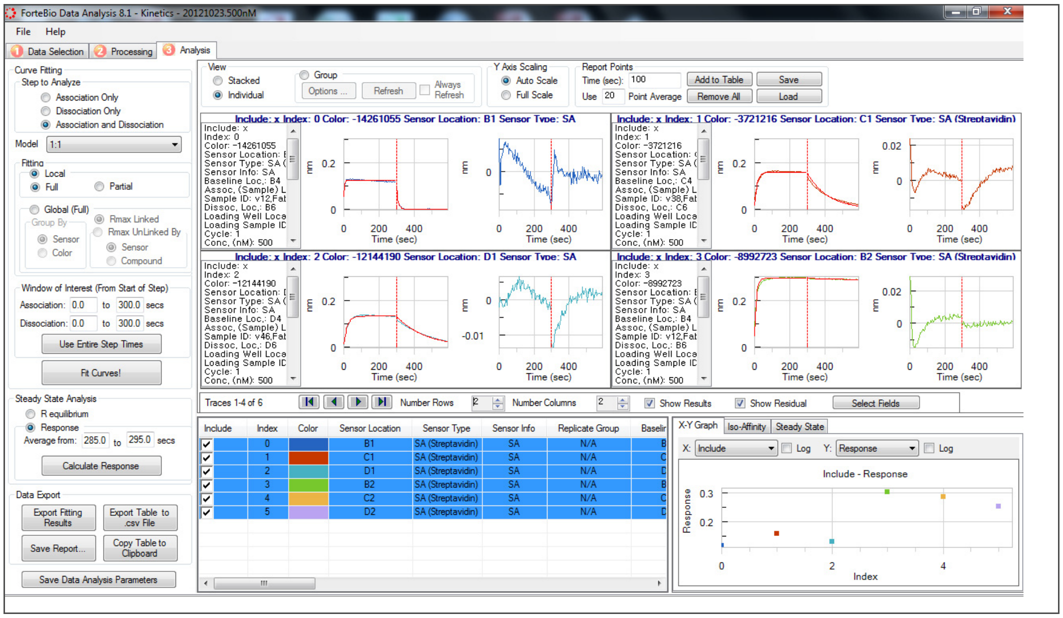

Figure 5-43: Fitting View and Residual View—Individual Option

NOTE: *The data for all biosensors and samples is displayed.*

Individual View

The Individual View option displays the binding curve from each biosensor in a separate graph. Users can modify the display using the options at the bottom of individual view graphs (Figure 5-44).

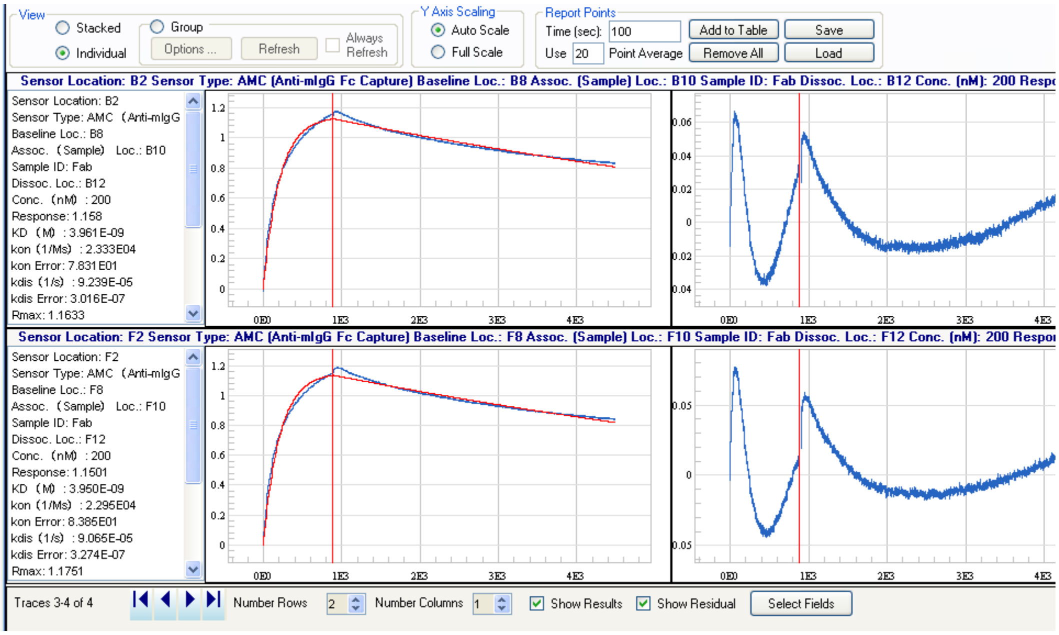

Figure 5-44: *Individual View Display Options*

1. To select the fields to display in the individual view, click **Select Fields** (Figure 5-45).

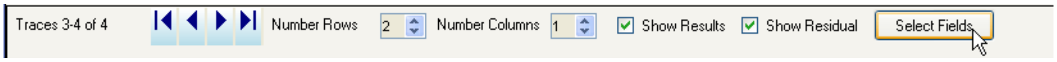

Figure 5-45: *Select Fields Menu Option*

The Choose Fields from Result Table dialog box displays (Figure 5-46).

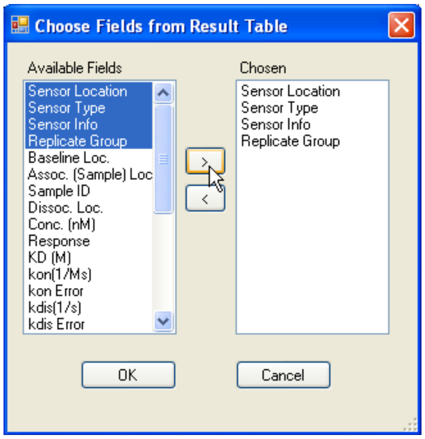

Figure 5-46: *Choose Fields from Result Table*

2. In the Available Fields column, select the desired fields, click > to move the fields to the Chosen column, then click **OK**.

   The information displayed in the individual view will update based on the new selections (Figure 5-47).

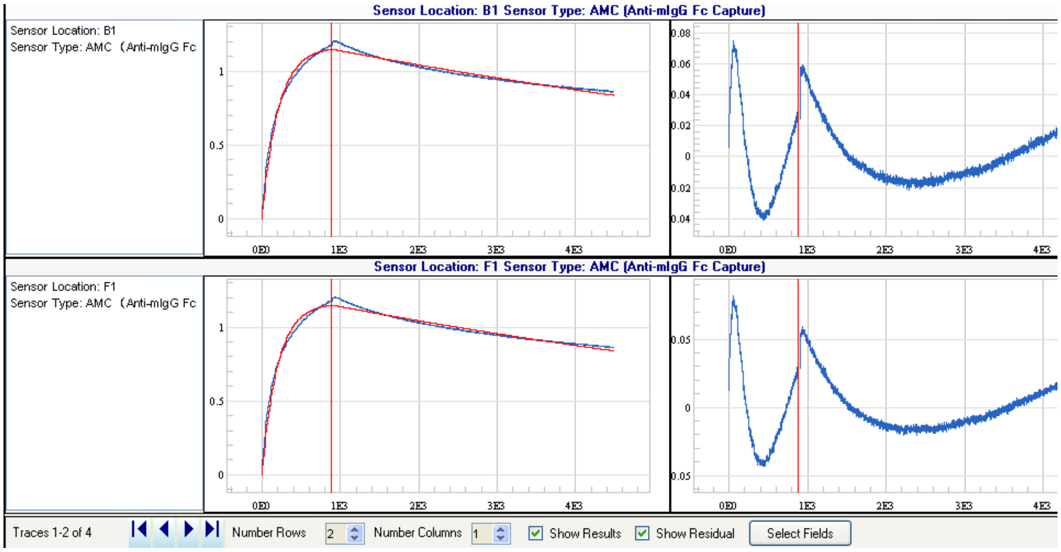

Figure 5-47: *Individual View (Modified)*

Grouping Results for Viewing

To group results for viewing:

1. Select the **Grouped** option and click **Options** to display the Grouped View Options dialog box (Figure 5-48)

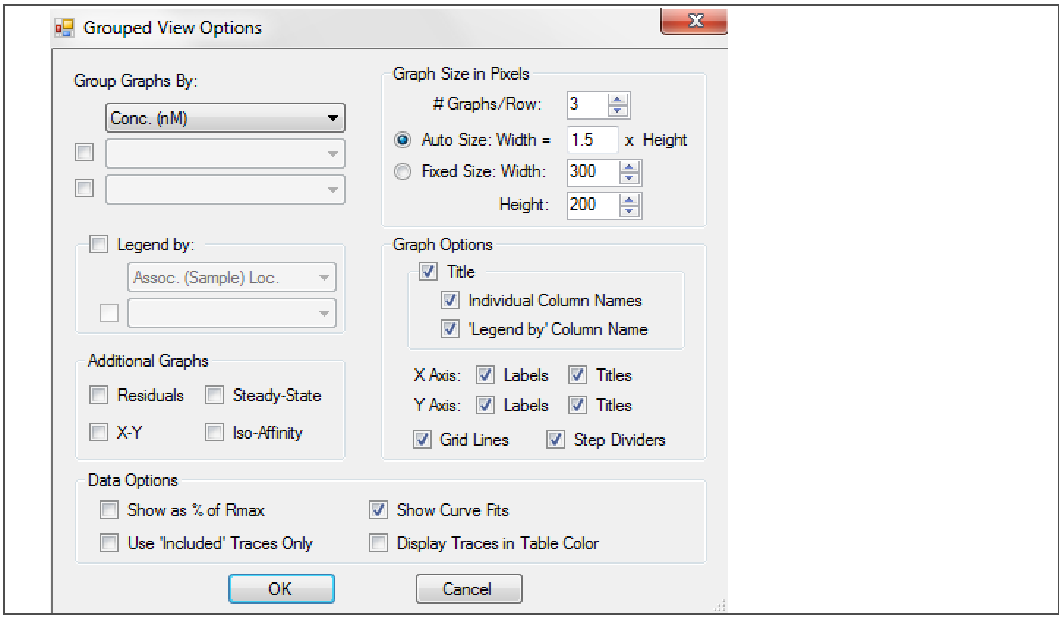

Figure 5-48: *Grouped View Options Dialog Box*

2. From the drop-down lists, select up to three categories for grouping.

   The following Grouped view options are available:

   - **Group Graphs By**—Select up to three categories for grouping the data across three independent parameters.
   - **Legend by**—Select up to two categories to include in the graph legends (Figure 5-49).
   - **Additional Graphs**—Select other graphs to display with the analyzed (fitted) data.
   - **Data Options**—Click the **Use "Included" Traces Only** check box to graph only the biosensors that are included in the analysis (marked with an "X" in the analysis results table.
   - **Graph Size in Pixels**—Options for graph size and the number of graphs to display per row.
   - **Graph Options**—Options for graph labels and other graph display features.

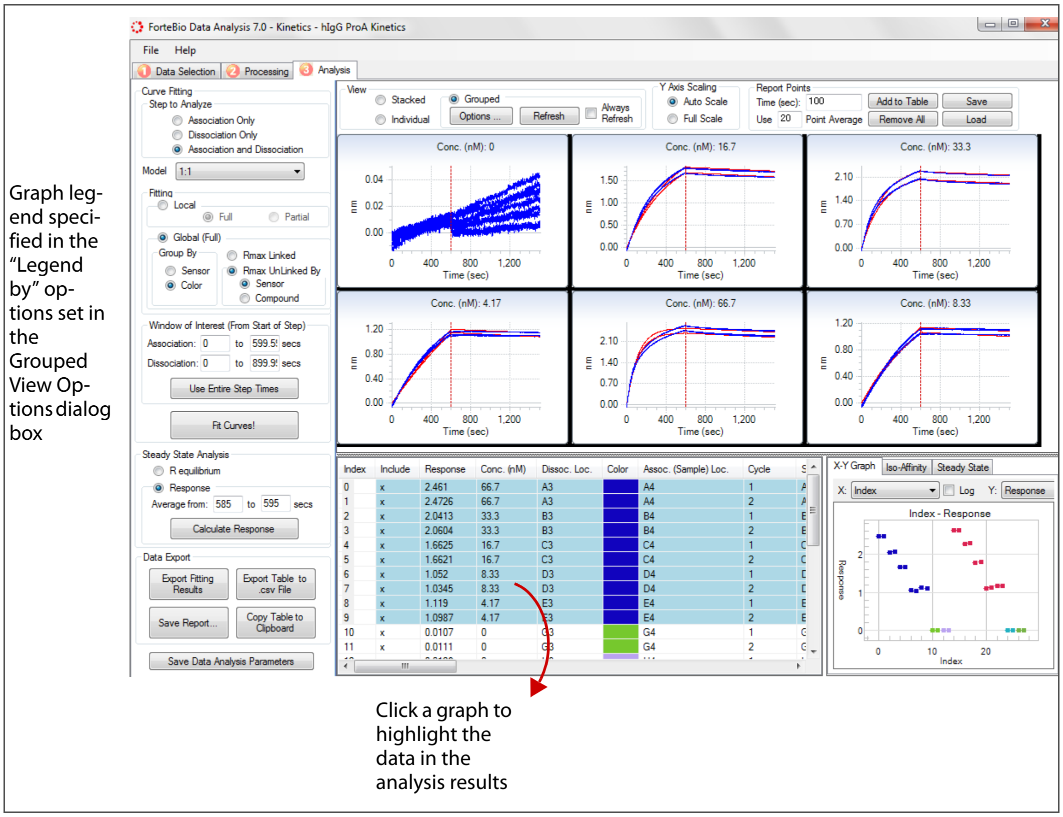

Figure 5-49: *Fitting View—Grouped Option*

Selecting Data for Viewing

The following options are available for selecting data for viewing:

- To view specific biosensor data, select the rows in the analysis results table.
- To select adjacent rows, hold down the **Shift** key while you click the first and last row in the selection.
- To select non-adjacent rows, hold down the **Ctrl** key while you click the rows of interest. The Fitting view, Residual view, and graphs (X-Y, iso-affinity, and steady state) are updated after each data selection.

After a kinetics analysis is completed, the default Fitting and Residual views display the data for all biosensors and samples.

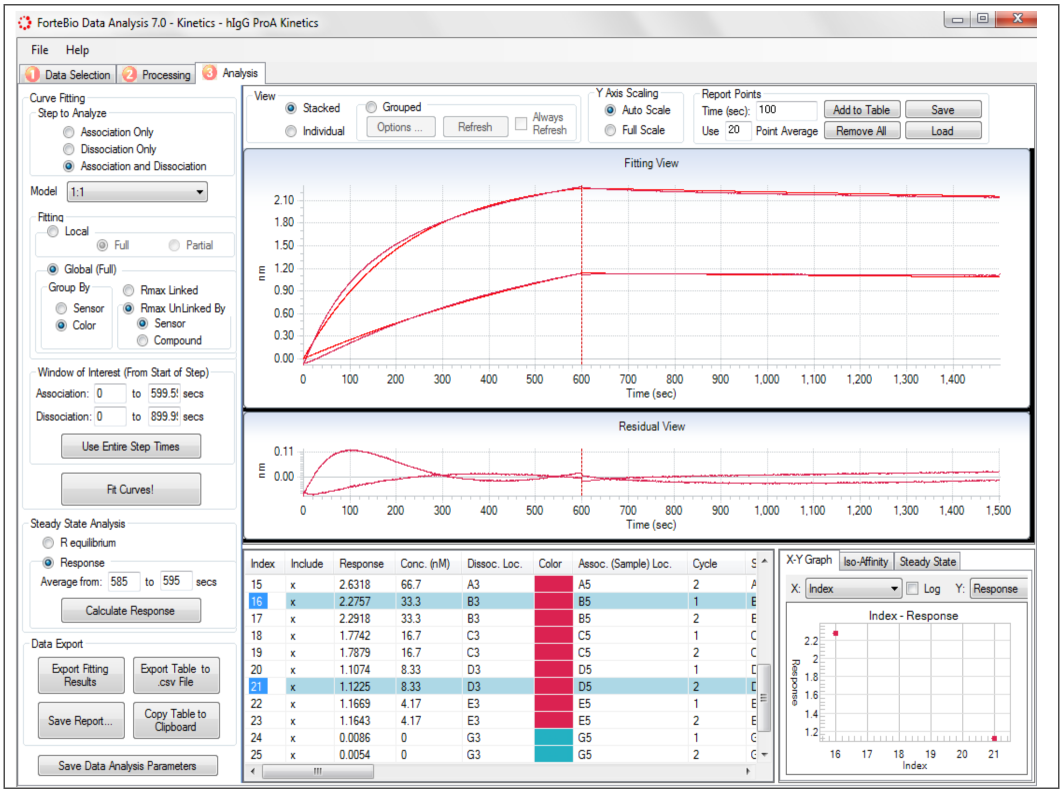

Figure 5-50: Selecting Analysis Results for Viewing in the Fitting View and Residual View

Analysis Results Table

Each row in the Analysis Results table displays the results for one set of association/dissociation data. By default, the Fitting and Residual views include all of the results in the table.

Kinetic analysis results:

- **Index**—Numbered order of the curves processed. The index is useful to sort back to the original order. It is also useful in the graphing applications in the lower right window pane.
- **Include**—"X" indicates data included in the analysis. If this field is blank, the data is not included in the analysis.
- **Color**—The color of the biosensor binding curve in the Fitting and Residual view.
- **Sensor Location**—Location of the biosensor in the sensor tray map.
- **Sensor Type**—The type of biosensor chemistry.
- **Sensor Info**—Information about the biosensor that was entered in the Octet System Data Acquisition software.
- **Baseline Loc.**—Well location in the sample plate or reagent plate (Octet 384 instruments only) in which the baseline was performed.
- **Assoc. (Sample) Loc.**—Sample well location in the sample plate.

- **Sample ID**—The sample ID entered during assay setup.
- **Dissoc. Loc.**— Well location in the sample plate or reagent plate (Octet 384 instruments only) where the dissociation was performed.
- **Conc (nM)**—The molar concentration of the sample used in the association step. The molar concentration is entered by the user or computed by the molarity calculator during experiment setup.
- **Response**—Response calculated from the time window entered in the Steady State Analysis section.
- **KD (M)**—Affinity constant. For the 2:1 and 1:2 models, the Octet System Data Acquisition software computes two K_D values.
- **kon (1/Ms)**—Rate of association. For the 2:1 and 1:2 models, the Octet System Data Acquisition software computes two k_{on} values.
- **kon Error**—Standard error of the rate of association.
- **kdis (1/s)**—Rate of dissociation. For the 2:1 and 1:2 models, the Octet System Data Acquisition software computes two k_{dis} values.
- **kdis Error**—Standard error of the rate of dissociation.
- **Rmax**—The maximum response determined from the fit of the binding data.
- **Rmax Error**—The standard error of Rmax. For the 2:1 and 1:2 models, the Octet System Data Acquisition software computes two Rmax values.
- **kobs (1/s)**—Observed binding rate. For the 2:1 and 1:2 models, the Octet System Data Acquisition software computes two k_{obs} values.
- **km**—The mass transport rate constant.
- **km error**—The standard error of the mass transport rate constant.
- **Req**—The calculated response at equilibrium that is determined from a fit of the binding data.
- **Req/Rmax(%)—**Ratio of Req to Rmax.
- **Full X2**—A measure of the goodness of curve fitting (not directly related to a parameter estimate). It is the sum of squared deviations, where *deviation* is the difference between the actual data point and the fitted curve. There is one value for each curve-fit. Values close to zero indicate a good curve fit.
- **Full R2—R2** is the coefficient of determination (COD). It is an estimate of the goodness of the curve fit and is not directly related to the estimate of a specific parameter. Values close to 1.0 indicate a good curve fit.
- **Report point #1–10**—Up to 10 report points can be added to the analysis results table using the Report Points feature in the Analysis window. The column heading of each report point is time value used to generate that report point. For example, if a report point is generated at 100 seconds, the column heading is “X=100”.

- **SSG K_D**—The steady state group K_D value. Use this feature to quickly view the steady state derived K_D values of groups defined within grouped view (not replicate groups). The column is populated by opening Grouped view, selecting up to three grouping parameter and activating **Steady-State** under additional graphs. The SSG K_D value is reported for the set of data within each pane of the Grouped view. Replicate grouping and global analysis are not used to determine this value.
- **SSG Rmax**—The steady state group Rmax value. Use this feature to quickly view the Rmax value of steady state data for a group defined within Grouped view (not replicate groups). The column is populated by opening Grouped view, selecting up to three grouping parameter and activating **Steady-State** under additional graphs. The SSG Rmax value is reported for the set of data within each pane of the Grouped view. Replicate grouping and global analysis are not used to determine this value.
- **SSG R^2**—The steady state group R^2 value. Use this feature to quickly view the R^2 value of steady state data for a group defined within Grouped view (not replicate groups). The column is populated by opening Grouped view, selecting up to three grouping parameter and activating **Steady-State** under additional graphs. The SSG R^2 value is reported for the set of data within each pane of the Grouped view. Replicate grouping and global analysis are not used to determine this value.
- **Loading Well Location**—Location of the sample well used during the load step of the experiment.
- **Cycle**—Number of biosensor regeneration cycles.

Working with the Analysis Results Table

To view a shortcut menu of display options (Figure 5-51), right-click the Analysis Results table.

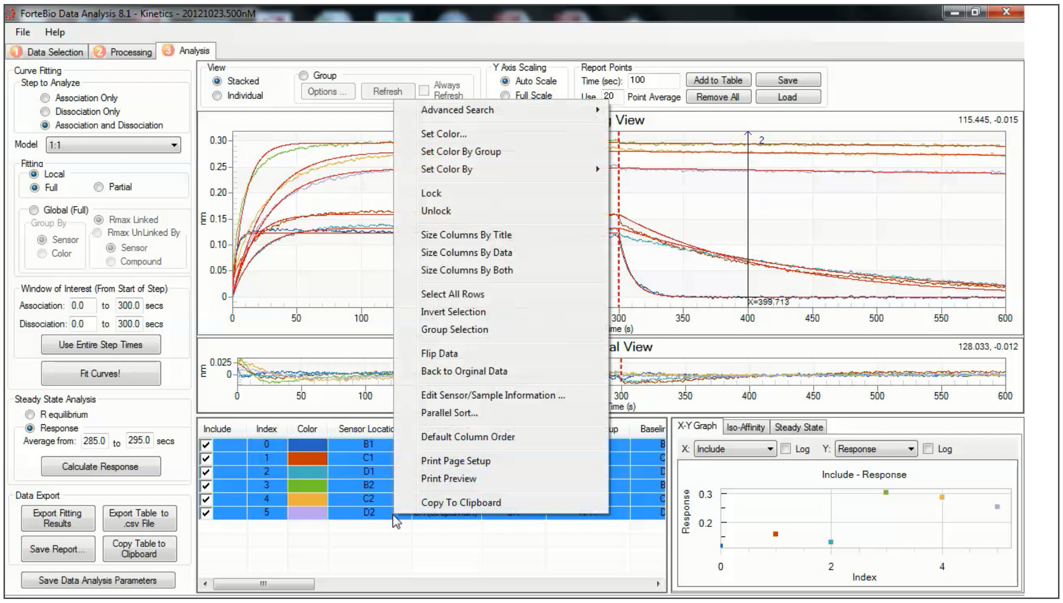

***Figure 5-51:** Analysis Results Table Shortcut Menu*

Analysis results table display options:

- **Advanced Search**—Searches the contents of the results table of the **Analysis** tab. Multiple levels and operators are available. Searches may be combined (using AND/ OR operator) and saved (see Figure 5-52).
- **Set Color**—Opens the color palette that enables you to choose a color for the selected results (see Figure 5-53).
- **Set Color By Group**—Color-codes the results according to the groups set in the Grouped View Options dialog box (see Figure 5-48).
- **Set Color By**—Enables you to color-code results according to a user-selected category from the analysis results table (see Figure 5-54).
- **Include Wells**—Removes the “X” in the Include column for the selected biosensors. Re-run the analysis to include these biosensors in the analysis.
- **Exclude Wells**—Adds an “X” in the Include column for the selected biosensors. Re-run the analysis to exclude these biosensors from the analysis.
- **Size Columns by Title**—Automatically sets the column width to fit the column title.
- **Size Columns by Data**—Automatically sets the column width to fit the data.
- **Select All Rows**—Selects all biosensors in the table and displays the data in the Fitting view and graphs.
- **Invert Selection**—Changes the wells status so that included wells become excluded wells and excluded wells become included wells. You must re-run the analysis to apply the inverted settings.
- **Set Column Order—**Opens a dialog box that enables you to change the order of the table columns.
- **Parallel Sorting—**Allows you to sort results in parallel based on specific sorting parameters.

Searching Analysis Results

The complete contents of the **Analysis** tab—results table is searchable using the **Advanced Search** tool. The search result is a highlighted set of table rows that meet the specified search criteria. The set of rows selected before activation of the Advanced Search dialog box may be combined with the results of the search using **ALL**, **AND**, and **OR** operators specified within the Advanced Search dialog box.

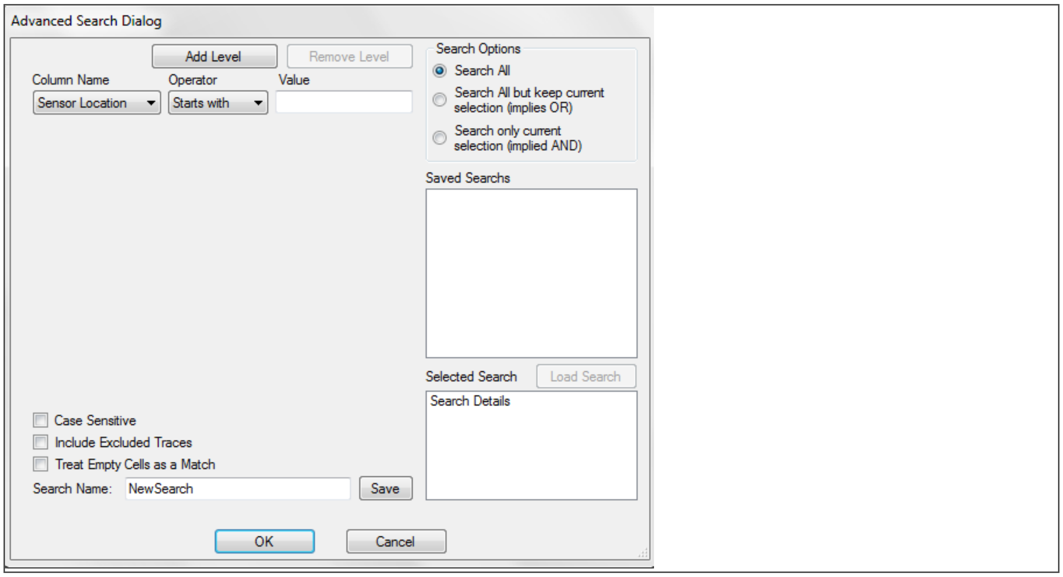

Figure 5-52: *Advanced Search Dialog Box*

Advanced Search options:

- **Column**—The column of the results table searched.
- **Operator**—The method of matching the search term with the searchable text. Options include *Starts with*, *Ends with*, *Contains*, *Does not contain*, and *Is empty*.
- **Value**—The search term for a single level.
- **Add Level**—Adds one additional search level to the search. The **AND** operator applies to all levels
- **Remove Level**—Removes the selected search level.
- **Case Sensitive**—Requires that the case of all characters (uppercase and lowercase) of the search results match the search term.
- **Include Excluded Traces**—Includes all data acquisition traces within the search regardless of exclusion during analysis.
- **Treat Empty Cells as Match**—Returns empty cells of the column as positive matches, useful for searches in which cells were inadvertently left empty during plate assignment or sample annotation.
- **Search Options**
  - **Search All**—Specifies all text within the Analysis table as searchable regardless of the set of rows selected before activation of the Advanced Search dialog. The search return is a highlighted set of rows in the table that match the search the search criteria.
  - **Search all but keep current selection (implies OR)**—Specifies all text in the Analysis table as searchable regardless of the set of rows selected before opening the Advanced Search dialog box.

- **Search only current selection (implies AND)**—Specifies the text as the set of rows selected before opening the Advanced Search dialog box.

Searching Contents of Results Table

To search the contents of the Results table:

1. On the **Analysis** tab, right-click a cell in the Results table, and select **Advanced Search** > **Edit Search**.
2. Using the **Column Name** pull-down menu, select a column to search.
3. Using the **Operator** pull-down menu, assign an operator.
4. Enter the search term under **Value**.
5. If the search is case sensitive, select the **Case Sensitive** option.
6. Optional. Select the **Include Excluded Traces** option to include traces excluded from analysis in the search.
7. Optional. Select the **Treat Empty Cells as a Match** option to include empty cells in the search.
8. Optional. Click **Add Level** and repeat steps 2–4 to add additional levels to the search.

   The set of rows selected in the table (when the Advanced Search dialog box was opened) can be applied to the search using **ALL**, **OR**, and **AND** operators.

   a. Select **Search All** to search the entire table (**ALL** operator).
   b. Select **Search All but keep current selection** to search the entire table with retention of the selection present when the Advanced Search dialog box was opened (**OR** operator).
   c. Select **Search only current selection** to search only the rows selected when the Advanced Search dialog box was opened (**AND** operator).

   The search can be saved by specifying a name under **Search Name** and clicking **Save**. The name of the saved search will appear in the **Saved Searches** list.

9. Click **OK** to execute the search.

   Rows that contain cells meeting the search criteria will be highlighted.

Editing a Search

To edit a search:

1. On the **Analysis** tab, right-click any cell in the Results table, and select **Advanced Search** > **Edit Search**.
2. Select a saved search from the **Saved Searches** list.
3. Click **Load Search**.

   The parameters for the specified search are restored.

4. Edit the search (see steps 2–9 in "Searching Contents of Results Table" on page 136).

The search can be saved by specifying a new name under **Search Name** and clicking **Save**. The name of the saved search will appear in the **Saved Searches** list.

5. Click **OK** to execute the search.

   Rows that contain cells meeting the search criteria will be highlighted.

Color-Coding Data

You can assign different colors to the binding curves as a follows:

- A particular color to user-selected results. This is useful when grouping for a global fit.
- Color according to a results category.

Color-Coding User-Selected Results

To color-code user-selected results:

1. Select one or more rows in the analysis table (click the row header).
2. Right-click and select **Set Color**.

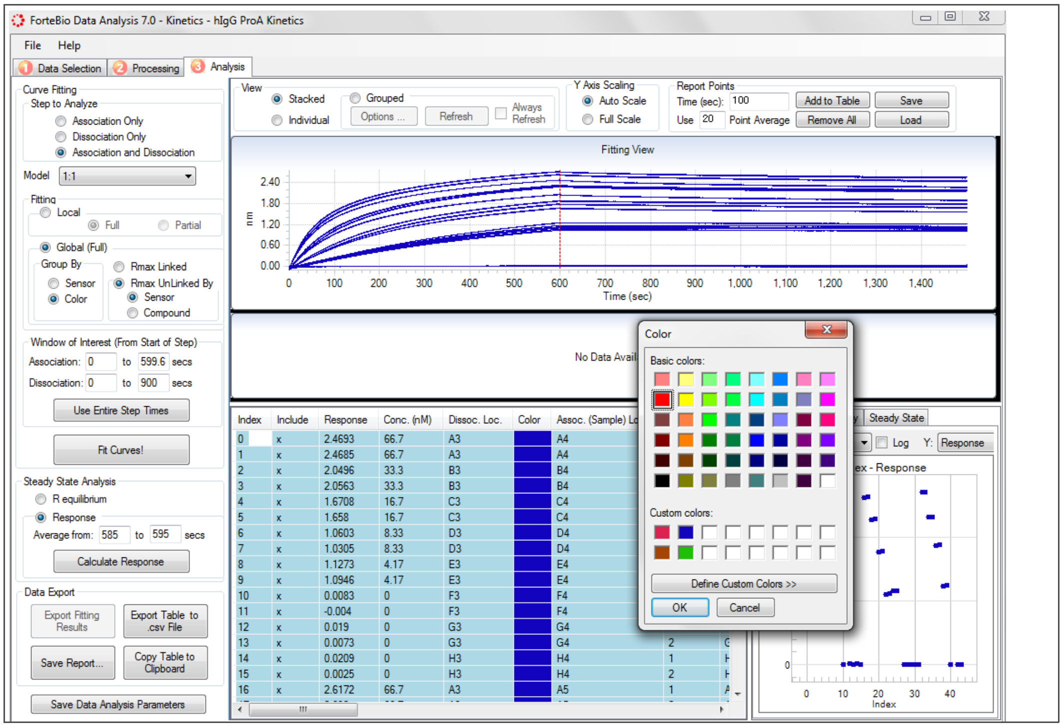

Figure 5-53: Changing the Color of User-Selected Results

3. In the color palette that appears, select a basic color or create a custom color. To define a custom color, click **Define Custom Colors**.
4. Click **OK** in the color palette.

The selected color is applied to the binding curve and appears in the table.

Results can be color-coded by category to group them for a global fit (for example, colored by compound), then fit using global fit by color. For the final display, the wells can be re-colored without affecting the results.

Color-Coding Analysis Results by Category

To color-code analysis results by category:

1. Right-click the analysis results table and select **Set Color By**.
2. Make a selection from the shortcut menu (Figure 5-54).

   In Figure 5-54's example, *category* = sample concentration.

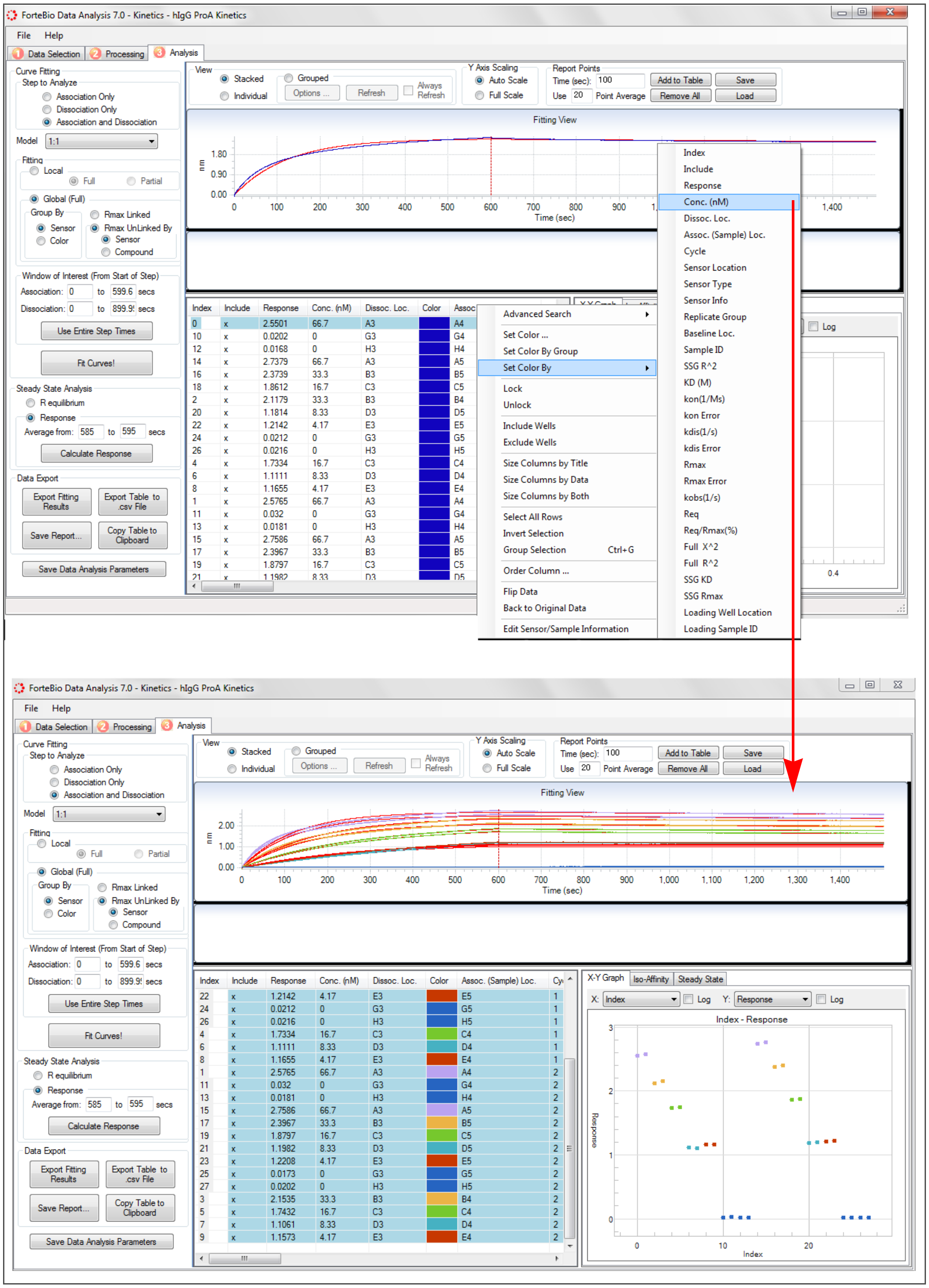

Figure 5-54: Setting Data Color by Category

Sorting Analysis Results

Sorting in Ascending or Descending Order

To sort results by any category (column header):

1. Click a column header.

   The results are displayed in descending order. In Figure 5-55, the results were color-coded by sample concentration, and then sorted.

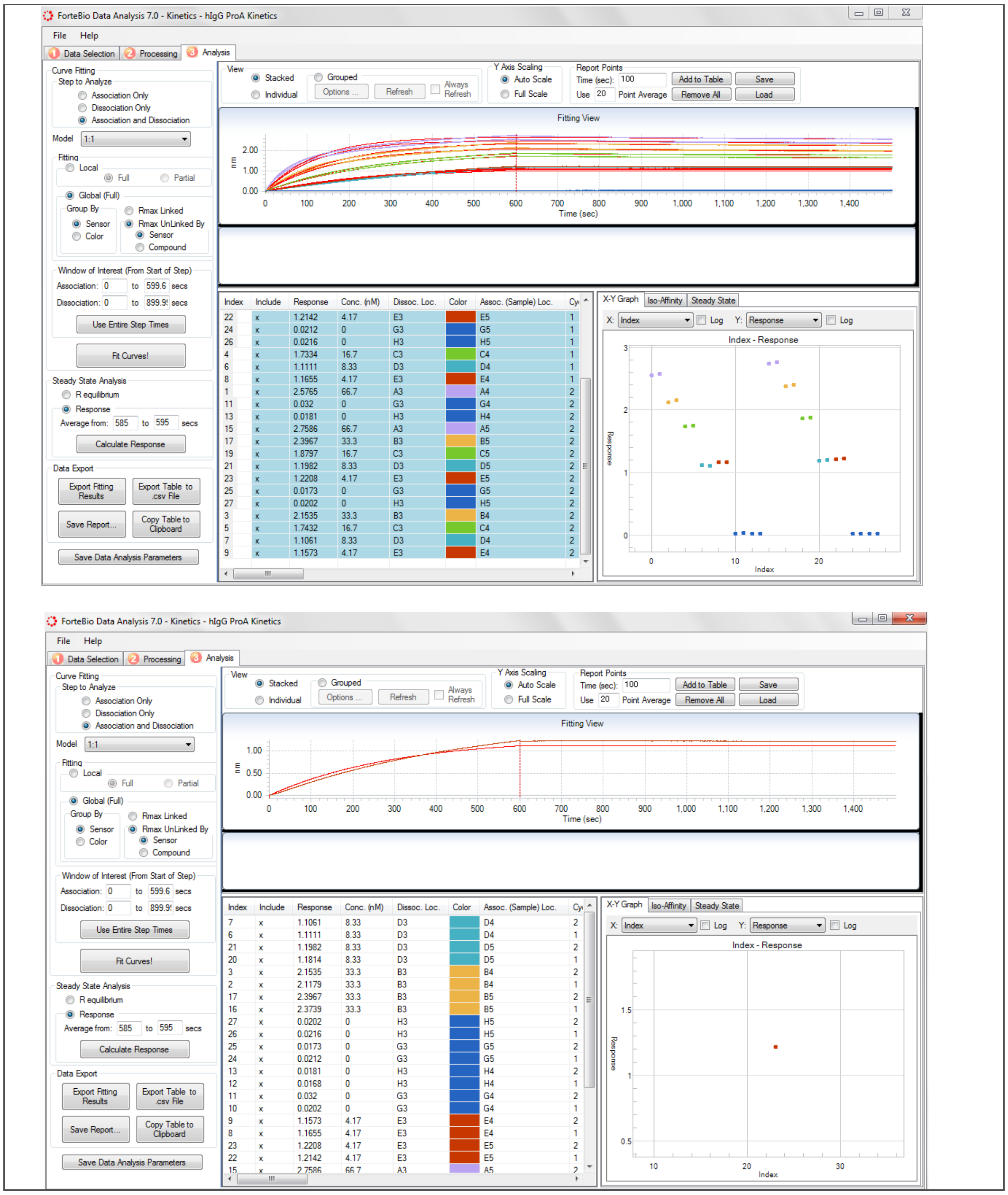

Figure 5-55: *Color-Coded Analysis Results—Sorted by Color*

2. Click the column header again to sort the results in ascending order.

Sorting in Parallel Order

To perform parallel sorting:

1. Right-click the Results table and select **Parallel Sorting** (Figure 5-56).

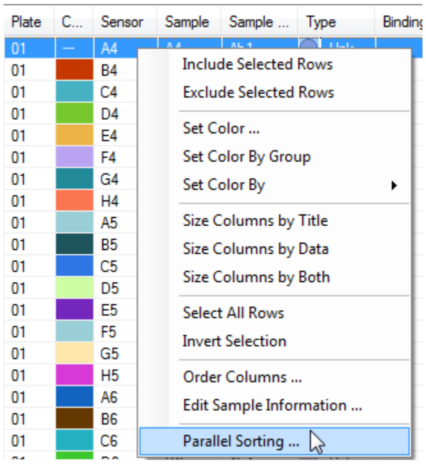

Figure 5-56: *Parallel Sorting Menu*

2. In the Parallel Sorting dialog box, select the desired sorting parameters and click **OK** (Figure 5-57).

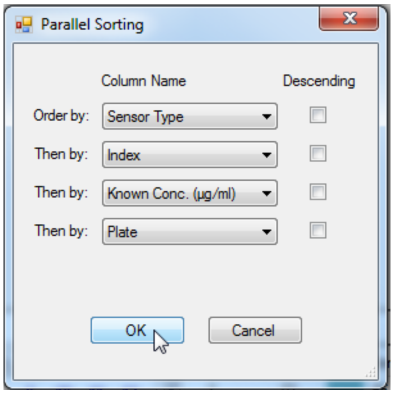

Figure 5-57: *Parallel Sorting Dialog Box*

The Results Table will re-sort based on the parameters and order selected.

WORKING WITH GRAPHS

The active analysis results are automatically presented in three graphical formats:

- X-Y
- Iso-Affinity
- Steady State Analysis

X-Y Graphs

The X-Y graph is a scatter plot from user-selected analysis results (x and y-variables). Both axes may be presented on either a logarithmic or linear scale.

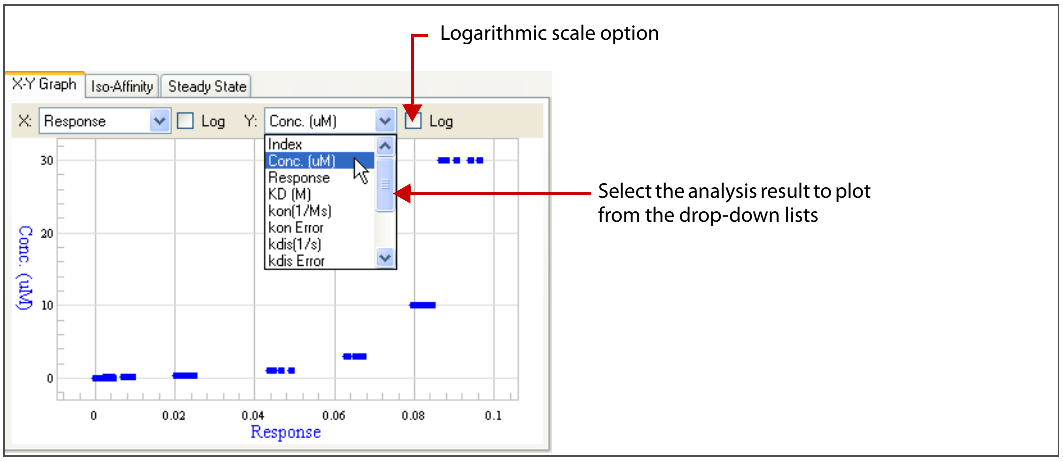

Figure 5-58: *X-Y Graph*

An X-Y plotting tool has been added to the **Results** tab of quantitation analysis. Previously available only in kinetics analysis, the X-Y plotting tool graphs several important parameters, such as binding rate, R2, calculated concentration, and residual. The axes may be independently selected to by logarithmic. In the example shown, the calculated concentration is plotted versus the R2 (Figure 5-59).

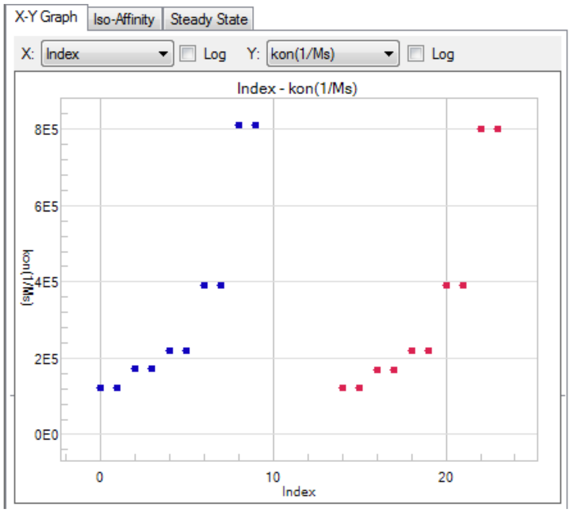

Figure 5-59: *X-Y Plotting Tool*

Iso-Affinity Graphs

The Iso-Affinity graph enables viewing of the continuum of k_{dis} and k_{on} values that generate a single value of K_D, providing a convenient way to view both kinetic and affinity data. The value of the affinity constant, K_D, is the ratio of the association rate k_{on} and dissociation rate k_{dis}. A single value of K_D can, therefore, be obtained from varying values of k_{on} and k_{dis}; for example, a K_D value of 1 µM can be the result of k_{dis}=1x10–3 1/S and k_{on}=1x10+3 1/Ms or k_{dis}=1x10-2 1/S and k_{on}=1x10+4 1/Ms.

Each Iso-Affinity plot has two red lines that correspond to a single K_D value. The position of the K_D lines is determined by taking the average of all K_D values and plotting one redline 10 fold lower than the average and one red line 10 fold higher than the average.

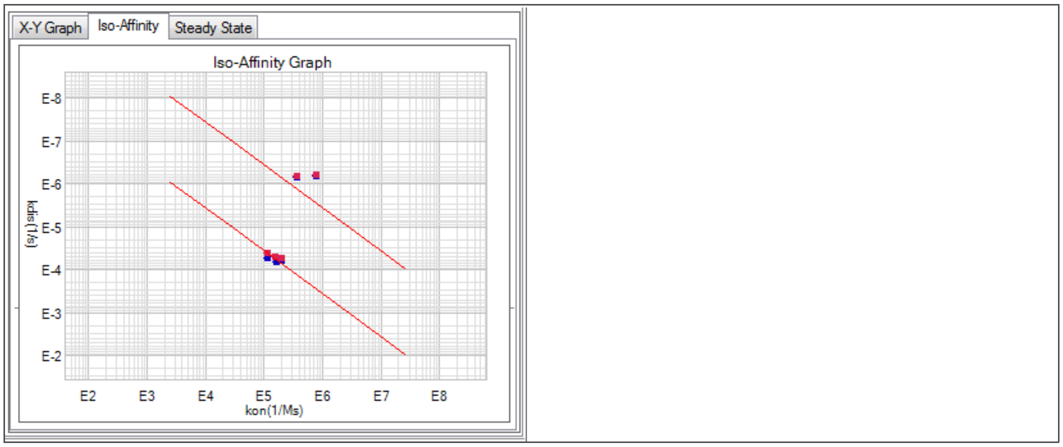

Figure 5-60: *Iso-Affinity graph—X axis = k_a, Y -axis = k_d*

Steady State Analysis Graphs

The Steady State Analysis graph (Figure 5-61) displays the results from the steady state analysis (see “Steady State Analysis Graphs” on page 144). The graph plots the response or Req vs. concentration and the curve fit.

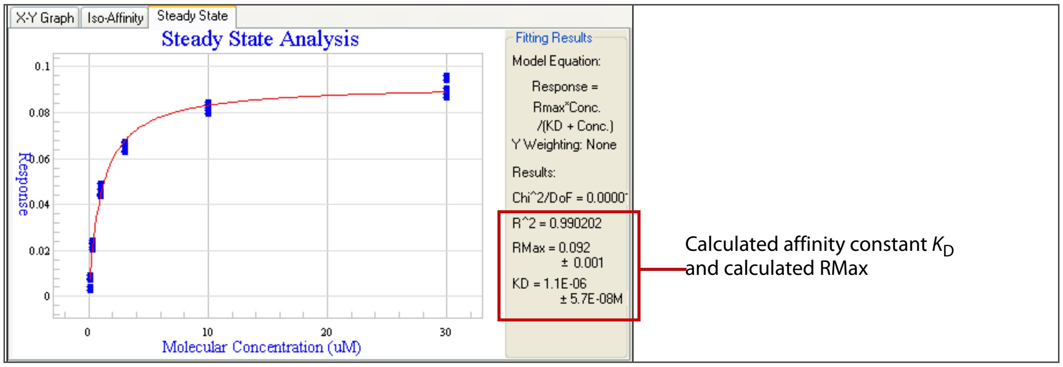

Figure 5-61: *Steady State Analysis Graph*

DATA EXPORT OPTIONS

The analysis results can be exported (.txt or .csv) or copied to the system clipboard. Information from the Data Selection, Processing, or Analysis windows can be selected to generate custom reports (.xls).

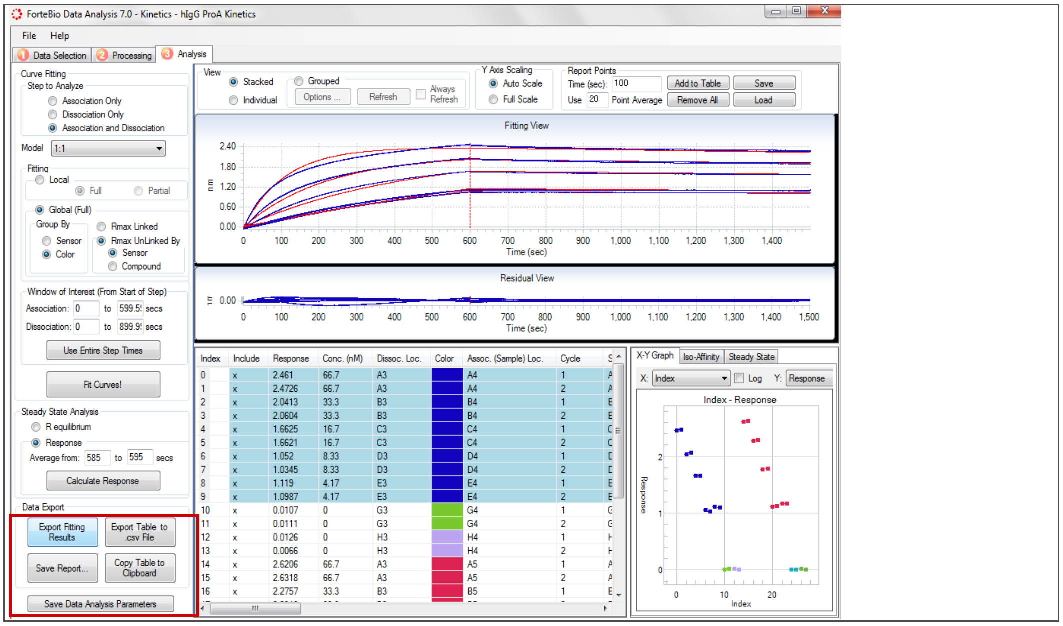

Figure 5-62: *Data Export Options in the Analysis Window*

The data export options include:

- **Export Fitting Results**—Saves the binding data and analysis results for each biosensor to a separate text file (.txt).
- **Export Table to .csv File**—Saves the results table to a .csv file that can be opened in a spreadsheet application.
- **Copy Table to Clipboard**—Saves the binding data and analysis results for the selected biosensors to the system clipboard.

MASKING DATA

The Mask Data feature lets you make a copy of your data folder with all proprietary info such as sample ID, sample info, sensor type and sensor info hidden, or 'masked' in both the .fmf and .frd files.

NOTE: *The Mask Data feature is not available in Octet Data Analysis 21 CFR Part 11 software.*

To do this:

1. In the Data Selection Tab, right click on an experiment in the folder tree.
2. Select **Mask Data** (Figure 5-63).

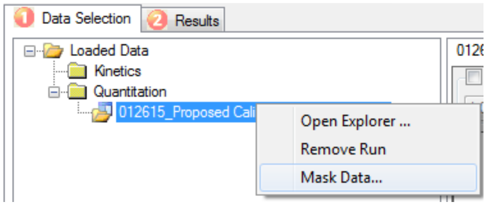

Figure 5-63: *Selecting Mask Data Option.*

A copy of your data folder can then be saved in the desired location.

GENERATING A REPORT

NOTES:

Generating reports for large data sets may take a few minutes. For faster report generation, minimize the number of items selected for the report. In particular, the sensor summary for a large data set can be time-consuming.

In Octet Data Analysis 21 CFR Part 11 software versions 8.2 and higher, the Summary Worksheet tab of exported reports will now also include the software name, version and the export date and time.

To generate a report:

1. On the menu bar **File** > **Save Report**. Alternatively you can click **Save Report** in the Data Export section of the Analysis tab (Figure 5-64).
2. In the displayed dialog box, select the information to include in the report.
3. Confirm the default location to which the file will be saved or specify a different location.
4. Click **Export**.

   In Octet Data Analysis software versions 8.2 and higher, once the report is exported, the contents of the exported .xls file are locked and cannot be changed.

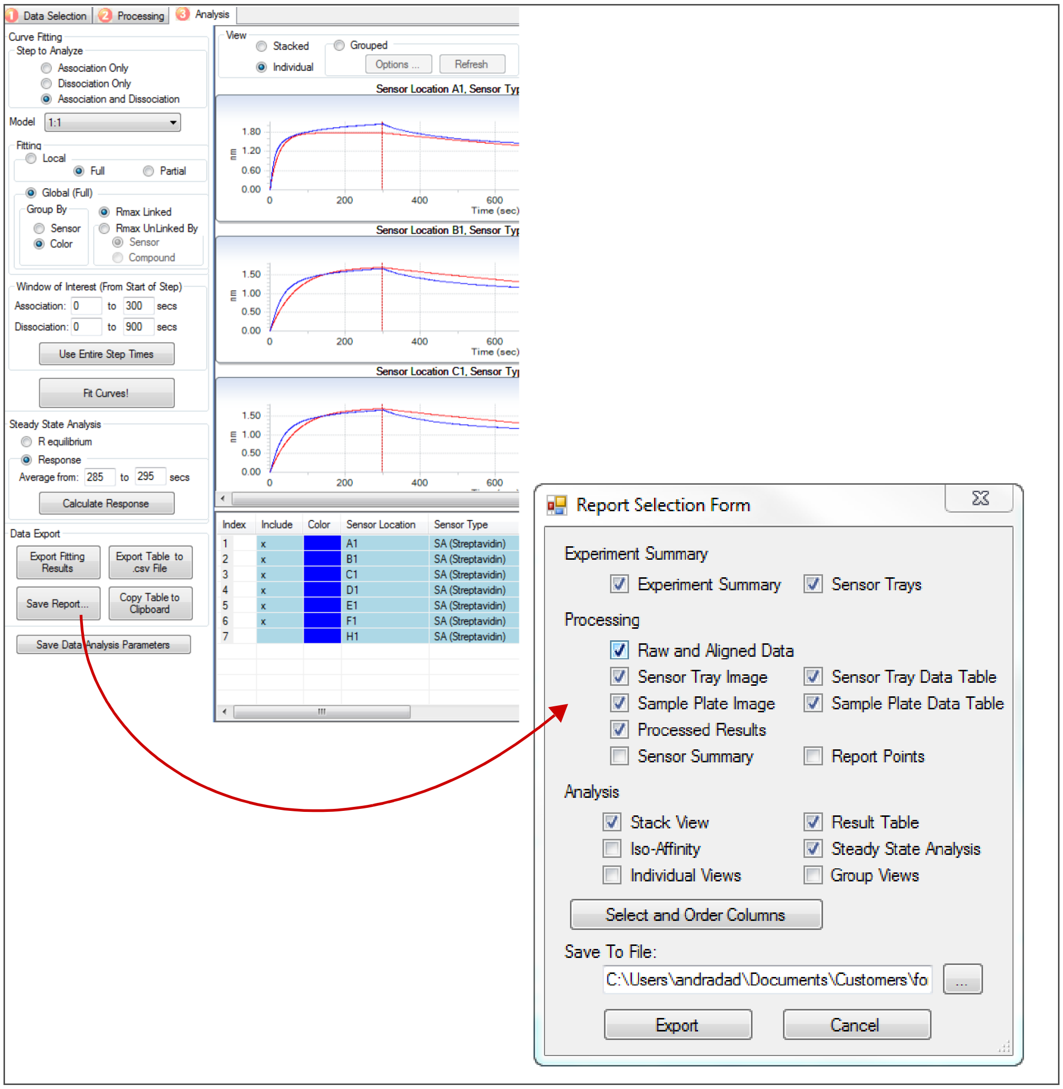

Figure 5-64: Report Selection Form

Changing the Column Order in a Saved Report

You can customize the columns and their order in a saved report. These customized report settings are also saved as the default for future reports.

1. In the Data Export section of the Analysis tab, click **Save Report** > **Select and Order Columns** > **OK** (Figure 5-64).
2. The Select And Order Columns dialog box displays (Figure 5-65). Select and order the columns, then click **OK**.

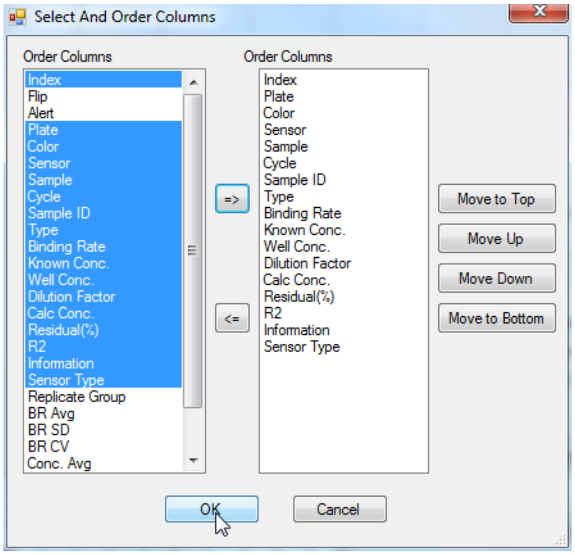

***Figure 5-65:** Select And Order Columns Dialog Box*

3. In the Report Selection Form dialog box (Figure 5-64), click **Export**. The Results tab will now display the selected columns and the desired column order.

Experiment Summary Options

To use the available experiment summary options, select the following from the Data Selection window (1 tab):

- Steps data table
- Assay steps data table

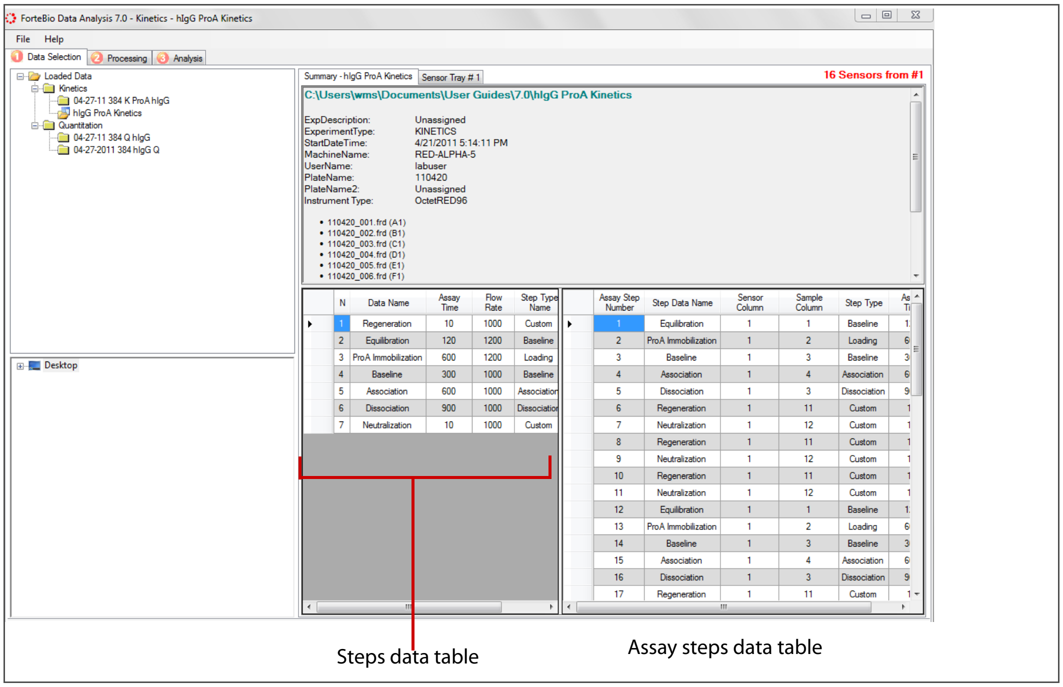

Figure 5-66: *Report Data Types—**Experiment Summary** Options*

Processing Options

To use the available processing options, select the following from the Processing window (tab):

- Raw and aligned data
- Sensor tray image
- Sample plate image
- Processed results
- Sensor summary
- Sensor tray data table
- Sample plate data table
- Report points

NOTE: *When working with large data sets, including the sensor summary in a report significantly increases the time required to generate the report.*

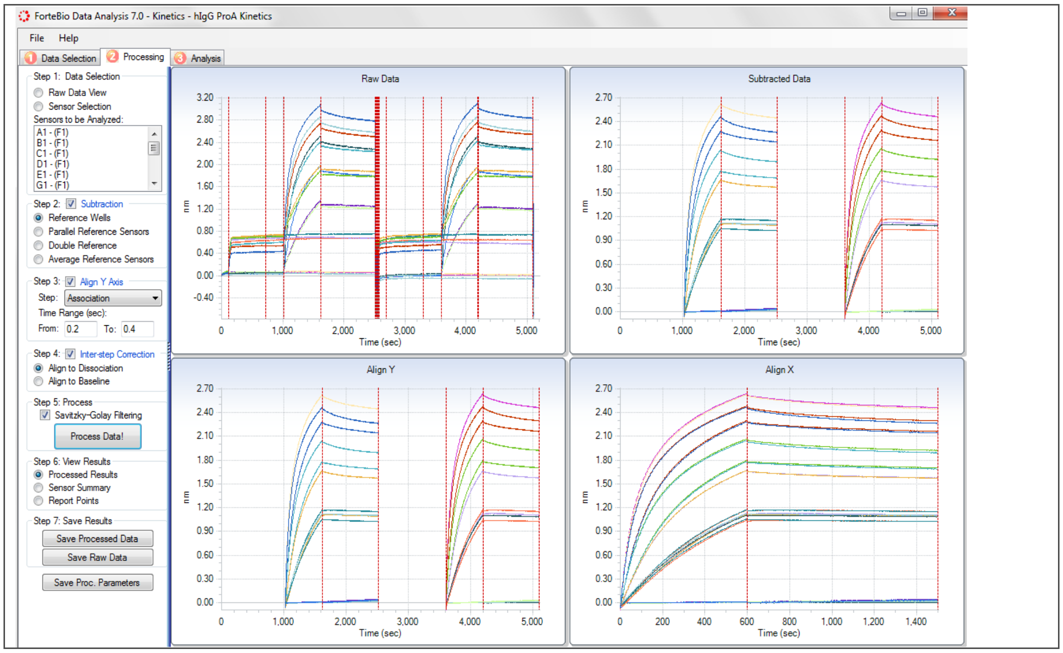

*Figure 5-67: Report Data Types—**Processing** Options—Raw Data and Aligned Data*

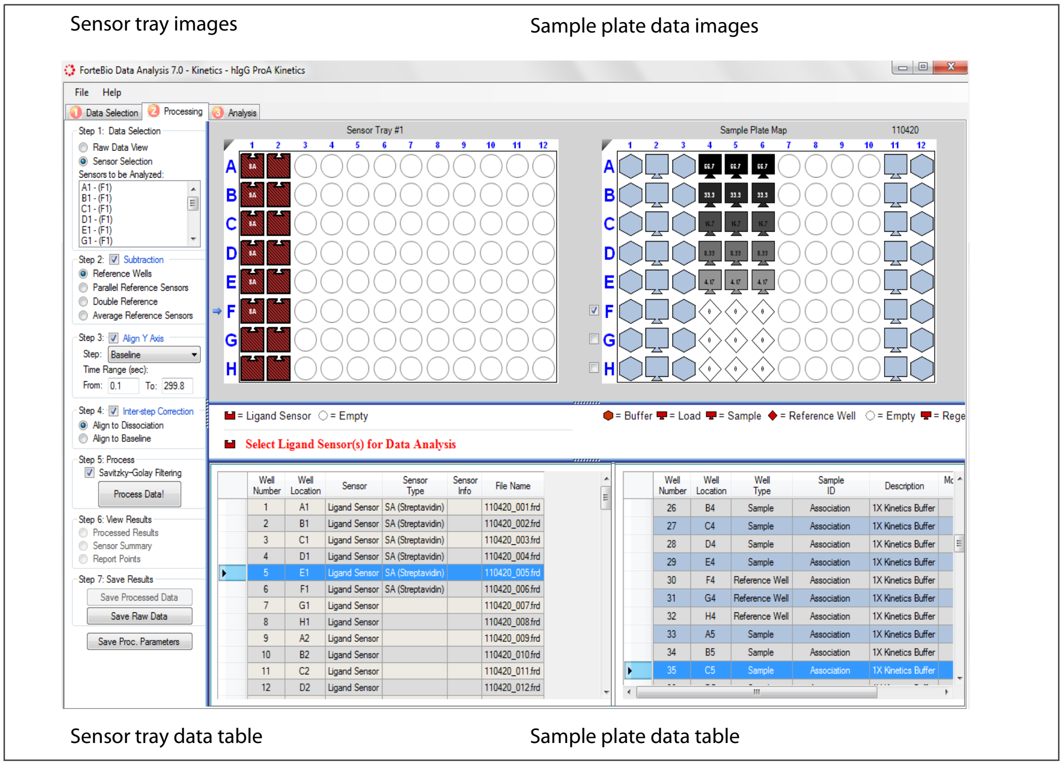

*Figure 5-68: Report Data Types—**Processing** Options—Sensor Tray and Sample Tray Information*

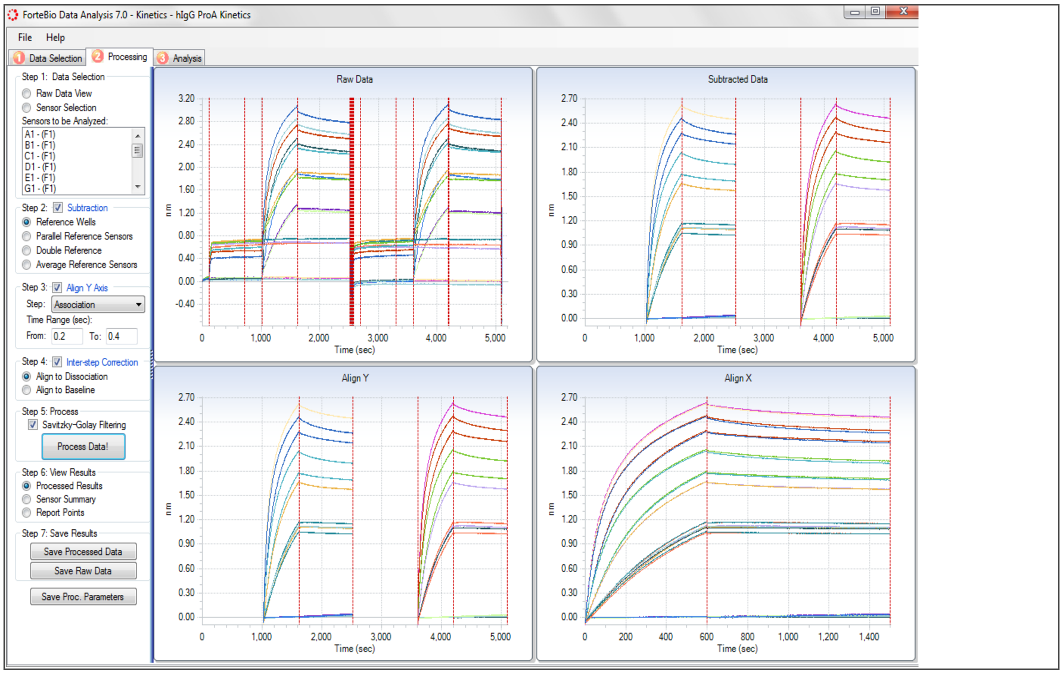

Figure 5-69: *Report Data Types—**Processing** Options—Processed Data*

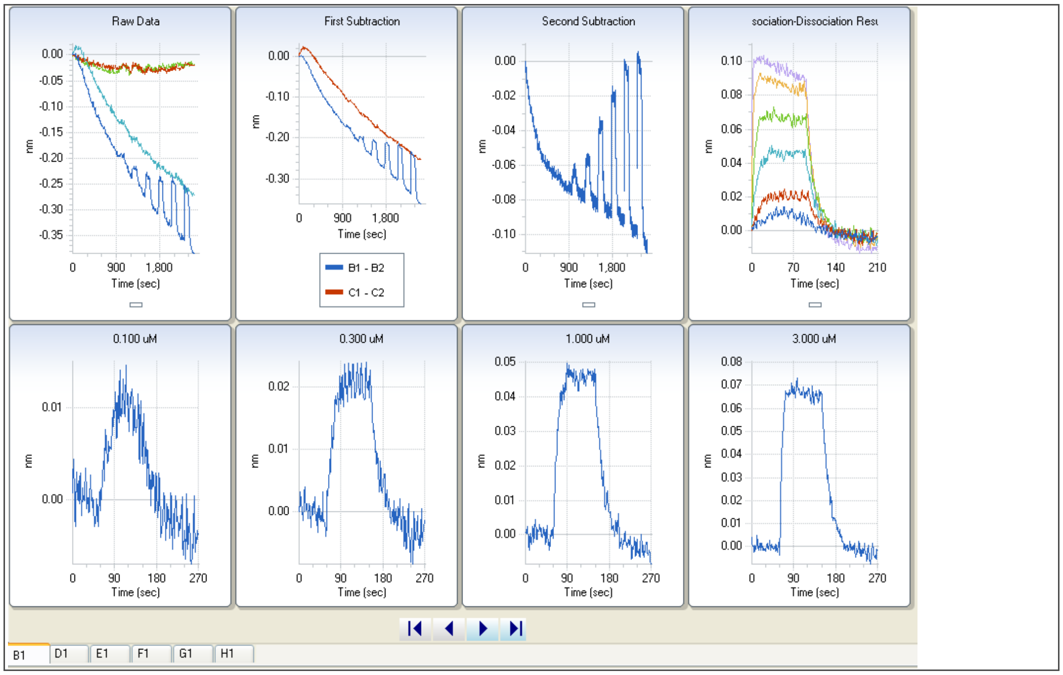

Figure 5-70: *Types of Report Data Included by the **Processing** Options—Sensor Summaries*

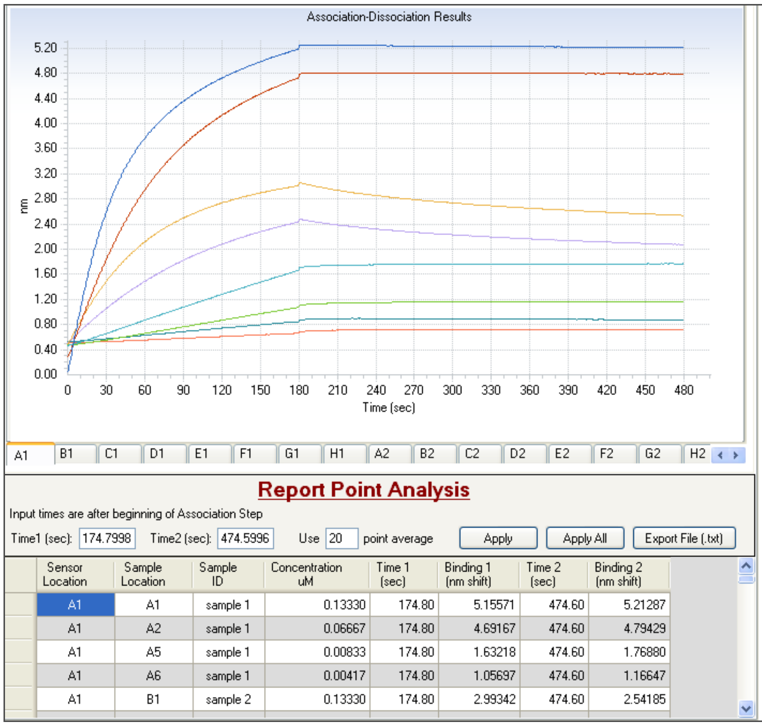

*Figure 5-71: Report Data Types—**Processing** Options—Report Point Analysis*

Analysis Options

To use the available analysis options, select the following from the Analysis window (3 tab):

- Kinetics analysis
- Iso-Affinity analysis
- Steady State analysis

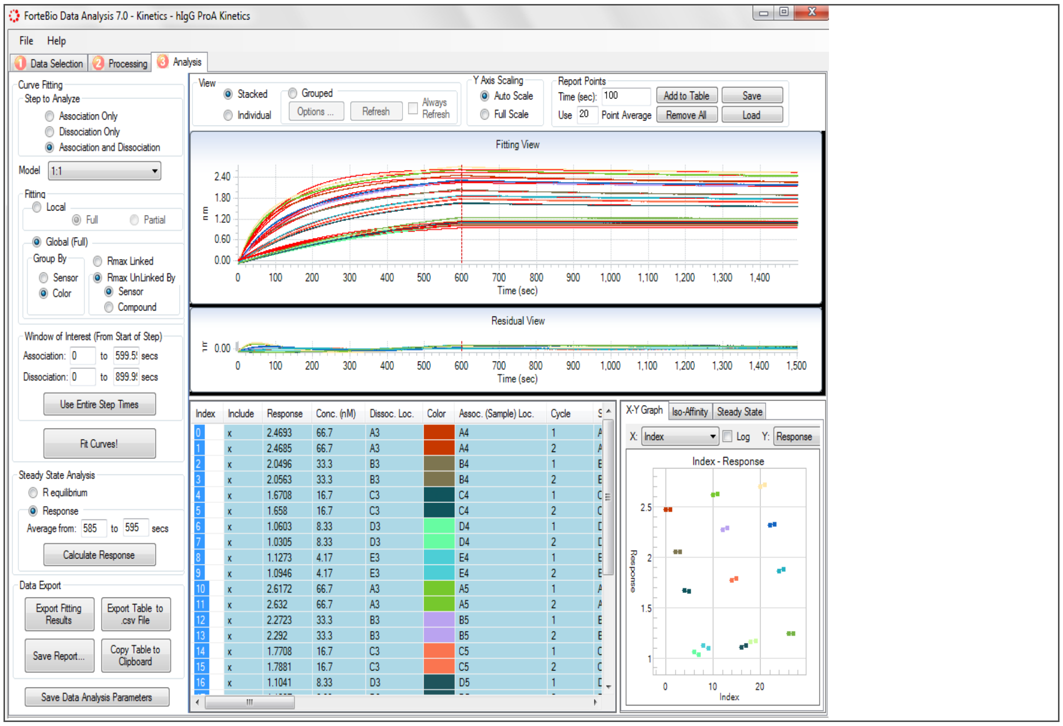

*Figure 5-72: Report Data Types—**Analysis Options**—Kinetics Analysis Results*

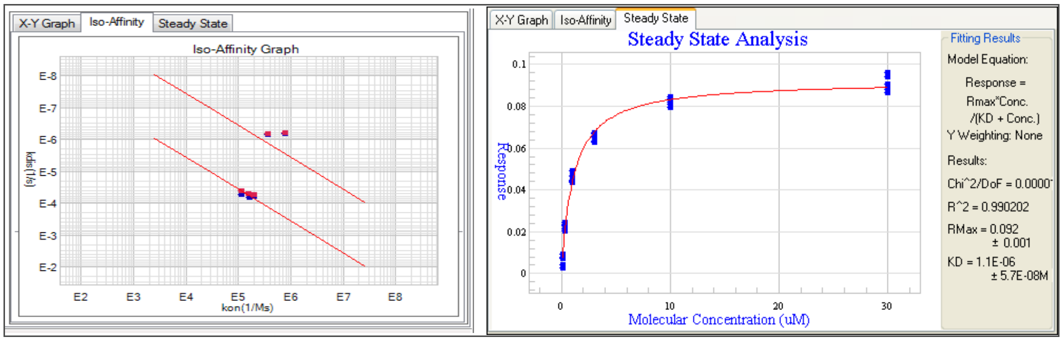

*Figure 5-73: Report Data Types—**Processing** Options—Iso-Affinity and Steady State Analysis Graphs*

EPITOPE BINNING

The goal of a typical epitope binning or cross-blocking experiment is to identify antibodies which bind to different or identical epitopes on the antigen. Antibodies are tested two at a time for competitive binding to one antigen. By competing antibodies against one another in a pairwise and combinatorial format, antibodies with distinct blocking behaviors can be discriminated and assigned to “bins”. The end result is matrix of pairwise binders and blockers.

Epitope binning experiments are run as kinetic experiments with many repeating steps in the Octet Data Acquisition software. After an experiment is complete, start a data analysis session. The steps to analyze the binning data include:

Table 5-1: *Octet Data Analysis Steps for Epitope Binning Assays*

Octet Software	*Functions*
Data Analysis	1. Load and open the experiment data.<br>2. Confirm experiment parameters and select biosensors in Data Selection.<br>3. Click **Process Epitope Binning** in Tab 2.<br>4. View and analyze graphical and tabular results in Tab 4 (Binning).<br>5. Export the results and generate a report.

The Epitope Binning data analysis feature provides easier visualization of larger data sets that use biosensor regeneration. Results are placed in a 2-D matrix that allows further cross-blocking data analysis. Typical epitope binning or cross-blocking analysis focuses on comparing cycles of repeating Association steps, with monitoring of baselines or Dissociation steps for binding stability.

Start a Data Analysis Session

1. Click the desktop Octet Data Analysis software icon to start a data analysis session.
2. Select a data file and view the experiment summary in Tab 1 (Data Selection).

NOTE: *More details on opening data files can be found "Working with Experiments" on page 80.*

NOTE: *The Sample plate and the Reagent plate are now referred to as "Plate 1" and "Plate 2" in the software.*

Processing Epitope Binning Data

1. Click Tab 2 (Processing).
2. Click the **Process Epitope Binning** button and open Tab 4 (Epitope Binning):

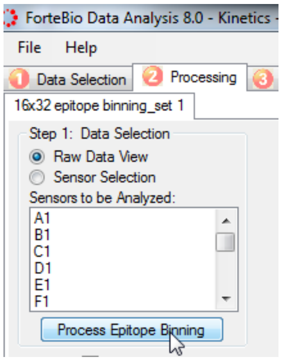

***Figure 5-74:** Process Epitope Binning Data Button*

Tab 4 (Binning) has three sections: Binning Parameters and Step Calculations (left), Graph of overlaid traces (upper right), and Table of averaged sensor and cycle data (bottom right):

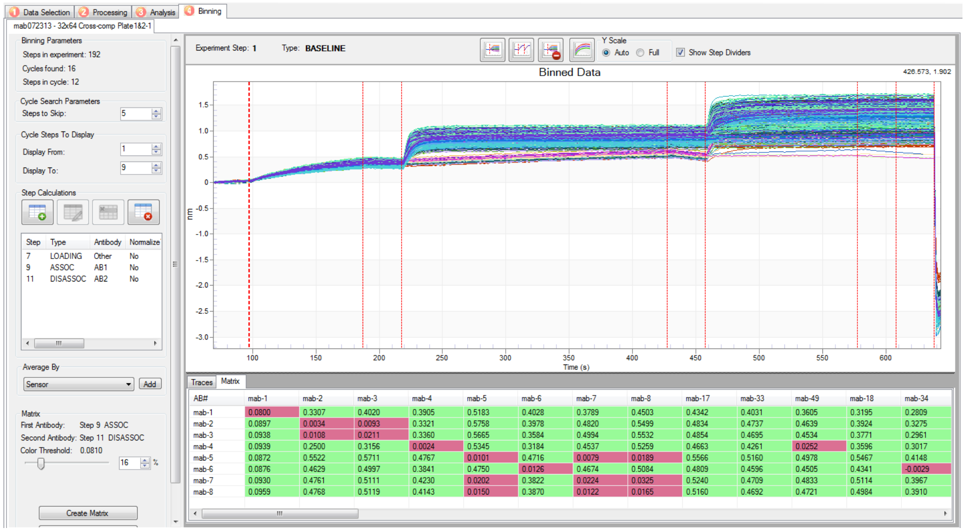

***Figure 5-75:** Tab 4 (Binning)*

Graph of Overlaid Traces. The software will automatically search for repeating sequences of baseline, loading and/or association steps and overlay them in a graph:

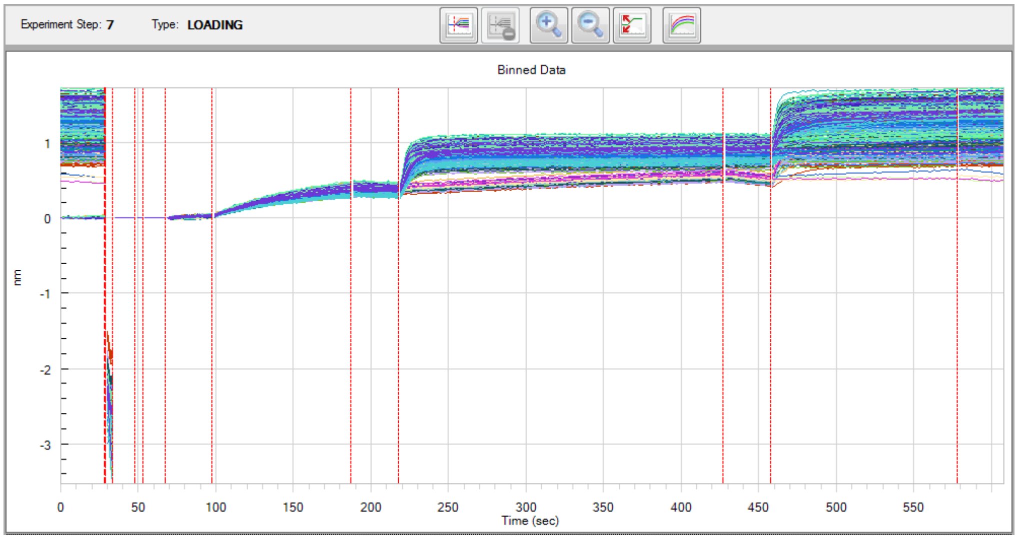

Figure 5-76: *Graph of Overlaid Traces*

The selected assay step icon is highlighted above the graph, in bold red-dashes on the graph itself and displayed as its Experiment Step in the upper left.

To change display options using the buttons above the graph:

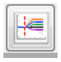 Align traces to start of selected step

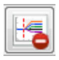 Remove trace alignment

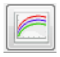 Show all traces

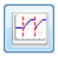 Align traces to start of each step

Binning Parameters. Includes the total number of steps found in the experiment, the number of cycles of repeating steps, and the number of steps found in one cycle:

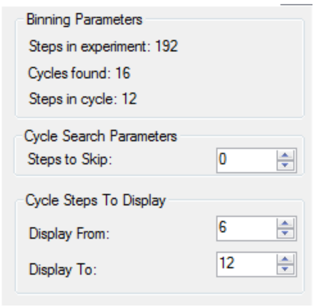

Figure 5-77: *Binning Parameters*

Steps to Skip. This is useful in assays where a number of initial assay steps are followed by a set of repetitive cycles. When comparing repeating association steps in a binning experiment, the Steps to Skip option allows the user to ignore steps that do not need to be overlaid for visualization of the binning results.

Cycle Steps to Display. The Cycle Steps to Display allows you to reduce the steps displayed on the graph view, such as any regeneration steps.

Adjust the Steps to Skip and/or Cycle Steps to Display to properly view the overlaid traces:

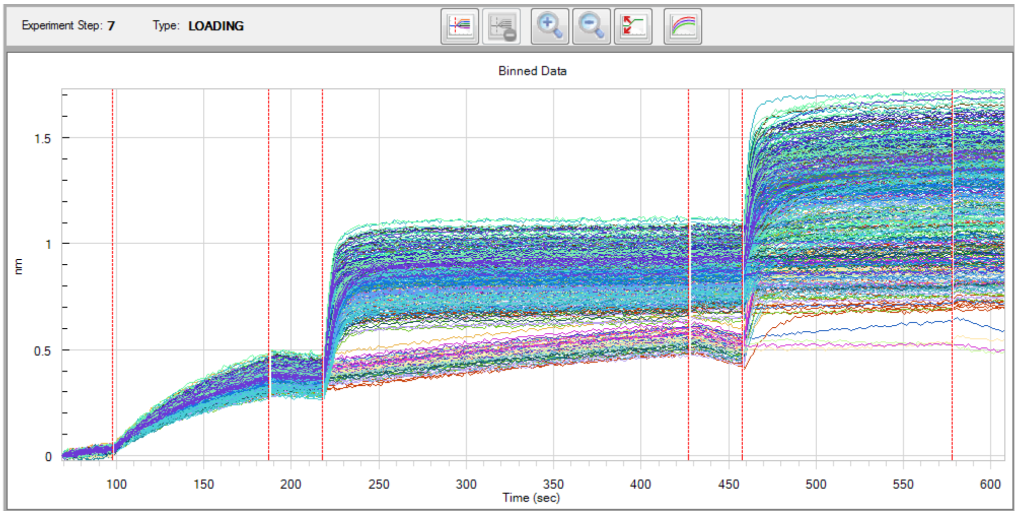

Figure 5-78: *Overlay of Traces with Steps to Skip Removed and Cycle Steps Displayed*

Step Calculations. Includes commands used to select assay steps for building the Step Calculation table and calculating parameters for the epitope binning matrix:

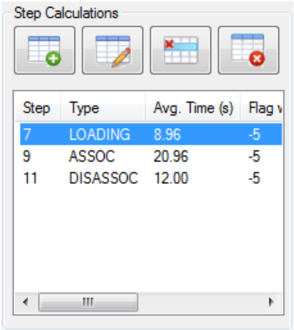

Figure 5-79: *Step Calculations*

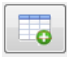 Add selected step to Step Calculation table

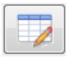

Edit selected step

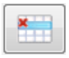

Remove selected step from Step Calculation table

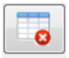

Clear Step Calculation table

Add a step for calculating the epitope binning matrix by selecting a step in the graph and clicking the **Add Step Calculation Parameters** button. This opens the Step Calculation Parameters box:

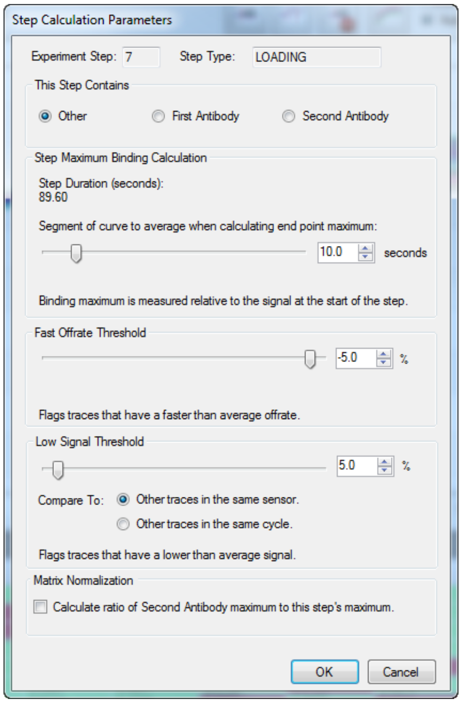

Figure 5-80: *Step Calculation Parameters Box*

To calculate the epitope binning matrix:

1. Select what this step contains: First Antibody, Second Antibody or None (e.g. Baseline or Antigen).
2. Change the default time window for determining the average nm shift at the end of the step.
3. Add weak binding and low response flags (flagged in the Traces tab):
   - **Weak Binding %:** flags traces that have dropped based on the % dissociation from the start of the step.

- **Low Response %:** flags traces that have not risen relative to the average nm shift of the step.

4. Normalize the second antibody step data by clicking the **Use this step to normalize the second antibody** check box.
5. Click **OK**.
6. Repeat the previous steps to build the Step Calculations table.

Creating the Matrix. Once the Step Calculations table is complete with at least the First Antibody and Second Antibody, a matrix can be generated by clicking the **Create Matrix** button:

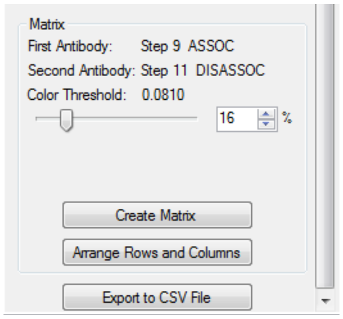

Figure 5-81: Create Matrix Button

The matrix created on the Matrix tab is the nm shift, or normalized data, of the First Antibody against the nm shift, or normalized data, of the Second Antibody competitively binding to the antigen:

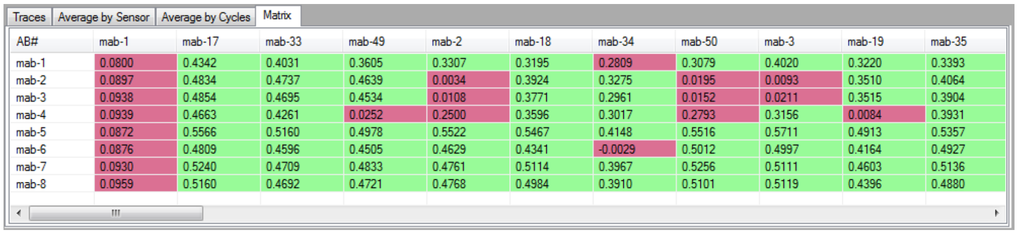

AB#	mab-1	mab-17	mab-33	mab-49	mab-2	mab-18	mab-34	mab-50	mab-3	mab-19	mab-35
mab-1	0.0800	0.4342	0.4031	0.3605	0.3307	0.3195	0.2809	0.3079	0.4020	0.3220	0.3393
mab-2	0.0897	0.4834	0.4737	0.4639	0.0034	0.3924	0.3275	0.0195	0.0093	0.3510	0.4064
mab-3	0.0938	0.4854	0.4695	0.4534	0.0108	0.3771	0.2961	0.0152	0.0211	0.3515	0.3904
mab-4	0.0939	0.4663	0.4261	0.0252	0.2500	0.3596	0.3017	0.2793	0.3156	0.0084	0.3931
mab-5	0.0872	0.5566	0.5160	0.4978	0.5522	0.5467	0.4148	0.5516	0.5711	0.4913	0.5357
mab-6	0.0876	0.4809	0.4596	0.4505	0.4629	0.4341	-0.0029	0.5012	0.4997	0.4164	0.4927
mab-7	0.0930	0.5240	0.4709	0.4833	0.4761	0.5114	0.3967	0.5256	0.5111	0.4603	0.5136
mab-8	0.0959	0.5160	0.4692	0.4721	0.4768	0.4984	0.3910	0.5101	0.5119	0.4396	0.4880

Figure 5-82: Matrix Tab

Matrix Parameters Summary. The steps associated with the First Antibody and Second Antibody are displayed, along with commands for Color Threshold and Arranging Rows and Columns:

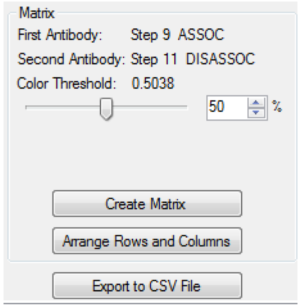

***Figure 5-83:** Matrix Parameters Summary*

1. Move the slider to change the threshold for changing color on the matrix.
2. Click the **Arrange Rows and Columns** button to sort and match columns and rows:

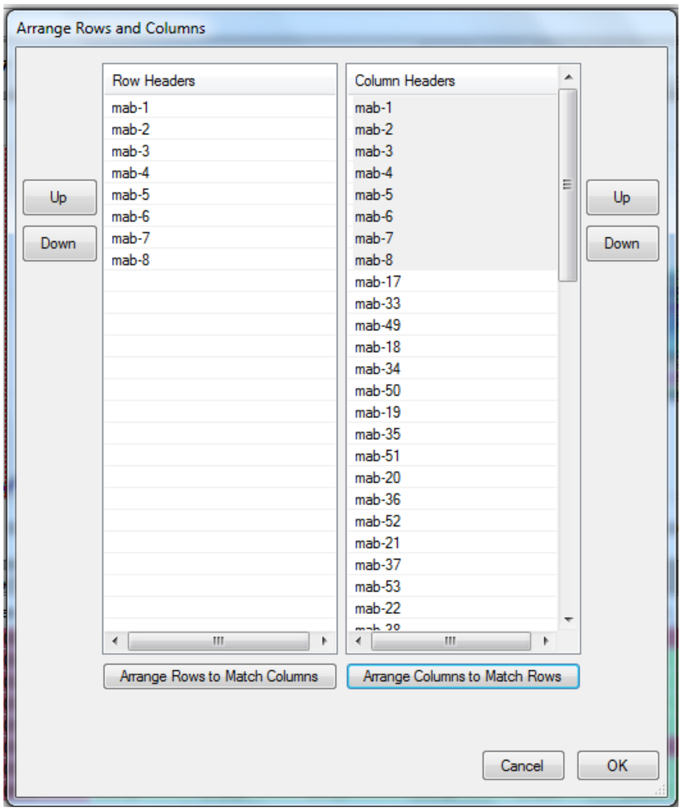

***Figure 5-84:** Arrange Rows and Columns Box*

- Rows and columns can be sorted alphabetically by clicking on the headers:

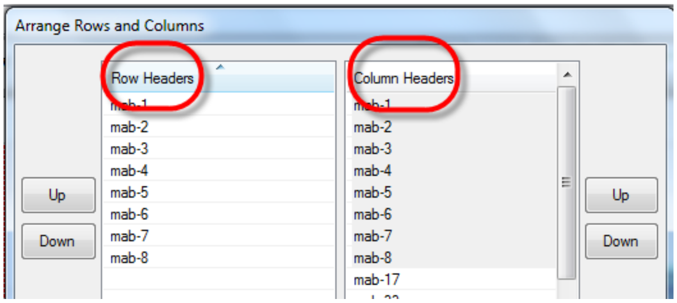

Figure 5-85: Alpha Row and Column Sorting

- Hold the **Ctrl key** to click and select multiple entries.
- Scroll through entries faster by holding down the **Up** or **Down** buttons.

3. Click **OK**.

Matrix data can be copied by right-clicking on the matrix, selecting **Copy** and pasting into another program. Data can be exported by clicking the **Export to CSV File** button.

- **Results Table.** Shows the trace data averaged for sensor and cycle:

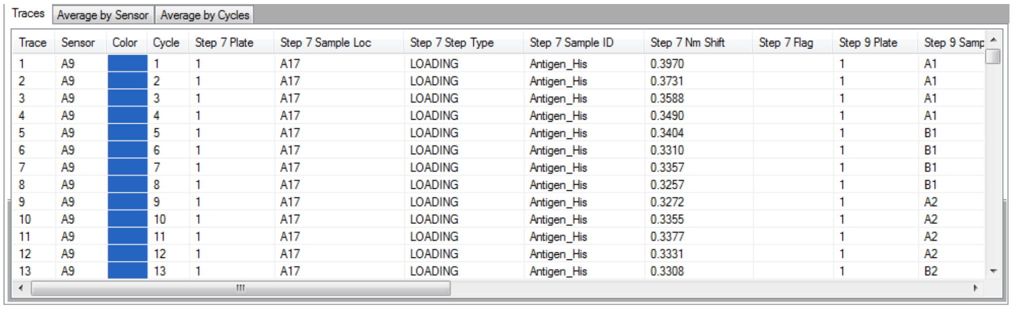

Figure 5-86: Results Table

- **Average By drop down list:** lets you choose the method for averaging trace information:

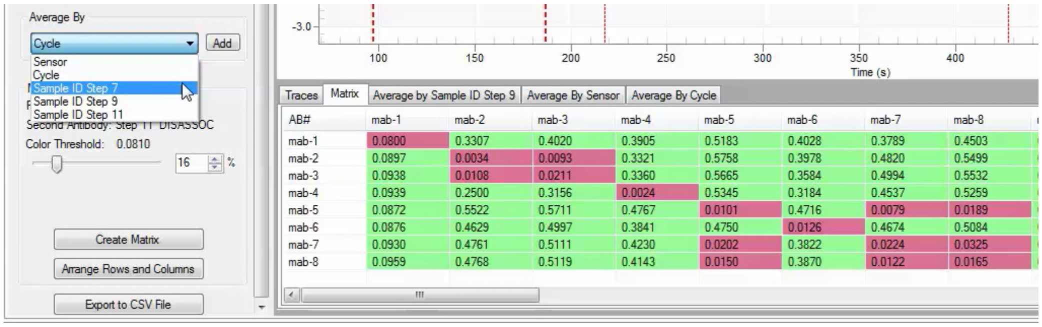

Figure 5-87: Average By Drop Down List

- **Traces tab:** contains individual cycle data for each sensor:

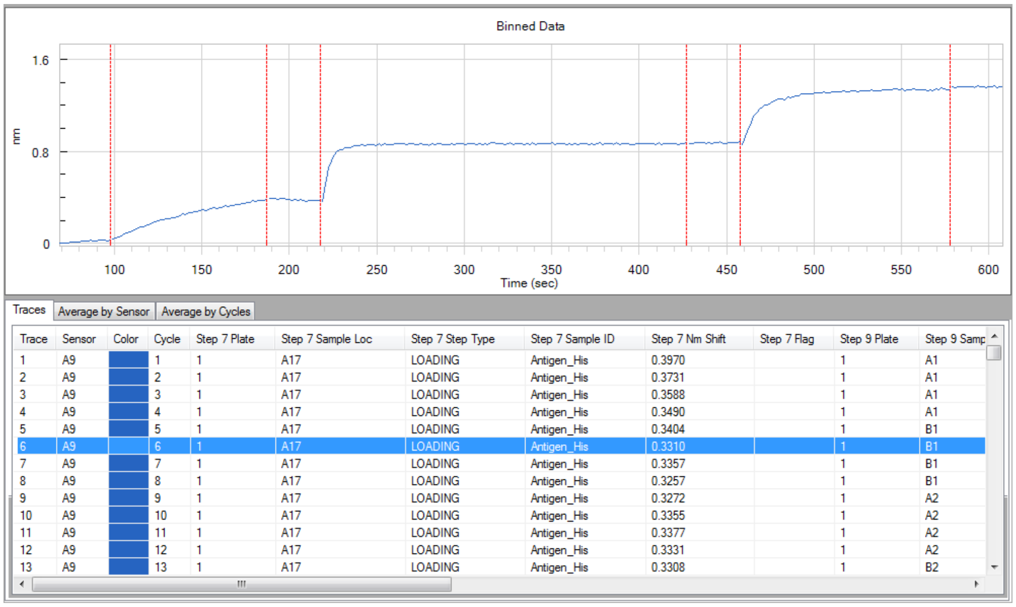

Trace	Sensor	Color	Cycle	Step 7 Plate	Step 7 Sample Loc	Step 7 Step Type	Step 7 Sample ID	Step 7 Nm Shift	Step 7 Flag	Step 9 Plate	Step 9 Samp
1	A9		1	1	A17	LOADING	Antigen_His	0.3970		1	A1
2	A9		2	1	A17	LOADING	Antigen_His	0.3731		1	A1
3	A9		3	1	A17	LOADING	Antigen_His	0.3588		1	A1
4	A9		4	1	A17	LOADING	Antigen_His	0.3490		1	A1
5	A9		5	1	A17	LOADING	Antigen_His	0.3404		1	B1
6	A9		6	1	A17	LOADING	Antigen_His	0.3310		1	B1
7	A9		7	1	A17	LOADING	Antigen_His	0.3357		1	B1
8	A9		8	1	A17	LOADING	Antigen_His	0.3257		1	B1
9	A9		9	1	A17	LOADING	Antigen_His	0.3272		1	A2
10	A9		10	1	A17	LOADING	Antigen_His	0.3355		1	A2
11	A9		11	1	A17	LOADING	Antigen_His	0.3377		1	A2
12	A9		12	1	A17	LOADING	Antigen_His	0.3331		1	A2
13	A9		13	1	A17	LOADING	Antigen_His	0.3308		1	B2

Figure 5-88: Traces Tab

- **Average by Sensor tab:** contains sensor information with average nm shift for each cycle across individual sensors:

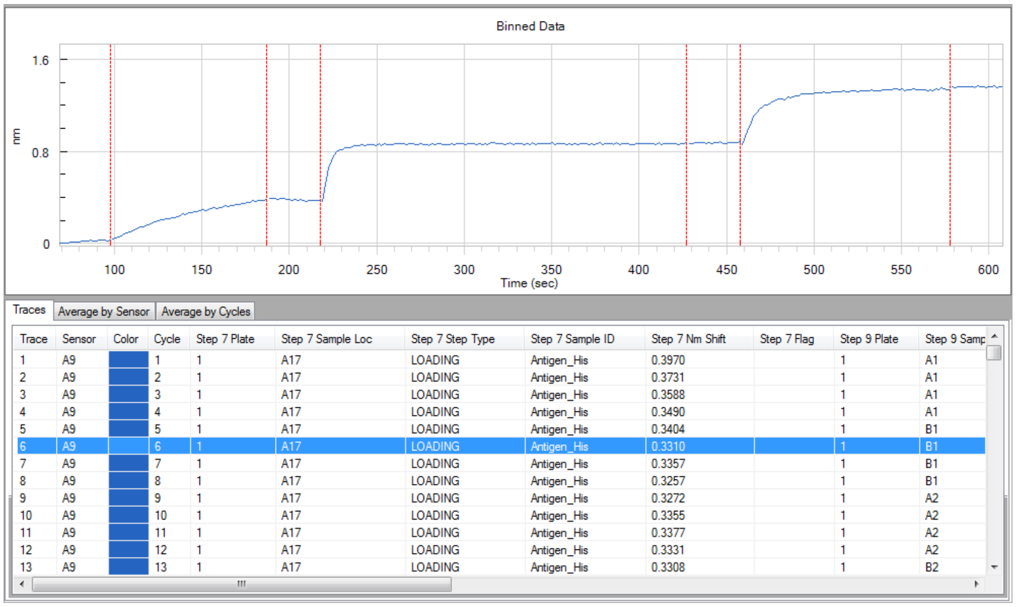

Trace	Sensor	Color	Cycle	Step 7 Plate	Step 7 Sample Loc	Step 7 Step Type	Step 7 Sample ID	Step 7 Nm Shift	Step 7 Flag	Step 9 Plate	Step 9 Samp
1	A9		1	1	A17	LOADING	Antigen_His	0.3970		1	A1
2	A9		2	1	A17	LOADING	Antigen_His	0.3731		1	A1
3	A9		3	1	A17	LOADING	Antigen_His	0.3588		1	A1
4	A9		4	1	A17	LOADING	Antigen_His	0.3490		1	A1
5	A9		5	1	A17	LOADING	Antigen_His	0.3404		1	B1
6	A9		6	1	A17	LOADING	Antigen_His	0.3310		1	B1
7	A9		7	1	A17	LOADING	Antigen_His	0.3357		1	B1
8	A9		8	1	A17	LOADING	Antigen_His	0.3257		1	B1
9	A9		9	1	A17	LOADING	Antigen_His	0.3272		1	A2
10	A9		10	1	A17	LOADING	Antigen_His	0.3355		1	A2
11	A9		11	1	A17	LOADING	Antigen_His	0.3377		1	A2
12	A9		12	1	A17	LOADING	Antigen_His	0.3331		1	A2
13	A9		13	1	A17	LOADING	Antigen_His	0.3308		1	B2

Figure 5-89: Average by Sensor Tab

- **Average by Cycles tab:** contains cycle information with average nm shift and standard deviation across individual cycles:

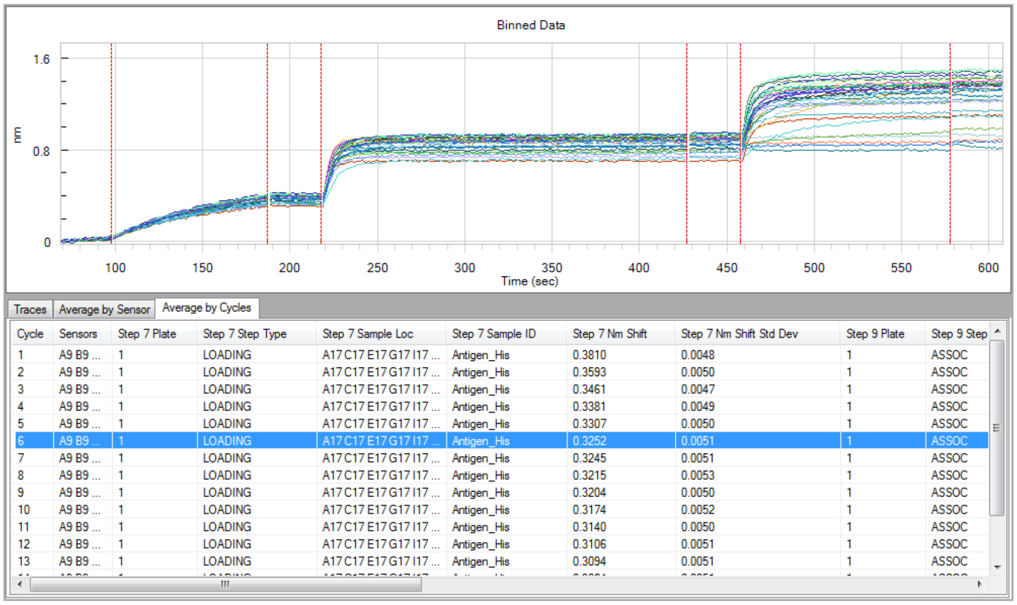

Cycle	Sensors	Step 7 Plate	Step 7 Step Type	Step 7 Sample Loc	Step 7 Sample ID	Step 7 Nm Shift	Step 7 Nm Shift Std Dev	Step 9 Plate	Step 9 Step
1	A9 B9 ...	1	LOADING	A17 C17 E17 G17 I17 ...	Antigen_His	0.3810	0.0048	1	ASSOC
2	A9 B9 ...	1	LOADING	A17 C17 E17 G17 I17 ...	Antigen_His	0.3593	0.0050	1	ASSOC
3	A9 B9 ...	1	LOADING	A17 C17 E17 G17 I17 ...	Antigen_His	0.3461	0.0047	1	ASSOC
4	A9 B9 ...	1	LOADING	A17 C17 E17 G17 I17 ...	Antigen_His	0.3381	0.0049	1	ASSOC
5	A9 B9 ...	1	LOADING	A17 C17 E17 G17 I17 ...	Antigen_His	0.3307	0.0050	1	ASSOC
6	A9 B9 ...	1	LOADING	A17 C17 E17 G17 I17 ...	Antigen_His	0.3252	0.0051	1	ASSOC
7	A9 B9 ...	1	LOADING	A17 C17 E17 G17 I17 ...	Antigen_His	0.3245	0.0051	1	ASSOC
8	A9 B9 ...	1	LOADING	A17 C17 E17 G17 I17 ...	Antigen_His	0.3215	0.0053	1	ASSOC
9	A9 B9 ...	1	LOADING	A17 C17 E17 G17 I17 ...	Antigen_His	0.3204	0.0050	1	ASSOC
10	A9 B9 ...	1	LOADING	A17 C17 E17 G17 I17 ...	Antigen_His	0.3174	0.0052	1	ASSOC
11	A9 B9 ...	1	LOADING	A17 C17 E17 G17 I17 ...	Antigen_His	0.3140	0.0050	1	ASSOC
12	A9 B9 ...	1	LOADING	A17 C17 E17 G17 I17 ...	Antigen_His	0.3106	0.0051	1	ASSOC
13	A9 B9 ...	1	LOADING	A17 C17 E17 G17 I17 ...	Antigen_His	0.3094	0.0051	1	ASSOC

***Figure 5-90:** Average by Cycles Tab*

- **Matrix tab:** contains the nm shift or normalized binding data for antibody 1 and antibody 2 binding to the antigen:

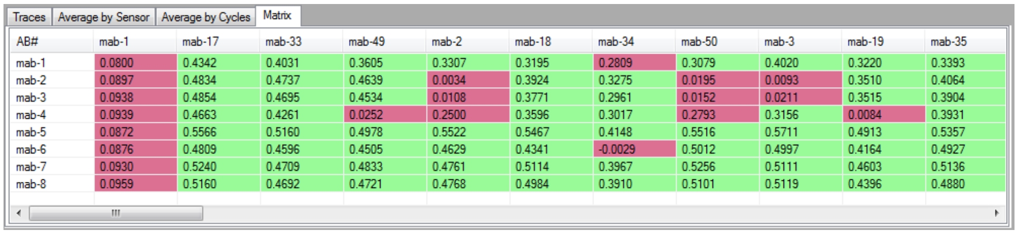

AB#	mab-1	mab-17	mab-33	mab-49	mab-2	mab-18	mab-34	mab-50	mab-3	mab-19	mab-35
mab-1	0.0800	0.4342	0.4031	0.3605	0.3307	0.3195	0.2809	0.3079	0.4020	0.3220	0.3393
mab-2	0.0897	0.4834	0.4737	0.4639	0.0034	0.3924	0.3275	0.0195	0.0093	0.3510	0.4064
mab-3	0.0938	0.4854	0.4695	0.4534	0.0108	0.3771	0.2961	0.0152	0.0211	0.3515	0.3904
mab-4	0.0939	0.4663	0.4261	0.0252	0.2500	0.3596	0.3017	0.2793	0.3156	0.0084	0.3931
mab-5	0.0872	0.5566	0.5160	0.4978	0.5522	0.5467	0.4148	0.5516	0.5711	0.4913	0.5357
mab-6	0.0876	0.4809	0.4596	0.4505	0.4629	0.4341	-0.0029	0.5012	0.4997	0.4164	0.4927
mab-7	0.0930	0.5240	0.4709	0.4833	0.4761	0.5114	0.3967	0.5256	0.5111	0.4603	0.5136
mab-8	0.0959	0.5160	0.4692	0.4721	0.4768	0.4984	0.3910	0.5101	0.5119	0.4396	0.4880

Figure 5-91: *Matrix Tab*

APPENDIX A:

Using Octet384 Systems with an Automation Interface

Automation Interface Overview 166

Design of the Automation Interface 166

AUTOMATION INTERFACE OVERVIEW

The Octet System Data Acquisition software provides support for an automation interface using a COM port (RS-232) or a Transmission Control Protocol/Internet Protocol (TCP/IP) socket/port (Figure A-1).

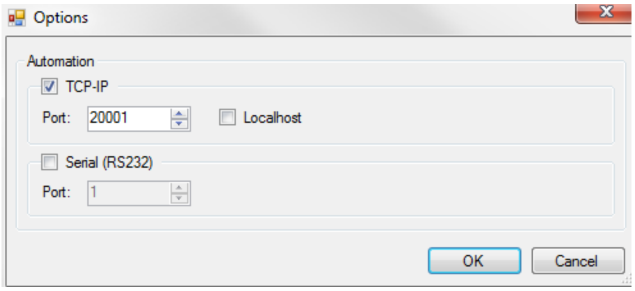

Figure A-1: *Options for Automation*

An example application for testing the automation interface (**AutomationClient.exe**) is included in the applications and Dynamic Link Libraries (DLLs) installed with the Octet System Data Acquisition software. The file is located in the **C:\Program Files\ForteBio\DataAnalysis** directory.

NOTES: *The automation interface can be used with Octet384 systems only. The examples that follow are illustrated using a TCP/IP connection, but the serial port connection behaves identically.*

DESIGN OF THE AUTOMATION INTERFACE

The automation interface is designed to be as universal as possible, making no assumptions about the communication medium or the language of the client application connecting to the Octet System Data Acquisition software.

The following guidelines apply:

- All commands and responses are ASCII strings, one per line.
- All lines are terminated with both carriage-return and line-feed characters ("\r\n").
- Each command starts with the name of the command and may then be followed by required and optional parameters.
- Each parameter starts with a switch definition (a la dos/unix command line) followed by the parameter itself, which allows parameters to be sent in any order.
- The command or response is terminated with a new line (CR/LF) sequence.
- Parameters containing embedded spaces need to be enclosed in double quotes.

Automation Interface Control Setup

Before the Octet System Data Acquisition software can be controlled using an automation interface, the correct automation options must be set. To do this, go to **File** > **Options** and select the appropriate port in the **Automation** box (Figure A-1).

NOTE: *The Octet System Data Acquisition software can be controlled via the Automation interface through a serial port (RS-232) or a TCP/IP socket.*

NOTE: *The* ***Localhost*** *option can be useful in developing the automation client on the same computer that runs the Octet System Data Acquisition software.*

NOTE: *Pall ForteBio recommends that the* ***Data File*** *repositories be set using shared folders addressed by "UNC" folder names so that the internal path used by the Data Analysis application corresponds to the external path used to access/retrieve the data files recorded during the experiment. Alternatively, the path returned by the* ***GetRunInfo*** *command to access the data files from another computer on the LAN.*

Analysis Automation API

```
//
**************************************************************************
//
//    Copyright (c) 2011 ForteBio.
//    All rights reserved.
//
//
**************************************************************************
// HEADER:  AutomationAPI.h
// PURPOSE: Defines the commands supported by the automation API.
// AUTHOR:  BHI  Nov 2008
//
#ifndef INC_ANALYSIS_AUTOMATIONAPI_H
#define INC_ANALYSIS_AUTOMATIONAPI_H

// NOTES:
// * The automation interface is string based. Commands and responses are
// strings, one per line.
```

```
// * Each command starts with the name of the command and may then be
// followed by required and
//   optional parameters.
// * Each parameter starts with a switch definition (a la dos/unix command
// line) followed by the
//   parameter itself. This allows parameters to be sent in any order.
// * The command or response is terminated with a new line (CR/LF)
sequence.
// * Parameters containing embedded spaces must be enclosed in double
// quotes.
// * Response items containing embedded spaces will be enclosed in double
// quotes.

// Version of thew API described in this header file.
const char AUT_API_VERSION[] = "1.0";

// Status return values
const char AUT_OK[]           = "OK";
const char AUT_RUNNING[]      = "Running";
const char AUT_ERROR[]        = "ERROR";
const char AUT_BUSY[]         = "Busy";
const char AUT_STOPPED[]      = "Stopped"; // Stopped by user.
const char AUT_EOL[]          = "\r\n";

// Parameter switches for the LOAD command
const char AUT_SWITCH_DATASET    = 'd';

// Parameter switches for the ANALYZE command
const char AUT_SWITCH_PARAMS     = 'p';
const char AUT_SWITCH_XMLINFO    = 'x';

// COMMAND API
// ===========

const char AUT_CMD_VERSION[] = "Version";
// Returns the version of the app being automated, and the API version.
// Args: (none)
// Response: App product version (e.g. "6.3.1.12 1.0\r\n")

const char AUT_CMD_LOAD[]     = "Load";
// Loads an experiment
// Args:
```

```
//   -d <path>      Path to experiment data files
// Response:
//   "OK\r\n"
//   "Error: <reason>\r\n"

const char AUT_CMD_ANALYZE[] = "Analyze";
// Runs an analysis
// Args:
//   -p <path>      Path to parameters (INI file)
//   -x <path>     Path to XML information file (optional, can be multiple
XML info files)
// Response:
//   "OK\r\n"
//   "Error: <reason>\r\n"

const char AUT_CMD_STATUS[]  = "Status";
// Returns status: OK=ready, Busy=running, Error=Action was terminated by
an error.
// Busy is followed by descriptive information on the progress of the
experiment (% complete)
// Args: (none)
// Response:
//   "OK\r\n"
//   "Busy\r\n"
//   "Running (nn%)\r\n"
//   "Error: <reason>\r\n"

#endif // INC_ANALYSIS_AUTOMATIONAPI_H
```

APPENDIX B:

21 CFR Part 11 Software Administrator Options

Installing the Data Acquisition 21 CFR Part 11 Software 172
Installing the Data Analysis 21 CFR Part 11 Software 174
Installing the ForteBio GxP Server Module 177
Database Backup and GxP Server Module Upgrade 182
Administrator Account Setup 182
Starting an Administrator User Session 187
Accessing Administrator Options 190
Accessing the ForteBio GxP Server Module Directly 206
Restarting the Pall ForteBio GxP Server Module 209

INSTALLING THE DATA ACQUISITION 21 CFR PART 11 SOFTWARE

NOTE: *Version 8.2 of Data Acquisition and Data Analysis 21 CFR Part 11 software require a new database schema and version 8.2 of the GxP Server module. The new database schema is installed and configured during the version 8.2 software installation. Version 8.2 software will automatically check the version of the GxP Server module in use and display a message if it is incompatible.*

To install the Data Acquisition 21 CFR Part 11 software:

1. Insert the software **CFR CD** into your CD drive.
   - If the Autoplay dialog box displays, choose to open the CD to view files.
   - If the Autoplay dialog box does not display, navigate to the CD using Windows Explorer.

   Optical drives are typically found under the **D:** or **E:** drive.
2. Double-click **DataAcquisition-CFR.exe** to launch the installation wizard (see Figure B-1).

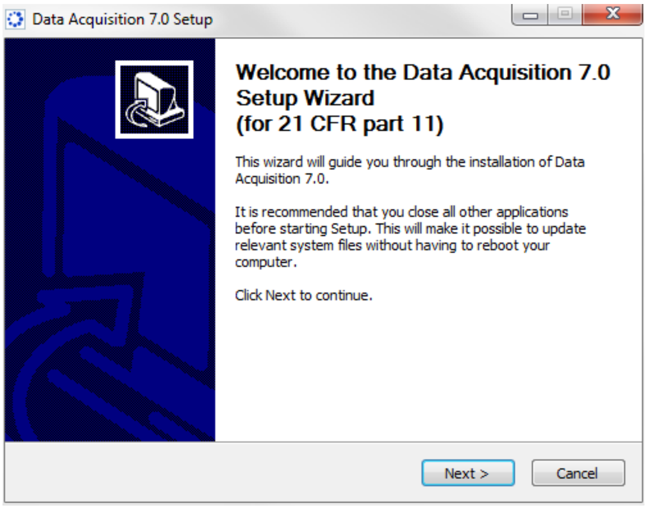

Figure B-1: *Data Acquisition (for 21 CFR Part 11) Software Setup Wizard*

3. Click **Next** to display the Choose Install Location dialog box (Figure B-2).

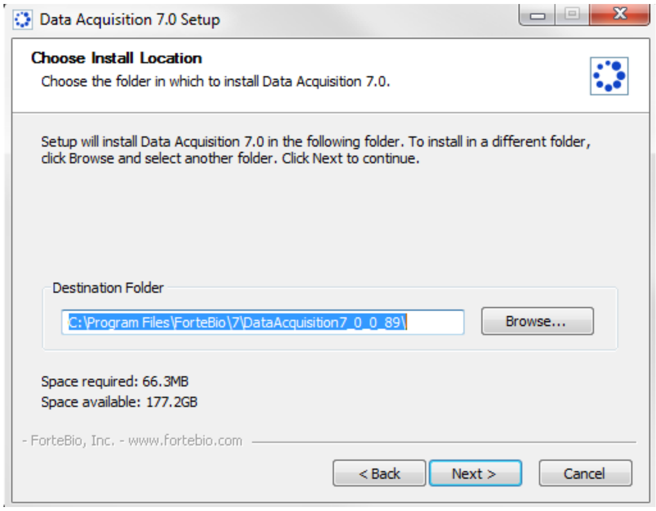

Figure B-2: Choose Install Location Dialog Box

The default location for the software on the local machine is **C:\Program Files\ForteBio\DataAcquisition**.

4. Click **Next** to accept this path location.

   The Choose Start Menu Folder dialog box displays (Figure B-3).

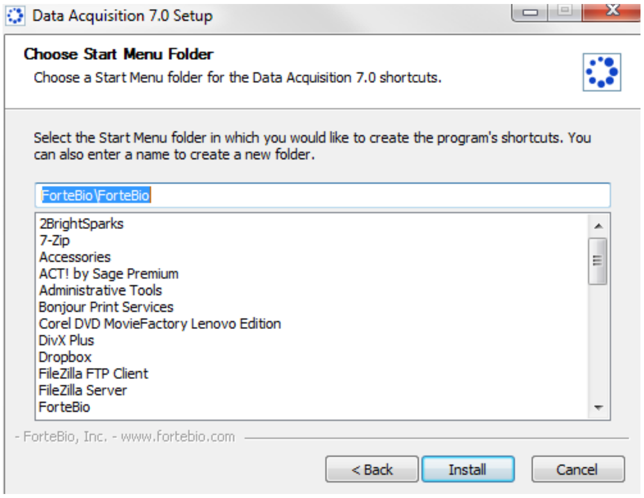

Figure B-3: Choose Start Menu Folder Dialog Box

The default Start Menu folder is **ForteBio**.

5. Click **Install**.

   The installation wizard takes a few seconds to install.

   When the installation is complete, the installation wizard displays the Completing the Data Acquisition Setup Wizard dialog box (Figure B-4).

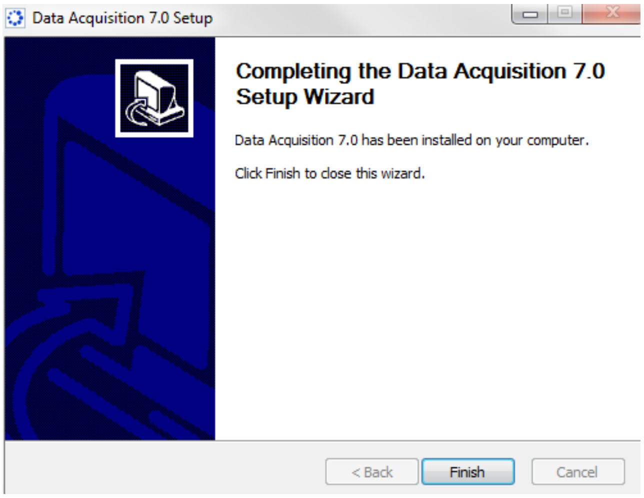

Figure B-4: *Completing the Data Analysis Setup*

6. Click **Finish** to complete the installation.

INSTALLING THE DATA ANALYSIS 21 CFR PART 11 SOFTWARE

NOTE: *Version 8.2 of Data Acquisition and Data Analysis 21 CFR Part 11 software require a new database schema and version 8.2 of the GxP Server module. The new database schema is installed and configured during the version 8.2 software installation. Version 8.2 software will automatically check the version of the GxP Server module in use and display a message if it is incompatible.*

To install the Data Analysis 21 CFR Part 11 software:

1. Insert the software CD into your CD drive.
2. Navigate to the window listing the files located on the installation CD.
3. Double-click **DataAnalysis-CFR.exe** to launch the installation wizard (see Figure B-5).

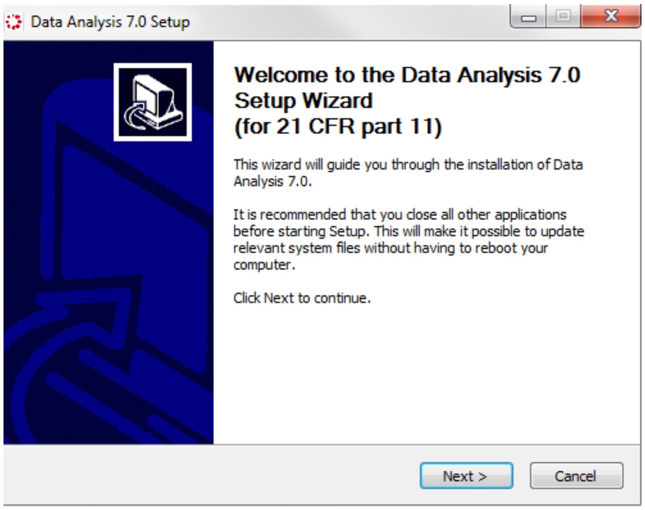

Figure B-5: *Data Analysis (for 21 CFR Part 11) Software Setup Wizard*

4. Click **Next** to display the Choose Install Location dialog box (Figure B-6).

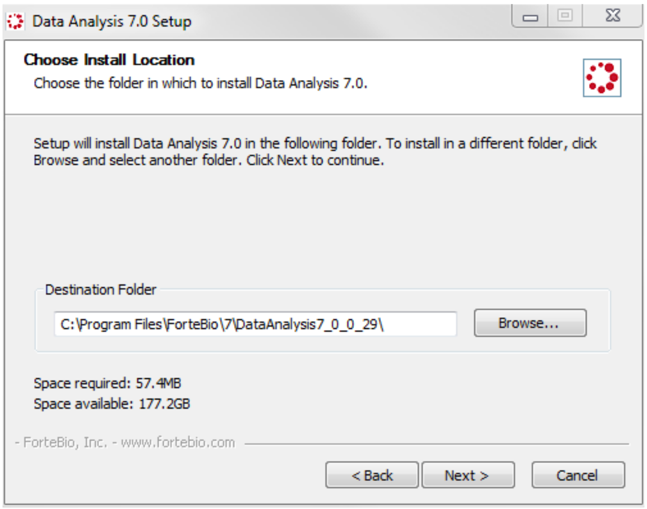

Figure B-6: *Choose Install Location Dialog Box*

The default location for the software on the local machine is **C:\Program Files\ForteBio\DataAnalysis**.

5. Click **Next** to accept this path location.

   The Choose Start Menu Folder dialog box displays (Figure B-7).

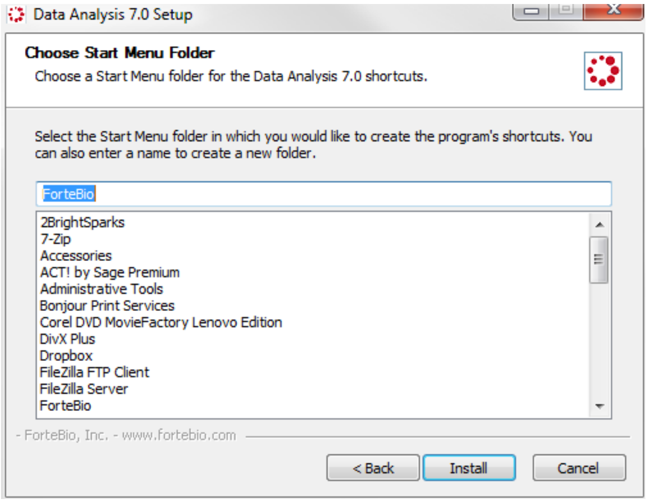

Figure B-7: Choose Start Menu Folder Dialog Box

The default Start Menu folder is **ForteBio**.

6. Click **Install**.

The installation wizard takes a few seconds to install (Figure B-8).

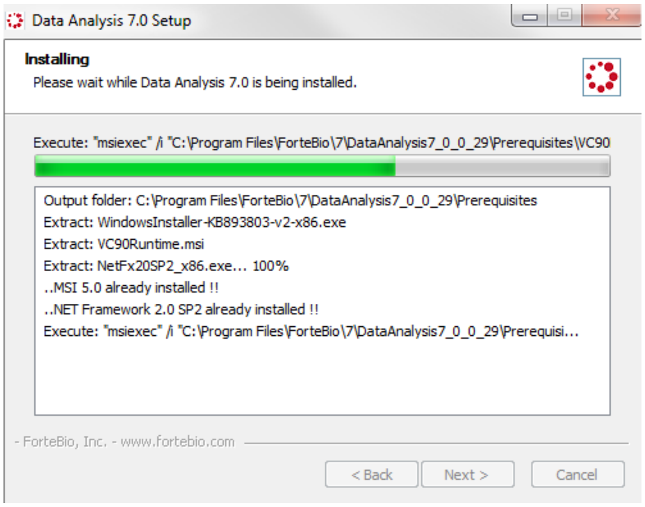

Figure B-8: Installation Progress

The installation wizard displays the Completing the Data Analysis Setup Wizard dialog box (Figure B-9).

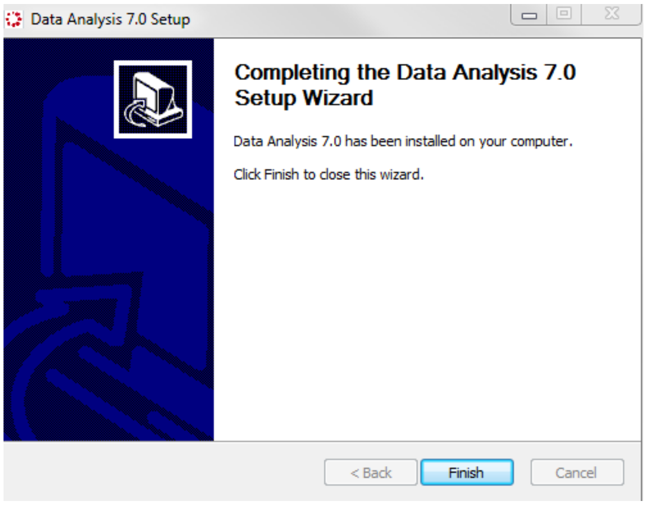

Figure B-9: *Completing the Data Analysis Setup*

7. Click **Finish** to complete the installation.

INSTALLING THE FORTEBIO GXP SERVER MODULE

NOTE*: It is highly recommended that the ForteBio GxP Server Module not be installed on the computer connected to the Octet instrument. Instead, it should be configured on the administrator's computer that is connected to the server. This allows the administrator to control the users who have accounts and specific privileges in the 21 CFR Part 11 Data Acquisition software. Both the Octet system computer and the computer with the ForteBio GxP Server Module should be connected to the server.*

The ForteBio GxP Server Module contains FBServer Config and FBServer Monitor (see Figure B-10). User accounts should be made in the FBServer Config with the required permissions. It is highly recommended that at least two users have administrative access to the FBServer Config. Once the accounts are made, FBServer Config is password protected so only users with administrative rights can access it. FBServer Monitor may be copied to the computer that is connected to the Octet instrument to monitor the audit trail (see Figure B-10).

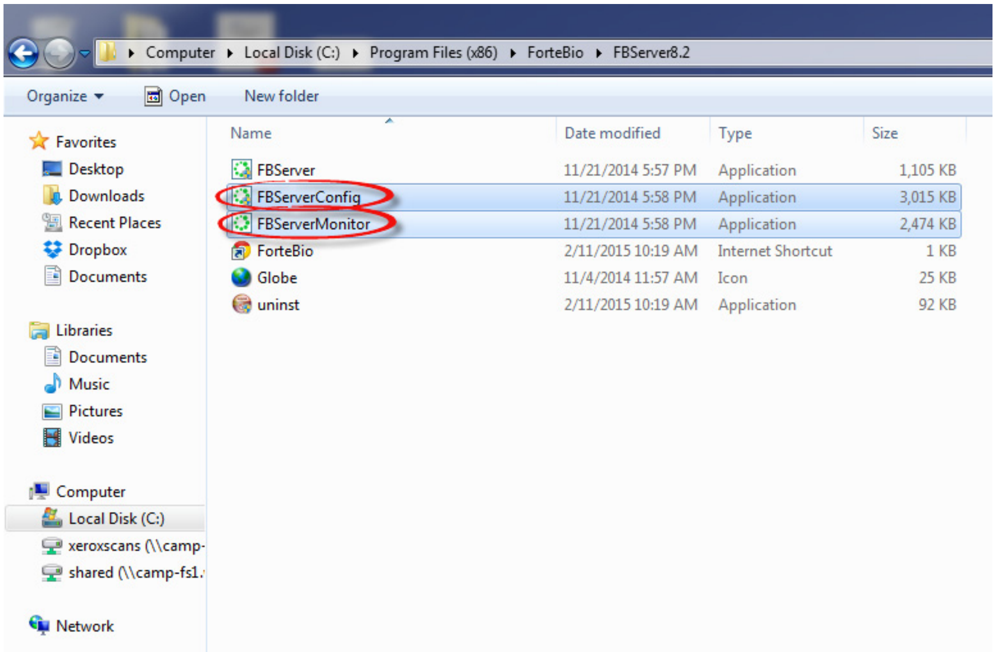

Figure B-10: *FBServer Config and FBServer Monitor on Local Disk in Windows Explorer.*

The ForteBio GxP Server module can be installed and run from the following locations:

- **Recommended:** A remote host computer networked to a machine where the Data Acquisition or Data Analysis 21 CFR Part 11 software is installed
- A local host computer where the Data Acquisition or Data Analysis 21 CFR Part 11 software is installed

Upon launching the Octet System Data Acquisition or Data Analysis CFR 11 software, you are required to select the GxP Server module host location. If the GxP Server module is installed in multiple locations, you can select any host server. The user session event record will be saved only to the host location selected, making it possible to have records for the same user in multiple locations.

NOTES: For administrators only.
To ensure that all records are saved to one location, Pall ForteBio recommends that one administrator install a single copy of the ForteBio GxP Server module on the administrator's computer that is connected to the network. For added security, the ForteBio GxP Server module should not be accessible to all users.

Version 8.2 of Data Acquisition and Data Analysis 21 CFR Part 11 software require version 8.2 of the GxP Server module. The software will automatically check the version of the GxP Server module in use and display a message if it is incompatible.

To install the ForteBio GxP Server software:

1. Navigate to the window listing the files located on the installation CD.
2. Double-click **ForteBio GxP Server.exe** to launch the installer.
3. If prompted with the *Do you want the following program from an unknown publisher to make changes to this computer?* message, reply **Yes**.

   The installation wizard should display (Figure B-11).

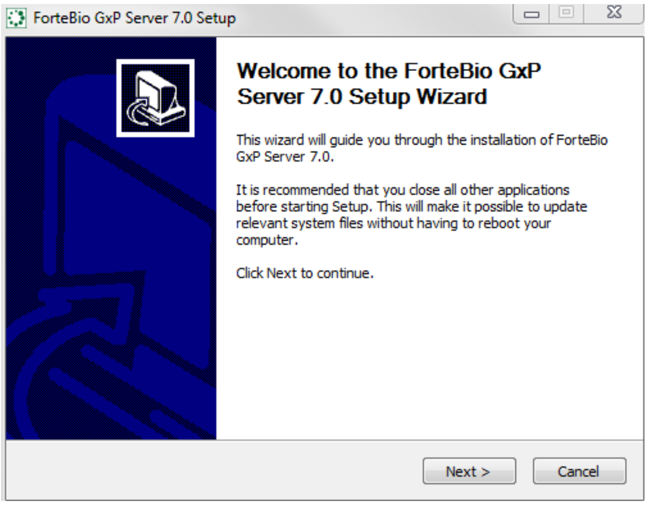

Figure B-11: *ForteBio GxP Server Software Setup Wizard*

4. Click **Next** to display the Choose Install Location dialog box (Figure B-12).

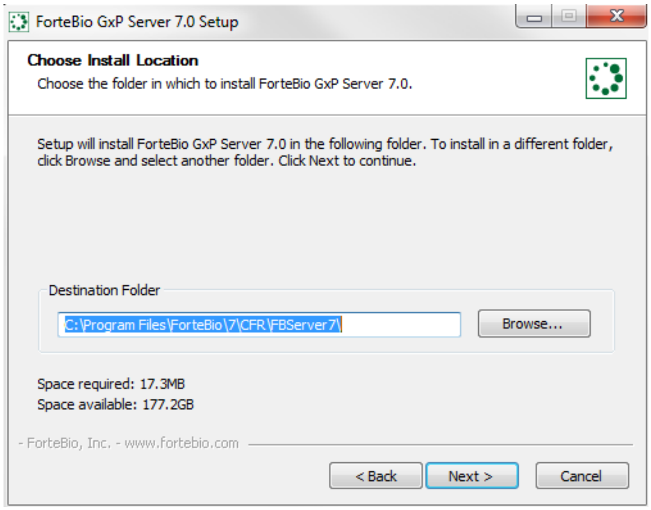

Figure B-12: Choose Install Location

The default location for the software on the local machine is **C:\Program Files\ForteBio\DataAnalysis**.

5. Click **Next** to accept this path location.

   The Choose Start Menu Folder dialog box displays (Figure B-13).

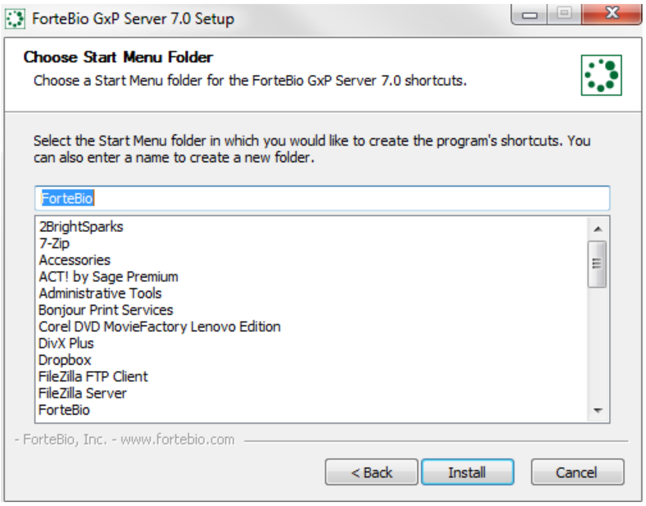

Figure B-13: Choose Start Menu Folder Dialog Box

The default Start Menu folder is **ForteBio**.

6. Click **Install**.

   The installation wizard takes a few seconds to install (Figure B-14).

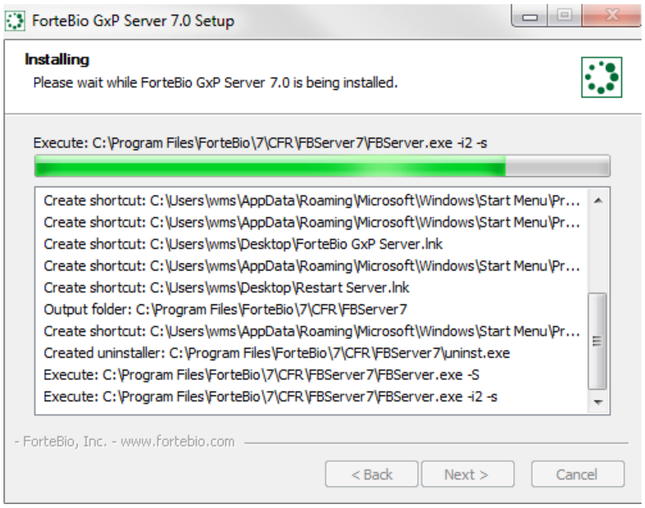

Figure B-14: *Installation Progress*

The installation wizard displays the Completing the ForteBio GxP Server Setup Wizard dialog box (Figure B-15).

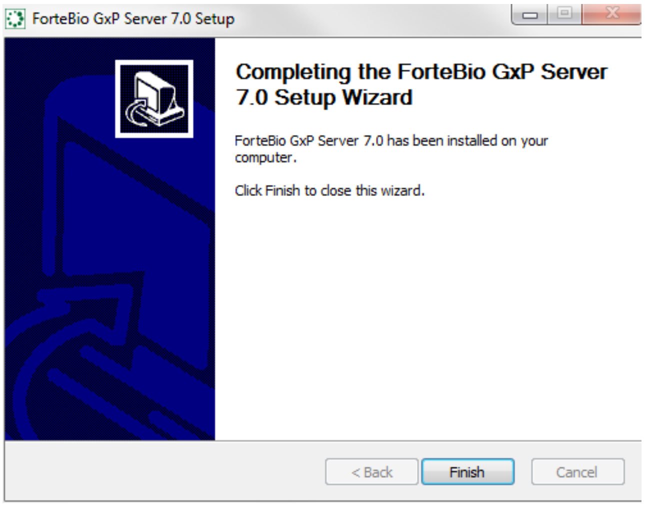

Figure B-15: *Completing the ForteBio GxP Server Software Setup*

7. Click **Finish** to complete the installation.

DATABASE BACKUP AND GXP SERVER MODULE UPGRADE

***IMPORTANT**: It is strongly recommended that you make a backup copy of the existing database before installing and upgrading to GxP FBServer Module v8.2.*

To backup your database and then upgrade from GxP FBServer Module versions 7 or 8.0 to version 8.2, follow the steps below. Once these steps are complete, the existing audit trail database that stores audit logs will be upgraded to the version 8.2 schema.

Step 1: Backup the Version 8.0 Database

The database for the GxP FBServer Module is a file-based database. So to make a backup of the database, all you need to do is make a copy of the database file and save it to an archival location.

1. Log on to the computer that is hosting the GxP FBServer Module v7 or v8.0.
2. Open Windows® Explorer and browse to the program data folder (for example: C:\ProgramData\ForteBio\FBServer)
3. Make a copy of the FBEventLog.db and FBServer.db files.
4. Save the copies to another location.

Step 2: Upgrade to GxP FBServer Module v8.2

1. From the Windows Start Menu, select **All Programs**. Scroll to the ForteBio FBServer folder and click on the folder to expand.
2. Select **Uninstall ForteBio GxP Server**.

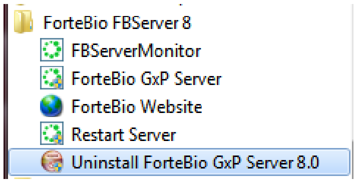

3. Install GxP FBServer Module v8.2 per the instructions in "Installing the ForteBio GxP Server Module" on page 177

   When the GxP FBServer Module v8.2 software starts, it will automatically update the audit trail database.

ADMINISTRATOR ACCOUNT SETUP

To set up the administrator account:

1. Launch the Octet System Data Acquisition or Data Analysis software by double-clicking the respective desktop icon; see Figure B-16.

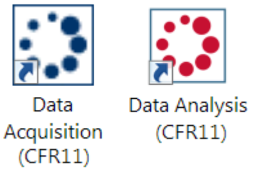

Figure B-16: *Data Acquisition or Data Analysis Software Desktop Icons*

The **Login** dialog box displays (Figure B-17).

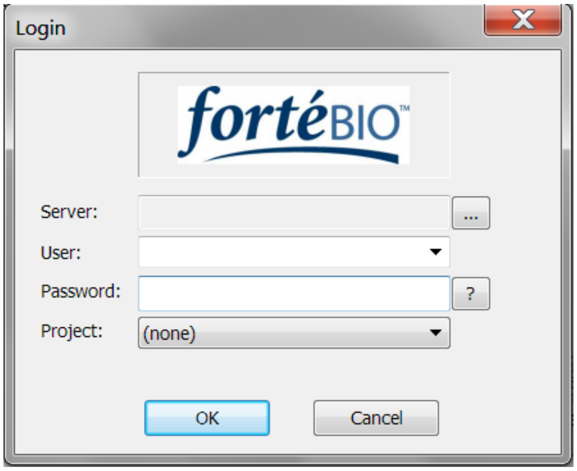

Figure B-17: *Login Dialog Box*

2. Select a **Server** location by clicking **...** (browse).

   The **Authentication Server** dialog box displays (Figure B-18).

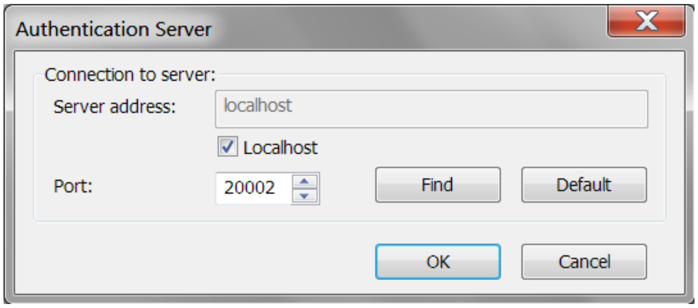

Figure B-18: *Authentication Server*

3. Click **Default** to recall the default server settings of localhost and Port 2002.

- ***Local host***—If the local computer is to be used as the GxP Server module host, click the **Localhost** check box. Change the Port number if needed.
- ***Remote host on same subnet***—If the GxP Server module is hosted on the same subnet, deselect the **Localhost** check box and click **Find**. A list of potential GxP Server module addresses will be listed. Choose the desired location from the list and click **OK**.

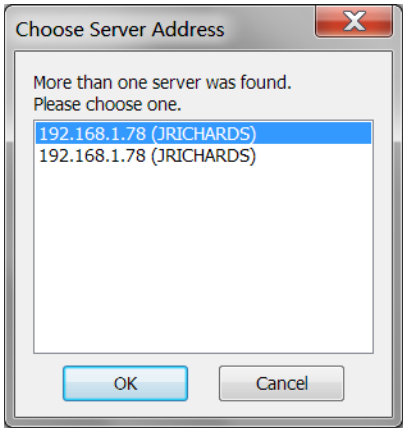

Figure B-19: Choose Server Address

- ***Remote host on another subnet***— If the GxP Server module is hosted on a different subnet, deselect the **Localhost** check box. Enter the IP address of the computer hosting the GxP Server module.

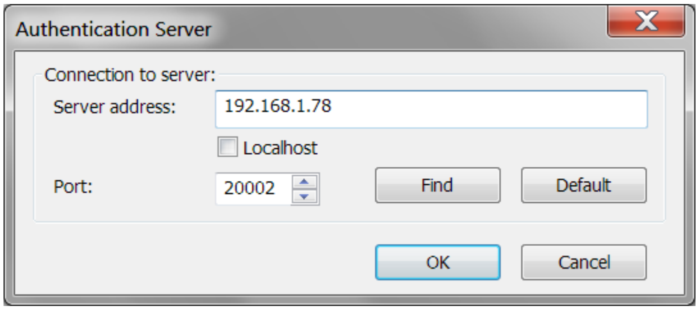

Figure B-20: Authentication Server

When the GxP Server module host location has been selected or entered, click **OK** to save changes and exit the **Authentication Server** dialog box. The GxP Server module location will now be listed as the **Server** in the **Login** dialog box.

NOTE: *Once the GxP Server module host location is selected, this location will be used as the default selection for the administrator account. It does not need to be reselected each time a new session is initiated.*

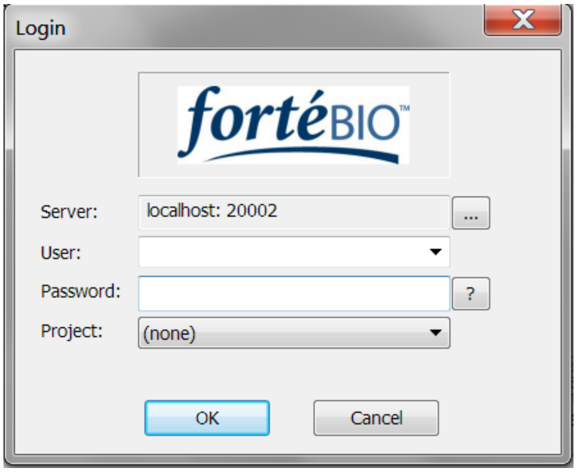

Figure B-21: Login Dialog Box with Server Parameter Configured

4. Select **Administrator** from the **User** drop-down list (Figure B-21).
5. Leave the **Password** blank, set the **Project** to **(none)** and click **OK** (Figure B-22).

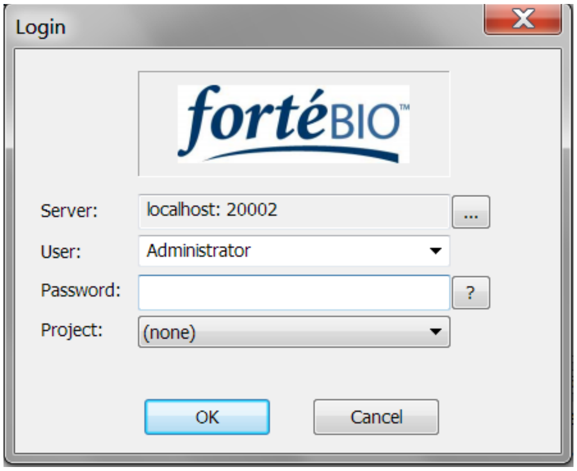

Figure B-22: Login Dialog Box with Server, User, and Project Settings Configured

The **Change Password** dialog box displays; see Figure B-23.

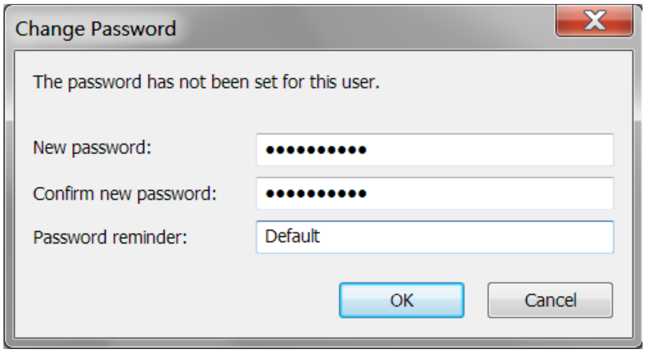

Figure B-23: *Change Password Dialog Box*

6. Enter a **New password** and **Password reminder** (optional) and click **OK**.

   The Octet System Data Acquisition or Data Analysis software launches and initiates an administrator user session that will allow access to administration options.

STARTING AN ADMINISTRATOR USER SESSION

Administrators initiate new user sessions the same way non-administrative users do.

1. Launch the Octet System Data Acquisition or Data Analysis software by double-clicking the respective desktop icon; see Figure B-24.

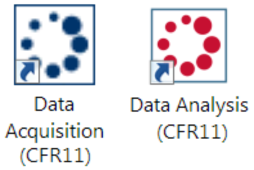

Figure B-24: *Data Acquisition or Data Analysis Software Desktop Icons*

The **Login** dialog box displays; see Figure B-25.

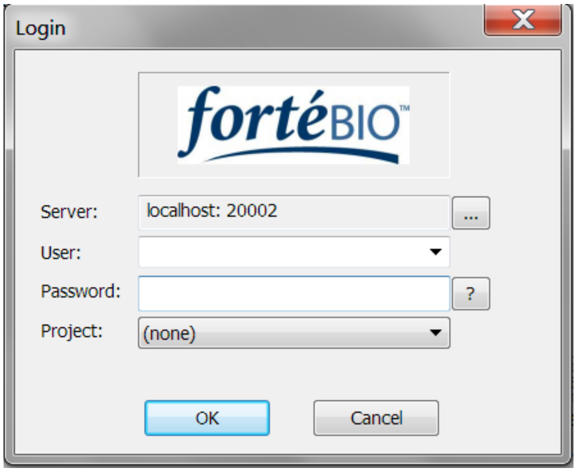

Figure B-25: *Login Dialog Box*

2. Confirm that the **Server** location is correct. If not, see “Administrator Account Setup” on page 182.
3. Select **Administrator** from the **User** drop-down list (Figure B-26).

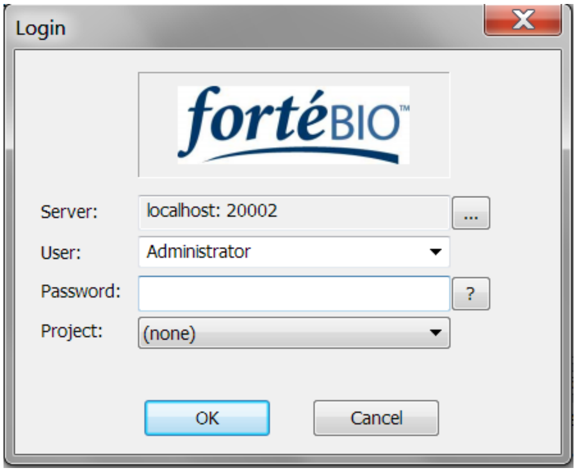

Figure B-26: *Login Dialog Box with Server, User, and Project Settings Configured*

4. Enter your **Password**. Click **?** for a password reminder (Figure B-27) if necessary.

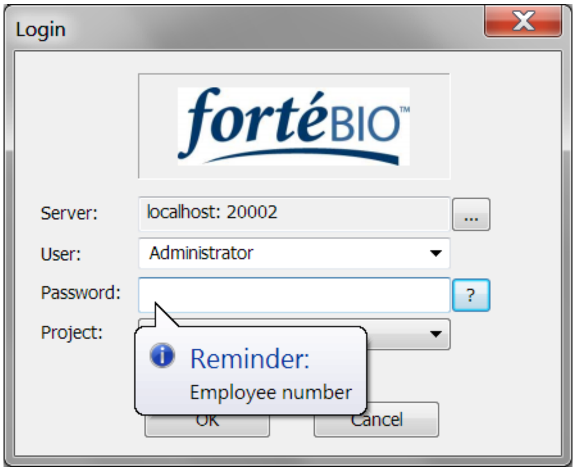

Figure B-27: *Password Reminder*

5. If required, select a project from the **Project** drop-down list (Figure B-28).

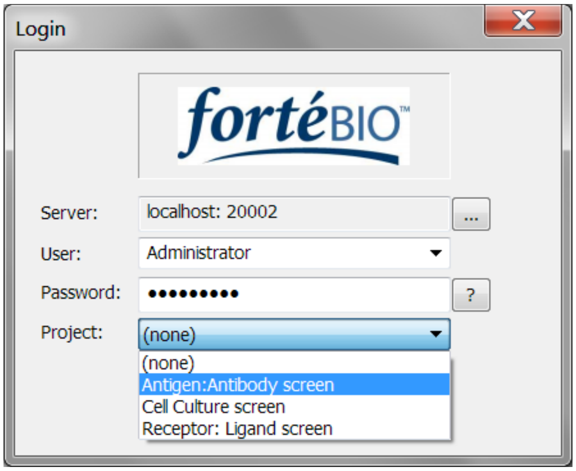

Figure B-28: *Login Dialog Box with Project Selections*

6. Click **OK**.

   The Octet System Data Acquisition or Data Analysis software launches and initiates the administrator session. During the session, the administrator account and project selected at login are displayed in the Data Acquisition software status bar.

NOTE: *Administrator and user sessions are automatically closed after a period of inactivity set using the* ***UserIdleMin*** *constant. Please see "Administrator Constants" on page 201 for more information.*

ACCESSING ADMINISTRATOR OPTIONS

The 21 CFR Part 11 software Server Administration options allow administrators to mange users, groups, projects and constants and view associated events.

These options can be accessed in the Octet System Data Acquisition and Data Analysis software or by launching the ForteBio GxP Server module directly.

- ***Data Acquisition and Data Analysis software***—Click **Security** > **Server Administration** (Figure B-29).

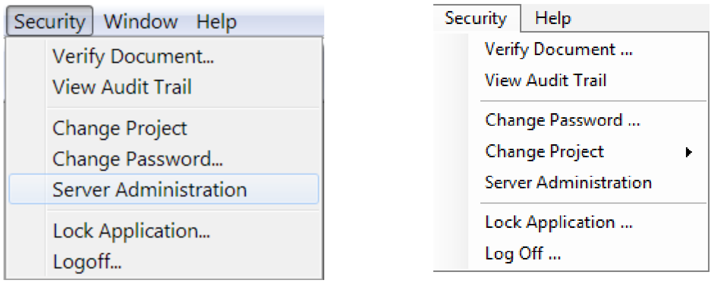

Data Acquisition Software   Data Analysis Software

Figure B-29: *Security > Server Administration Menu*

- ***ForteBio GxP Server module on network location—***Double-click the **FBServerConfig.exe** file in the FBServer folder from the installed location (Figure B-30).

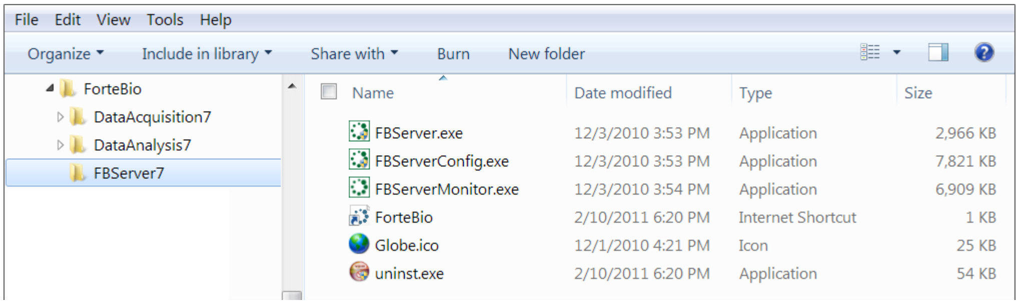

Figure B-30: *Installation Location*

- ***ForteBio GxP Server module on a local host computer***—Double-click the ForteBio GxP Server desktop icon (Figure B-31).

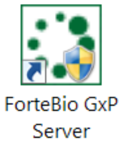

Figure B-31: *ForteBio GxP Server Desktop Icon*

NOTES:

When accessing the ForteBio GxP Server module directly, additional tools are also provided to test server functionality. Please see “Accessing the ForteBio GxP Server Module Directly” on page 206 for more information.

After the initial setup of the ForteBio GxP Server and the formation of user accounts, the ForteBio GxP Server will be password protected and only administrator(s) can access it.

The **ForteBio GxP Server Configuration** window displays.

Administrator Tabs

Five tabs are available in the **ForteBio GxP Server Configuration** window:

- **Users**—Allows user and password management and individual privileges selection.
- **Groups**—Allows user group management and group privileges selection.
- **Projects—**Allows project management and setup.
- **Constants**—Allows setup of GxP server parameters.
- **Events**—Displays event logs for individual user accounts, projects, or machines.

Click any of the tabs to view the respective information contained within the tab.

Tab View

Each tab displays a list of administrator entries and associated setting information that can be sorted by clicking any of the column headers (Figure B-32).

Login Name	Full Name	Group	Privileges	Password Age	Info
GMoreno	George Moreno	Guest	run	00:11:23	External collaborator
RBrown	Richard Brown	Supervisor	review	00:12:32	
JBlack	John Black	Developer	change, plate, run	00:13:30	
PSmith	Paul Smith	Lab User	run	00:14:46	
Administrator	Administrator	Administrator	admin, review, change, plate	1 day 22:17:19	Default administrator user

Figure B-32: *Tab View Example*

Tab Menu

Right-clicking an entry or a blank area in the tab displays the tab menu. Tab menu options vary depending on the tab selected.

User Account Administration

The **Users** tab allows administrators to add and inactivate user accounts, as well as set and change individual user account privileges and passwords.

Creating a New User Account

To create a new user account:

1. Right-click anywhere in the **Users** tab and select **New User**, or double-click in a blank area; see Figure B-33.

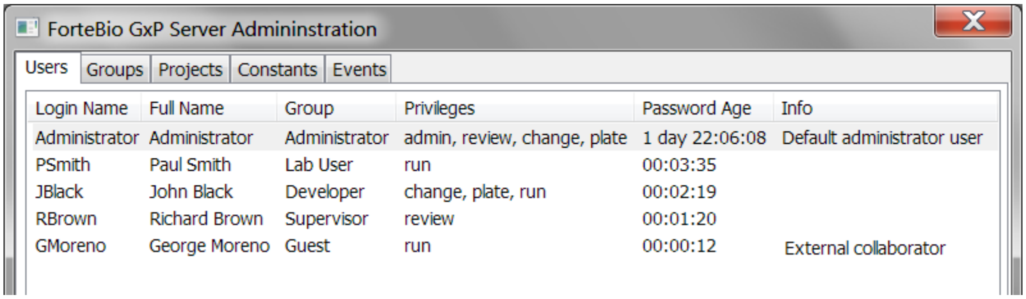

Figure B-33: *New User Menu*

The **New User** dialog box displays (Figure B-34).

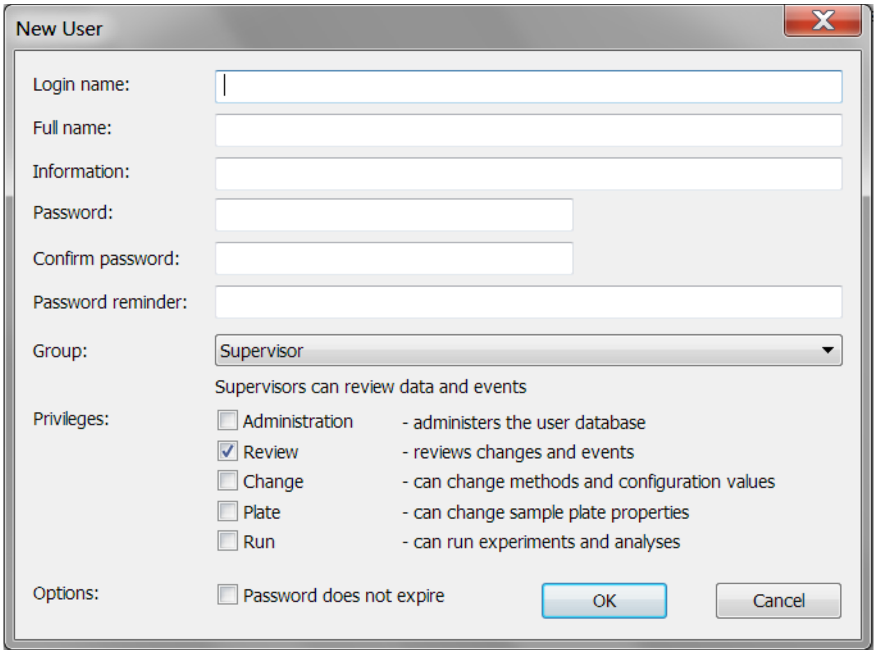

Figure B-34: *New User Dialog Box*

2. ***Assign Account Details.*** Enter the user's **Login name**, **Full name**, **Information** (optional), **Password**, and **Password reminder** (optional).
3. ***Assign a User Group.*** Select a user group from the **Group** drop-down list. The following default group selections are available:
   - **Administrator**—Add, inactivate, and change user accounts and groups.
   - **Supervisor**—Review data and events.
   - **Developer**—Create, run, save, and export data.
   - **Lab User**—Only run experiments.
   - **Guest**—No explicit privileges; these must be assigned by the administrator.

If other user groups have been created by an administrator, they will also be available for selection in the **Group** drop down box. For more information, please see "Creating a New User Group" on page 198.

4. ***Assign Privileges.*** Each user account can be assigned specific privileges. The privileges displayed initially will be those defined in the user group selected in the previous step. Table B-1 outlines the privileges for the default user groups. If needed, change user account privileges by selecting or deselecting the check boxes next to each privileges.
   - **Administration—**Can administer the user database.
   - **Review—**Can review changes and events.
   - **Change—**Can change methods and configuration values.
   - **Plate—**Can change sample plate properties.
   - **Run—**Can run experiments and analyses.

Table B-1: *Default user group privileges.*

Privilege	*Administrator*	*Supervisor*	*Developer*	*Lab User*	*Guest*
Administration	✓				
Review	✓	✓			
Change	✓		✓		
Plate	✓		✓		
Run			✓	✓	

5. ***Options***—Click the **Password does not expire** check box if desired. By default, this check box is not selected. Clicking this option will let user account passwords expire at the set PasswordTTL constant. For more information on setting constants, see “Administrator Constants” on page 201.
6. Click **OK** to save changes and exit.

Viewing and Changing User Account Settings

To view and change user account settings:

1. On the **Users** tab, right-click the user account and select **Edit User**, or double-click the user account.

   The **Edit User** window displays (Figure B-35).

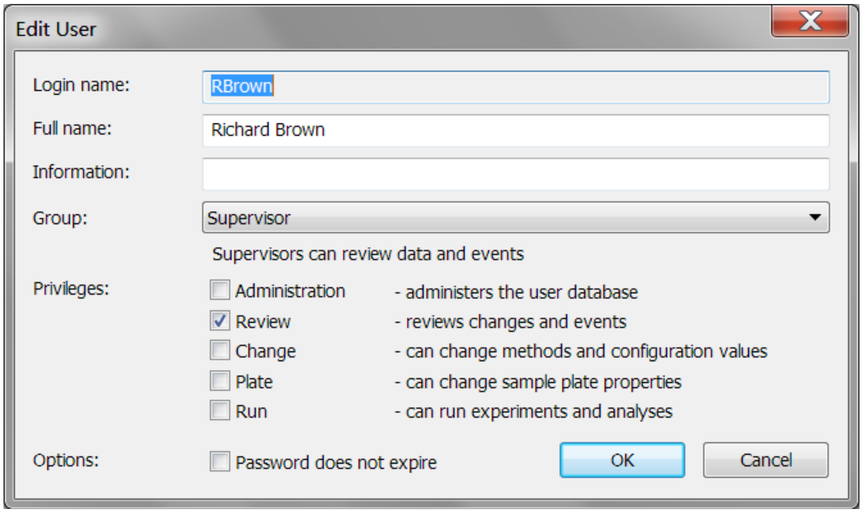

Figure B-35: *Edit User Dialog Box*

2. If needed, modify the user account settings. For more details on individual settings, see "Creating a New User Account" on page 192.
3. Click **OK** to save changes and exit.

Inactivating User Accounts

To inactivate a user account:

1. On the **Users** tab, right-click the user account and select **Inactivate User**.
2. Click **OK** in the dialog box displayed.

NOTE: Users can only be inactivated, not deleted, from the GxP server.

Changing User Account Passwords

To change a user account password:

1. On the **Users** tab, right-click the user account and select **Set Password**.

   The **Change Password** dialog box displays (Figure B-36).

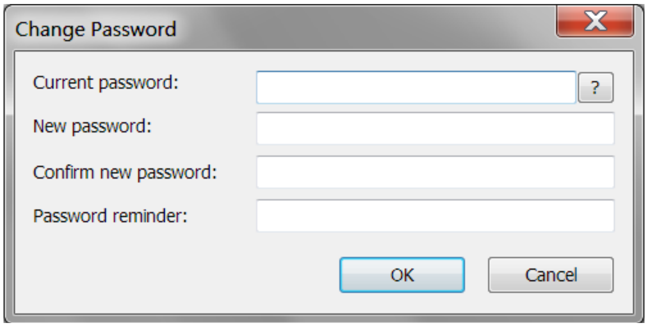

Figure B-36: *Change Password Dialog Box*

2. Enter the **New Password**, confirm the new password, and provide a **Password reminder** (optional).
3. Click **OK** to save changes and exit.

Changing the Administrator Password

To change the administrator password:

1. Initiate a new administrator user session.
2. When the software launches, on the main menu, click **Security** > **Change Password**.

   The **Change Password** window displays (Figure B-37).

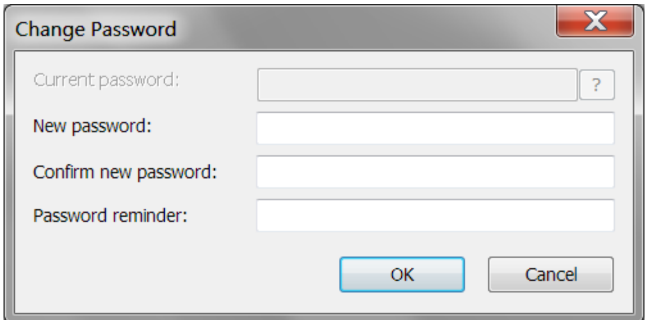

Figure B-37: *Change Password Dialog Box (Administrator)*

> ***NOTE**: You can also access the **Change Password** dialog box by right-clicking on the administrator account in the **Users** tab and selecting **Set Password** from the tab menu.*

3. Enter the **Current password** for your user account. Click **?** for a password reminder.
4. Enter the **New Password** and **Password reminder** (optional).
5. Click **OK** to save changes and exit.

Group Administration

The **Groups** tab (Figure B-38) allows administrators to add and inactivate user groups as well as set and change group privileges.

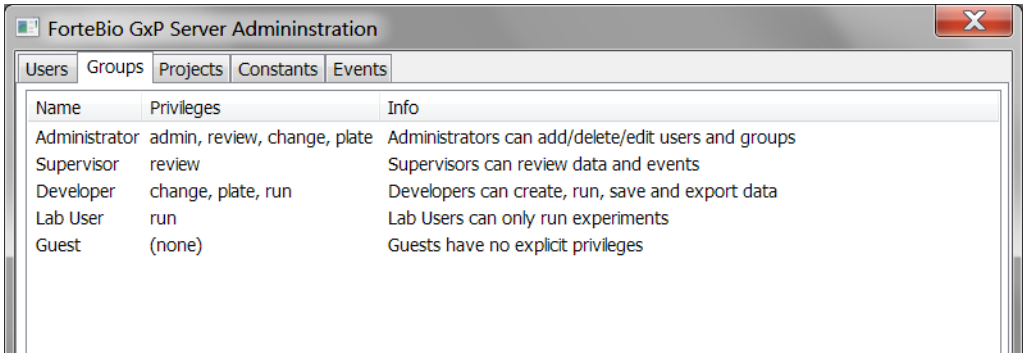

***Figure B-38:** ForteBio GxP Server Administration*

When a user account is assigned to a user group, the privileges defined in the group are also applied to the individual user account. The following default user groups are available and the privileges assigned to each are shown Table B-2:

- **Administrators**—Can add, inactivate and change user accounts and groups
- **Supervisors**—Can review data and events
- **Developers**—Can create, run, save and export data
- **Lab Users**—Can only run experiments
- **Guests**—Have no explicit privileges, these must be assigned by the administrator

Table B-2: *Default User Group Privileges*

Privilege	*Administrator*	*Supervisor*	*Developer*	*Lab User*	*Guest*
Administration					
Review	✓	✓			
Change	✓		✓		
Plate	✓		✓		
Run			✓	✓	

Creating a New User Group

To create a new user group:

1. Right-click anywhere in the **Groups** tab and select **New Group** or double-click in a blank area.

   The **New Group** window displays (Figure B-39).

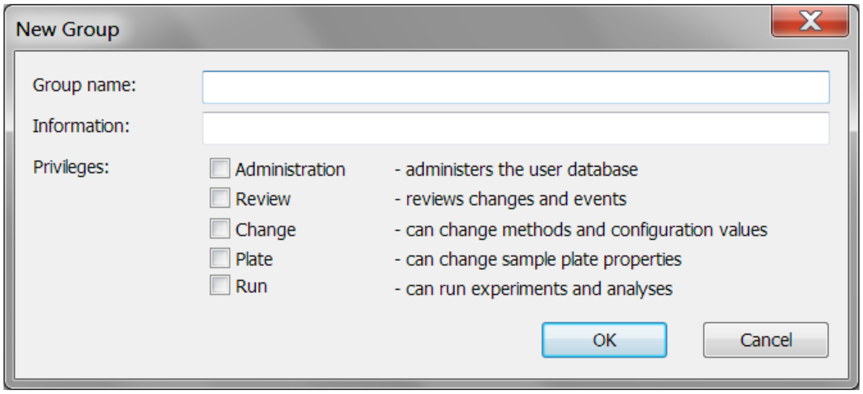

Figure B-39: *New Group Dialog Box*

2. Enter the **Group name** and **Information** (optional).
3. ***Privileges***—Each group can be assigned specific privileges. Add group privileges by selecting or deselecting the check boxes next to each privilege:
   - **Administration**—Can administer the user database
   - **Review**—Can review changes and events
   - **Change**—Can change methods and configuration values
   - **Plate**—Can change sample plate properties
   - **Run**—Can run experiments and analyses
4. Click **OK** to save changes and exit.

Viewing and Changing Group Settings

To view and change group settings:

1. Right-click on the group and select **Edit Group**, or double click the group.

   The **Edit Group** window displays (Figure B-40).

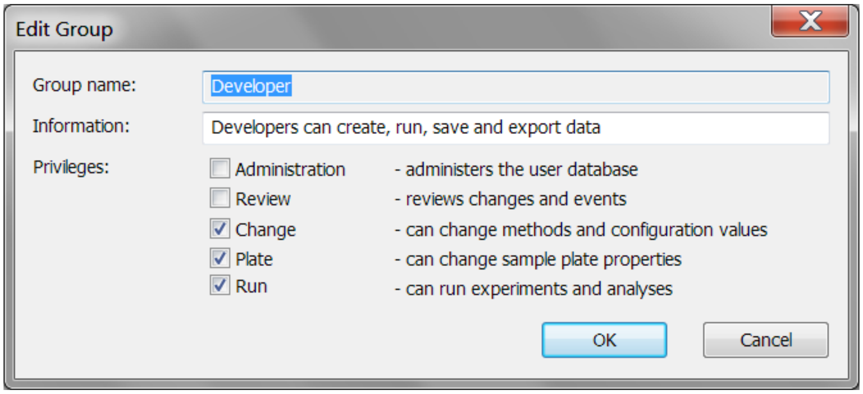

Figure B-40: *Edit Group Dialog Box*

2. If needed, modify the group settings. For more details on individual settings, see “Creating a New User Group” on page 198.
3. Click **OK** to save changes and exit.

Inactivating a User Group

To inactivate a user group:

1. Right-click the group and select **Inactivate Group**.
2. Click **OK** in the dialog box displayed.

Project Administration

The **Projects** tab (Figure B-41) allows administrators to add and delete user projects. Projects are selected when a new user session is initiated in the Octet System Data Acquisition or Data Analysis software, allowing all user, system and software events for a particular project to be monitored.

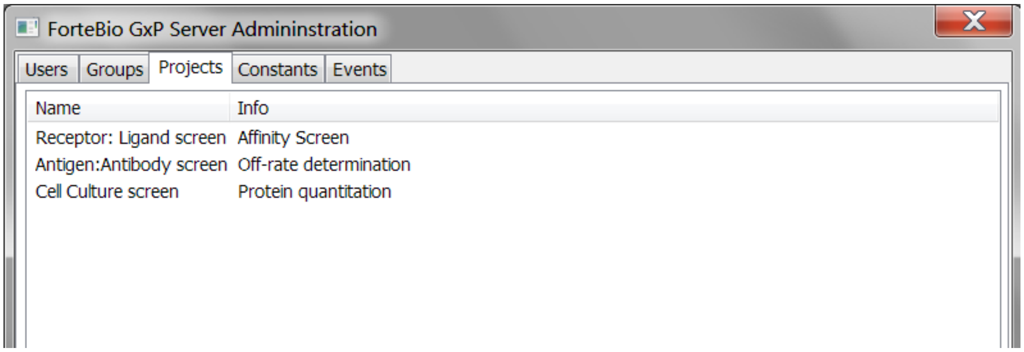

Figure B-41: *Projects*

Creating a New Project

To create a new project:

1. Right-click anywhere in the **Projects** tab and select **New Project**, or double-click in a blank area.

   The **New Project** window displays (Figure B-42).

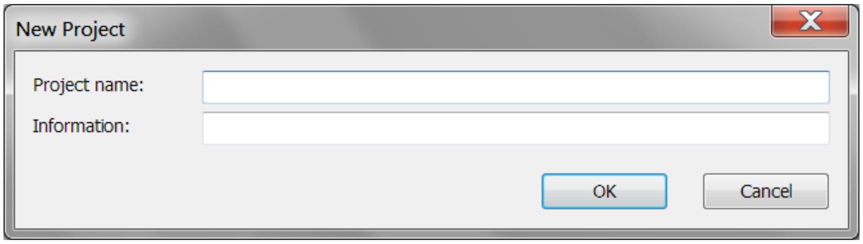

Figure B-42: *New Project*

2. Enter the **Project name** and **Information** (optional).
3. Click **OK** to save changes and exit.

Viewing and Changing Project Settings

To view and change project settings:

1. Right-click on the project and select **Edit Project**, or double-click on the project.

   The **Edit Project** window displays (Figure B-43).

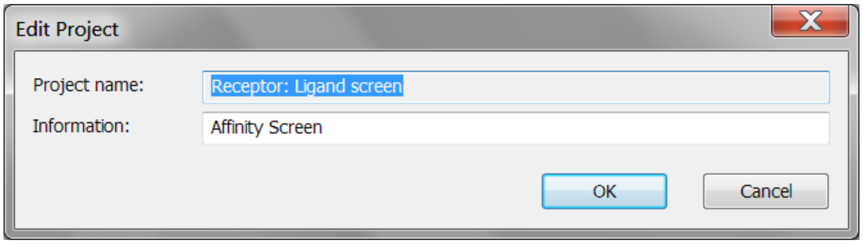

Figure B-43: *Edit Project*

2. If needed, modify the project settings.
3. Click **OK** to save changes and exit.

Deleting a Project

To delete a project;

1. Right-click the project and select **Delete Project**.
2. Click **OK** in the dialog box displayed.

Administrator Constants

The **Constants** tab allows administrators to set GxP Server module constant settings. These constants are applied to all user accounts and sessions.

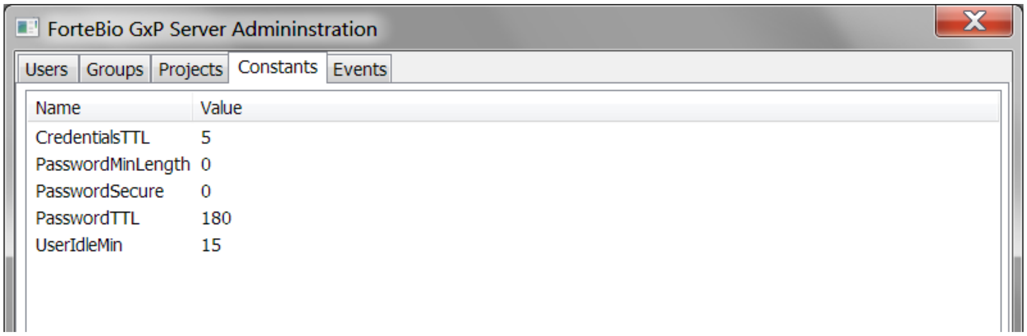

Figure B-44: *Constants Tab*

Available administrator constants and their associated value ranges are shown in Table B-3.

Table B-3: *Administrator Constants*

Constant	*Description*	*Default Value*	*Value Range*
**CredentialsTTL**	The number of days that the server settings are stored in the cache. This allows the software to operate in case the server is temporarily down.	5	Minimum=0, no maximum value
**PasswordMin-Length**	Minimum number of characters that a password must contain.	0	Minimum=0, no maximum value
**PasswordSecure**	Level of password complexity. Setting the constant to 0 has no password restrictions. Setting the constant to 1 requires passwords to contain at least one alpha, one numeric, and one punctuation character.	0	0–1
**PasswordTTL**	Amount of time that a password is allowed to remain unchanged.	180	Minimum=0, no maximum value
**UserIdleMin**	Idle time allowed during a user session after which the session is automatically closed.	15	Minimum=0, no maximum value

Creating a New Constant

To create a new constant:

1. Right-click anywhere in the **Constants** tab and select **New Constant**, or double-click in a blank area.

   The **New Constant** window displays (Figure B-45).

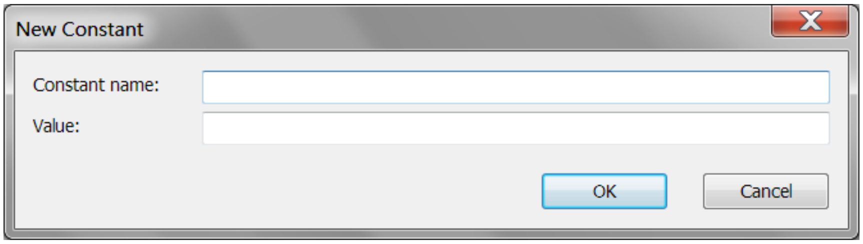

Figure B-45: *New Constant*

2. Enter the **Constant name** and **Value**. Refer to Table B-3 for a list of available constants and value ranges.
3. Click **OK** to save changes and exit.

Viewing and Changing Constants

To view and change constants:

1. Right-click the constant and select **Edit Constant**, or double-click the constant.

   The **Edit Constant** dialog box displays (Figure B-46).

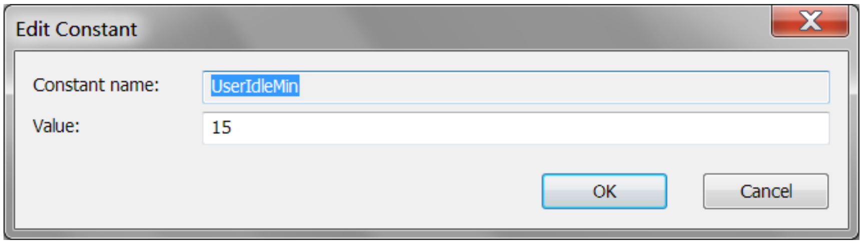

Figure B-46: *Edit Constant Dialog Box*

2. If needed, modify the constant settings. For more information on available constants and their values, see Table B-3 on page 202.
3. Click **OK** to save changes and exit.

Deleting a Constant

To delete a constant:

1. Right-click the constant and select **Delete Constant**.
2. Click **OK** in the dialog box displayed.

Event Log

The **Events** tab allows administrators to view all the user, system, and software event information recorded by the Pall ForteBio GxP Server module.

NOTE: *GxP Server module version 8.2 and higher will also display event information recorded in BLItz Pro software for BLItz systems.*

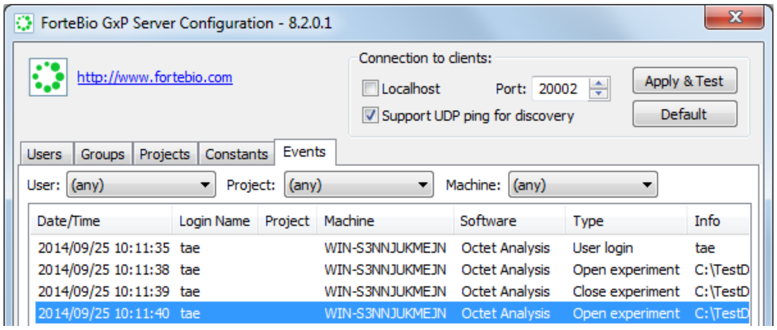

Figure B-47: *Events Tab*

Events are tracked for individual user accounts, projects and machines. By default, a historical log of all events recorded on the active Pall ForteBio GxP Server module displays:

- **Date and Time** - When the event occurred
- **Login Name** - User name associated with the event
- **Project** - Name of project associated with the event
- **Machine** - Name of instrument used (includes both Octet and BLItz instruments for GxP Server module versions 8.2 and higher)
- **Software** - Which software the event was logged in (available in GxP Server module versions 8.2 and higher only, includes Octet Data Acquisition, Octet Data Analysis and BLItz Pro software events)
- **Type** - Event type

NOTE: *In Octet Data Analysis 21 CFR Part 11 software versions 8.2 and higher, all events related to data analysis, processing and inclusion or exclusion of data are now also logged in the Audit Trail.*

- **Info** - Any additional information recorded with the event

Viewing Events

To view events for a specific user account, project, or computer, click the **User** (Figure B-48), **Project**, or **Machine** drop-down list, and select an entry:

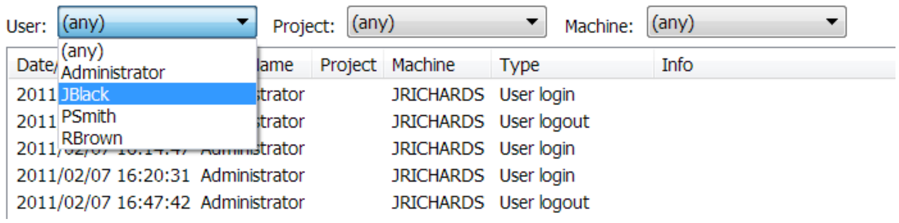

Figure B-48: *Viewing Events from the User Drop-Down List*

NOTE: *Selections can be made in either one or all of the **User**, **Project**, or **Machine** drop-down lists.*

The list then only displays events for the entries selected (Figure B-49).

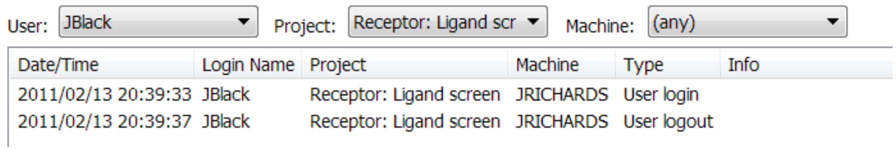

Figure B-49: *Selected Entries*

In addition to the specific user, project, and machine selections, the following list options are also available:

- **(any)**—Displays all user, project, or machine events.
- **(none)**—Displays all user and machine events not associated with a specific project (**Project** list only).

ACCESSING THE FORTEBIO GXP SERVER MODULE DIRECTLY

Administrators can directly access the Pall ForteBio GxP Server module without initiating an administrator user session. Direct access provides server testing options, as well as access to all administrative functions discussed earlier in this section.

To access the Pall ForteBio GxP Server module directly:

- ***If the*** Pall ForteBio ***GxP Server module is installed on a network location***—Double-click the **FBServerConfig.exe** file in the FBServer folder from the installed location (Figure B-50).

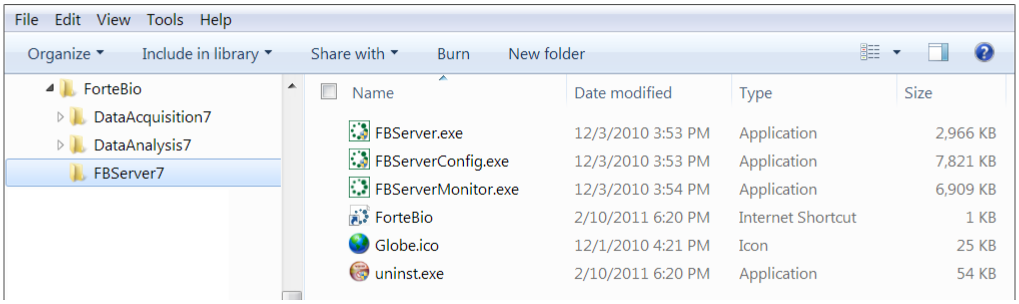

Figure B-50: *ForteBio GxP Server Module Installed on a Network Location*

- ***If the GxP Server module is installed on a local host computer***—Double-click the ForteBio GxP Server desktop icon (Figure B-51).

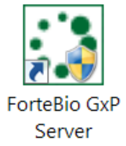

Figure B-51: *Pall ForteBio GxP Server Desktop Icon*

The **ForteBio GxP Server Configuration** window displays (Figure B-52).

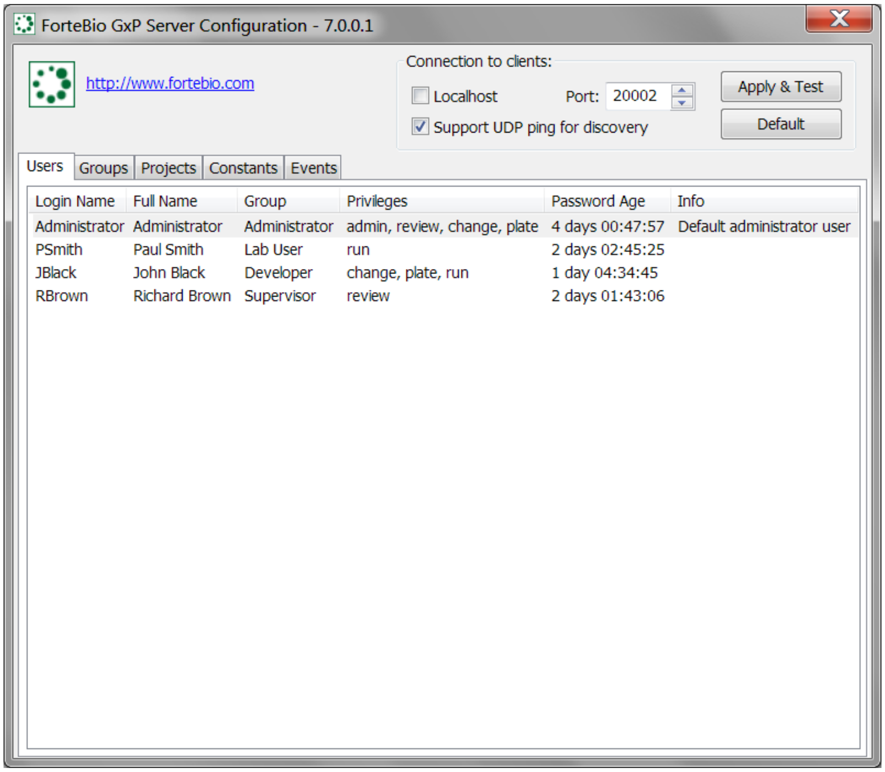

Figure B-52: *Pall ForteBio GxP Server Configuration Window*

Use of the **User**, **Groups**, **Projects**, **Constants**, and **Events** tabs are described in “Accessing Administrator Options” on page 190.

ForteBio GxP Server Module Testing

The Pall ForteBio GxP Server module can be tested to ensure it is accessible and functioning properly? Wasn't sure about this functionality.

To test the Pall ForteBio GxP Server module:

1. Optional. In the **Connections to Clients** box (Figure B-53), make changes to the server settings if necessary.

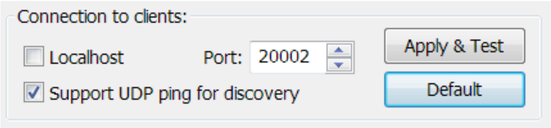

Figure B-53: *Connection to Clients*

2. Click **Apply & Test**.

   If the Pall ForteBio GxP Server module is found and functioning properly, the following message displays (Figure B-54):

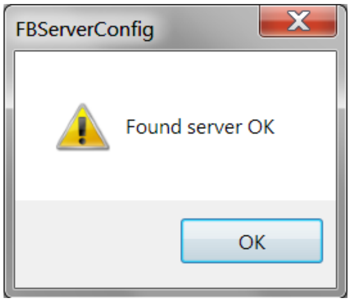

Figure B-54: *Message Confirmation of Found Server*

   To return to the originally configured Pall ForteBio GxP Server module settings, click **Default** at any time.

RESTARTING THE PALL FORTEBIO GXP SERVER MODULE

If the host location of the GxP Server module cannot be found during user login, or if you are unable to log in with valid credentials, the Pall ForteBio GxP Server module may be offline and must be restarted.

***NOTE:** Pall ForteBio recommends contacting your IT department to confirm whether or not network or firewall settings may have been changed. This may also be preventing access to the Pall ForteBio GxP Server module.*

To restart the ForteBio GxP Server module, choose one of the following two options:

- ***If the Pall ForteBio GxP Server module is installed on a network location***—Double-click the **FBServer.exe** file in the FBServer folder from the installed location (Figure B-55).

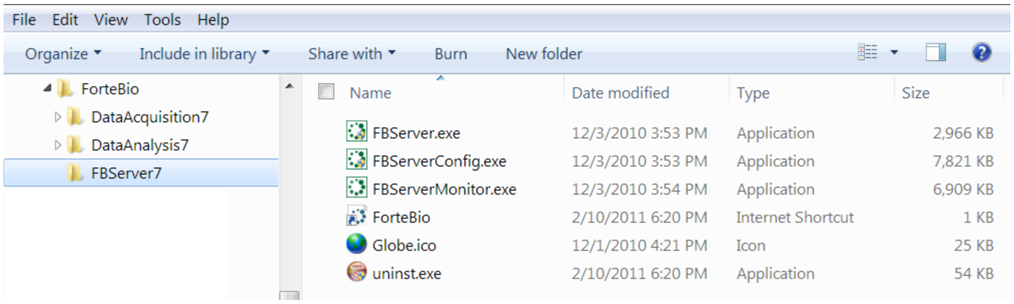

***Figure B-55:** ForteBio GxP Server Module Installed on a Network Location*

- ***If the GxP Server module is installed on a local computer***—Double-click the Restart Server desktop icon (Figure B-56).

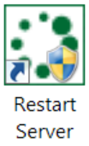

***Figure B-56:** Restart Server Desktop Icon*

The **Restart Server** console display momentarily as the Pall ForteBio GxP Server module restarts (Figure B-57).

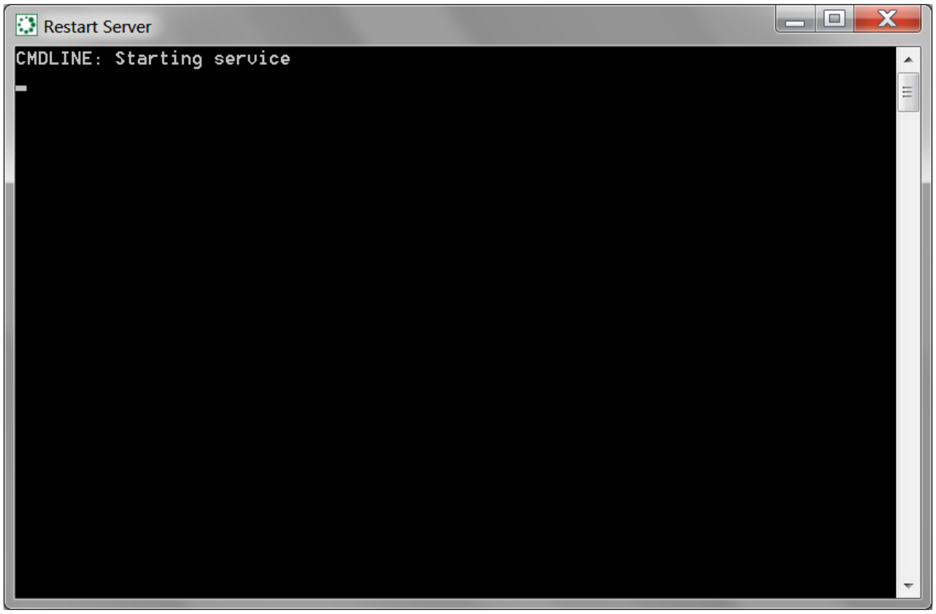

Figure B-57: *Restart Server Console*
